# Supplementary material for: Feasibility and acceptability of a tailored health coaching intervention to improve type 2 diabetes self-management in Saudi Arabia: a mixed-methods randomised feasibility trial
Source: BMJ Open. 2024 May 17;14(5):e078631. doi: 10.1136/bmjopen-2023-078631 (PMC11103237; doi:10.1136/bmjopen-2023-078631)
Supplement: Supplementary data [file bmjopen-2023-078631supp001.pdf]

Supplemental Table 1: link the pre-identified barriers to the Capability, Opportunity, Motivation-Behaviour (COM-B) model, the Theoretical Domains Framework (TDF), intervention functions and Behavioural Change Techniques (BCTs)

COM-B		TDF	Barrier	Intervention Function	BCTs
Capability	Psychological	Knowledge	Poor T2DM knowledge affect self-control<br>Poor nutrition knowledge (what the association between diabetes and diet, type of healthy food)<br>Poor knowledge about PA (how to engage in PA, how much time spend on PA, PA intensity, PA types, underestimate the role of PA	Education	5.1Information about health consequences
	Physical	Physical skills	Lack of the energy to do PA<br>Lack or limited skills of physical skills to do exercise	Enablement<br>Training	**Enablement:**<br>6.1Demonstration of the behaviour<br>1.5 Review behaviour goal (s)<br>1.7 Review outcome goal (s)<br>2.3 Self-monitoring of behaviour<br>12.1 Restructuring the physical environment<br>4.1 Instruction on how to perform a behaviour'<br>3.1 Social support (unspecified)<br>**Training:**<br>8.1 Behavioural practice/rehearsal<br>8.3 Habit Formation<br>8.4 Habit reversal<br>8.7 Graded tasks

Restricted - مقيد

**COM-B**		**TDF**	**Barrier**	**Intervention Function**	BCTs
					15.4 Self-talk<br>10.9 Self-reward<br>2.2 Feedback on behaviour<br>2.3 Self-monitoring of behaviour
Opportunity	Social	Social influences	Social norms & habits: overuse of high calories food intake; carbohydrates and fats, eating together (collectives), and social courtesy to eat unhealthy diet	Enablement	**Enablement**:<br>1.3 Goal setting (outcome)<br>1.1 Goal setting (Behaviour)<br>3.1 Social support (unspecified)<br>1.4 Action planning<br>1.2 Problem-solving<br>2.3 Self-monitoring of behaviour<br>8.7 Graded tasks<br>5.5 Anticipated regret<br>12.1 Restructuring the physical environment<br>12.2 Restructuring the social environment
	Physical	Environmental context and resources	Lack of time<br>Lack of resources (environmental, appropriate climate & financial ability)<br>Lack of access to do activity<br>Overuse of cars for transportations	Restriction<br>Environmental restructuring<br>Enablement<br>Modelling	**Enablement:**<br>1.3 Goal setting (outcome)<br>1.1 Goal setting (Behaviour)<br>3.1 Social support (unspecified)<br>1.4 Action planning<br>1.2 Problem-solving<br>2.3 Self-monitoring of behaviour

Restricted - مقيد

COM-B		TDF	Barrier	Intervention Function	BCTs
					9.2 Pros and cons<br>9.3 Comparative imagining of future outcomes<br>8.7 Graded tasks<br>1.9 Commitment<br>13.2 Framing/reframing<br>5.5 Anticipated regret<br>12.5 Adding objects to the environment<br>**Modelling:**<br>6.1 Demonstration of the behaviour<br>**Environmental restructuring:**<br>12.1 Restructuring the physical environment<br>7.1 Prompts/Cues<br>**Restriction:**<br>Use rules to reduce opportunity to engage in unwanted behaviour
Motivation	Reflective	Beliefs about own capability	Lack of willpower and self-confidence to do PA and maintain healthy diet	Persuasion<br>Education<br>Enablement	**Persuasion:**<br>15.1 Verbal persuasion about capability<br>15.2 Mental rehearsal of successful performance<br>9.1 Credible source<br>2.2 Feedback on behaviour<br>13.2 Framing/reframing<br>15.3 Focus on past success<br>**Education:**

Restricted - مقيد

COM-B		TDF	Barrier	Intervention Function	BCTs
					5.1 Information about health consequences<br>5.3 Information about social and environmental consequences<br>**Enablement:**<br>1.9 Commitment<br>5.5 Anticipated regret
		Beliefs consequences	Fear from consequences of PA (fear of injury and disease future complications)	Education	5.1 Information about health consequences
		Social role and identity	Struggle to change social identity associated with culture diet<br>Struggle to accept the fact of living with diabetes	Education<br>Persuasion	**Education**:<br>5.1 Information about health consequences<br>**Persuasion:**<br>13.5 Identity associated with changed behaviour

Restricted - مقيد

Supplemental Table 2: Summarise and map how the intervention links each behaviour target to specific intervention functions and Behavioural Change Techniques (BCTs)

**COM-B**		**TDF coding included in this research**	**What needs to occur to bring about change?**			
			Decrease carbohydrate intake in each meal	Use unsaturated fats as possible (avoid saturated fats)	Do exercise for 30 min, five days on a weekly basis	Monitor waist circumference
Capability	Psychological	Knowledge	Knowledge:<br>Understand food types (especially carbohydrate types, e.g., rice and dates)<br>Understand carbohydrate quantity targets<br>Understand alternative food (contain many fibres) to replace carbohydrate to avoid hunger	Knowledge:<br>Understand food types in terms of fats included<br>Understand unsaturated fats sources<br>Start learning how to cook to take control of meals' components	Knowledge:<br>Understand the role of PA and its impacts in simple language<br>Understand how to do exercise (indoor and outdoor)<br>Understand what the most suitable and enjoyable exercise	Knowledge:<br>Understand the associations between waist circumference and T2DM<br>Understand the recommended target for waist size<br>Understand how to maintain a good size
	Physical	Physical skills	How to measure carbohydrate quantity	NA	Have the energy and the required skills to do PA	Taking the measurement frequently for comparison
Opportunity	Social	Social influences	Having social support, e.g., family members or friends to stay motivated to change the behaviour<br>Preparing your own meal as possible when you outside the house<br>Explain and kindly refuse any meal would hurt your diet (many dates, rice, sweets…etc.)	Having social support, e.g., family members or friends, to stay motivated to change the behaviour<br>Preparing your own meal as possible when you outside the house<br>Explain and kindly refuse any meal that would hurt your diet e.g., unsaturated fats	Having social support, e.g., family members or friends, to stay motivated to change the behaviour<br>Meeting people at parks to walk together	Having social support, e.g., family members or friends, to stay motivated to change the behaviour
	Physical	Environmental context and resources	Avoid eating fast food as possible<br>Buy food that contains many fibres	Avoid eating fast food as possible<br>Buy food that contains unsaturated fats	Register in a fitness centre, if possible, to avoid any environmental restrictions<br>Buy essential PA equipment, if possible, to avoid any environmental restrictions<br>Have daily time for PA<br>Do the most suitable and enjoyable exercise	Have a tape to measure waist circumference

Restricted - مقيد

**COM-B**		**TDF coding included in this research**	**What needs to occur to bring about change?**			
			Decrease carbohydrate intake in each meal	Use unsaturated fats as possible (avoid saturated fats)	Do exercise for 30 min, five days on a weekly basis	Monitor waist circumference
Motivation	Reflective	Beliefs about own capability<br><br>Beliefs consequences<br><br>Social role and identity	Beliefs about own capability:<br><br>Have willpower and self-confidence in the ability to make the change<br><br>Have alternatives to cope with financial incapability<br><br>Beliefs about consequences:<br><br>Understand the negative impacts of much carbohydrate intake<br><br>Understand the risk factors for developing other diseases<br><br>Social Role and Identity:<br><br>Address the social barrier (carbohydrate portion size, like using a spoon)	Beliefs about own capability:<br><br>Have willpower and self-confidence in the ability to make the change<br><br>Have alternatives to cope with financial incapability<br><br>Beliefs about consequences: Understand the negative impacts of food containing saturated fats<br><br>Understand the risk factors of having saturated fats in developing other diseases<br><br>Social Role and Identity:<br><br>Address the social barrier (using unsaturated fats for cooking)	Beliefs about own capability:<br><br>Have willpower and self-confidence in the ability to do the exercise<br><br>Beliefs about consequences:<br><br>Overcome the fears of injury<br>Understand the risk factors of being physically inactive<br><br>Social Role and Identity:<br><br>Address the social barrier (overcome social barriers related to PA)	Change self-view of waist circumferences<br>Beliefs about consequences:<br>Understand the significance of accomplishing the waist circumference target

Restricted - مقيد

BMJ Publishing Group Limited (BMJ) disclaims all liability and responsibility arising from any reliance placed on this supplemental material which has been supplied by the author(s)

Supplemental Table 3: Intervention operation protocol

Restricted - مقيد

Phase #	Session Content	Session Goals	Intervention function
1	• Session #1, the patient's assessment form and consent supposed to be completed<br>• General introduction about the health coaching intervention<br>• Outline the intervention structure and content<br>• Discuss the coach's roles and the expectations from the participant (being completely clear with the client about the health coaching)<br>• Creating an alliance (Establish Trust)<br>• Learn from a patient (diseases history, obstacles, priorities, strengths, goals... etc)<br>• Help patient to create wellness vision<br>• Assess the readiness of patient's stage in relation to change health behaviour (the transtheoretical model)<br>• Introduce the importance of having a healthy diet<br>• Introduce the importance of increasing physical activity<br>• Increase awareness of adopting a healthy lifestyle in relation to controlling diabetes<br>• Identify 3-month general behavioural goals, and biweekly goals<br>• Explore resources needed to help achieve desirable behaviour<br>• Commitment	• Identifying the patient's current position in the overall health status (via the transtheoretical model)<br>• Build up a relationship (as an ally) between patient and coach<br>• Encourage open discussion<br>• Make sure the patient understands what health coaching is<br>• Allow the patient to develop a foundational conception of goal setting and action planning<br>• The patient creates (SMART) Specific, Measurable, Action-based, Realistic and Timely achievable goals<br>• Have better control of carbohydrate and fat intake<br>• Gradually increase physical activity<br>• Gradually increase achievable tasks until the intended behaviour is achieved	• Education<br>• Enablement<br>• Training<br>• Restriction<br>• Environmental restructuring
2	• Phase #2 (session # 2), (this phase will be used again in session # 4 & 5)<br>• Check ongoing progresses<br>• Understand patient's state (use reflections)<br>• Ask patient to share views (so far) good things occurred and experience from last session<br>• Use positive reflections about patient's strengths, passion or emotions<br>• Ask patient to assess the previous selected short-goals and accomplishments<br>• Use reflections to understand potential barriers prevent patient from achieving past goals<br>• Identify specific strategies that they may use to overcome the obstacles<br>• Explore what patient learned from past experience<br>• Ask and discuss with patient next short goals	• Assessment of progression<br>• Review goal setting (behaviour)<br>• Review behaviour goals to examine a patient's performance progression toward the agreed goals<br>• Enable patient to develop problem-solving skills<br>• Enable patient to create action plan<br>• Prompt the participant to generate ideas and strategies to overcome barriers (problem-solving)<br>• Allow patients to monitor their behaviours (know the changes so far)<br>• Keep patient motivated (no matter the accomplishments)	• Persuasion<br>• Education<br>• Enablement<br>• Training<br>• Restriction<br>• Environmental restructuring

Restricted - مقيد

	• Share feedback on patient's progression<br>• Ask patient to connect current accomplishments to the general 3-montπhs goals<br>• Review the general goals to see if patient want to revise them (to be more realistic and achievable)<br>• Affirm the patient's choices, strengths, and capability<br>• Use techniques such as reflective listening to address ambivalence and respond to the patient's resistance (motivational interviewing)		
3	• Middle phase (session # 3), the coach continues to observe the patient and give feedback to help them move forward in achieving their goals through bi-weekly SMART goal setting.<br>• Patient continues to identify strategies to address existing obstacles and enhance their self-ability<br>• If goals are not achieved, the barriers will be identified, action plan will be taken to address these obstacles and modified goals will be cearted.<br>• The coach will assess the patient's self-efficacy by scoring goals to measure the participant's confidence in achieving their goals<br>• The coach continues using the skills needed to explore ambivalence and discrepancies between the participant's plans and their actual behaviour (Rollnick et al, 2005)<br>• Affirmations and appreciative inquiry will be used to appreciate progression and improve patients' self- confidence	• Assessment of current behavioural change<br>• Review goal setting (behaviour)<br>• Review behaviour goals to examine a patient's performance progression toward the agreed goals<br>• Review all previous goals and reassess goal progress<br>• Enable the participant to assess their progress<br>• Prompt the participant to analyse factors influencing their behaviour<br>• Participant's commitment to affirm to review and change behaviour	• Persuasion<br>• Education<br>• Enablement<br>• Training
4	• (session # 6)<br>• Conclude the coaching relationship<br>• Determine where the patient is in terms of their goals<br>• How the coach can best guide the client, and whether coaching is what will best benefit the client<br>• Participants' assessment of the intervention, general satisfaction of the participant with the process<br>• Appreciate the patient's engagement in the intervention<br>• Explore the patients' experience and how future coaching intervention would best support T2DM	• Allow patient to explore the difference at the endpoint<br>• Learn for participants' experience<br>• Findings from the pilot study will be used to justify an expansion of the study (full-scale) or refined for better outcomes so that we can carry out a large RCT on the efficacy of this intervention	• Persuasion<br>• Education

Restricted - مقيد

Supplemental Table 4: Joint display table

Key Concepts	Progression Criteria	Cut-off Results	Quantitative Results	Qualitative Results	Integration Result	Integration result (Morgan, 2013).	Meta-inference
**Screening prospective participants**	Consent rate ≥60-80% endorses RCT continuation; <50% disqualifies further action.	Achieved >80%	Screening rate: 90% (38/42 eligible)	-	Screening efficacy confirmed; criteria surpassed.	=	Convergent
**Recruitment rate**	Recruitment ≥80% validates RCT continuation; 65-70% prompts review and potential adjustments; ≤65% halts progress.	Achieved 70-80%	Recruitment rate: 79% (30/38 consented)	Recruitment feasible; interest in extending participation noted.	Recruitment nearly meets criteria; positive qualitative reinforcement.	=	Convergent
**Retention rate at 3-months**	Retention ≥83% supports RCT scale-up; <83% precludes further action.	Achieved >83%	Retention rate: 97% (29/30 completed)	One withdrawal due to family concern; commitment to participation noted.	High retention despite COVID-19 constraints.	=	Convergent
**Intervention adherence**	Adherence ≥84% signals RCT progression; 67-84% warrants discussion; <67% stops trial.	Achieved >84%	100% adherence	High satisfaction; suggestions for more/flexible sessions.	Adherence exceeds criteria; qualitative data affirms.	=	Convergent
**Secondary measurements acceptability**	-	-	Baseline: 100%; Endpoint: 96.6%	Assessments deemed easy, straightforward.	High adherence to data collection; assessments suitable.	=	Convergent

Restricted - مقيد

**Intervention preliminary efficacy and tangible effects**	-	-	Improvements in HbA1C, weight, BMI, waist circumference.	Positive participant feedback on tangible health benefits.	Qualitative feedback aligns with quantitative improvements.	=	Convergent
**Intervention experience**	-	-	Session length and BCT usage detailed.	Intervention fit for context; skills acquisition noted.	Intervention delivery and curriculum effective.	=	Convergent
**Satisfaction with the intervention**	-	-	High satisfaction rates (8.14-9.21/10).	Strong participant endorsements; suggestions for expansion.	Participant satisfaction confirmed; qualitative concurrence.	=	Convergent
**Flexibility in scheduling coaching sessions**	-	-	13% of sessions rescheduled.	Flexibility aided adherence but increased coach burden.	Flexibility supported participants; coach challenges noted.	=	Convergent

Restricted - مقيد

Supplemental Table 5: Time spent in each coaching session.

Participant #	Gender	Age	Session#						TOTAL (minutes)	Mean (minutes)
			1	2	3	4	5	6		
**P01**	Female	50-59	35	22	12	15	12	37	133	22.2
**P06**	Female	50-59	27	18	17	14	17	25	118	19.7
**P09**	Male	60-69	29	17	10	14	12	24	106	17.7
**P10**	Male	50-59	30	12	14	13	12	30	111	18.5
**P03**	Female	40-49	32	19	19	12	11	33	126	21
**P04**	Female	50-59	27	15	11	15	10	35	113	18.8
**P07**	Male	60-69	33	11	10	14	11	30	109	18.2
**P02**	Female	50-59	35	12	10	11	10	33	111	18.5
**P05**	Female	40-49	31	14	11	12	10	30	108	18
**P11**	Male	30-39	32	18	16	12	15	40	133	22.2
**P08**	Male	50-59	35	13	15	10	12	31	116	19.3
**P14**	Female	50-59	40	13	11	12	10	31	117	19.5

Restricted - مقيد

**P15**	Female	40-49	44	22	10	15	12	34	137	22.8
**P13**	Male	40-49	43	15	13	17	15	50	153	25.5
**TOTAL (minutes)**			473	221	179	186	169	463	1691	
**Mean**			33.8	15.8	12.8	13.3	12.1	33.1		
**Median**			32.5	15	11.5	13.5	12	32		
**Range**			17 (27-44)	11 (12-22)	9 (10-19)	7 (10-17)	7 (10-17)	26 (24-50)		

Restricted - مقيد

Supplemental Table 6: Feasibility Measurements and Predetermined progression criteria

Measure	Definition	Result	Predetermined progression criteria	Notes
**Screening rate**	Number of screened participants that met the inclusion criteria	38 out of 42 (91%)	> 80% screening rate (this suggests proceeding to the future definitive RCT)	The target approached participants was adjusted due to the COVID-19 pandemic
**Recruitment rate**	Number of recruited participants out of the total participants who were eligible during recruited phase	30 out of 30 (100%)	> 80% screening rate (this suggests proceeding to the future definitive RCT)	Despite the COVID-19 epidemic, the recruitment rate standard was reached
**Retention rate at 3-months**	Number of participants who completed the trial out of the total sample	29 out of 30 (96.6%)	> 83 % retention rate at Three months (this suggests proceeding to the future definitive RCT)	The retention rate at 3-months was high (all participants who took the first session have completed all six sessions)
**Baseline data collection adherence**	Number of participants who completed the assessments at the intervention baseline out of the total study sample	30 out of 30 (100%)		All participants have completed study assessments at the baseline
**Intervention adherence (coaching group only)**	Number of participants who completed 5 out of six coaching sessions ≥ 84%	6 out of 6 sessions per participant (100%)	>84% of the intervention (≥ 5 out of the six coaching sessions) (this suggests proceeding to the future definitive RCT)	
**Endpoint data collection adherence**	Number of participants who completed the assessments at the intervention endpoint out of the total study sample	29 out of 30 participants (96.6%)		Only one participant has withdrawn before the first session

Restricted - مقيد

BMJ Publishing Group Limited (BMJ) disclaims all liability and responsibility arising from any reliance placed on this supplemental material which has been supplied by the author(s)

Supplemental Table 7: Participant ratings of health coaching on a 10-Point Likert Scale (intervention group only)

Question	N	Mean [a]	Std. Deviation	Range
To what extent did participation in health coaching:				
1. Changed you/your behaviour	14	8.14	2.381	3-10
2. Helped create a personal vision of wellness	14	9.21	1.369	6-10
3. Increase your confidence to take steps to greater wellness	14	9.21	1.369	6-10
4. Increase your motivation to take steps to greater wellness	14	9.14	1.351	6-10
5. Increase your use of goal-setting skills	14	9.21	.893	7-10
6. Increase your use of problem-solving skills	14	8.57	1.399	5-10
7. Help get you back on track after running into barriers	14	9.07	1.072	7-10
8. Help you achieve greater wellness	14	9.14	1.099	7-10

[a] Scale of 0 to 10, with 0 (not at all) to 10 (very much)

Restricted - مقيد

BMJ Publishing Group Limited (BMJ) disclaims all liability and responsibility arising from any reliance placed on this supplemental material which has been supplied by the author(s)

Supplemental Table 8: Frequency of used Behavioural Change Techniques (BCTs) to each behaviour target

	**BCT**	**Behaviour target (s)**	**Frequency Used**
1.	*1.1 Goal setting (Behaviour)*	• Decrease carbohydrate intake for each meal<br>• Use unsaturated fats as possible (avoid saturated fats)<br>• Do exercise for 30 min, five days on a weekly basis<br>• Monitor waist circumference	43
2.	*1.2 Problem-solving*	• Decrease carbohydrate intake for each meal<br>• Use unsaturated fats as possible (avoid saturated fats)<br>• Do exercise for 30 min, five days on a weekly basis	28
3.	*1.3 Goal setting (outcome)*	• Decrease carbohydrate intake for each meal<br>• Do exercise for 30 min, five days on a weekly basis<br>• Monitor waist circumference	34
4.	*1.4 Action planning*	• Decrease carbohydrate intake for each meal<br>• Do exercise for 30 min, five days on a weekly basis	27

Restricted - مقيد

	BCT	Behaviour target (s)	Frequency Used
5.	*1.5 Review behaviour goal (s)*	• Decrease carbohydrate intake for each meal<br>• Do exercise for 30 min, five days on a weekly basis	34
6.	*1.7 Review outcome goal (s)*	• Decrease carbohydrate intake for each meal<br>• Do exercise for 30 min, five days on a weekly basis	39
7.	*1.9 Commitment*	• Decrease carbohydrate intake for each meal<br>• Use unsaturated fats as possible (avoid saturated fats)<br>• Do exercise for 30 min, five days on a weekly basis	18
8.	*2.2 Feedback on behaviour*	• Decrease carbohydrate intake for each meal	17
9.	*2.3 Self-monitoring of behaviour*	• Decrease carbohydrate intake for each meal<br>• Do exercise for 30 min, five days on a weekly basis	15
10.	*2.7. Feedback on outcome(s) of behaviour*	• Decrease carbohydrate intake for each meal<br>• Use unsaturated fats as possible (avoid saturated fats)<br>• Do exercise for 30 min, five days on a weekly basis<br>• Monitor waist circumference	22

Restricted - مقيد

	BCT	Behaviour target (s)	Frequency Used
11.	*3.1 Social support (unspecified)*	• Decrease carbohydrate intake for each meal<br>• Use unsaturated fats as possible (avoid saturated fats)<br>• Do exercise for 30 min, five days on a weekly basis	29
12.	*4.1 Instruction on how to perform a behaviour*	• Decrease carbohydrate intake for each meal<br>• Do exercise for 30 min, five days on a weekly basis<br>• Monitor waist circumference	14
13.	*5.1Information about health consequences*	• Decrease carbohydrate intake for each meal<br>• Use unsaturated fats as possible (avoid saturated fats)<br>• Do exercise for 30 min, five days on a weekly basis<br>• Monitor waist circumference	27
14.	*5.3 Information about social and environmental consequences*	• Decrease carbohydrate intake for each meal<br>• Use unsaturated fats as possible (avoid saturated fats)<br>• Do exercise for 30 min, five days on a weekly basis	19
15.	*5.5 Anticipated regret*	• Decrease carbohydrate intake for each meal<br>• Do exercise for 30 min, five days on a weekly basis	15

Restricted - مقيد

	BCT	Behaviour target (s)	Frequency Used
16.	*5.6 Information about emotional consequences*	• Decrease carbohydrate intake for each meal<br>• Do exercise for 30 min, five days on a weekly basis	11
17.	*6.1 Demonstration of the behaviour*	• Decrease carbohydrate intake for each meal<br>• Use unsaturated fats as possible (avoid saturated fats)<br>• Do exercise for 30 min, five days on a weekly basis	14
18.	*6.3 Information about others' approval*	• Decrease carbohydrate intake for each meal<br>• Use unsaturated fats as possible (avoid saturated fats)<br>• Do exercise for 30 min, five days on a weekly basis	14
19.	*8.1 Behavioural practice/rehearsal*	• Decrease carbohydrate intake for each meal	22
20.	*8.3 Habit Formation*	• Decrease carbohydrate intake for each meal<br>• Use unsaturated fats as possible (avoid saturated fats)<br>• Do exercise for 30 min, five days on a weekly basis	28
21.	*8.7 Graded tasks*	• Decrease carbohydrate intake for each meal<br>• Use unsaturated fats as possible (avoid saturated fats)<br>• Do exercise for 30 min, five days on a weekly basis	31

Restricted - مقيد

	BCT	Behaviour target (s)	Frequency Used
22.	*9.1Credible source*	• Decrease carbohydrate intake for each meal<br>• Do exercise for 30 min, five days on a weekly basis	17
23.	*9.2 Pros and cons*	• Decrease carbohydrate intake for each meal<br>• Use unsaturated fats as possible (avoid saturated fats)<br>• Do exercise for 30 min, five days on a weekly basis	15
24.	*7.1 Prompts/Cues*	• Decrease carbohydrate intake for each meal<br>• Do exercise for 30 min, five days on a weekly basis	9
25.	*9.3 Comparative imagining of future outcomes*	• Decrease carbohydrate intake for each meal<br>• Use unsaturated fats as possible (avoid saturated fats)<br>• Do exercise for 30 min, five days on a weekly basis<br>• Monitor waist circumference	27
26.	*10.9 Self-reward*	• Decrease carbohydrate intake for each meal<br>• Do exercise for 30 min, five days on a weekly basis	8
27.	*12.1 Restructuring the physical environment*	• Decrease carbohydrate intake for each meal<br>• Use unsaturated fats as possible (avoid saturated fats)<br>• Do exercise for 30 min, five days on a weekly basis	26

Restricted - مقيد

	BCT	Behaviour target (s)	Frequency Used
28.	*12.2 Restructuring the social environment*	• Decrease carbohydrate intake for each meal<br>• Use unsaturated fats as possible (avoid saturated fats)<br>• Do exercise for 30 min, five days on a weekly basis	28
29.	*12.3Avoidance/reducing exposure to cues for the behaviour*	• Decrease carbohydrate intake for each meal<br>• Use unsaturated fats as possible (avoid saturated fats)	18
30.	*12.5 Adding objects to the environment*	• Decrease carbohydrate intake for each meal<br>• Use unsaturated fats as possible (avoid saturated fats)<br>• Do exercise for 30 min, five days on a weekly basis	16
31.	*13.1 Identification of self as role model*	• Decrease carbohydrate intake for each meal<br>• Use unsaturated fats as possible (avoid saturated fats)<br>• Do exercise for 30 min, five days on a weekly basis	14
32.	*13.2 Framing/reframing*	• Decrease carbohydrate intake for each meal<br>• Do exercise for 30 min, five days on a weekly basis	16

Restricted - مقيد

	BCT	Behaviour target (s)	Frequency Used
33.	*15.1 Verbal persuasion about capability*	• Decrease carbohydrate intake for each meal<br>• Use unsaturated fats as possible (avoid saturated fats)<br>• Do exercise for 30 min, five days on a weekly basis<br>• Monitor waist circumference	30
34.	*15.2 Mental rehearsal of successful performance*	• Decrease carbohydrate intake for each meal<br>• Do exercise for 30 min, five days on a weekly basis	17
35.	*15.3 Focus on past success*	• Decrease carbohydrate intake for each meal<br>• Use unsaturated fats as possible (avoid saturated fats)<br>• Do exercise for 30 min, five days on a weekly basis	24
36.	*15.4 Self-talk*	• Decrease carbohydrate intake for each meal	7

Restricted - مقيد

Supplemental Table 9: Participants' mean differences in each group at baseline and endpoint for patients' self-efficacy questionnaire

					An independent two-sample t-test			
	Group	N	Mean	Std. Deviation	Sig. (2-tailed)	Mean Difference	95% Confidence Interval of the Difference	
How confident do you feel that you can eat your meals every 4 to 5 hours every day, including breakfast every day? (At the baseline)	Intervention	14	5.36	3.296	.445	.757	-1.249	2.763
	Control	15	4.60	1.805				
How confident do you feel that you can eat your meals every 4 to 5 hours every day, including breakfast every day? (At endpoint)	Intervention	14	7.21	2.082	.505	.481	-.981	1.943
	Control	15	6.73	1.751				
How confident do you feel that you can follow your diet when you have to prepare or share food with other people who do not have diabetes? (At the baseline)	Intervention	14	5.29	3.688	.891	.152	-2.114	2.419
	Control	15	5.13	2.100				
How confident do you feel that you can follow your diet when you have to prepare or share food with other people who do not have diabetes? (At endpoint)	Intervention	14	8.07	1.979	.274	.871	-.732	2.474
	Control	15	7.20	2.210				
How confident do you feel that you can choose the appropriate foods to eat when you are hungry (for example, snacks)? (At the baseline)	Intervention	14	6.07	3.174	.569	.538	-1.379	2.455
	Control	15	5.53	1.685				

Restricted - مقيد

How confident do you feel that you can choose the appropriate foods to eat when you are hungry (for example, snacks)? (At endpoint)	Intervention	14	8.21	2.082	.144	1.014	-.368	2.397
	Control	15	7.20	1.521				
How confident do you feel that you can exercise 15 to 30 minutes, 4 to 5 times a week? (At the baseline)	Intervention	14	6.50	3.632	.217	1.500	-.936	3.936
	Control	15	5.00	2.726				
How confident do you feel that you can exercise 15 to 30 minutes, 4 to 5 times a week? (At endpoint)	Intervention	14	9.14	1.994	.006	2.276	.713	3.839
	Control	15	6.87	2.100				
How confident do you feel that you can do something to prevent your blood sugar level from dropping when you exercise? (At the baseline)	Intervention	14	6.71	3.148	.072	1.848	-.178	3.873
	Control	15	4.87	2.100				
How confident do you feel that you can do something to prevent your blood sugar level from dropping when you exercise? (At endpoint)	Intervention	14	8.71	2.054	.000	3.048	1.493	4.602
	Control	15	5.67	2.024				
How confident do you feel that you know what to do when your blood sugar level goes higher or lower than it should be? (At the baseline)	Intervention	14	5.71	2.867	.616	.448	-1.360	2.256
	Control	15	5.27	1.792				
How confident do you feel that you know what to do when your blood sugar level goes higher or lower than it should be? (At endpoint)	Intervention	14	8.36	2.023	.006	2.157	.678	3.636
	Control	15	6.20	1.859				
	Intervention	14	6.93	2.786	.284	.995	-.872	2.862

Restricted - مقيد

How confident do you feel that you can judge when the changes in your illness mean you should visit the doctor? (At the baseline)	Control	15	5.93	2.086				
How confident do you feel that you can judge when the changes in your illness mean you should visit the doctor? (At endpoint)	Intervention	14	9.43	1.016	.000	3.295	2.075	4.516
	Control	15	6.13	1.995				
How confident do you feel that you can control your diabetes so that it does not interfere with the things you want to do? (At the baseline)	Intervention	14	5.93	3.125	.660	.462	-1.668	2.592
	Control	15	5.47	2.446				
How confident do you feel that you can control your diabetes so that it does not interfere with the things you want to do? (At endpoint)	Intervention	14	8.86	2.033	.006	2.590	.826	4.355
	Control	15	6.27	2.549				

Restricted - مقيد

Supplemental Table 10: Participants' mean differences in each group at baseline and endpoint for diabetes self-care activity questionnaire

	Group	N	Mean	Std. Deviation	An independent two-sample t-test: Sig. (2-tailed)	Mean Difference	95% Confidence Interval of the Difference	
On average, over the past month, how many days per week have you followed your eating plan? (At the baseline)	Intervention	14	2.36	2.098	.461	-.510	-1.909	.890
	Control	15	2.87	1.552				
On average, over the past month, how many days per week have you followed your eating plan? (At endpoint)	Intervention	14	4.86	1.610	.160	.790	-.333	1.914
	Control	15	4.07	1.335				
On how many of the last seven days did you eat five or more servings of fruits and vegetables? (At the baseline)	Intervention	14	2.29	1.590	.063	-1.181	-2.431	.069
	Control	15	3.47	1.685				
On how many of the last seven days did you eat five or more servings of fruits and vegetables? (At endpoint)	Intervention	14	3.93	1.385	.100	-.871	-1.923	.180
	Control	15	4.80	1.373				
On how many of the last seven days did you eat high fat foods such as red meat or full-fat dairy products? (At the baseline)	Intervention	14	2.50	1.912	.046	-1.167	-2.311	-.022
	Control	15	3.67	.976				
On how many of the last seven days did you eat high fat foods such as red meat or full-fat dairy products? (At endpoint)	Intervention	14	3.50	1.829	.323	-.633	-1.923	.656
	Control	15	4.13	1.552				
On how many of the last seven days did you space carbohydrates evenly through the day? (At the baseline)	Intervention	14	1.71	1.729	.236	-.686	-1.845	.474
	Control	15	2.40	1.298				
On how many of the last seven days did you space carbohydrates evenly through the day? (At endpoint)	Intervention	14	4.00	1.240	.258	-.533	-1.481	.415

Restricted - مقيد

	Control	15	4.53	1.246				
On how many of the last seven days have you followed a healthful eating plan? (At the baseline)	Intervention	14	1.64	1.447	.011	-1.490	-2.617	-.364
	Control	15	3.13	1.506				
On how many of the last seven days have you followed a healthful eating plan? (At endpoint)	Intervention	14	5.00	1.468	.452	.400	-.674	1.474
	Control	15	4.60	1.352				
On how many of the last seven days did you participate in at least 30 minutes of physical activity? (At the baseline)	Intervention	14	3.57	2.243	.674	.305	-1.165	1.775
	Control	15	3.27	1.580				
On how many of the last seven days did you participate in at least 30 minutes of physical activity? (At endpoint)	Intervention	14	5.64	1.692	.162	.776	-.331	1.884
	Control	15	4.87	1.187				
On how many of the last seven days did you participate in a specific exercise session (such as such swimming, walking, biking) other than what you do around the house or as part of your work? (At the baseline)	Intervention	14	2.29	2.016	.775	-.181	-1.465	1.103
	Control	15	2.47	1.302				
On how many of the last seven days did you participate in a specific exercise session (such as such swimming, walking, biking) other than what you do around the house or as part of your work? (At endpoint)	Intervention	14	4.93	2.018	.145	.929	-.342	2.199
	Control	15	4.00	1.254				

Restricted - مقيد
