# Supplementary figures and images for: Feasibility and acceptability of a tailored health coaching intervention to improve type 2 diabetes self-management in Saudi Arabia: a mixed-methods randomised feasibility trial
Source: BMJ Open. 2024 May 17;14(5):e078631. doi: 10.1136/bmjopen-2023-078631 (PMC11103237; doi:10.1136/bmjopen-2023-078631)

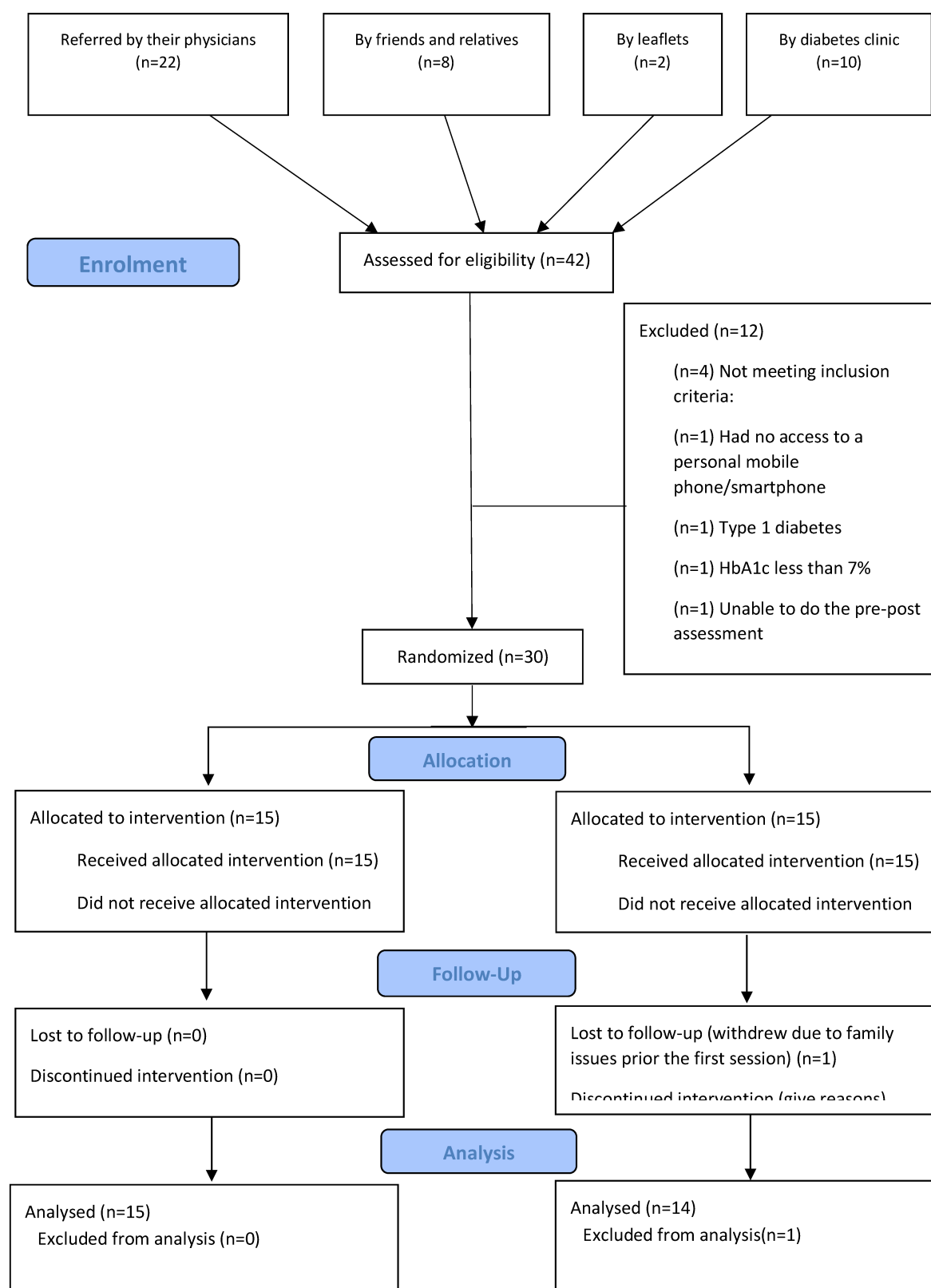

Figure 1: The study CONSORT flow chart

Supplement: Supplementary data [file bmjopen-2023-078631supp002.pdf]

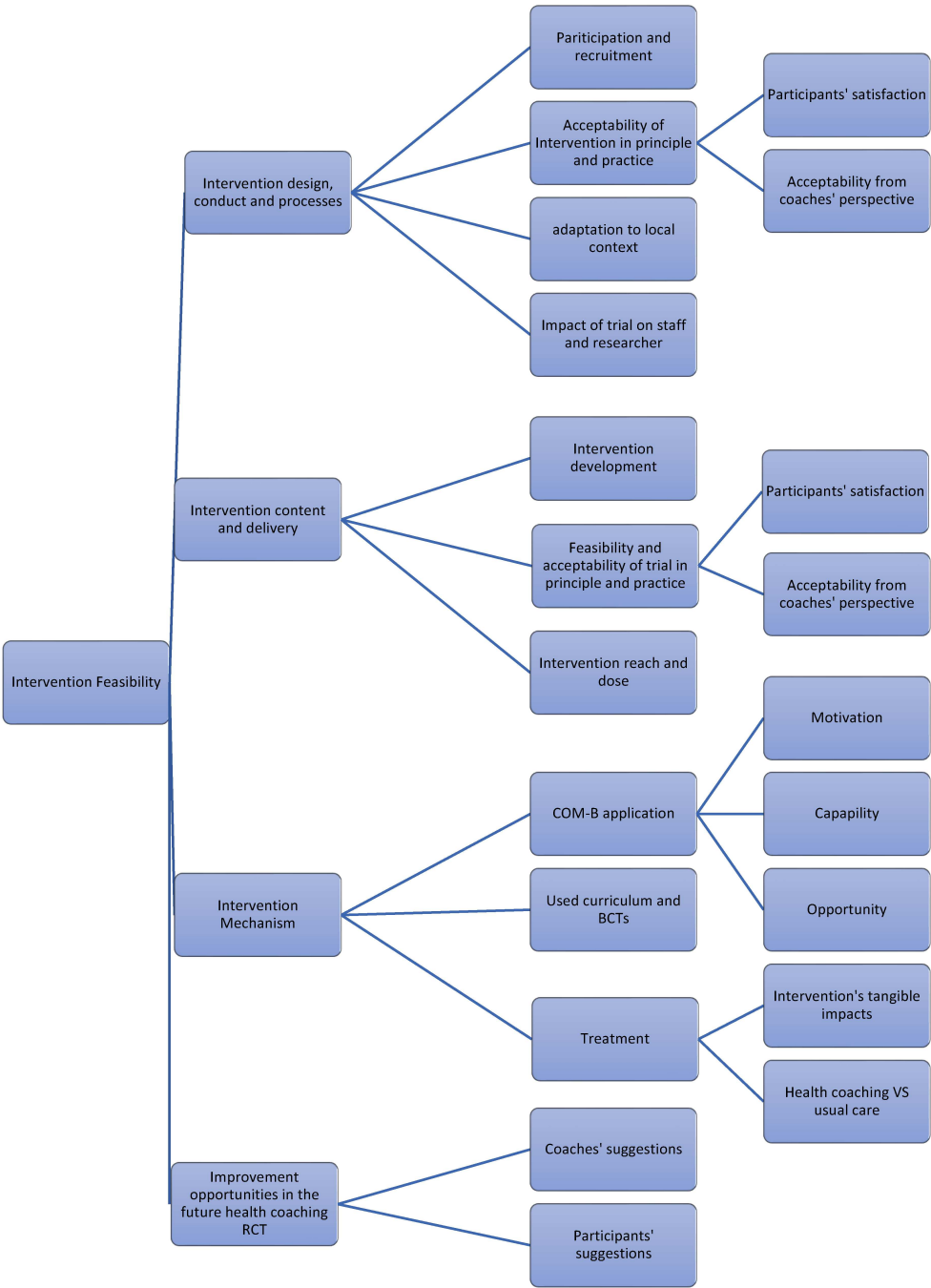

Figure 2: Thematic analysis map

Supplement: Supplementary data [file bmjopen-2023-078631supp003.pdf]
