# Supplementary material for: Feasibility and acceptability of a tailored health coaching intervention to improve type 2 diabetes self-management in Saudi Arabia: a mixed-methods randomised feasibility trial
Source: BMJ Open. 2024 May 17;14(5):e078631. doi: 10.1136/bmjopen-2023-078631 (PMC11103237; doi:10.1136/bmjopen-2023-078631)
Supplement: Author's manuscript [file bmjopen-2023-078631.draft_revisions.pdf]

BMJ Open is committed to open peer review. As part of this commitment we make the peer review history of every article we publish publicly available.

When an article is published we post the peer reviewers' comments and the authors' responses online. We also post the versions of the paper that were used during peer review. These are the versions that the peer review comments apply to.

The versions of the paper that follow are the versions that were submitted during the peer review process. They are not the versions of record or the final published versions. They should not be cited or distributed as the published version of this manuscript.

BMJ Open is an open access journal and the full, final, typeset and author-corrected version of record of the manuscript is available on our site with no access controls, subscription charges or pay-per-view fees (http://bmjopen.bmj.com).

If you have any questions on BMJ Open's open peer review process please email info.bmjopen@bmj.com

BMJ Open

Feasibility and Acceptability of Tailored Health Coaching Intervention to Improve Type 2 Diabetes Self-Management in Saudi Arabia: A Mixed-Methods Randomised Controlled Trial

Journal:	*BMJ Open*
Manuscript ID	bmjopen-2023-078631
Article Type:	Original research
Date Submitted by the Author:	07-Aug-2023

Complete List of Authors:	Almulhim, Abdullah; The University of Sheffield, School of Medicine and Population Health; Saudi Electronic University, College of Health Sciences<br>Alhowaish, Atheer; King Fahad Medical City<br>Madani, Alaa; King Fahad Medical City<br>AlQaddan, Munirah; King Fahad Medical City<br>Altuwalah, Abdulaziz S.; King Fahad Medical City<br>Goyder, Elizabeth; The University of Sheffield, School of Medicine and Population Health

Keywords:	PUBLIC HEALTH, DIABETES & ENDOCRINOLOGY, Behavior

SCHOLARONE™
Manuscripts

I, the Submitting Author has the right to grant and does grant on behalf of all authors of the Work (as defined in the below author licence), an exclusive licence and/or a non-exclusive licence for contributions from authors who are: i) UK Crown employees; ii) where BMJ has agreed a CC-BY licence shall apply, and/or iii) in accordance with the terms applicable for US Federal Government officers or employees acting as part of their official duties; on a worldwide, perpetual, irrevocable, royalty-free basis to BMJ Publishing Group Ltd ("BMJ") its licensees and where the relevant Journal is co-owned by BMJ to the co-owners of the Journal, to publish the Work in this journal and any other BMJ products and to exploit all rights, as set out in our licence.

The Submitting Author accepts and understands that any supply made under these terms is made by BMJ to the Submitting Author unless you are acting as an employee on behalf of your employer or a postgraduate student of an affiliated institution which is paying any applicable article publishing charge ("APC") for Open Access articles. Where the Submitting Author wishes to make the Work available on an Open Access basis (and intends to pay the relevant APC), the terms of reuse of such Open Access shall be governed by a Creative Commons licence – details of these licences and which Creative Commons licence will apply to this Work are set out in our licence referred to above.

Other than as permitted in any relevant BMJ Author's Self Archiving Policies, I confirm this Work has not been accepted for publication elsewhere, is not being considered for publication elsewhere and does not duplicate material already published. I confirm all authors consent to publication of this Work and authorise the granting of this licence.

For peer review only

Feasibility and Acceptability of Tailored Health Coaching Intervention to Improve Type 2 Diabetes Self-Management in Saudi Arabia: A Mixed-Methods Randomised Controlled Trial

Abdullah N. Almulhim[1,2]*, Atheer Alhowaish[3], Alaa Madani[3], Munirah AlQaddan[3], Abdulaziz S. Altuwalah[3], Elizabeth Goyder[1]

1 School of Medicine and Population Health, University of Sheffield, Sheffield S1 4DA, UK

2 Public Health Department, College of Health Sciences, Saudi Electronic University, Riyadh 13316, Saudi Arabia

3 Health Education Administration, King Fahad Medical City, Riyadh 12231, Saudi Arabia

* Correspondence: aalmulhim@seu.edu.sa

Abstract

Background: Around a third of the population of Saudi Arabia have been diagnosed with Type 2 diabetes, a condition often requiring lifestyle changes. Personalised health coaching, a strategy developed to assist individuals in overcoming challenges to adopt healthy behaviours, has not yet been widely applied in the country.

Aims: To explore the feasibility and acceptability of tailored health coaching in Saudi Arabia, in order to aid those with Type 2 diabetes to more effectively managing their condition.

Methods: Using a mixed-methods approach, this research involved a randomised controlled trial with 30 Saudi adults who have Type 2 diabetes. They were randomly allocated into either the intervention or control arm for 12 weeks. The Capability, Opportunity, Motivation, and Behaviour framework was used to guide the intervention implementation along with the Behaviour Change Techniques Taxonomy version 1. The primary goal was to assess the suitability and duration of the intervention, recruitment, retention, and completion rates. The secondary outcome focused on the preliminary efficacy of the health coaching measured by the glycaemic index, blood pressure, body mass index, waist circumference, weight, patient self-efficacy, and diabetes self-management.

Results: The results showed high rates of eligibility, recruitment, and retention (a screening rate of 90%, a recruiting rate of 100%, and a retention rate of 100%), exceeding the predetermined progression criteria. The participants were satisfied with the intervention and requested its expansion.

Conclusion: The findings demonstrated positive outcomes, supporting the need for a larger randomised controlled trial to evaluate the efficacy of health coaching in improving diabetes self-management among individuals with Type 2 diabetes in Saudi Arabia.

Keywords: health behaviour change; health coaching; self-management; behaviour change techniques; type 2 diabetes

For peer review only

Summary box:

Strengths and limitations of this study:

- This is the first health coaching intervention to employ the COM-B model and BCTs taxonomy, addressing a significant knowledge gap. It is also the first study of this kind to be applied in Saudi Arabia.
- The study adhered to the CONSORT 2010 statement's recommended progression criteria, thereby supporting decision-making for future trials.
- The study achieved a comprehensive impact assessment of health coaching through both qualitative and quantitative data.
- The use of a mixed-methods approach bolstered the reliability and validity of the findings.
- The study's generalizability was limited due to its specific setting, small sample size, and lack of statistical power. Therefore, the findings may not apply to primary care settings or younger populations. This limitation arises from the fact that recruitment was done from a public hospital, focusing on an older adolescent demographic.

Introduction:

Type 2 Diabetes Mellitus (T2DM) is a chronic illness that is becoming more common globally and is a significant public health concern. Lifestyle factors, including obesity and physical inactivity, are crucial in the development of this disease[1]. T2DM can lead to complications such as cardiovascular disease, kidney failure, nerve damage, and ocular and auditory problems, significantly increasing the risk of heart disease and stroke in people with diabetes[2].

Managing diabetes can be a challenging task that encompasses various aspects of life, including social, economic, and healthcare domains[3]. To help T2DM patients effectively manage their condition, it is important to prioritise self-management[4]. T2DM is a prevalent chronic disease in Saudi Arabia (SA), with one of the highest rates in the Middle East and the seventh-highest globally[5]. Unhealthy diets, inactive lifestyles, and rapid urbanisation are all contributing factors to this concerning trend. [6]

Despite national efforts to control T2DM in the SA context, the disease continues to pose significant challenges[7]. While awareness campaigns are being employed, there is a notable difficulty among most T2DM patients in adopting healthier diets and lifestyles[7]. To address this issue, it is necessary to implement patient-centred approaches that prioritise the development of self-management skills rather than relying solely on education-based programs[8].

Health coaching, derived from various disciplines and behaviour change theories, has emerged as a client-centred approach gaining recognition in the field of health promotion. Although it has been widely explored in the literature and has shown promising results, this personalised one-on-one intervention has not yet been implemented within the healthcare systems of SA. Studies have demonstrated that health coaching, centred around patients' values, may offer additional benefits compared to traditional T2DM education programs[9]. By focusing on

For peer review only

enhancing patients' self-efficacy and essential skills such as goal-setting and problem-solving, health coaching can prove highly effective and financially viable[10]. Its successful application in diverse contexts has shown it to be a valuable tool in improving diabetes self-management[11 12].

However, the effectiveness of existing health coaching interventions exhibits inconsistency due to variations in methodology, duration, and intervention content[11]. Recent systematic reviews of randomised controlled trials employing health coaching have reported mixed results, with some studies suggesting its effectiveness while others claim it to be ineffective[11 12]. There is no agreed-upon health coaching intervention model, which creates uncertainty regarding the most effective strategies to adopt, particularly in the SA context. The inconsistent findings across studies may be due to the lack of agreement on the active ingredients and content of successful health coaching interventions[13]. Additionally, cultural differences between SA and Western countries highlight the need for tailored interventions to ensure that proposed health coaching models are appropriate and relevant[14].

This feasibility study aims to assess the usability and acceptability of a health coaching intervention for T2DM self-management in SA, with the ultimate goal of reducing HbA1c levels and informing a future large-scale randomised controlled trial.

Materials and Methods

Design

This study adhered to the CONSORT guidelines[15], using a double-blind, randomised two-arm feasibility trial to evaluate a 3-month intervention This study adopted a mixed-methods approach enabling both quantitative and qualitative data collection. An RCT design to both evaluate the feasibility of RCT methods for recruitment, deliver and data collection as well as a valid comparison of health coaching's effectiveness between the groups[16]. The Behaviour, Capability, Opportunity, and Motivation (COM-B) model was used as guidance in adapting the intervention for a SA context. The BCTTv1 taxonomy was used to code the active ingredients of health coaching intervention and mapped it to the BCW framework[17]. To facilitate this process and link the intervention to the COM-B model, Theoretical Domains Framework (TDF) functions and BCTs, behavioural analysis was conducted to address the pre-identified barriers from the literature review (see Supplemental Table 1). As a result, 36 relevant BCTs were selected and mapped to address the behaviours barriers (see Supplemental Table 2). The intervention's target population was those with poorly managed T2DM. This enabled the interventionist to define the problem of those persons in behavioural terms, as well as the intervention's target behaviours as advised by BCW advice[18].

Intervention development

There have been various identified target behaviours that would result in the adoption of a healthy lifestyle and diet as recommended by UK lifestyle guidelines[19]. As of now, there are no evidence-based guidelines in SA for T2DM to adopt a healthy lifestyle and diet. Therefore, we have referred to the UK's guidelines on lifestyle as they share similarities in their healthcare systems [19 20]. These guidelines have been used to determine the most suitable behaviour targets

For peer review only - http://bmjopen.bmj.com/site/about/guidelines.xhtml

for the Saudi context. We consulted healthcare professionals in the intervention setting, including a dietician, to establish these targets. Through collaborative discussions, we assessed and prioritised behaviours based on their potential impact, the feasibility of measurement, and achievability. As a result of this process, we have proposed four behaviour targets [17].

The four general behaviours targets of the intervention were[19]:

- Decrease carbohydrate intake for each meal
- Use unsaturated fats as possible (avoid saturated fats)
- Do exercise for 30 min five days on a weekly basis
- Monitor waist circumference, and maintain it below (80 cm for women and 94 cm for men)

The health coaching intervention used these targets as the benchmarks to track the progress and changes that the study population would make over three months.

We utilised diverse instruments, including questionnaires, interviews, focus groups, and clinical measures, to assess the intervention's feasibility, acceptability, and preliminary impact at two points: baseline and three months post-intervention. Ethical clearance for the study was granted by the University of Sheffield and the KFMC's Institutional Review Board (IRB Log Number: 21-062E). The protocol of this study has been published elsewhere[21].

Participant recruitment

Inclusion criteria were: aged over 18, Haemoglobin A1c level of 7.0% or higher, able to read and understand Arabic, have access to a personal mobile phone, be willing to complete the intervention period, be willing to stay in Riyadh, and be able to read, understand, and sign the informed consent form.

The recruitment process involved advertising the intervention through multiple channels, including posters, brochures, social media, and healthcare provider referrals. Interested and eligible individuals met the research team for a brief study overview and questionnaire completion, providing demographic data and baseline laboratory reports.

Participants were randomly assigned to one of two groups in a 1:1 ratio by an independent individual using a computer-generated random numbers system, ensuring equal allocation chances. Research team members and participants were blinded to the group assignment until after baseline assessment completion.

Intervention delivery

The health coaching intervention was completed in 12 weeks. A detailed intervention curriculum was used in delivering the intervention activities toward achieving the four target behaviours (see Supplemental Table 3).

Three trained and qualified health coaches, AH, AM, and MG, delivered intervention sessions. They received training in biweekly F2F meetings over six weeks on the intervention curriculum. A workbook was provided as a guide to help them deliver coaching sessions as planned. A

For peer review only - http://bmjopen.bmj.com/site/about/guidelines.xhtml

detailed explanation has been published elsewhere[21]. Each coach was asked to document the BCTs they use in each session. Different BCTs were reported in each session. Each participant was assigned to one of three coaches at the baseline. Each participant received six sessions in total, which were delivered biweekly via video meetings and telephone coaching over three months. Due to the COVID-19 pandemic, the first and last meetings were conducted via video call instead of face-to-face as planned, while the rest were via telephone. Participants were encouraged to contact their coaches via WhatsApp between sessions if they had any related concerns. Eighty-four sessions were undertaken for all participants in the intervention group.

Usual Care (Control Group)

The participants in the control group were provided with the standard care, which involved receiving written materials and brochures on diabetes to increase awareness and promote the advantages of making changes to their health behaviours. Typically, individuals with T2DM have regular appointments with endocrine specialists to monitor their diabetes management. During these visits, the primary objective is to assess if any adjustments are necessary regarding the patient's medication, such as replacement, dosage increase, or maintaining the current prescription.

Data Collection

Baseline data collection included: demographic information, HbA1c, blood pressure, BMI, weight, and waist circumference measurements.

Subsequently, we asked participants to complete two electronic questionnaires: the Diabetes Self-Care Activities (SDSCA) scale and the Self-efficacy Scale for Diabetes. The post-intervention data collection replicated the initial session, with repeat measurements of HbA1c, blood pressure, BMI, weight, and waist circumference. Participants were also required to fill out the two initial questionnaires again, along with a Satisfaction Questionnaire designed specifically for the intervention group.

Reach and retention

As per the study protocol[21], this study aimed to recruit a minimum of 9% (n = 30) of the required sample size for conducting a complete trial[22]. Each group was expected to have a minimum of 12 participants to ensure meaningful data interpretation for informing a future definitive randomized controlled trial[23]. The retention rate was evaluated based on the predefined progression criteria described elsewhere[21], with successful retention defined as achieving an 83% retention rate at the intervention endpoint.

Fidelity assessment

All coaching sessions audio recorded and transcribed. An Arabic native speaker reviewed the entire transcription for accuracy. To ensure consistency and validity, a 10% sample of the transcription (approximately 50 minutes) was translated from Arabic to English, back-translated, and then examined by a peer reviewer. The data was stored twice in Microsoft Excel throughout the trial, including coaching sessions, time spent in each session, semi-structured

For peer review only

interviews, focus groups, and questionnaires. Adherence to the intervention was defined by the number of participants who completed 5 out of six coaching sessions ≥ 84%.

Feasibility

Evaluation of feasibility included: participant eligibility and recruitment, retention, data collection adherence, and adherence to the intervention. We recorder participants' views about their engagement, capturing their reasons for interest, active participation, and lack of interest. We tracked and logged the number and percentage of those who were eligible and interested, those who completed the intervention, and those who withdrew to provide insight into the study's recruitment efficiency and participant retention

Acceptability

Acceptability of the intervention and its implementation was assessed using qualitative and quantitative data. The aim was to detect any potential challenges within the methodology used to deliver both the trial and the intervention. We conducted post-intervention semi-structured qualitative interviews and focus groups. The intervention's acceptability was primarily gauged through a Likert-scale Satisfaction Questionnaire (14 items) applied to the intervention group (n=14). This questionnaire, originally devised by the Dan Abrahams Healthy Living Centre and subsequently employed by DeJesus et al. (2018), incorporates a variety of question styles (open & closed). Using a Likert scale, it offered a response range from 0 (not at all) to 10 (very much) to capture participant feedback. Given that this scale had not been used in Arabic studies before, we translated it into Arabic and pretested it on a smaller subset before implementing it for the full study sample.

Data analysis

We conducted both quantitative and qualitative analyses. For the quantitative part, descriptive analysis was undertaken including screening and recruitment rates, retention rates, adherence to coaching sessions, and recruitment duration. We evaluated the use of BCTs and interactions between participants and coaches during these sessions. A Satisfaction Questionnaire captured participants' experiences, while changes in diabetes self-management and patient self-efficacy were assessed at the beginning and end of the study. The data were entered into Excel and subsequently transferred to SPSS and STATA for advanced analysis, which included a linear regression to estimate the mean differences in outcomes.

Qualitative data were primarily sourced from audio-recorded interviews, focus groups, and coaching sessions. We employed a reflexive thematic analysis process, adhering to six systematic phases: familiarising ourselves with the data, coding, generating themes, revising themes, defining, and finally reporting[24].

In addition, we integrated the qualitative and quantitative data for comprehensive understanding[25]. Using a joint display table (see Supplemental Table 4)[26], we compared and combined these data types. The display helped us identify areas of agreement (convergence), disagreement (divergence), and enhancement (expansion) between the two data types [27]. In the integrated data, we denoted convergence with "=", complementarity with "+", and inconsistencies with "≠".

For peer review only

Although this feasibility study may not conclusively determine intervention effects, it provides valuable insights for estimating the sample size for a larger trial based on the mean difference and standard deviation of HbA1c.

Results:

Participant characteristics

The control group (mean age=53.40, SD=8.47) was slightly older than the intervention group (mean age=52, SD=8.32). The majority were married, with varying monthly incomes. About a quarter had completed high school, and an equal proportion had primary education or were literate without formal education. Approximately 31% had lived with T2DM for over ten years, with 24.1% of the intervention group having T2DM for 1-3 years. The majority (89.7%) were on diabetes medications. Table 1 summarises the demographic characteristics of both groups.

Table 1: Summary of the participants' demographic characteristics

For peer review only

		Intervention group		Control group	
		Total N (%)	Median (IQR)	Total N (%)	Median (IQR)
Gender	Male	6 (42.9%)		7 (46.7%)	
	Female	8 (57.1%)		8 (53.3%)	
Age	Year	14	54.5 (44.5 to 59.25)	15	54 (50 to 60)
Marital status	Married	14 (100.0%)		14 (93.3%)	
	Single	0 (0.0%)		1 (6.7%)	
Monthly income	less than 5000	4 (28.6%)		7 (46.7%)	
	5000-10000	2 (14.3%)		0 (0.0%)	
	10000-15000	0 (0.0%)		2 (13.3%)	
	more than 15000	1 (7.1%)		1 (6.7%)	
	Prefer not to declare	7 (50.0%)		5 (33.3%)	
Education level	Illiterate	0 (0.0%)		0 (0.0%)	
	Primary school	3 (21.4%)		3 (20.0%)	
	Secondary school	2 (14.3%)		2 (13.3%)	
	High school	3 (21.4%)		4 (26.7%)	
	Bachelor's degree	2 (14.3%)		3 (20.0%)	
	Diploma	1 (7.1%)		0 (0.0%)	
	Can read and write	3 (21.4%)		3 (20.0%)	
Since when you were diagnosed with Type 2 diabetes	less than a year	2 (14.3%)		0 (0.0%)	
	1-3	6 (42.9%)		0 (0.0%)	
	3-5	1 (7.1%)		2 (13.3%)	
	5-7	0 (0.0%)		1 (6.7%)	
	7-10	1 (7.1%)		6 (40.0%)	
	more than ten years	3 (21.4%)		6 (40.0%)	
	do not know	1 (7.1%)		0 (0.0%)	
Do you use diabetes medications ?	Yes	12 (85.7%)		14 (93.3%)	
	No	2 (14.3%)		1 (6.7%)	

For peer review only - http://bmjopen.bmj.com/site/about/guidelines.xhtml

Feasibility of the intervention

Eligibility and recruitment

The recruitment process took about five weeks, starting on May 1st, 2021, to June 5th, 2021. Leaflets were used for advertising the intervention and were distributed in different places in the hospital, including in waiting areas and on hospital wall notice boards. In addition, we met physicians in-person to introduce the intervention and gave them leaflets with more details to encourage their patients to join in the study.

Of the 42 potential participants initially identified and assessed for eligibility, 38 met the study criteria, resulting in a screening rate of approximately 90%. These 42 individuals were referred by different sources. Twenty-two were referred by their doctors, while the others were recruited through various methods. Two were from leaflets, eight were from suggestions by friends or relatives, and ten were directly engaged at the diabetes clinic. Twelve were excluded, of which four were ineligible because they did not meet the intervention inclusion criteria for the following reasons: one had no access to a personal mobile phone/smartphone, one patient was diagnosed with T1DM, one had A1c below 7%, and one was unable to do pre-and post-assessments. Eight were excluded for other reasons; two did not respond, and six for different reasons, e.g., being too busy. Thirty eligible patients were recruited to take part in the intervention. All the eligible 30 participants consented to participate in the intervention (recruitment rate = 100%). All of them completed baseline assessments and questionnaires, and then randomly, 15 patients were allocated to the intervention group, while the other 15 participants were assigned to the control group. One person who was part of the intervention group withdrew from the trial prior to the first session and was thus not included in the study (see Figure 1 CONSORT diagram below for more details).

For peer review only

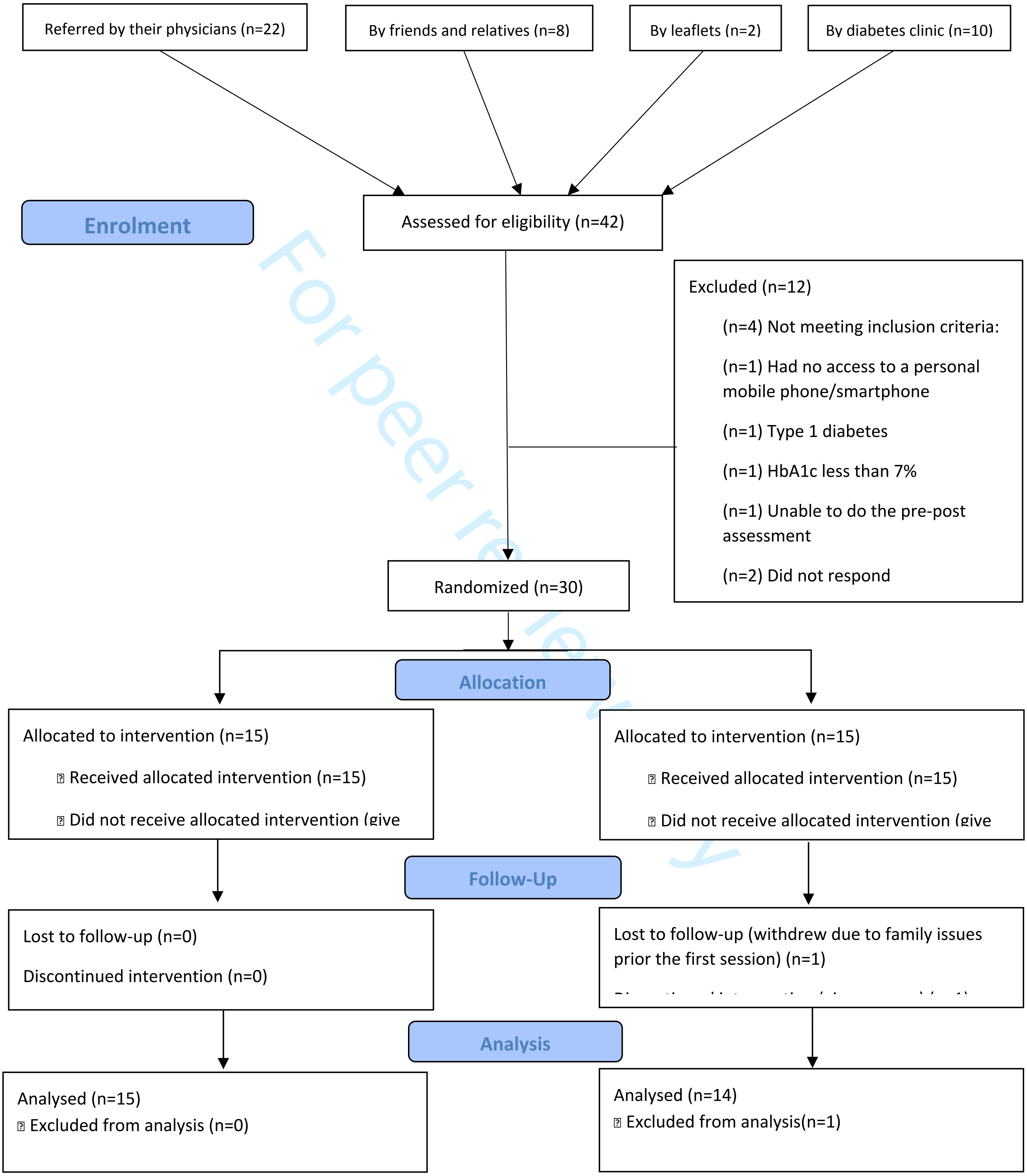

Figure 1: CONSORT Flow Diagram of the health coaching RCT[15]

For peer review only - http://bmjopen.bmj.com/site/about/guidelines.xhtml

Intervention delivery

The intervention started with the first session on June 15th, 2021, and ended on September 23rd 2021. A total of 1691 minutes of health coaching were during the intervention time. The average time spent per participant was between ~ 17.7 to 25.5 in each session. The total range time of all sessions per patient was 109-153 minutes, with an average of 120.8 (SD= 13.7).

An average of 33.8 minutes with a median of 32.5 and a range of (~ 27-44) minutes were spent for the first sessions. The last sessions took an average of 33.1 minutes across all 11 participants, with a median of 32 and a range of (~ 24-50) minutes. The rest of the four sessions took almost the same time as planned. Participants in the second session spent an average of 15.8 minutes, with a median of 15 minutes and a range of 11 (~12-22). In the third session, participants have undertaken an average of 12.8 minutes of coaching sessions with a median of 11.5 minutes and a range of 9 (~10-19) minutes. An average of 13.3 minutes of coaching sessions were taken, with a median of 13.5 minutes and a range of 7 (~10-17) minutes. In addition, the average of minutes spent in the fourth session was 12.1, with a median of 12 minutes and a range of 7 (~10-17) minutes over 12 weeks. See Supplemental Table 5 for more details about the time duration of each session.

Retention of Participants

Thirty eligible participants consented to participate in the trial and completed the baseline assessment randomly allocated either to the coaching group (n=15) or the control group (n=15). Of those, all twenty-nine who started the intervention and took the first session remained till the endpoint and completed all the intervention activities. Only one participant allocated to the coaching group had withdrawn and discontinued the study before the first session (retention rate = 100%). All twenty-nine have completed the intervention endpoint assessments. The main progress criteria and feasibility measurements are summarised in Supplemental Table 6.

Adherence to the coaching sessions

Fifteen out of thirty participants were randomly allocated to the coaching group. Before the first session, one participant withdrew (P12) due to family issues. The rest of the fourteen have completed all their coaching sessions (adherence rate 100%), which met the predetermined progression criteria of adhering to ≥ 84%. Half of the participants took their sessions at the planned time as previously scheduled. However, health coaches have rescheduled different sessions for seven participants for different reasons. The rescheduled sessions varied between 1 to 2 sessions for each one of them. Out of 84 sessions, only eleven sessions have been rescheduled (13%). The reasons for the rescheduling were home/work/appointment conflicts (n = 6 sessions), travel (n=1 session), illness (n=3 sessions), and being busy (n=1 session). All participants started their coaching sessions at the same time on June 15th, 2021, except four participants who started later on July 1st, 2021. Fifteen days of delay because their coach could not begin at that time for family issues. In addition, the same four participants had another delay between the fourth and fifth sessions due to the coach's college exams. This led them to finish their last session on September 23rd, 2021.

For peer review only - http://bmjopen.bmj.com/site/about/guidelines.xhtml

Data collection adherence:

Participants recruited for the study were invited to Al-Zulfi General Hospital on June 10th, 2021. During this visit, they were given consent forms and information sheets about the intervention, providing them with an opportunity to ask questions and discuss any concerns related to their participation. Furthermore, baseline data, including measures of HbA1c, blood pressure, BMI, weight, and waist circumference, were collected during this visit. Due to the restrictions imposed by COVID-19, all required measurements and paperwork were completed in a single visit. Participants were then asked to complete two electronic questionnaires: the Diabetes Self-Care Activities (SDSCA) scale and the Self-efficacy Scale for Diabetes. The baseline data collection was successfully completed by all 30 participants, yielding a 100% completion rate. The time taken to complete the questionnaires ranged from 7 to 12 minutes, with no participants reporting difficulties or issues in the completion process.

At the end of the intervention, participants were once again invited to the hospital to gather post-intervention data. This second data collection session was much like the first, where we again measured HbA1c, blood pressure, BMI, weight, and waist circumference. Participants were also asked to complete the initial two questionnaires, with the addition of a Likert-scale Satisfaction Questionnaire for the intervention group only. This additional questionnaire resulted in a slightly longer completion time, ranging from 9 to 15 minutes, due to the inclusion of open-ended questions and certain items requiring justifications or explanations for selected responses. Overall, all participants effectively completed the data collection process, demonstrating successful adherence to our study's data collection procedures.

Progression criteria

The feasibility measurements and predetermined progression criteria yielded positive results. The screening rate was 91%, exceeding the target of 80%, suggesting proceeding to the future definitive RCT. The recruitment rate reached 100%, meeting the criteria for progression. The retention rate atthree3 months was 96.6%, surpassing the required 83% rate. Baseline data collection adherence and intervention adherence both achieved 100%. Endpoint data collection adherence was 96.6%, with only one participant withdrawing before the first session. Overall, these results support the decision to proceed to the future definitive RCT, indicating the feasibility and acceptability of the health coaching approach for individuals with T2DM in SA.

Sample size

In the conducted feasibility study, one of the key goals was to project the necessary sample size for the upcoming main trial. Given the large effect size of -0.93 noted in this study, it could be more suitable to base the sample size estimation on the literature for a clinically significant mean difference of 0.5% and a standard deviation of 1.4. With a power of 0.8 and an alpha level of 0.05, the upcoming main study would need 125 participants per group. A larger sample size will also facilitate subgroup analyses to determine if the effect size is more substantial in specific settings or amongst certain patient groups.

Acceptability of the intervention

For peer review only

Acceptability and suitability of the intervention were assessed through a questionnaire (Likert-scale Satisfaction Questionnaire, 14 items) for the intervention group (n = 14). Using a Likert scale of 0 (not at all) to 10, most participants' responses show that the intervention affected their behaviour "quite a bit " (mean: 8.2 (2.3)). Participants replied, “very much” (mean 9.2 (1.4)) when asked how much participation in the intervention helped them establish a personal vision of wellness. When asked how the intervention boosted their confidence in taking actions toward improved well-being, the overall response was "very much" (mean 9.2 (1.4)). Their motives for making efforts toward enhanced well-being were "quite a bit" (mean 8.3 (1)). The average response to the usage of goal setting was "very much" (mean 9.2. (0.9)). Whereas their mean response to their use of problem-solving abilities was "quite a bit" (mean 8.6 (1.4)). Participants shared that the intervention greatly assisted them in overcoming obstacles and achieving increased levels of wellness, with a high mean score of 9 (1.1) reported. From the first through the eighth question, participants' responses are presented in Supplemental Table 7.

Participants were asked three "Yes" or "No" questions, and they all replied "Yes" when asked whether they expected to continue making improvements (n=14, 100%). The majority (n=13, 92.9%) responded with "Yes" the intervention met their expectations. All 14 participants who were asked whether they would recommend the intervention to others responded with a positive "yes".

Thematic Analysis findings

We analysed 1691 minutes of coaching sessions to identify the used BCTs (see Supplemental Table 8) and transcribed 497 minutes of focus groups and interviews. The accuracy of the entire transcription was confirmed by a native Arabic speaker. To further ensure validity, about 10% of the transcription (50 minutes) underwent a back-translation process checked by a professional native speaker. Throughout the intervention, the researcher maintained observational notes. Due to COVID-19 limitations, all interviews and focus groups were conducted online.

Evaluation of Participants and Health Coaches' Experiences

Various qualitative methods were used to assess participant experiences during the intervention, including interviews, focus groups, and field notes. After the intervention, three focus groups took place. Participants were given time to understand the questions before engaging in interviews and focus groups, fostering a deeper discussion. The researcher conducted all focus groups and interviews, enabling active interaction and understanding of participant perspectives.

Between June 15th and September 23rd, 2021, 14 semi-structured interviews were conducted with seven participants (4 males, three females) from the intervention group, both pre-and post-study. Participants predominantly had a high school education or less, and the median age was 59. Three had long-standing diabetes of over ten years, three had a recent diagnosis of less than three years, and one was uncertain of the duration. Six of the seven were on diabetes medication.

For peer review only - http://bmjopen.bmj.com/site/about/guidelines.xhtml

Two post-study focus groups were conducted online. The first consisted of six participants (three males and three females) from the intervention group with a median age of 52.5 years. The majority had a high school education or less, and most were on diabetes medication with a mix of recent and long-term diagnoses. The demographic characteristics of the participants can be seen in Table 2.

The second focus group included three female health coaches from KFMC in Riyadh, aimed at understanding their experiences delivering the intervention. All had health coaching backgrounds, with two holding master's degrees in health education. The session lasted 265 minutes.

For peer review only

Table 2: Participants' Demographic characteristics

		**Qualitative interview participants (n=7)**		**Focus group participants (n=6)**	
Demographic characteristics		Number	%	Number	%
Gender	Male	4	57.1	3	50
	Female	3	42.9	3	50
Age	Years	43 to 62	Median 59	34 to 60	Median 52.5
Marital status	Married	7	100	6	100
Monthly income	less than 5000	1	14.3	2	33.3
	5000-10000	1	14.3	1	16.7
	10000-15000	1	14.3	0	
	Prefer not to declare	4	57.1	3	50
Education level	Primary school	2	28.6	1	16.7
	Secondary school	2	28.6	1	16.7
	High school	2	28.6	3	50
	Bachelor's degree	1	14.3	1	16.7
Since when you were diagnosed with Type 2 diabetes	less than a year	2	28.6	1	16.7
	1-3	1	14.3	3	50
	more than ten years	3	42.9	1	16.7
	do not know	1	14.3	1	16.7

For peer review only - http://bmjopen.bmj.com/site/about/guidelines.xhtml

Do you use diabetes medications?	Yes	6	85.7	5	83.3
	No	1	14.3	1	16.7

The evaluation of the trial's acceptability, feasibility, and the experiences of participants and coaches with the health coaching intervention was explored through thematic analysis using inductive and deductive coding. Initial open coding identified emergent themes, followed by deductive coding grounded in the guidelines for qualitative research in feasibility studies and the MRC framework.

The analysis of translated transcripts yielded four key themes, two of which were predetermined. The first theme pertained to trial design, conduct, and processes, divided into four sub-themes covering different aspects of intervention implementation. The second theme focused on intervention content and delivery, consisting of sub-themes on intervention components, perceived consequences, feasibility and acceptability in practice, and reach and dose. The third theme, intervention mechanism, encompassed sub-themes around COM-B model application, use of BCTs and intervention curriculum, and intervention impact. The fourth theme, future RCT, captured participant and coach suggestions. The thematic map used in the analysis is outlined in Supplemental Figure 2.

Theme 1: Intervention design, conduct, and processes

This theme primarily focuses on the implementation and design of the intervention, along with the processes involved. Challenges were encountered regarding the practicality of recruitment during the pandemic, causing a shift from traditional in-person clinics to remote settings. This transition not only made recruitment a challenging task but also stretched the recruitment period beyond the planned timeframe.

Notably, a health coach shared their experience stating, *"...the recruitment phase, it was complicated only because all clinics become online and we could not see people with type 2 diabetes come to the hospital as usual, so the chance of meeting those people was very rare."* (FGC, C01, Female).

The lack of understanding and familiarity with health coaching in SA led to some miscommunications about the intervention. This highlighted the need for improved communication with recruiters, as one health coach observed, *"...from my communication with recruiters, some of them did not understand, so participants may get a wrong idea"* (FGC, C01, Female).

Limited hospital access due to public health guidelines complicated recruitment further. However, the suggestion to recruit from a secondary hospital made the recruitment process manageable. Participants and health coaches suggested that physicians could serve as vital intermediaries in the recruitment process due to the trust and rapport they have established with patients; as one of them stated, *".....I think one of the best and most reliable ways to reach and recruit patients is to start from their direct physicians by suggesting the program for patients and encourage them to join"* (FGC, C02, Female).

For peer review only - http://bmjopen.bmj.com/site/about/guidelines.xhtml

To reduce dropout rates, clear upfront communication regarding program expectations and commitments was proposed, with one participant suggesting to *“ask participants to sign a contract to commit and if you are hesitating or you have the intention to withdraw to tell us [referred to program provider] at the beginning to give the opportunity to someone who needs it”* (FG, P03, Female).

Both health coaches and participants were generally satisfied with the intervention's design, and they believed that it effectively accommodated the local context of Saudi society. One participant expressed, *"I’m very very very satisfied and I think the program is easy to follow and very acceptable as it gradually improves different skills which last with a patient after the program…it is completely different from other programs.."* (P13, Male).

An unexpected challenge was the additional workload on health coaches who had to juggle their regular duties with their voluntary tasks for the intervention. The COVID-19 pandemic further complicated matters by requiring greater flexibility in scheduling coaching sessions, particularly for participants with children engaged in online learning. To alleviate these issues, health coaches proposed securing formal workplace approval to dedicate full-time to the intervention. As one health coach noted, *“As you know and because of the workload, I was thinking of withdrawing from the intervention...... I think it is imperative to work a full-time job as a health coach in the intervention, not as a volunteer, and get formal permission from my work on this..”* (FGC, C01, Female).

Theme 2: Intervention content and delivery

This theme encompassed multiple sub-themes reflecting on the intervention's implementation, including its acceptability and adaptability within the Saudi context, potential unintended consequences, the feasibility of the practical application, and intervention reach and dosage.

Health coaches provided insight into ways the intervention could be modified for enhanced acceptability and suitability to the Saudi context. Emphasising the need for professionalism and structure, they proposed using formal platforms for communication, as one coach stated, *“...communication with the participant should be through a formal platform or application... to make the program more formal and organised...”* (FGC, C02, Female). They also suggested the importance of physical meetings or video calls for a more serious engagement, as expressed by another coach, *"I prefer to have a physical meeting where I can sit with the patient face-to-face…”* (FGC, C01, Female).

Coaches noted challenges stemming from a lack of designated spaces for conducting coaching sessions, highlighting the necessity of a prepared private location to give a professional image to the intervention. As one coach expressed, *“..As a coach, there should be a certain prepared private place at work to conduct all of my coaching sessions..”* (FGC, C02, Female).

Visual coaching sessions, either in-person or via video calls, were seen as preferable to phone calls. Coaches felt that seeing the participant was vital for effective communication and coaching. A health coach expressed this, saying, *“...visual interaction with participants is very very important, you know the importance of reading and understanding the body language for me as a coach..”* (FGC, C03, Female).

For peer review only - http://bmjopen.bmj.com/site/about/guidelines.xhtml

Participants found the intervention straightforward and effective in helping them make progress towards their goals. Some suggested a desire for additional sessions to further adopt and maintain new habits. One participant commented, *"I think three months was enough" (P03, Female), while another remarked, "...it enabled me to stay committed, continue, and achieve my goals"* (P09, Male).

Participants appreciated the use of health coaching techniques, such as action plans and SMART goals, which helped set achievable targets and keep them motivated. The flexibility and graduality of the program were also well-received, as one participant mentioned, *"...the most thing I liked about the program was the graduality to achieve goals .."* (P13, Male).

Coaches affirmed that the intervention content was acceptable and had observable positive impacts. They also suggested the addition of monthly sessions or an extension of the intervention to six months for better patient progress monitoring and maintenance. A coach stated, *"... I suggest the intervention last longer… I think it would be good if the intervention lasts six months with a session each month …"* (FGC, C01, Female).

Theme 3: Intervention Mechanism

This theme emphasises the practical application and effects of the COM-B health coaching model. Coaches received specialised training on the curriculum, and behavioural change techniques (BCTs), utilising several tools for efficient delivery. Notably, coaches documented the BCTs employed in each session, enabling tailored strategies to target specific behaviours. Participants appreciated this approach, stating, *"The BCTs helped me during each session to address some behaviours…"* (FGC, C01, Female).

The COM-B model was instrumental in the intervention, targeting four behaviours. Participants noted marked improvements in their knowledge and motivation pre-and post-intervention. Testimonials showed that they were more knowledgeable about their condition, diet, and physical activity. One participant said, *"I became more knowledgeable...now I know diet types and what carbohydrates mean…"* (FG, P05, Female).

The intervention also impacted participants' social and physical environment, leading to a supportive atmosphere for behavioural change. They indicated that the changing social environment in SA encouraged the adoption of a healthier lifestyle and influenced their social networks. As one participant put it, *"While I was changing some of my old habits, my social network adapted..."* (P03, Female).

The individualised nature of the intervention fostered motivation and led to a change in participants' views about their condition. This motivation was further fueled by the noticeable changes in outcomes such as HbA1c levels. Participants recognised the power of lifestyle changes and felt empowered by their improved health results, stating, *"...after the intervention, I realised modifying the lifestyle is the actual treatment..."* (P13, Male). Consequently, there were requests to extend the intervention, given the observable impacts, including reductions in A1c levels and body weight.

Participants in SA observed notable differences between the novel concept of health coaching and traditional care. The former was seen as superior, with participants expressing

For peer review only - http://bmjopen.bmj.com/site/about/guidelines.xhtml

dissatisfaction with traditional care's impersonal nature. Health coaching, in contrast, provided a more personalised approach, allowing patients to voice their concerns. As one participant expressed, *"I would choose this program without hesitation; at least I finish each session while I'm comfortable..."* (P03, Female).

Traditional care was perceived as a top-down process, with patients passively receiving pre-prepared information or plans. The health coaching intervention, however, positioned participants as active stakeholders in their own care by encouraging goal-setting and plan creation. A participant noted, *"Your program helped me use new skills such as setting plans and having short-term and long-term goals..."* (P08, Male).

Frustration and even anger marked participants' feelings about traditional care, citing time constraints, a lack of empathy, and the expense of private consultations. Health coaching was seen as a way to alleviate these issues, with participants suggesting the incorporation of health coaching clinics in hospitals to facilitate meaningful patient discussions and foster better disease self-management. A participant suggested, *"I think it is important to recruit a coach in the hospital... 90% of patients with chronic diseases need to sit with someone to have meaningful discussions..."* (P08, Male).

Theme 4: Improvement opportunities in the future health coaching RCT

As this was Saudi Arabia's initial health coaching intervention, participants and coaches suggested several enhancements for future large-scale implementation. Participants favoured increased coaching frequency, more face-to-face sessions, and prioritising highly committed patients with uncontrolled diabetes. One participant commented, *"I would rather have a longer time with you and more face-to-face sessions"* (P01, Female).

Health coaches suggested including dietitians and physicians for prompt consultations or referrals given diabetes' dietary and medical implications. Regular meetings among health coaches to exchange experiences were also advised. One coach stated, *"One of the modifications I suggest is having a nutritionist and a physician in the program..."* (FGC. C03, Female).

Incentives, whether financial or expanded access to hospital services, were suggested to foster participation. Health coaches also proposed creating a short explanatory video about the program, highlighting a real-life coaching session. This visual aid aims to streamline the introductory process; as one coach explained, *"I would suggest creating and sharing a small video explaining the idea of health coaching"* (FGC, C02, Female).

Preliminary effects of the intervention

The preliminary intervention effects consist of primary and secondary outcomes. HbA1c was the primary outcome, while the intervention's secondary outcomes included Blood pressure, Body mass index (BMI), Waist circumference, Weight, Patients' self-efficacy, and Diabetes self-management. Each one of these outcomes is discussed in more detail below.

Glycosylated haemoglobin (HbA1c)

A linear regression model was used to evaluate the efficacy of a health coaching intervention aimed at reducing participants' HbA1c levels between the two groups adjusted for baseline

For peer review only - http://bmjopen.bmj.com/site/about/guidelines.xhtml

HbA1c levels. At the study's onset, the two groups' baseline values were reasonably balanced. The overall average HbA1c level among all participants decreased from 8.9% (SD= 2.09) at baseline to 8.14% (SD= 1.79) at the end of the study. The mean difference in HbA1c levels between pre-and post-intervention was 0.8 for all participants. The estimated mean difference in post-intervention HbA1c levels between the two groups after adjusting for the baseline HbA1c level from the regression analysis model is -1.86 (95% CI: -2.71 to -1.01, P<0.001). Participants in the intervention group with elevated baseline HbA1c levels (≥7%, n=14) achieved a significant reduction in HbA1c levels of 1.84% over a period of three months, from 9.1% (SD= 2.50) at baseline to 7.2% (SD= 1.38) post-intervention. Conversely, the HbA1c levels in the control group increased from 8.7% (SD= 1.69) at baseline to 8.9% (SD= 1.81) by the end of the study (see Tables 3 and 4).

Mean Arterial Pressure (MAP)

The mean differences were computed using Mean Arterial Pressure (MAP), which represents the average arterial pressure over a single cardiac cycle that includes both systole and diastole (see Tables 3 and 4) [28]. The average MAP for all participants (n=29) was 89.1 mmHg (SD= 12.64) at baseline and 91.4 mmHg (SD= 11.1) at the end of the study. The estimated mean difference in post-intervention MAP scores between the two groups after adjusting for the baseline MAP level is -.683 (95% CI: 7.46 to 6.09, p=0.83).

Weight

The weight difference between the two groups was assessed by comparing their means using a linear regression model. A slight reduction in the average weight of all participants (n=29) was observed, decreasing from 82.3 kg (SD= 12.22) at baseline to 80.9 kg (SD= 11.56) at the endpoint (see Tables 3 and 4). Although the intervention group (n=14) had a decrease in weight of 2.79 kg, the difference was not statistically significant, with a mean difference of -2.58 (95% CI: -5.25 to .082, P= 0.057).

BMI

The average BMI for all participants (n=29) was similar at baseline (30.6, SD= 4.95) and endpoint (30.15, SD= 4.84). However, the intervention group (n=14) showed a greater change from baseline (30.39, SD= 4.57) to 29.39 (SD= 4.33) after the intervention, compared to the control group (from 30.87, SD= 5.43 to 30.86, SD= 5.31). The post-intervention mean difference between the two groups was significant, with -1.02 (95% CI: -2.01 to -.041, P=0.042). See Tables 3 and 4 for further details.

Waist Circumference

The average waist circumference for all participants (n=29) decreased from 107.9 cm ± 11.83 at baseline to 104.56 cm (SD= 11.30) at the endpoint. The intervention group (n=14) showed a larger reduction from 106.6 cm (SD= 13.60) at baseline to 102.21 cm (SD= 12.55) after the intervention. Conversely, the control group (n=15) had a slight increase in waist circumference by 0.09 cm at the endpoint (see Tables 3 and 4). The estimated mean difference in post-intervention waist circumference scores between the two groups after adjusting for the baseline waist circumference scores is -6.89 (95% CI: -10.17 to -3.61, P<0.001).

Table 3: Groups means and SDs at study baseline and endpoint.

For peer review only - http://bmjopen.bmj.com/site/about/guidelines.xhtml

	Group	N	Mean	Std. Deviation
A1c level at the study baseline	Intervention Group	14	9.129	2.50
	Control Group	15	8.733	1.69
A1c level at the study endpoint	Intervention Group	14	7.293	1.38
	Control Group	15	8.933	1.81
Mean Arterial Pressure at the study baseline	Intervention Group	14	91.76	13.56
	Control Group	15	86.73	11.66
Mean Arterial Pressure at the study endpoint	Intervention Group	14	92.57	9.11
	Control Group	15	90.33	12.91
Height (cm) at the study baseline	Intervention Group	14	166	7.24
Height (cm) at the study baseline	Control Group	15	162.5	7.59
Weight in Kg at the study baseline	Intervention Group	14	83.500	11.97
	Control Group	15	81.213	12.76
Weight in Kg at the study endpoint	Intervention Group	14	80.707	10.80
	Control Group	15	81.213	12.60
BMI at the study baseline	Intervention Group	14	30.3933	4.578
	Control Group	15	30.8720	5.43
BMI at the study endpoint	Intervention Group	14	29.3927	4.33
	Control Group	15	30.8632	5.318
Waist Circumference (cm) at the study baseline	Intervention Group	14	109.357	13.60
	Control Group	15	106.667	10.22
Waist Circumference (cm) at the study endpoint	Intervention Group	14	102.214	12.55
	Control Group	15	106.760	9.91

Table 4: Outcomes mean differences taking into account the baseline value regression

Endpoint HbA1c	Mean difference	t	P>t	[95% CI]	
Group*	-1.864463	-4.50	<0.001	-2.716065	-1.012862
Baseline HbA1c	.566714		<0.001	.3601448	.7732833
Endpoint MAP	Mean difference	t	P>t	[95% CI]	
Group*	-.6833264	-0.21	0.837	-7.463123	6.09647
Baseline MAP	.5809645	4.38	<0.001	.3083653 8	.853563
Endpoint Weight	Mean difference	t	P>t	[95% CI]	
Group	-2.584974	-1.99	0.057	-5.252461	.0825133
Baseline Weight T1	.9090889	16.84	<0.001	.7981218	1.020056
Endpoint BMI	Mean difference	t	P>t	[95% CI]	
Group	-1.024214	-2.14	0.042	-2.006754	-.0416741
Baseline BMI T1	.9322785	19.02	<0.001	.8315092	1.033048
Endpoint WC	Mean difference	t	P>t	[95% CI]	
Group	-6.895251	-4.32	<0.001	-10.17924	-3.611259
Baseline WC	.8732792	12.72	<0.001	.7322131	1.014345

*(Group = Intervention mean – Control mean)

Patients' self-efficacy questionnaire

The self-efficacy of patients was assessed using an 8-item scale[29], where scores ranged from 1 (least confident) to 10 (most confident). Higher mean scores indicated greater self-efficacy. Both intervention and control groups displayed an increased average response from baseline to the study endpoint, with higher improvements observed in the intervention group (see Supplemental Table 9). The groups exhibited a statistically significant difference (P = .006, 59 % CI .713 to 3.839) in confidence related to controlling their condition and maintaining exercise. Similarly, a significant difference (P = .006, 59 % CI 0.82 to 4.35) was noted in confidence regarding appropriate food choices when hungry. This suggests the intervention group saw greater improvements in self-efficacy compared to the control group.

For peer review only - http://bmjopen.bmj.com/site/about/guidelines.xhtml

Diabetes Self-Care Activity

The Diabetes Self-Care Activity scale[30], scoring 0 (no adherence) to 7 (complete adherence), measured patients' adherence to specific activities over the past week. Average scores showed overall improvements in self-care for both the intervention and control groups across all activities, with the intervention group often meeting or exceeding American Diabetes Association recommendations (see Supplemental Table 10)[31]. For instance, adherence to a healthy eating plan in the intervention group increased from an average of 1.64 to 5.00 days per week. Physical exercise also increased in both groups, with a larger improvement in the intervention group (3.57 to 5.64 days). Similarly, there was improved management of carbohydrate intake in the intervention group, from 1.71 to 4.00 days per week.

Integration Results:

The key findings from both quantitative and qualitative data, aligned with research objectives, were assembled in a joint display table (see Supplemental Table 4). The table was divided into five columns, with the first signifying the study's critical concepts and the second presenting the study's progression criteria to further elucidate the mixed-methods findings. The third and fourth columns displayed the quantitative and qualitative results relevant to the key concept, respectively, and the final column encapsulated the derived meta-inferences from the integrated results.

The integration of quantitative and qualitative data insights offered enhanced comprehension of findings collated and analysed separately. This approach bolstered the credibility and transparency of the overall results through data triangulation, allowing for comparison and validation of findings that a single method wouldn't permit. The integrated results substantiated the pertinence of mixed approaches, essential to the study's feasibility and acceptability exploration in the Saudi context for a large-scale intervention with T2DM patients.

The study reported excellent screening, recruitment, and retention rates at 90.5%, 100%, and 100%, respectively, exceeding the progression criteria. These results were corroborated by the integrated analysis, suggesting a promising potential for the study within the Saudi setting. This was further supported by participants' willingness to engage in the intervention, extend invitations to family members, and their adherence to complete the intervention, despite some rescheduled sessions.

Notably, improvements were observed across five out of seven preliminary outcomes, which were validated by qualitative data. Participants and coaches reported positive experiences with the intervention's usability, design, and content. Moreover, qualitative results echoed the high satisfaction levels among participants identified in the quantitative Likert-scale Satisfaction Questionnaire. Despite the challenge posed by session rescheduling flexibility for coaches, it facilitated participant adherence, as supported by quantitative data: all participants completed their sessions with only eleven sessions rescheduled.

Discussion:

The findings of this feasibility study support the transition to a larger RCT for a health coaching intervention targeting patients with T2DM in SA. The study demonstrated a high screening rate

For peer review only - http://bmjopen.bmj.com/site/about/guidelines.xhtml

of 90.5%, surpassing the criteria set for progression and outperforming similar studies[32-34]. Despite the challenges posed by COVID-19 restrictions, the feasibility study achieved a remarkable recruitment rate of 100%, surpassing the recruitment rates reported in previous studies. For instance, Basak C & Schou (2013) recruited 90.3% of their sample[34], Kim et al. (2015) achieved a recruitment rate of 90.7% [32], and Cho et al. (2011) reported a recruitment rate of 69.6% [33]. This highlights the promising recruitment success of the feasibility study.

Comparatively, the retention rate in this study exceeded that of a similar study (83.6%)[32], as well as the rates reported by Basak C & Schou (2013) (87%) [34] and Cho et al. (2011) (90%) [33]. Furthermore, the study achieved an unexpected retention rate of 100%, even in the face of unprecedented circumstances caused by the pandemic and the novelty of the intervention for an older cohort. The high adherence rate observed in the study was attributed to the flexibility of rescheduling coaching sessions. However, this flexibility added to the workload of the coaches, which should be considered in future RCTs.

The integrated findings from both quantitative and qualitative methods confirmed the feasibility of conducting a larger RCT, even under COVID-19 restrictions. With comprehensive data collection, appropriate outcome assessments, and high participant adherence, this trial holds promise for successful execution and data collection in a future large-scale RCT. To enhance reach and diversity, future efforts should consider employing various recruitment methods, such as social media, physicians, and relatives. Additionally, creating a brief explanatory video of the intervention could be useful in saving time and ensuring a consistent understanding of the intervention's approach.

The feasibility study utilised the COM-B model and the BCTs taxonomy, which proved to be effective in guiding intervention content and addressing participant barriers. The most frequently used BCTs aligned with previous literature. The study adapted to telephone-based sessions due to COVID-19 restrictions, but a mixed approach with face-to-face interactions was preferred by both participants and coaches. A longer intervention duration of up to six months with more face-to-face sessions was recommended to ensure sustained behaviour changes.

The intervention yielded positive outcomes, with significant improvements observed in key outcomes, including the primary outcome of HbA1c, favouring the health coaching group over the control group. This study supports the previous review's findings [35], that using HbA1c as the main intervention outcome can improve effectiveness. Participants actively developed and utilised key skills to manage their condition, indicating the efficacy of the coaching model. The patient-centred approach of the intervention was well-received by participants, contrasting with their usual brief healthcare visits.

Although feasibility studies are not formally powered to detect conclusive effects on interventions [36], this study demonstrated the high acceptability and usability of the intervention in the Saudi context. However, concerns were raised by health coaches regarding their volunteer roles and the challenges of finding suitable locations for coaching sessions. These issues need to be addressed in planning for the larger RCT. The ongoing societal changes in

For peer review only - http://bmjopen.bmj.com/site/about/guidelines.xhtml

SA, along with participants' satisfaction with the intervention, suggest potential acceptance for a large-scale RCT.

Participants expressed a high level of satisfaction with the intervention, even surpassing initial expectations considering the challenging circumstances of the COVID-19 pandemic and the novelty of the approach in Saudi Arabia's traditional healthcare system. The intervention was recommended by all participants, and the potential role of health coaches within the healthcare system was emphasised.

In planning for the future definitive RCT, feedback and perspectives from both participants and coaches should be considered to enhance applicability and implementation success. Recommendations included extending the intervention duration to six months with more face-to-face sessions, involving experts from other fields for quick consultations, having regular meetings among coaches, offering incentives for participation and recruitment, and creating a short video to clarify the concept of health coaching during the recruitment process.

This research marks the introduction of a patient-centred health coaching approach in SA, with feedback from participants demonstrating the approach's promise in managing T2DM and other chronic diseases. The intervention's personalised nature was favourably received, signalling the potential for analogous strategies in managing chronic conditions. The detailed curriculum used in the study addresses the frequent pitfalls of imprecision and insufficiency in health coaching programs, thus providing a roadmap for future interventions. The successful application of the COM-B model and BCTs accentuates the importance of providing comprehensive training and administrative support to health coaches. In alignment with the Ministry of Health's shift towards behaviour-based strategies, these results endorse the broader adoption of health coaching, stressing the importance of client-centred and customised programs. Consequently, these findings significantly affect current T2DM practices and broader chronic disease management in SA.

Overall, this feasibility study demonstrates the potential effectiveness of health coaching for managing T2DM. The intervention yielded positive outcomes, was well-received by participants, and showed high levels of satisfaction. These findings provide valuable insights for guiding a future definitive RCT and contribute to the growing evidence on the acceptability and usability of health coaching interventions.

Strengths and limitations of this study

This study employed the BCW framework, which proved beneficial in understanding the problem of T2DM in the context of SA through a comprehensive analysis of previous literature. The COM-B model facilitated a narrative review to identify barriers and facilitators to controlling T2DM, addressing a knowledge gap. This study was the first to utilise the BCW, COM-B model, and BCTs taxonomy, analysing every health coaching session and coding each applied BCT, resulting in a clear intervention description.

The inclusion of progression criteria, as recommended by the CONSORT 2010 statement, aided in decision-making for the larger trial[37]. The study findings, including effect size and retention rate, informed the future main trial's sample size estimation. The study also utilised

For peer review only - http://bmjopen.bmj.com/site/about/guidelines.xhtml

both the Summary of Diabetes Self-Care Activity questionnaire and the Self-efficacy Scale for Diabetes scale, providing a comprehensive assessment of health coaching's impact. A mixed methods approach was employed to gather diverse perspectives, enhance validity and reliability, and produce more comprehensive findings.

However, the study had limitations regarding generalizability due to the specific setting and population. The small sample size and lack of statistical power limited the detection of significant differences. The intervention sample's recruitment from a public hospital may not be applicable to primary care settings, and the age cohort of older adolescents may limit applicability to younger individuals.

Conclusion

This mixed methods feasibility RCT demonstrated the feasibility and acceptability of implementing a health coaching approach for individuals with T2DM in SA. The integration of qualitative and quantitative findings yielded positive outcomes, providing strong support for conducting a larger RCT to further investigate the effectiveness of health coaching in enhancing diabetes self-management among individuals with T2DM in SA. Importantly, this study offers valuable insights for future trials, particularly in terms of incorporating the COM-B model and BCTs taxonomy into the health coaching approach. The positive feedback received for implementing a patient-centred approach indicates that it could help manage other chronic diseases. This aligns with the Ministry of Health's behaviour-focused strategies, providing more evidence to support the expansion of health coaching. As a result, these insights have significant implications for the current management practices of T2DM and beyond. Future research should also explore the social and environmental impacts of Vision 2030, the national vision of SA, on diabetes self-management. By considering contextual factors that may influence the implementation and outcomes of health coaching interventions for diabetes management in SA, further investigations can deepen our understanding and inform strategies to improve diabetes care and other chronic diseases in the country.

For peer review only

For peer review only - http://bmjopen.bmj.com/site/about/guidelines.xhtml

Author Contributions: A.N.A. was responsible for the study's conception and design, data acquisition, analysis, and interpretation, as well as the drafting the manuscript. E.G. contributed to the critical revision of the manuscript based on data analysis. AH, AM, MG, A.A were responsible for the implementation of the intervention. All authors have thoroughly reviewed and approved the final version of the manuscript for publication

Funding: This study received no external funding.

Competing interests: The authors declare no conflicts of interest.

Patient consent for publication: Not required.

Ethics approval: Ethical approval was obtained by the University of Sheffield and the Institutional Review Board (IRB) committee form ethical approval at (KFMC) (IRB Log Number: 21-062E).

Data Availability Statement: All data generated or analysed during this study are available upon reasonable request from the corresponding author.

Acknowledgement: We express our sincere gratitude to the study participants for their valuable time and willingness to participate. We are also grateful to KFMC for granting permission to conduct the intervention. Furthermore, we extend our thanks to the University of Sheffield Institutional Open Access Fund for their support towards this research. Finally, we would like to acknowledge that this paper will be published under a Creative Commons Attribution (CC BY) license.

For peer review only - http://bmjopen.bmj.com/site/about/guidelines.xhtml

References

1. Alqurashi KA, Aljabri KS, Bokhari SA. Prevalence of diabetes mellitus in a Saudi community. *Annals of Saudi Medicine* 2011;31(1):19-23. doi: 10.4103/0256-4947.75773
2. Association AD. Diagnosis and classification of diabetes mellitus: American Diabetes Association, 2010:S62-S62.
3. Hex N, Bartlett C, Wright D, et al. Article: Health Economics Estimating the current and future costs of Type 1 and Type 2 diabetes in the UK, including direct health costs and indirect societal and productivity costs. doi: 10.1111/j.1464-5491.2012.03698.x
4. Alqahtani M, Almutairi FE, Albasseet AO, et al. Knowledge, Attitude, and Practice of Diabetes Mellitus Among the Saudi Population in Riyadh, Saudi Arabia: A Quantitative Study. *Cureus* 2020 doi: 10.7759/cureus.6601
5. Organization WH. Noncommunicable diseases 2022 [Available from: https://www.who.int/news-room/fact-sheets/detail/noncommunicable-diseases accessed 17/07/2022 2022.
6. Alneami YM, Coleman CL. Risk Factors for and Barriers to Control Type-2 Diabetes among Saudi Population. *Global Journal of Health Science* 2016;8(9) doi: 10.5539/gjhs.v8n9p10
7. Alabdulbaqi D. A Cultural Adaptation of a Diabetes Self-Management Programme for Patients with Type 2 Diabetes Attending a Primary Health Care Centre in Saudi Arabia, 2019.
8. Al Slamah T, Nicholl BI, Alslail FY, et al. Cultural adaptation of self-management of type 2 diabetes in Saudi Arabia (qualitative study). *PLOS ONE* 2020;15(7):e0232904-e04. doi: 10.1371/journal.pone.0232904
9. Wolever RQ, Dreusicke M, Fikkan J, et al. Integrative Health Coaching for Patients With Type 2 Diabetes. *The Diabetes Educator* 2010;36(4) doi: 10.1177/0145721710371523
10. Wong-Rieger D, Rieger FP. Health coaching in diabetes: empowering patients to self-manage: Elsevier, 2013:41-44.
11. Hill B, Richardson B, Skouteris H. Do we know how to design effective health coaching interventions: A systematic review of the state of the literature. *American Journal of Health Promotion* 2015;29(5):e158-e68. doi: 10.4278/ajhp.130510-LIT-238
12. Pirbaglou M, Katz J, Motamed M, et al. Personal Health Coaching as a Type 2 Diabetes Mellitus Self-Management Strategy: A Systematic Review and Meta-Analysis of Randomized Controlled Trials: SAGE Publications Inc., 2018:1613-26.
13. Hill B, Richardson B, Skouteris H. Do we know how to design effective health coaching interventions: a systematic review of the state of the literature. *American journal of health promotion : AJHP* 2015;29(5):e158-68. doi: 10.4278/ajhp.130510-LIT-238
14. Alanazi FK, Alotaibi JS, Paliadelis P, et al. Knowledge and awareness of diabetes mellitus and its risk factors in saudi arabia: Saudi Arabian Armed Forces Hospital, 2018:981-89.

For peer review only - http://bmjopen.bmj.com/site/about/guidelines.xhtml

15. Eldridge SM, Chan CL, Campbell MJ, et al. CONSORT 2010 statement: Extension to randomised pilot and feasibility trials. *The BMJ* 2016;355 doi: 10.1136/bmj.i5239
16. Hariton E, Locascio JJ. Randomised controlled trials – the gold standard for effectiveness research: Study design: randomised controlled trials: Blackwell Publishing Ltd, 2018:1716-16.
17. Michie S, Ashford S, Sniehotta FF, et al. A refined taxonomy of behaviour change techniques to help people change their physical activity and healthy eating behaviours: The CALO-RE taxonomy. *Psychology and Health* 2011;26(11):1479-98. doi: 10.1080/08870446.2010.540664
18. Michie S, van Stralen MM, West R. The behaviour change wheel: A new method for characterising and designing behaviour change interventions. *Implementation Science* 2011;6(1):42-42. doi: 10.1186/1748-5908-6-42
19. Dyson PA, Twenefour D, Breen C, et al. Diabetes UK evidence-based nutrition guidelines for the prevention and management of diabetes. *Diabetic Medicine* 2018;35(5):541-47. doi: 10.1111/dme.13603
20. Al Slamah T, Nicholl BI, Alslail FY, et al. Self-management of type 2 diabetes in gulf cooperation council countries: A systematic review. *PLOS ONE* 2017;12(12):e0189160-e60. doi: 10.1371/journal.pone.0189160
21. Almulhim AN, Goyder E, Caton SJ. Assessing the Feasibility and Acceptability of Health Coaching as a New Diabetes Management Approach for the People with Type 2 Diabetes in Saudi Arabia: A Protocol for a Mixed Methods Feasibility Study. *International Journal of Environmental Research and Public Health* 2022;19(22):15089.
22. Cocks K, Torgerson DJ. Sample size calculations for pilot randomized trials: A confidence interval approach. *Journal of Clinical Epidemiology* 2013;66(2):197-201. doi: 10.1016/j.jclinepi.2012.09.002
23. Julious SA. Sample size of 12 per group rule of thumb for a pilot study. *Pharmaceutical Statistics* 2005;4(4):287-91. doi: 10.1002/pst.185
24. Braun V, Clarke V. Using thematic analysis in psychology. *Qualitative Research in Psychology* 2006;3(2):77-101. doi: 10.1191/1478088706qp063oa
25. McCrudden MT, Marchand G, Schutz PA. Joint displays for mixed methods research in psychology. *Methods in Psychology* 2021;5:100067.
26. Morgan DL. Commentary—After triangulation, what next?: Sage Publications Sage CA: Los Angeles, CA, 2019:6-11.
27. Creswell JW, Clark VLP. Designing and conducting mixed methods research: Sage publications 2017.
28. DeMers D, Wachs D. Physiology, Mean Arterial Pressure (2021). *Disponıvel em* [*http://www*](http://www) *ncbi nlm nih gov/pubmed/30855814 Ultimo acesso* 2020;14(03)
29. BOOK QC. Stanford Patient Education Research Center. *American Journal of Public Health* 1990;80:446-52.
30. Toobert DJ, Hampson SE, Glasgow RE. The summary of diabetes self-care activities measure: Results from 7 studies and a revised scale. *Diabetes Care* 2000;23(7):943-50. doi: 10.2337/diacare.23.7.943
31. Association AD. Understanding A1C Diagnosis: ADA; 2022 [Available from: https://diabetes.org/diabetes/a1c/diagnosis accessed 15 Feb 2023 2023.
32. Kim MT, Kim KB, Huh B, et al. The Effect of a Community-Based Self-Help Intervention. *American Journal of Preventive Medicine* 2015;49(5) doi: 10.1016/j.amepre.2015.04.033
33. Cho J-H, Kwon H-S, Kim H-S, et al. Effects on diabetes management of a health-care provider mediated, remote coaching system via a PDA-type glucometer and the Internet. *Journal of Telemedicine and Telecare* 2011;17(7) doi: 10.1258/jtt.2011.100913
34. Basak Cinar A, Schou L. Health promotion for patients with diabetes: health coaching or formal health education? *International Dental Journal* 2014;64(1) doi: 10.1111/idj.12058

For peer review only

35. Almulhim AN, Hartley H, Norman P, et al. Behavioural Change Techniques in Health Coaching-Based Interventions for Type 2 Diabetes: A Systematic Review and Meta-Analysis. *BMC Public Health* 2023;23(1):1-21.
36. Lee EC, Whitehead AL, Jacques RM, et al. The statistical interpretation of pilot trials: should significance thresholds be reconsidered? *BMC medical research methodology* 2014;14(1):1-8.
37. Schulz KF, Altman DG, Moher D. CONSORT 2010 Statement: Updated guidelines for reporting parallel group randomised trials. *BMJ (Online)* 2010;340(7748):698-702. doi: 10.1136/bmj.c332

For peer review only

For peer review only - http://bmjopen.bmj.com/site/about/guidelines.xhtml

Supplemental Table 1: link the pre-identified barriers to the COM-B model, TDF framework, intervention functions and BCTs

COM-B		TDF	Barrier	Intervention Function	BCTs
Capability	Psychological	Knowledge	Poor T2DM knowledge affect self-control<br>Poor nutrition knowledge (what the association between diabetes and diet, type of healthy food)<br>Poor knowledge about PA (how to engage in PA, how much time spend on PA, PA intensity, PA types, underestimate the role of PA	Education	5.1Information about health consequences
	Physical	Physical skills	Lack of the energy to do PA<br>Lack or limited skills of physical skills to do exercise	Enablement<br>Training	**Enablement:**<br>6.1Demonstration of the behaviour<br>1.5 Review behaviour goal (s)<br>1.7 Review outcome goal (s)<br>2.3 Self-monitoring of behaviour<br>12.1 Restructuring the physical environment<br>4.1 Instruction on how to perform a behaviour'<br>3.1 Social support (unspecified)<br>**Training:**<br>8.1 Behavioural practice/rehearsal<br>8.3 Habit Formation<br>8.4 Habit reversal<br>8.7 Graded tasks<br>15.4 Self-talk<br>10.9 Self-reward<br>2.2 Feedback on behaviour

					2.3 Self-monitoring of behaviour
Opportunity	Social	Social influences	Social norms & habits: overuse of high calories food intake; carbohydrates and fats, eating together (collectives), and social courtesy to eat unhealthy diet	Enablement	**Enablement**:<br>1.3 Goal setting (outcome)<br>1.1 Goal setting (Behaviour)<br>3.1 Social support (unspecified)<br>1.4 Action planning<br>1.2 Problem-solving<br>2.3 Self-monitoring of behaviour<br>8.7 Graded tasks<br>5.5 Anticipated regret<br>12.1 Restructuring the physical environment<br>12.2 Restructuring the social environment
	Physical	Environmental context and resources	Lack of time<br>Lack of resources (environmental, appropriate climate & financial ability)<br>Lack of access to do activity<br>Overuse of cars for transportations	Restriction<br>Environmental restructuring<br>Enablement<br>Modelling	**Enablement:**<br>1.3 Goal setting (outcome)<br>1.1 Goal setting (Behaviour)<br>3.1 Social support (unspecified)<br>1.4 Action planning<br>1.2 Problem-solving<br>2.3 Self-monitoring of behaviour<br>9.2 Pros and cons<br>9.3 Comparative imagining of future outcomes<br>8.7 Graded tasks<br>1.9 Commitment

					13.2 Framing/reframing<br>5.5 Anticipated regret<br>12.5 Adding objects to the environment<br>**Modelling:**<br>6.1 Demonstration of the behaviour<br>**Environmental restructuring:**<br>12.1 Restructuring the physical environment<br>7.1 Prompts/Cues<br>**Restriction:**<br>Use rules to reduce opportunity to engage in unwanted behaviour
Motivation	Reflective	Beliefs about own capability	Lack of willpower and self-confidence to do PA and maintain healthy diet	Persuasion<br>Education<br>Enablement	**Persuasion:**<br>15.1 Verbal persuasion about capability<br>15.2 Mental rehearsal of successful performance<br>9.1 Credible source<br>2.2 Feedback on behaviour<br>13.2 Framing/reframing<br>15.3 Focus on past success<br>**Education:**<br>5.1 Information about health consequences<br>5.3 Information about social and environmental consequences<br>**Enablement:**<br>1.9 Commitment<br>5.5 Anticipated regret

		Beliefs consequences	Fear from consequences of PA (fear of injury and disease future complications)	Education	5.1 Information about health consequences
		Social role and identity	Struggle to change social identity associated with culture diet<br>Struggle to accept the fact of living with diabetes	Education<br>Persuasion	**Education**:<br>5.1 Information about health consequences<br>**Persuasion:**<br>13.5 Identity associated with changed behaviour

For peer review only

Supplemental Table 2:Summarise and map how the intervention links each behaviour target to specific intervention functions and BCTs

For peer review only

COM-B	TDF coding included in	What needs to occur to bring about change?

For peer review only

		**this research**	Decrease carbohydrate intake in each meal	Use unsaturated fats as possible (avoid saturated fats)	Do exercise for 30 min, five days on a weekly basis	Monitor waist circumference
Capability	Psychological	Knowledge	Knowledge:<br>Understand food types (especially carbohydrate types, e.g., rice and dates)<br>Understand carbohydrate quantity targets<br>Understand alternative food (contain many fibres) to replace carbohydrate to avoid hunger	Knowledge:<br>Understand food types in terms of fats included<br>Understand unsaturated fats sources<br>Start learning how to cook to take control of meals' components	Knowledge:<br>Understand the role of PA and its impacts in simple language<br>Understand how to do exercise (indoor and outdoor)<br>Understand what the most suitable and enjoyable exercise	Knowledge:<br>Understand the associations between waist circumference and T2DM<br>Understand the recommended target for waist size<br>Understand how to maintain a good size
	Physical	Physical skills	How to measure carbohydrate quantity	NA	Have the energy and the required skills to do PA	Taking the measurement frequently for comparison
Opportunity	Social	Social influences	Having social support, e.g., family members or friends to stay motivated to change the behaviour<br>Preparing your own meal as possible when you outside the house<br>Explain and kindly refuse any meal would hurt your diet (many dates, rice, sweets…etc.)	Having social support, e.g., family members or friends, to stay motivated to change the behaviour<br>Preparing your own meal as possible when you outside the house<br>Explain and kindly refuse any meal that would hurt your diet e.g., unsaturated fats	Having social support, e.g., family members or friends, to stay motivated to change the behaviour<br>Meeting people at parks to walk together	Having social support, e.g., family members or friends, to stay motivated to change the behaviour
	Physical	Environmental context and resources	Avoid eating fast food as possible<br>Buy food that contains many fibres	Avoid eating fast food as possible<br>Buy food that contains unsaturated fats	Register in a fitness centre, if possible, to avoid any environmental restrictions<br>Buy essential PA equipment, if possible, to avoid any environmental restrictions<br>Have daily time for PA<br>Do the most suitable and enjoyable exercise	Have a tape to measure waist circumference

Motivation	Reflective	Beliefs about own capability<br><br>Beliefs consequences<br><br>Social role and identity	Beliefs about own capability:<br><br>Have willpower and self-confidence in the ability to make the change<br><br>Have alternatives to cope with financial incapability<br><br>Beliefs about consequences:<br><br>Understand the negative impacts of much carbohydrate intake<br><br>Understand the risk factors for developing other diseases<br><br>Social Role and Identity:<br><br>Address the social barrier (carbohydrate portion size, like using a spoon)	Beliefs about own capability:<br><br>Have willpower and self-confidence in the ability to make the change<br><br>Have alternatives to cope with financial incapability<br><br>Beliefs about consequences: Understand the negative impacts of food containing saturated fats<br><br>Understand the risk factors of having saturated fats in developing other diseases<br><br>Social Role and Identity:<br><br>Address the social barrier (using unsaturated fats for cooking)	Beliefs about own capability:<br><br>Have willpower and self-confidence in the ability to do the exercise<br><br>Beliefs about consequences:<br><br>Overcome the fears of injury<br>Understand the risk factors of being physically inactive<br><br>Social Role and Identity:<br><br>Address the social barrier (overcome social barriers related to PA)	Change self-view of waist circumferences<br>Beliefs about consequences:<br>Understand the significance of accomplishing the waist circumference target

peer revie

Phase #	***Session Content***	***Session Goals***	***Intervention function***
1	• Session #1, the patient's assessment form and consent supposed to be completed<br>• General introduction about the health coaching intervention<br>• Outline the intervention structure and content<br>• Discuss the coach's roles and the expectations from the participant (being completely clear with the client about the health coaching)<br>• Creating an alliance (Establish Trust)<br>• Learn from a patient (diseases history, obstacles, priorities, strengths, goals... etc)<br>• Help patient to create wellness vision<br>• Assess the readiness of patient's stage in relation to change health behaviour (the transtheoretical model)	• Identifying the patient's current position in the overall health status (via the transtheoretical model)<br>• Build up a relationship (as an ally) between patient and coach<br>• Encourage open discussion<br>• Make sure the patient understands what health coaching is<br>• Allow the patient to develop a foundational conception of goal setting and action planning<br>• The patient creates (SMART) Specific, Measurable, Action-based, Realistic and Timely achievable goals<br>• Have better control of carbohydrate and fat intake<br>• Gradually increase physical activity<br>• Gradually increase achievable tasks until the intended behaviour is achieved	• Education<br>• Enablement<br>• Training<br>• Restriction<br>• Environmental restructuring

Supplemental Table 3:Intervention operation protocol

	• Introduce the importance of having a healthy diet<br>• Introduce the importance of increasing physical activity<br>• Increase awareness of adopting a healthy lifestyle in relation to controlling diabetes<br>• Identify 3-month general behavioural goals, and biweekly goals<br>• Explore resources needed to help achieve desirable behaviour<br>• Commitment		
2	• Phase #2 (session # 2), (this phase will be used again in session # 4 & 5)<br>• Check ongoing progresses<br>• Understand patient's state (use reflections)<br>• Ask patient to share views (so far) good things occurred and experience from last session<br>• Use positive reflections about patient's strengths, passion or emotions<br>• Ask patient to assess the previous selected short-goals and accomplishments<br>• Use reflections to understand potential barriers prevent patient from achieving past goals<br>• Identify specific strategies that they may use to overcome the obstacles<br>• Explore what patient learned from past experience<br>• Ask and discuss with patient next short goals<br>• Share feedback on patient's progression<br>• Ask patient to connect current accomplishments to the general 3-montπhs goals<br>• Review the general goals to see if patient want to revise them (to be more realistic and achievable)<br>• Affirm the patient's choices, strengths, and capability<br>• Use techniques such as reflective listening to address ambivalence and respond to the patient's resistance (motivational interviewing)	• Assessment of progression<br>• Review goal setting (behaviour)<br>• Review behaviour goals to examine a patient's performance progression toward the agreed goals<br>• Enable patient to develop problem-solving skills<br>• Enable patient to create action plan<br>• Prompt the participant to generate ideas and strategies to overcome barriers (problem-solving)<br>• Allow patients to monitor their behaviours (know the changes so far)<br>• Keep patient motivated (no matter the accomplishments)	• Persuasion<br>• Education<br>• Enablement<br>• Training<br>• Restriction<br>• Environmental restructuring

For peer review only

3	• Middle phase (session # 3), the coach continues to observe the patient and give feedback to help them move forward in achieving their goals through bi-weekly SMART goal setting.<br>• Patient continues to identify strategies to address existing obstacles and enhance their self-ability<br>• If goals are not achieved, the barriers will be identified, action plan will be taken to address these obstacles and modified goals will be cearted.<br>• The coach will assess the patient's self-efficacy by scoring goals to measure the participant's confidence in achieving their goals<br>• The coach continues using the skills needed to explore ambivalence and discrepancies between the participant's plans and their actual behaviour (Rollnick et al, 2005)<br>• Affirmations and appreciative inquiry will be used to appreciate progression and improve patients' self- confidence	• Assessment of current behavioural change<br>• Review goal setting (behaviour)<br>• Review behaviour goals to examine a patient's performance progression toward the agreed goals<br>• Review all previous goals and reassess goal progress<br>• Enable the participant to assess their progress<br>• Prompt the participant to analyse factors influencing their behaviour<br>• Participant's commitment to affirm to review and change behaviour	• Persuasion<br>• Education<br>• Enablement<br>• Training
4	• (session # 6)<br>• Conclude the coaching relationship<br>• Determine where the patient is in terms of their goals<br>• How the coach can best guide the client, and whether coaching is what will best benefit the client<br>• Participants' assessment of the intervention, general satisfaction of the participant with the process<br>• Appreciate the patient's engagement in the intervention<br>• Explore the patients' experience and how future coaching intervention would best support T2DM	• Allow patient to explore the difference at the endpoint<br>• Learn for participants' experience<br>• Findings from the pilot study will be used to justify an expansion of the study (full-scale) or refined for better outcomes so that we can carry out a large RCT on the efficacy of this intervention	• Persuasion<br>• Education

Supplementary Table 4: Joint display table

Key concepts	Predetermined progression criteria	Predetermined Cut-off criteria results	Quantitative results	Qualitative results	Integration result (Morgan, 2013).	Integration (meta-inference results: converge, diverge, or extend)

Screening prospective participants	If 60–80% or more of those eligible to participate in the study consented, this supports conducting a large RCT scale trial, but if the percentage is less than 50%, there is no significance moving forward.	> 80% screening rate (this suggests proceeding to the future definitive RCT)	38 out of 42 participants were eligible to participate in the study (screening rate: 90.5%)	No data	=	The screening rate was high and exceeded the study progression criteria.<br>The study's inclusion criteria were appropriate to target the potential population.
Recruitment rate	If the recruitment rate of people who were eligible and consented was ≥80%, this would support conducting a large RCT trial. If the rate was between 70–65%, this needs further discussion to explore the	> 80% recruitment rate (this suggests proceeding to the future definitive RCT)	30 out of 30 participants consented and enrolled in the study (recruitment rate: 100%)	*Theme 1: subtheme 1: participation and recruitment:*<br><br>*"…the recruitment should not be difficult in normal conditions…"* (FGC, C01, Female)<br><br>*".. recruiting more people will be achievable based on what I've seen*	=	Converge<br><br>Justification:<br><br>The recruitment rate was high and exceeded the study progression criteria (all eligible participants agreed and enrolled in the intervention).<br><br>Qualitative data confirmed that participants were happy to join the intervention and they other relatives and friends to join.

	reasons and whether, if they can be modified, then the trial may progress with caution. If the rate was ≤65%, there is no significance to moving forward.			*from participants' reactions..''* (FGC, C02, Female)<br><br>*".. some participants asked if it is possible to enroll some of their relatives or friends in the program..''* (FGC, C03, Female)		From the researcher's observations, there were a number of calls from other people requesting to join in the future
Retention rate at 3-months	If the rate was ≥83%, this supports conducting a large RCT scale trial. If the rate was less than 83%, there is no significance to moving forward.	> 83 % retention rate at 3-months (this suggests proceeding to the future definitive RCT)	29 out of 30 participants completed the study (100%).<br><br>Only one participant has withdrawn <u>before</u> the first session	Only one person withdrew before the first session. The participants reached out to explore the possible reasons, which involved a family concern.<br><br>Participants emphasised the importance of commitment as they valued participation in the intervention and	=	Converge<br><br>Justification:<br><br>Given the circumstances and restrictions imposed such as multiple lockdowns due to COVID-19, the retention rate was high. This fact was emphasised by the participants' experience (QUAL) during the intervention.

For peer review only

				believed the opportunity should only be given to a committed person.<br><br>"*you should ask them at the beginning if they have any intention to withdraw the program and prevent someone from having this opportunity …*" (FG, P09, Male)		
Intervention adherence	If the adherence rate was ≥84% of the intervention (≥5 out of the 6 coaching sessions). If the rate was between 84–67% (≥5.4 out of the 6 coaching sessions), this needs further discussion to explore the	>84% of the intervention (≥ 5 out of the 6 coaching sessions) (this suggests proceeding to the future definitive RCT)	All participant in the intervention group completed all their coaching sessions (adherence rate 100%)	*Theme 2: subtheme 4: reach and dose of the intervention:*<br><br>All participants completed their sessions despites the 11 rescheduled sessions. In general, they were happy and requested to have more sessions<br><br>"*…I suggested more sessions, a*	=	Converge<br><br>Justification:<br><br>The adherence rate exceeded the progression criteria<br><br>Qualitative data emphasised and explained the high adherence rate<br><br>Many participants expressed their desires by suggesting to increase the number of coaching sessions

For peer review only

	reasons and whether, if they can be modified, then the trial may progress with caution. If the adherence rate was less than 67%, there is no significance in moving forward.			*maximum of six months, to be able to monitor the impacts and progress that a participant achieves.. as I told you before, some of the participants asked me to continue with them to help them maintain progress..”* (FGC, C03, Female)<br><br>*“.. I prefer face-to-face sessions and more than 3 months in order to maintain the progress I make”* (P9, Male)		The way health coaches delivered the sessions contributed in improving participant adherence and commitment.
Secondary measurements acceptability	No data	No data	All participants completed the measurements' assessments at the study baseline and endpoint:	HbA1C, blood pressure, body mass index (BMI), weight and waist circumference.<br><br>Coaches believed the selected		Converge<br><br>Justification:<br><br>Despite the restrictions imposed due to COVID-19, the adherence to data collection was high as all participants in both groups completed their assessments in the planned time

For peer review only

			At the baseline: 30 out of 30 (100%)<br><br>At the endpoint: 29 out of 30 (96.6%)<br><br>Only one participant has withdrawn before the first session	outcomes to measure were appropriate<br><br>*"… measurements served well the main purpose of the program (diabetes).. no need to add new measurements."* (FGC, C01, Female)<br><br>We asked participants about their experience with baseline and endpoint measurements assessments.<br><br>*".. I think the same way you did the assessments; it was easy and straightforward.."* (FGP, P03, Female)	=	Coaches agreed that the secondary outcome assessments were adequate as it covered the intervention purpose<br><br>Qualitative data confirmed this, as participants felt the data collection process was acceptable
Intervention preliminary	No data	No data	Observed improvements were seen across	Participants were happy about the		Converge<br><br>Justification:

For peer review only

efficacy and tangible effects			different secondary outcomes:<br>1- Significant reduction in HbA1C favouring health coaching group: -1.86 (95% CI: -2.71 to -1.01, P<0.001)<br>2- Weight improvement was seen more in the intervention group : -2.58 (95% CI: -5.25 to .082, P= 0.057)<br>3- BMI improvement was seen more in the intervention group :<br>-1.02 (95% CI: -2.01 to -.041, P=0.042)<br>4- larger reduction was seen in the intervention group: -6.89 (95% CI: -10.17	intervention's tangible effects.<br><br>*".. I' 'm happy because my weight decreased by 3 kg … Also, my blood sugar improved. Before the intervention, the fasting blood sugar was 147 but now 107.."* (P03, Female)<br><br>*".. number talks, when I started the program, the A1c was 11.5% and now is 5.8%.."* (P13, Male)	=	Qualitative data confirmed the improvements in the quantitative results

For peer review only

			to -3.61, P<0.001)<br>5- The patients' self-efficacy questionnaire revealed that self-efficacy improved in both groups,but was much greater in the intervention group.			
Intervention experience	No data	No data	The overall session length per patient ranged from 106 to 153 minutes, with an average of 120.8 (SD= 13.7)<br><br>Among 36 BCTs, the most used BCTs:<br><br>*1.3 Goal setting (outcome),* which has been used 43 times with three behaviours targets; decrease	Participants felt the intervention was easy to follow and fits the Saudi Context<br><br>“ *.. it is an appropriate program, especially now while our society and new generations are changing..”* (P03, female)<br><br>Coaches believed that the curriculum including the	=	Converge<br><br>Justification:<br><br>Qualitative data confirmed and explained quantitative results about participants’ and health coaches’ experience.<br><br>Participants found the intervention easy to follow and fits the local context<br><br>Health coaches felt the curriculum was doable and helpful

For peer review only

			carbohydrate intake for each meal, exercise for 30 min five days weekly, and monitor waist circumference *1.7 Review outcome goal (s)* was the second most frequent technique (reported 39 times)<br><br>Participants responded to one of the related question (To what extent did participation in health coaching:<br><br>1- Increase your use of goal setting skill?<br><br>12 participants responded with	COM-B model and BCTs helped them deliver each session as planned<br><br>*".. I followed exactly the intervention guidance… you know .. I used the schedule in each session so it helped me to remember the content of each session and the goals, and skills that need to be discussed with a participant.. it helped a lot to prepare myself before each session…"* (FGC, C03, Female)<br><br>*" ..it was useful [the curriculum]… you know to have proposed techniques targeting different*		

			"Very Much" and 2 responded with "Quite a bit"<br><br>2-Increase your use of problem-solving skills?<br><br>8 participants responded with "Very Much" and 5 responded with "Quite a bit" and only one participant responded with "Somewhat"	*behaviours, so as a coach, you know you do not need to waste time on this… I just needed to select appropriate techniques to build new behaviours or change others.."* (FGC, C01, Female)<br><br>*"..your program helped me use some new skills such as setting plans and having short-term and long-term goals.."* (P08, Male)		
Satisfaction with the intervention	No data	No data	Likert-scale Satisfaction Questionnaire revealed that the participants were highly satisfied with the intervention	Overall, participants were satisfied.<br><br>*".. I'm very very very satisfied and I think the program*		Converge<br><br>Justification:<br><br>Qualitative data confirmed quantitative results about participants' satisfaction

For peer review only

			(Likert scale questions: 0 (not at all) to 10. (very much)). Participant satisfaction average rates ranged from 8.14 to 9.21 out of 10.<br><br>The majority (n=13, 92.9%) responded with "Yes" the intervention met their expectations<br><br>When asked if they would recommend the intervention to others, all participants (n=14, 100%) responded with "yes"	*is easy to follow and very acceptable..”* (P13, Male)<br><br>*“.. many of my surrounding people have known about the program and I highly recommend it to them…. I'm very satisfied..”* (P03, Female)<br><br>*“If I were asked to choose one [usual care vs health coaching], I would choose this program without hesitation..”* P03, Female)<br><br>*“ I suggest that and you must have a role in the hospital; why do not you have a clinic in the hospital so other patients can have an opportunity to sit with a coach..*	=	

				I'm willing if you want me to make a communication with the health authority and request this…" (P13, Male)		
Flexibility in scheduling coaching sessions	No data	No data	Out of 84 sessions, only eleven sessions have been rescheduled (13%)	The intervention flexibility helped participants adhere to and complete their sessions. *"…the thing that helped me a lot was the flexibility and easy communication…"* " *the most challenging thing was the conflict between my routine duty at work and the intervention, especially when participants ask me to reschedule a session.*	=	Converge and qualitative data further expand justifications for rescheduling sessions for participants. The flexibility helped participants but on the other hand, added load on coaches

For peer review only

				Participants should commit to their scheduled sessions as they can" (FGC, C01, Female)		

For peer review only

Supplemental Table 5: Time spent in each coaching session

Session# / Participant #	1	2	3	4	5	6	TOTAL (minutes)	Mean (minutes)
**P01**	35	22	12	15	12	37	133	22.2

**P06**	27	18	17	14	17	25	118	19.7
**P09**	29	17	10	14	12	24	106	17.7
**P10**	30	12	14	13	12	30	111	18.5
**P03**	32	19	19	12	11	33	126	21
**P04**	27	15	11	15	10	35	113	18.8
**P07**	33	11	10	14	11	30	109	18.2
**P02**	35	12	10	11	10	33	111	18.5
**P05**	31	14	11	12	10	30	108	18
**P11**	32	18	16	12	15	40	133	22.2
**P08**	35	13	15	10	12	31	116	19.3
**P14**	40	13	11	12	10	31	117	19.5
**P15**	44	22	10	15	12	34	137	22.8
**P13**	43	15	13	17	15	50	153	25.5
**TOTAL (minutes)**	473	221	179	186	169	463	1691	
**Mean**	33.8	15.8	12.8	13.3	12.1	33.1		
**Median**	32.5	15	11.5	13.5	12	32		

For peer review only

**Range**	17 (27-44)	11 (12-22)	9 (10-19)	7 (10-17)	7 (10-17)	26 (24-50)		

For peer review only

Supplemental Table 6: Feasibility Measurements and Predetermined progression criteria

Measure	Definition	Result	Predetermined progression criteria	Notes
**Screening rate**	Number of screened participants that met the inclusion criteria	38 out of 42 (91%)	> 80% screening rate (this suggests proceeding to the future definitive RCT)	The target approached participants was adjusted due to the COVID-19 pandemic

**Recruitment rate**	Number of recruited participants out of the total participants who were eligible during recruited phase	30 out of 30 (100%)	> 80% screening rate (this suggests proceeding to the future definitive RCT)	Despite the COVID-19 epidemic, the recruitment rate standard was reached
**Retention rate at 3-months**	Number of participants who completed the trial out of the total sample	29 out of 30 (96.6%)	> 83 % retention rate at Three months (this suggests proceeding to the future definitive RCT)	The retention rate at 3-months was high (all participants who took the first session have completed all six sessions)
**Bassline data collection adherence**	Number of participants who completed the assessments at the intervention baseline out of the total study sample	30 out of 30 (100%)		All participants have completed study assessments at the baseline
**Intervention adherence (coaching group only)**	Number of participants who completed 5 out of six coaching sessions ≥ 84%	6 out of 6 sessions per participant (100%)	>84% of the intervention (≥ 5 out of the six coaching sessions) (this suggests proceeding to the future definitive RCT)	
**Endpoint data collection adherence**	Number of participants who completed the assessments at the intervention endpoint out of the total study sample	29 out of 30 participants (96.6%)		Only one participant has withdrawn before the first session

Supplemental table 7: Participant ratings of health coaching on a 10-Point Likert Scale (intervention group only)

Question	N	Mean [a]	Std. Deviation	Range

For peer review only - http://bmjopen.bmj.com/site/about/guidelines.xhtml

To what extent did participation in health coaching:				
1. Changed you/your behaviour	14	8.14	2.381	3-10
2. Helped create a personal vision of wellness	14	9.21	1.369	6-10
3. Increase your confidence to take steps to greater wellness	14	9.21	1.369	6-10
4. Increase your motivation to take steps to greater wellness	14	9.14	1.351	6-10
5. Increase your use of goal-setting skills	14	9.21	.893	7-10
6. Increase your use of problem-solving skills	14	8.57	1.399	5-10
7. Help get you back on track after running into barriers	14	9.07	1.072	7-10
8. Help you achieve greater wellness	14	9.14	1.099	7-10

[a] Scale of 0 to 10, with 0 (not at all) to 10 (very much)

peer review only

Supplemental Table 8: Frequency of used BCTs to each behaviour target

	BCT	Behaviour target (s)	Frequency Used
1.	*1.1 Goal setting (Behaviour)*	• Decrease carbohydrate intake for each meal<br>• Use unsaturated fats as possible (avoid saturated fats)<br>• Do exercise for 30 min, five days on a weekly basis<br>• Monitor waist circumference	43
2.	*1.2 Problem-solving*	• Decrease carbohydrate intake for each meal<br>• Use unsaturated fats as possible (avoid saturated fats)<br>• Do exercise for 30 min, five days on a weekly basis	28
3.	*1.3 Goal setting (outcome)*	• Decrease carbohydrate intake for each meal<br>• Do exercise for 30 min, five days on a weekly basis<br>• Monitor waist circumference	34
4.	*1.4 Action planning*	• Decrease carbohydrate intake for each meal<br>• Do exercise for 30 min, five days on a weekly basis	27
5.	*1.5 Review behaviour goal (s)*	• Decrease carbohydrate intake for each meal<br>• Do exercise for 30 min, five days on a weekly basis	34

For peer review only

	BCT	Behaviour target (s)	Frequency Used
6.	*1.7 Review outcome goal (s)*	• Decrease carbohydrate intake for each meal<br>• Do exercise for 30 min, five days on a weekly basis	39
7.	*1.9 Commitment*	• Decrease carbohydrate intake for each meal<br>• Use unsaturated fats as possible (avoid saturated fats)<br>• Do exercise for 30 min, five days on a weekly basis	18
8.	*2.2 Feedback on behaviour*	• Decrease carbohydrate intake for each meal	17
9.	*2.3 Self-monitoring of behaviour*	• Decrease carbohydrate intake for each meal<br>• Do exercise for 30 min, five days on a weekly basis	15
10.	*2.7. Feedback on outcome(s) of behaviour*	• Decrease carbohydrate intake for each meal<br>• Use unsaturated fats as possible (avoid saturated fats)<br>• Do exercise for 30 min, five days on a weekly basis<br>• Monitor waist circumference	22
11.	*3.1 Social support (unspecified)*	• Decrease carbohydrate intake for each meal<br>• Use unsaturated fats as possible (avoid saturated fats)<br>• Do exercise for 30 min, five days on a weekly basis	29

For peer review only

	BCT	Behaviour target (s)	Frequency Used
12.	*4.1 Instruction on how to perform a behaviour*	• Decrease carbohydrate intake for each meal<br>• Do exercise for 30 min, five days on a weekly basis<br>• Monitor waist circumference	14
13.	*5.1Information about health consequences*	• Decrease carbohydrate intake for each meal<br>• Use unsaturated fats as possible (avoid saturated fats)<br>• Do exercise for 30 min, five days on a weekly basis<br>• Monitor waist circumference	27
14.	*5.3 Information about social and environmental consequences*	• Decrease carbohydrate intake for each meal<br>• Use unsaturated fats as possible (avoid saturated fats)<br>• Do exercise for 30 min, five days on a weekly basis	19
15.	*5.5 Anticipated regret*	• Decrease carbohydrate intake for each meal<br>• Do exercise for 30 min, five days on a weekly basis	15
16.	*5.6 Information about emotional consequences*	• Decrease carbohydrate intake for each meal<br>• Do exercise for 30 min, five days on a weekly basis	11
17.	*6.1 Demonstration of the behaviour*	• Decrease carbohydrate intake for each meal<br>• Use unsaturated fats as possible (avoid saturated fats)	14

For peer review only

	BCT	Behaviour target (s)	Frequency Used
		• Do exercise for 30 min, five days on a weekly basis	
18.	*6.3 Information about others' approval*	• Decrease carbohydrate intake for each meal<br>• Use unsaturated fats as possible (avoid saturated fats)<br>• Do exercise for 30 min, five days on a weekly basis	14
19.	*8.1 Behavioural practice/rehearsal*	• Decrease carbohydrate intake for each meal	22
20.	*8.3 Habit Formation*	• Decrease carbohydrate intake for each meal<br>• Use unsaturated fats as possible (avoid saturated fats)<br>• Do exercise for 30 min, five days on a weekly basis	28
21.	*8.7 Graded tasks*	• Decrease carbohydrate intake for each meal<br>• Use unsaturated fats as possible (avoid saturated fats)<br>• Do exercise for 30 min, five days on a weekly basis	31
22.	*9.1Credible source*	• Decrease carbohydrate intake for each meal<br>• Do exercise for 30 min, five days on a weekly basis	17

For peer review only

	BCT	Behaviour target (s)	Frequency Used
23.	*9.2 Pros and cons*	• Decrease carbohydrate intake for each meal<br>• Use unsaturated fats as possible (avoid saturated fats)<br>• Do exercise for 30 min, five days on a weekly basis	15
24.	*7.1 Prompts/Cues*	• Decrease carbohydrate intake for each meal<br>• Do exercise for 30 min, five days on a weekly basis	9
25.	*9.3 Comparative imagining of future outcomes*	• Decrease carbohydrate intake for each meal<br>• Use unsaturated fats as possible (avoid saturated fats)<br>• Do exercise for 30 min, five days on a weekly basis<br>• Monitor waist circumference	27
26.	*10.9 Self-reward*	• Decrease carbohydrate intake for each meal<br>• Do exercise for 30 min, five days on a weekly basis	8
27.	*12.1 Restructuring the physical environment*	• Decrease carbohydrate intake for each meal<br>• Use unsaturated fats as possible (avoid saturated fats)<br>• Do exercise for 30 min, five days on a weekly basis	26

For peer review only

	BCT	Behaviour target (s)	Frequency Used
28.	*12.2 Restructuring the social environment*	• Decrease carbohydrate intake for each meal<br>• Use unsaturated fats as possible (avoid saturated fats)<br>• Do exercise for 30 min, five days on a weekly basis	28
29.	*12.3Avoidance/reducing exposure to cues for the behaviour*	• Decrease carbohydrate intake for each meal<br>• Use unsaturated fats as possible (avoid saturated fats)	18
30.	*12.5 Adding objects to the environment*	• Decrease carbohydrate intake for each meal<br>• Use unsaturated fats as possible (avoid saturated fats)<br>• Do exercise for 30 min, five days on a weekly basis	16
31.	*13.1 Identification of self as role model*	• Decrease carbohydrate intake for each meal<br>• Use unsaturated fats as possible (avoid saturated fats)<br>• Do exercise for 30 min, five days on a weekly basis	14
32.	*13.2 Framing/reframing*	• Decrease carbohydrate intake for each meal<br>• Do exercise for 30 min, five days on a weekly basis	16

For peer review only

	BCT	Behaviour target (s)	Frequency Used
33.	*15.1 Verbal persuasion about capability*	• Decrease carbohydrate intake for each meal<br>• Use unsaturated fats as possible (avoid saturated fats)<br>• Do exercise for 30 min, five days on a weekly basis<br>• Monitor waist circumference	30
34.	*15.2 Mental rehearsal of successful performance*	• Decrease carbohydrate intake for each meal<br>• Do exercise for 30 min, five days on a weekly basis	17
35.	*15.3 Focus on past success*	• Decrease carbohydrate intake for each meal<br>• Use unsaturated fats as possible (avoid saturated fats)<br>• Do exercise for 30 min, five days on a weekly basis	24
36.	*15.4 Self-talk*	• Decrease carbohydrate intake for each meal	7

Supplemental Figure 2: Thematic analysis map

For peer review only

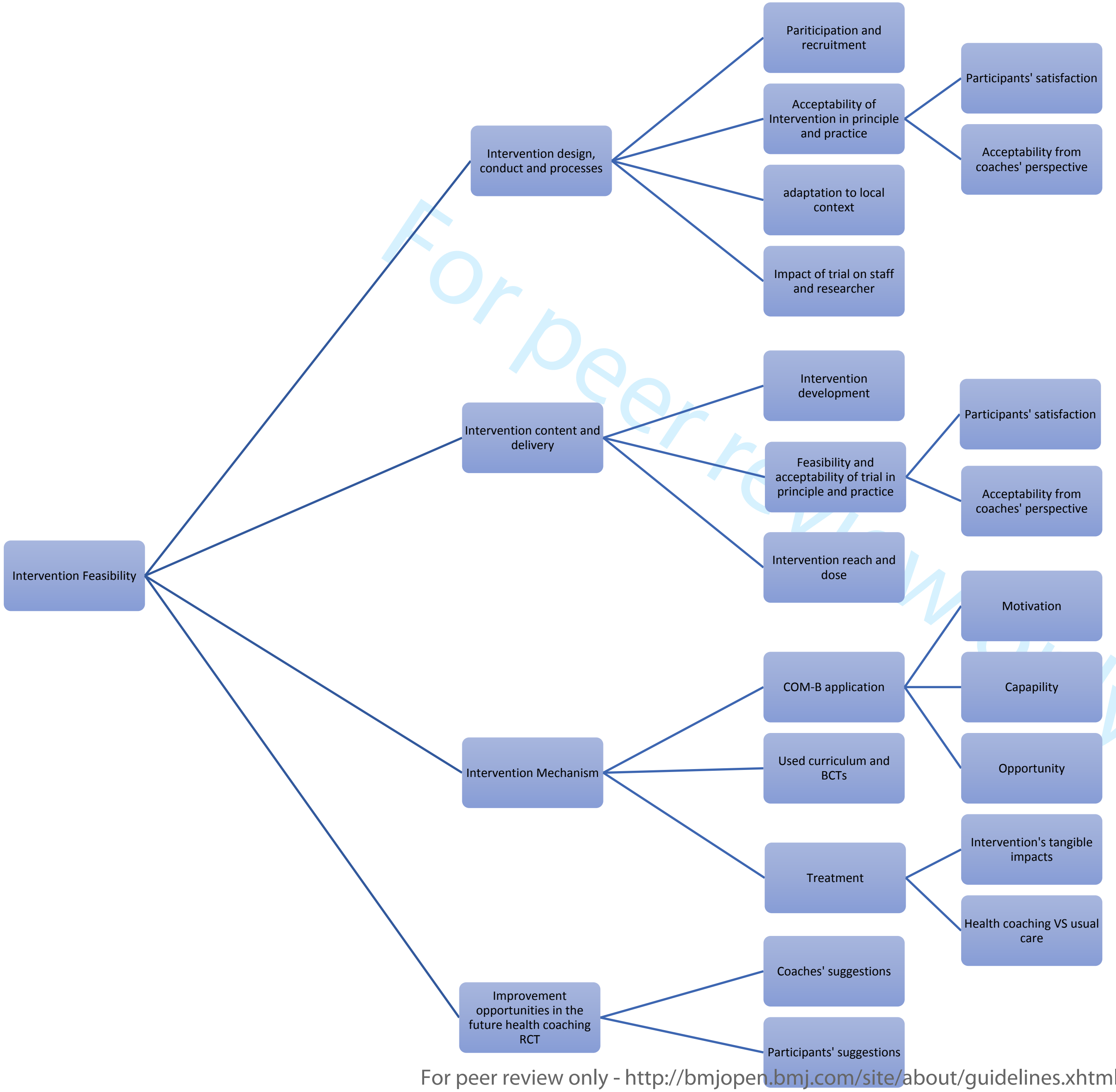
Intervention Feasibility
Intervention design, conduct and processes
Pariticipation and recruitment
Acceptability of Intervention in principle and practice
Participants' satisfaction
Acceptability from coaches' perspective
adaptation to local context
Impact of trial on staff and researcher
Intervention content and delivery
Intervention development
Feasibility and acceptability of trial in principle and practice
Participants' satisfaction
Acceptability from coaches' perspective
Intervention reach and dose
Intervention Mechanism
COM-B application
Motivation
Capapility
Opportunity
Used curriculum and BCTs
Treatment
Intervention's tangible impacts
Health coaching VS usual care
Improvement opportunities in the future health coaching RCT
Coaches' suggestions
Participants' suggestions

Supplemental Table 9: Participants' mean differences in each group at baseline and endpoint for patients' self-efficacy questionnaire

	Group	N	Mean	Std. Deviation	An independent two-sample t-test Sig. (2-tailed)	Mean Difference	95% Confidence Interval of the Difference	
How confident do you feel that you can eat your meals every 4 to 5 hours every day, including breakfast every day? (At the baseline)	Intervention	14	5.36	3.296	.445	.757	-1.249	2.763
	Control	15	4.60	1.805				
How confident do you feel that you can eat your meals every 4 to 5 hours every day, including breakfast every day? (At endpoint)	Intervention	14	7.21	2.082	.505	.481	-.981	1.943
	Control	15	6.73	1.751				
How confident do you feel that you can follow your diet when you have to prepare or share food with other people who do not have diabetes? (At the baseline)	Intervention	14	5.29	3.688	.891	.152	-2.114	2.419
	Control	15	5.13	2.100				
How confident do you feel that you can follow your diet when you have to prepare or share food with other people who do not have diabetes? (At endpoint)	Intervention	14	8.07	1.979	.274	.871	-.732	2.474
	Control	15	7.20	2.210				
How confident do you feel that you can choose the appropriate foods to eat when you are hungry (for example, snacks)? (At the baseline)	Intervention	14	6.07	3.174	.569	.538	-1.379	2.455
	Control	15	5.53	1.685				

How confident do you feel that you can choose the appropriate foods to eat when you are hungry (for example, snacks)? (At endpoint)	Intervention	14	8.21	2.082	.144	1.014	-.368	2.397
	Control	15	7.20	1.521				
How confident do you feel that you can exercise 15 to 30 minutes, 4 to 5 times a week? (At the baseline)	Intervention	14	6.50	3.632	.217	1.500	-.936	3.936
	Control	15	5.00	2.726				
How confident do you feel that you can exercise 15 to 30 minutes, 4 to 5 times a week? (At endpoint)	Intervention	14	9.14	1.994	.006	2.276	.713	3.839
	Control	15	6.87	2.100				
How confident do you feel that you can do something to prevent your blood sugar level from dropping when you exercise? (At the baseline)	Intervention	14	6.71	3.148	.072	1.848	-.178	3.873
	Control	15	4.87	2.100				
How confident do you feel that you can do something to prevent your blood sugar level from dropping when you exercise? (At endpoint)	Intervention	14	8.71	2.054	.000	3.048	1.493	4.602
	Control	15	5.67	2.024				
How confident do you feel that you know what to do when your blood sugar level goes higher or lower than it should be? (At the baseline)	Intervention	14	5.71	2.867	.616	.448	-1.360	2.256
	Control	15	5.27	1.792				
How confident do you feel that you know what to do when your blood sugar level goes higher or lower than it should be? (At endpoint)	Intervention	14	8.36	2.023	.006	2.157	.678	3.636
	Control	15	6.20	1.859				
How confident do you feel that you can judge when the	Intervention	14	6.93	2.786	.284	.995	-.872	2.862

changes in your illness mean you should visit the doctor? (At the baseline)	Control	15	5.93	2.086				
How confident do you feel that you can judge when the changes in your illness mean you should visit the doctor? (At endpoint)	Intervention	14	9.43	1.016	.000	3.295	2.075	4.516
	Control	15	6.13	1.995				
How confident do you feel that you can control your diabetes so that it does not interfere with the things you want to do? (At the baseline)	Intervention	14	5.93	3.125	.660	.462	-1.668	2.592
	Control	15	5.47	2.446				
How confident do you feel that you can control your diabetes so that it does not interfere with the things you want to do? (At endpoint)	Intervention	14	8.86	2.033	.006	2.590	.826	4.355
	Control	15	6.27	2.549				

Supplemental Table 10: Participants' mean differences in each group at baseline and endpoint for diabetes self-care activity questionnaire

An independent two-sample t-test

	Group	N	Mean	Std. Deviation	Sig. (2-tailed)	Mean Difference	95% Confidence Interval of the Difference	
On average, over the past month, how many days per week have you followed your eating plan? (At the baseline)	Intervention	14	2.36	2.098	.461	-.510	-1.909	.890
	Control	15	2.87	1.552				
On average, over the past month, how many days per week have you followed your eating plan? (At endpoint)	Intervention	14	4.86	1.610	.160	.790	-.333	1.914
	Control	15	4.07	1.335				
On how many of the last seven days did you eat five or more servings of fruits and vegetables? (At the baseline)	Intervention	14	2.29	1.590	.063	-1.181	-2.431	.069
	Control	15	3.47	1.685				
On how many of the last seven days did you eat five or more servings of fruits and vegetables? (At endpoint)	Intervention	14	3.93	1.385	.100	-.871	-1.923	.180
	Control	15	4.80	1.373				
On how many of the last seven days did you eat high fat foods such as red meat or full-fat dairy products? (At the baseline)	Intervention	14	2.50	1.912	.046	-1.167	-2.311	-.022
	Control	15	3.67	.976				
On how many of the last seven days did you eat high fat foods such as red meat or full-fat dairy products? (At endpoint)	Intervention	14	3.50	1.829	.323	-.633	-1.923	.656
	Control	15	4.13	1.552				
On how many of the last seven days did you space carbohydrates evenly through the day? (At the baseline)	Intervention	14	1.71	1.729	.236	-.686	-1.845	.474
	Control	15	2.40	1.298				
On how many of the last seven days did you space carbohydrates evenly through the day? (At endpoint)	Intervention	14	4.00	1.240	.258	-.533	-1.481	.415
	Control	15	4.53	1.246				
On how many of the last seven days have you followed a	Intervention	14	1.64	1.447	.011	-1.490	-2.617	-.364

healthful eating plan? (At the baseline)	Control	15	3.13	1.506				
On how many of the last seven days have you followed a healthful eating plan? (At endpoint)	Intervention	14	5.00	1.468	.452	.400	-.674	1.474
	Control	15	4.60	1.352				
On how many of the last seven days did you participate in at least 30 minutes of physical activity? (At the baseline)	Intervention	14	3.57	2.243	.674	.305	-1.165	1.775
	Control	15	3.27	1.580				
On how many of the last seven days did you participate in at least 30 minutes of physical activity? (At endpoint)	Intervention	14	5.64	1.692	.162	.776	-.331	1.884
	Control	15	4.87	1.187				
On how many of the last seven days did you participate in a specific exercise session (such as such swimming, walking, biking) other than what you do around the house or as part of your work? (At the baseline)	Intervention	14	2.29	2.016	.775	-.181	-1.465	1.103
	Control	15	2.47	1.302				
On how many of the last seven days did you participate in a specific exercise session (such as such swimming, walking, biking) other than what you do around the house or as part of your work? (At endpoint)	Intervention	14	4.93	2.018	.145	.929	-.342	2.199
	Control	15	4.00	1.254				

International Journal of
Environmental Research and Public Health

MDPI

Protocol

Assessing the Feasibility and Acceptability of Health Coaching as a New Diabetes Management Approach for the People with Type 2 Diabetes in Saudi Arabia: A Protocol for a Mixed Methods Feasibility Study

Abdullah N. Almulhim [1,2,*], Elizabeth Goyder [1] and Samantha J. Caton [1]

[1] School of Health and Related Research (ScHARR), University of Sheffield, Sheffield S1 4DA, UK
[2] Public Health Department, College of Health Sciences, Saudi Electronic University, Riyadh 13316, Saudi Arabia
* Correspondence: aalmulhim@seu.edu.sa

Abstract: Background: Over recent years, the Middle East, and especially Saudi Arabia, has faced multiple changes, including structural-demographic and economic shifts. This has led to massive changes in the population's lifestyle, including more unhealthy diets and increases in physical inactivity. As a result, accelerating rates of chronic diseases, including type 2 diabetes mellitus (T2DM) are a major public health concern. Current diabetes care in Saudi Arabia focuses on increasing the awareness of patients through various approaches, mainly based on health education, which is found to be suboptimal and ineffective for improving long-term outcomes. This study aims to assess the feasibility and acceptability of using a client-centred approach called health coaching that supports, enables, and engages T2DM patients to take the central role of controlling their own conditions by developing new crucial skills. Methods: A mixed methods randomised controlled feasibility study of health coaching will be used. Participants (n = 30) are adults with T2DM with poorly controlled diabetes (A1C $\geq$ 7) who can read and write in Arabic. Eligible participants are randomly allocated to either an intervention or control group for 12 weeks. COM-B model and Behaviour Change Technique Taxonomy version 1 (BCTTv1) guide the intervention curriculum. Predetermined progression criteria will be used to determine whether to proceed to a larger trial or not. Outcomes will be measured at baseline and 3 months. The study's primary aim is to assess the process of eligibility, recruitment, retention and completion rates, acceptability and suitability of intervention and the time to complete each procedure. The preliminary efficacy of health coaching is the secondary outcome that includes different measurements, such as HbA1c, blood pressure, body mass index (BMI), waist circumference, weight, patients' self-efficacy, and diabetes self-management. Discussion: This is the first study to explore the feasibility, acceptability, and preliminary efficacy of health coaching that used the Capability, Opportunity, Motivation, Behaviour (COM-B) model and BCTTv1 as guidance to develop the intervention for adults with T2DM in Saudi Arabia. The findings of this study will be used to inform the larger RCT trial if it is shown to be feasible and acceptable.

Keywords: health behaviour change; health coaching; self-management; behaviour change techniques; type 2 diabetes

check for updates

Citation: Almulhim, A.N.; Goyder, E.; Caton, S.J. Assessing the Feasibility and Acceptability of Health Coaching as a New Diabetes Management Approach for the People with Type 2 Diabetes in Saudi Arabia: A Protocol for a Mixed Methods Feasibility Study. *Int. J. Environ. Res. Public Health* **2022**, *19*, 15089. https://doi.org/10.3390/ijerph192215089

Academic Editor: Emanuela Orsi

Received: 4 October 2022
Accepted: 11 November 2022
Published: 16 November 2022

Publisher's Note: MDPI stays neutral with regard to jurisdictional claims in published maps and institutional affiliations.

Copyright: © 2022 by the authors. Licensee MDPI, Basel, Switzerland. This article is an open access article distributed under the terms and conditions of the Creative Commons Attribution (CC BY) license (https://creativecommons.org/licenses/by/4.0/).

1. Background

Type 2 diabetes mellitus (T2DM) is a widespread chronic disease that poses a real worldwide health threat that is becoming a significant public health concern. T2DM is profoundly impacted by an excess body mass index (BMI) and physical inactivity [1]. Diabetes contributes to lead patients developing severe complications such as heart and stroke disorders, eye problems and complications with hearing, kidney failure, nerve injury, amputations, oral issues, and foot problem [2]. Adults with diabetes are two or three

For peer review only - http://bmjopen.bmj.com/site/about/guidelines.xhtml

times more likely to develop heart problems and strokes [3]. In addition, there are other negative impacts of diabetes on social life, economic status, and health care services. Since diabetes is a preventable disease, which is strongly related to and affected by lifestyle, self-management is an optimal way to effectively manage it [4].

Worldwide, and especially in Saudi Arabia (SA), the prevalence of T2DM is growing at an alarming pace. According to a WHO report (2017), SA has the second highest rate of T2DM and is ranked the seventh highest in the globe. Additionally, the prevalence rate of T2DM has increased tenfold in the past three decades in SA. About 89% to 97% of all Saudi patients with diabetes are diagnosed with T2DM [5]. About seven million people suffer from T2DM in Saudi Arabia and three million are pre-diabetic. T2DM affects approximately 30% of the Saudi population and this increases with age [6]. The prevalence of T2DM is higher among women than men and with higher BMI individuals [6]. Moreover, it is more than likely that the burden of this chronic disease will only increase owing to factors such as an increased rate of obesity, an ageing population, changing lifestyles and poor diet [7]. The widespread increase in sedentary behaviour and physical inactivity among Saudis contributed to increasing the prevalence of T2DM [8] In SA the high rate of T2DM is associated with the high prevalence of obesity as a result of the fast urbanisation that led to adopting modern western dietary habits such as fast-food and increasing the level of physical inactivity [9].

The healthcare system in SA is still trying to address the alarming challenge of T2DM through several national programs and initiatives, but the outcome is still suboptimal. In SA, several studies have provided evidence that T2DM is not managed effectively [10]. While the current attempts are heavily reliant on awareness-raising campaigns, T2DM patients are still struggling to adopt a healthy diet and lifestyle to manage their conditions effectively. Adopting a healthy diet and lifestyle is an essential element of effective self-management of diabetes [11]. It is noteworthy that lifestyle interventions seem to be at least as effective as medications [12].

Diabetes education-based programs have not always been entirely adequate in bringing about the desired change. Many studies make it clear that awareness alone does not contribute to better self-management practices [13]. The responsibility of controlling T2DM is largely dependent on individual behaviour. T2DM patients are the key players to address this issue, so they need enough time to address their needs to improve their self-management skills. Healthcare providers often do not have adequate time for a face-to-face discussion with patients since regular visits at primary care clinics are usually restricted to 10 to 15 min, which leads to patients feeling negative, overwhelmed, and frustrated. Cost-effective interventions are needed to bridge the gap by listening to patients and helping them to overcome potential barriers. Developing a patient's self-management skills is crucial to delay or reducing the risk of T2DM. The occurrence of the chronic conditions associated with T2DM can be remarkably diminished or delayed—by 75% or more—with the help of healthier lifestyle behaviour choices made by patients [14]. Self-management interventions are needed that enable patients to control and manage T2DM [15] consistent with the current diabetes treatment philosophy that promotes patient-centred approaches [16].

There have been recent attempts to improve diabetes care in SA with T2DM through diabetes self-management programs. These programs are very uncommon and not gaining widespread adoption [17]. Most lifestyle interventions still rely on education as the core element to improve patients' self-care. The impact of diabetes education programs is suboptimal [17]. However, most studies have emphasised the significant need to shift the current attention toward employing new approaches that help patients to develop self-management skills. A recent systematic review, conducted on gulf cooperation council countries, included studies in T2DM self-management between 1996 and 2015 and found that none of the studies employed the skills needed to improve patients' self-management capability, such as problem-solving skills [5]. Moreover, none of the studies has employed a patient-centred approach to work with a patient as an ally rather than an educator. Client-

For peer review only - http://bmjopen.bmj.com/site/about/guidelines.xhtml

centred interventions that are tailored to the patient's needs are more likely to bring about desired outcomes [18].

Health coaching is a client-centred approach that is derived from different disciplines and behaviour change theories. It has become an emerging trend in the literature and is gaining widespread acceptance in the health promotion fields but this one-on-one type of tailored intervention has not yet been used in Saudi healthcare systems. Different studies have revealed that health coaching as an intervention centred on patients' values may provide added benefits to traditional T2DM education programmes [19]. It can be a highly effective and financially viable model when it focuses on improving patients' self-efficacy and important skills, including goal-setting, and problem-solving [20]. Health coaching has been applied in multiple different contexts and showed as an effective tool to improve diabetes self-management [21,22].

However, recent systemic reviews of randomised controlled trials utilising health coaching have reported mixed results, with some reporting that health coaching is effective, while others claim it is ineffective [21,22]. One of the explanations for the inconsistent reported findings across different studies is the lack of agreement on the active ingredients and content of effective health coaching interventions.

There is currently no consensus in the literature on designing an effective health coaching intervention, including the selection of a suitable theoretical basis and active ingredients, or behaviour change techniques [22]. In the absence of such consensus, there is uncertainty towards which coaching methods are more appropriate and effective to use including intervention content as well as the duration, length, and mode of delivery of sessions, particularly when applying health coaching in a new setting [23]. A lack of guidance, ineffective methods used to develop interventions, inappropriately selected intervention components and variation in the reporting of outcomes have been suggested to contribute to the mixed effectiveness of health coaching interventions [24–26]. Furthermore, health coaching interventions that have been developed and have succeeded in Western or other contexts may not be effective for people in different contexts such as SA. Consequently, such interventions may require adaptation and modification to make them more feasible, acceptable, and suitable in the context of Saudi society.

A feasibility study is highly recommended prior to a full-scale trial to test potential uncertainties, especially when a complex intervention is to be applied in a new context [27]. Feasibility studies help to explore specific issues such as study protocol, context-specific relevance, practicality, and the possibility of efficacy [27,28]. In addition, feasibility studies help examine the procedures' acceptability, assess the recruitment and retention process, and determine the appropriate sample size for a future full-scale trial [27].

To the best of our knowledge, none of the previous health coaching programs have used the BCW model and BCTs taxonomy as a guide to build the intervention content systematically in order to analyse and achieve planned outcomes. This is aside from the fact the application of diabetes self-management studies within the Saudi context is very uncommon and often focuses on providing health education [17]. Moreover, none of the self-management interventions have engaged stakeholders during the intervention development process, so the interventions may have failed to bring about the change and address the difficulties patients encounter in managing their diabetes [17].

As a result, the future full-scale intervention would be effectively refined and/or inactive ingredients replaced with other appropriate BCTs. Careful selection of theoretical framework and BCTs prior to the intervention being conducted is crucial and widely recommended [29].

1.1. Study Aims

The present feasibility study aims to determine whether the health coaching intervention for type 2 diabetes is feasible and acceptable to improve self-management and reduce HbA1C to inform a full-scale RCT trial in SA. The objectives of the study are categorised into the process evaluation objectives (primary) and outcome (secondary) objectives. The

For peer review only - http://bmjopen.bmj.com/site/about/guidelines.xhtml

process evaluation objectives are related to the implementation phase and mainly focus on the feasibility of processes and procedures of the study. The outcome objectives are related to the efficacy of the intervention.

1.1.1. Primary Objective: (Study Feasibility-Process Evaluation-Objectives)

To assess the feasibility of conducting a future definitive study to evaluate the effectiveness of using an adapted health coaching intervention in the Saudi context by:

1. Assessing recruitment, and retention rates, and estimating the effect size;
2. Assessing the implementation process, including data collection procedures;
3. Assessing and determining the sample size for the larger-scale trial using the findings of this feasibility study;
4. Exploring the acceptability and suitability of intervention through participants' perceptions of, and experiences with, the health coaching intervention.

1.1.2. Secondary Objective: (Outcome Objectives)

To assess the preliminary efficacy of health coaching to improve diabetes self-management and reduce HbA1C by evaluating its influence on participants' behaviours and their self-management ability compared to usual diabetes care by:

HbA1c and other variables will be assessed pre- and post-intervention at baseline and at 3 months. The intervention's clinical goal for T2DM self-management is an HbA1c level of <7.0%. Other variables will also be assessed; namely, glycosylated haemoglobin (HbA1C), blood pressure, body mass index (BMI), waist circumference, weight, patients' self-efficacy, and diabetes self-management.

2. Methods

The protocol of this study is following the guidance of CONSORT [30]. The methods are categorised into subsections presented below (see Supplementary File S1).

2.1. Study Location

Saudi Arabia will be the location of this study. The trial will only be conducted at one site, namely the obesity, endocrine, and metabolism centre at King Fahad Medical City (KFMC), the hub of the Riyadh Second Health Cluster. KFMC is one of Saudi Arabia's most significant and well-known medical cities, which serves the country's largest medical complex.

2.2. Trial Design

The present study will adopt a double-blind randomised two-arm feasibility trial to investigate the feasibility of a 3-month intervention for participants who have had difficulty managing their T2DM. Mixed methods will be employed in the study's design to gather and analyse both quantitative and qualitative data. A mixed methods approach allows the study to address additional research questions [31]. An RCT will be used since it is the best design for comparing the effectiveness of health coaching between the two groups [32]. The primary and secondary trial outcomes will be examined using various approaches, including questionnaires, interviews, focus groups, and clinical measures, to assess the intervention's feasibility, acceptability, and preliminary effects. The study evaluation will be conducted at two points: at the intervention's baseline and three months following the intervention (endpoint). To conduct this study, ethical approval was obtained by the University of Sheffield and the Institutional Review Board (IRB) committee form ethical approval at (KFMC) (IRB Log Number: 21-062E). The CONSORT flow diagram will describe the route participants take through the health coaching intervention, including the schedule of enrolment, interventions, and assessments [33].

2.3. Participants

The target population of this study is adult men with T2DM (HbA1c) ≥7.0%. Participants will be eligible for recruitment as per the following criteria:

For peer review only - http://bmjopen.bmj.com/site/about/guidelines.xhtml

2.4. Eligibility Criteria

- Aged >18 years old;
- The participant diagnosed with T2DM;
- Haemoglobin A1c (A1c) ≥7.0%;
- The participant can read and understand Arabic;
- The participant has access to a personal mobile phone/smartphone;
- The participant is willing to complete the intervention period;
- The participant is willing to remain in Riyadh.

Patients are not eligible if they: (1) had physical impairments that prevented them from participating in physical activity, (2) patients are unable to understand or unwilling to give their informed consent.

2.5. Recruitment

Recruitment will take place at a primary care centre at KFMC. Advertising for the intervention will use multiple methods, including posters, brushers, social media and through health care provider referrals. Interested individuals will be assessed for eligibility to participate in the intervention. Those who meet the eligibility criteria will be contacted to meet with the research team to briefly explain the study and answer any related questions. Interested individuals will be given a booklet that explained the study and its aims before signing the consent form. Participants will complete a short initial screening assessment, including demographic information and baseline-related laboratory reports.

2.6. Randomisation and Blinding

All recruited participants will be randomised on a 1:1 ratio into one of two groups. The randomisation process will be carried out by an independent person out of this study and used a computer to generate random numbers. To minimise selection bias, eligible participants will have an equal opportunity of being allocated to each group. The researchers will remain unaware of which group a participant had been assigned to before the baseline assessment. Participants will be informed which group they have been assigned to after the baseline assessment.

2.7. Sample Size

To the best of our knowledge, there was no previous health coaching RCT feasibility study in the field to use as a reference for the sample size. Thus, this study seeks to recruit at least 9% (n = 30) of the sample size required to carry out a full trial [34], with a minimum of 12 participants in each group [35]. Participants will be randomly allocated to either the coaching group (n = 15) or the control group (n = 15).

2.8. Intervention

2.8.1. Intervention Content (Intervention Group)

Health coaching in the context of T2DM can be characterised as a complex intervention due to the various direct and indirect interacting components that impact the intervention's outcomes. Health coaching, as a complex approach, has arisen from multiple different behaviour change theoretical bases. While complex intervention is not a straightforward study with direct clear causal and effects interaction, several crucial requirements and careful considerations are required when replicating it. This is aside from the fact that health coaching combines various disciplines such as psychology with numerous theories into one approach. Thus, we used the model and Theoretical Domains Framework (TDF) as guidance in adapting the intervention into a new context, namely Saudi Arabia.

The intervention is drawn on the COM-B model and TDF to provide a precise and systematic description of the health coaching content. COM-B model is the central part of the Behaviour Change Wheel (BCW) that helps in identifying the interactive process from; capability, opportunity, and motivation required to bring about the desired behaviours of an intervention [23]. The Theoretical Domains Framework (TDF) is a tool to provide further

For peer review only

comprehensive explanations that link the COM-B model for a better understanding of the needed changes and determining the target behaviour [36]. Several barriers have been identified based on a literature review of previous work and what needs to be changed to bring about the desired outcomes. In addition, different target behaviours have been identified that would lead to adopting a healthy diet and lifestyle as advocated by UK lifestyle guidelines [37]. To the best of our knowledge, there are no evidence-based guidelines in SA for T2DM to adopt a healthy diet and lifestyle. Therefore, the UK lifestyle guidelines will be used due to their relevance to the Saudi context [5,37]. The UK lifestyle guideline will be utilised to identify the possible behaviour targets. In order to specify target behaviours, a discussion will be conducted with health practitioners at the health centre (the intervention setting), including a dietician, to reach an agreement on the behaviours list. The discussion helps assess and prioritise the target behaviours according to their potential effects, and possibility to be measured and achieved [38].

The four proposed behaviour targets are [37]:

- Decrease carbohydrate intake for each meal;
- Use unsaturated fats as much as possible (avoid saturated fats);
- Do exercise for 30 min, five days in a week;
- Monitor waist circumference, maintain it below (80 cm for women and 94 cm for men).

Based on the results from COM-B and TDF behaviour analyses, different intervention functions will be identified and linked to the key barriers (see Table 1). Next, the intervention functions will be assessed for their suitability based on APEASE criteria; affordability, practicability, effectiveness/cost-effectiveness, acceptability, safety/side effects and equity, as recommended by the BCW guide [23].

As recommended by BCW guide, multiple BCTs could be used for each identified intervention function [23]. The BCW guide suggests the most common and less frequently used BCTs for each intervention function. Different possible BCTs were identified and included as a part of intervention functions to deliver certain coaching activities and address the key barriers. The BCW guide, previous literature review analysis, including recent systematic review results for the most effective BCTs associated with a clinically significant reduction in HbA1C were all used to help in selecting BCTs. Some BCTs were selected as per the competencies of health coaching as recommended by ICF and the intervention-underpinning theories. As advocated by [39], the BCTs have been selected and matched to the underpinning theories used in this study motivational interviewing (MI) and the transtheoretical model (TTM). The BCTs used that match the MI techniques are presented (see Supplementary File S2) [40]. In addition, other BCTs used in the health coaching intervention that match the TTM are presented (see Supplementary File S3) [41]. All the identified BCTs were assessed according to APEASE criteria to ensure the possibility of using them in the intervention context. Table 2 maps the BCTs selected and their link to the key barriers and intervention functions.

For peer review only

Int. J. Environ. Res. Public Health **2022**, *19*, 15089

Table 1. The BCTs selected and its link to the key barriers and intervention functions.

COM-B		TDF	Barrier	Intervention Function	BCTs
Capability	Psychological	Knowledge	Poor T2DM knowledge affect self-control<br>Poor nutrition knowledge (what the association between diabetes and diet, type of healthy food)<br>Poor knowledge about PA (how to engage in PA, how much time spend on PA, PA intensity, PA types, underestimate the role of PA	Education	5.1 Information about health consequences
	Physical	Physical skills	Lack of energy to do PA<br>Lack or limited skills of physical skills to do exercise	Enablement<br>Training	Enablement:<br>6.1 Demonstration of the behaviour<br>1.5 Review behaviour goal (s)<br>1.7 Review outcome goal (s)<br>2.3 Self-monitoring of behaviour<br>12.1 Restructuring the physical environment<br>4.1 Instruction on how to perform a behaviour′<br>3.1 Social support (unspecified)<br>Training:<br>8.1 Behavioural practice/rehearsal<br>8.3 Habit Formation<br>8.4 Habit reversal<br>8.7 Graded tasks<br>15.4 Self-talk<br>10.9 Self-reward<br>2.2 Feedback on behaviour<br>2.3 Self-monitoring of behaviour
Opportunity	Social	Social influences	Social norms and habits: overuse of high calories food intake; carbohydrates and fats, eating together (collectives), and social courtesy to eat unhealthy diet	Enablement	Enablement:<br>1.3 Goal setting (outcome)<br>1.1 Goal setting (behaviour)<br>3.1 Social support (unspecified)<br>1.4 Action planning<br>1.2 Problem-solving<br>2.3 Self-monitoring of behaviour<br>8.7 Graded tasks<br>5.5 Anticipated regret<br>12.1 Restructuring the physical environment<br>12.2 Restructuring the social environment

For peer review only

Table 1. *Cont.*

COM-B		TDF	Barrier	Intervention Function	BCTs
	Physical	Environmental context and resources	Lack of time<br>Lack of resources (environmental, appropriate climate and financial ability)<br>Lack of access to do activity<br>Overuse of cars for transportations	Restriction<br>Environmental restructuring<br>Enablement<br>Modelling	Enablement:<br>1.3 Goal setting (outcome)<br>1.1 Goal setting (Behaviour)<br>3.1 Social support (unspecified)<br>1.4 Action planning<br>1.2 Problem-solving<br>2.3 Self-monitoring of behaviour<br>9.2 Pros and cons<br>9.3 Comparative imagining of future outcomes<br>8.7 Graded tasks<br>1.9 Commitment<br>13.2 Framing/reframing<br>5.5 Anticipated regret<br>12.5 Adding objects to the environment<br>Modelling:<br>6.1 Demonstration of the behaviour<br>Environmental restructuring:<br>12.1 Restructuring the physical environment<br>7.1 Prompts/Cues<br>Restriction:<br>Use rules to reduce opportunity to engage in unwanted behaviour
Motivation	Reflective	Beliefs about own capability	Lack of willpower and self-confidence to do PA and maintain healthy diet	Persuasion<br>Education<br>Enablement	Persuasion:<br>15.1 Verbal persuasion about capability<br>15.2 Mental rehearsal of successful performance<br>9.1 Credible source<br>2.2 Feedback on behaviour<br>13.2 Framing/reframing<br>15.3 Focus on past success<br>Education:<br>5.1 Information about health consequences<br>5.3 Information about social and environmental consequences<br>Enablement:<br>1.9 Commitment<br>5.5 Anticipated regret
		Beliefsconsequences	Fear from consequences of PA (fear of injury and disease future complications)	Education	5.1 Information about health consequences
		Social role and identity	Struggle to change social identity associated with culture diet<br>Struggle to accept the fact of living with diabetes	Education<br>Persuasion	Education:<br>5.1 Information about health consequences<br>Persuasion:<br>13.5 Identity associated with changed behaviour

For peer review only

Table 2. Health coaching intervention detailed protocol.

Phase #	Session Content	Session Goals	Intervention Function
1	• Session #1, the patient's assessment form and consent supposed to be completed<br>• General introduction about the health coaching intervention<br>• Outline the intervention structure and content<br>• Discuss the coach's roles and the expectations from the participant (being completely clear with the client about the health coaching)<br>• Creating an alliance (Establish Trust)<br>• Learn from a patient (disease history, obstacles, priorities, strengths, goals, etc.)<br>• Help patient to create wellness vision<br>• Assess the readiness of patient's stage in relation to change health behaviour (the transtheoretical model)<br>• Introduce the importance of having a healthy diet<br>• Introduce the importance of increasing physical activity<br>• Increase awareness of adopting a healthy lifestyle in relation to controlling diabetes<br>• Identify 3-month general behavioural goals and biweekly goals<br>• Explore resources needed to help achieve desirable behaviour<br>• Commitment	• Identifying the patient's current position in the overall health status (via the transtheoretical model)<br>• Build up a relationship (as an ally) between patient and coach<br>• Encourage open discussion<br>• Make sure the patient understands what health coaching is<br>• Allow the patient to develop a foundational conception of goal setting and action planning<br>• The patient creates (SMART) measurable, action-based, realistic and timely achievable goals<br>• Have better control of carbohydrate and fat intake<br>• Gradually increase physical activity<br>• Gradually increase achievable tasks until the intended behaviour is achieved	• Education<br>• Enablement<br>• Training<br>• Restriction<br>• Environmental restructuring

For peer review only

Int. J. Environ. Res. Public Health **2022**, *19*, 15089

Table 2. *Cont.*

Phase #	Session Content	Session Goals	Intervention Function
2	• Phase #2 (session # 2), (this phase will be used again in sessions # 4 and 5)<br>• Check ongoing progress<br>• Understand patient's state (use reflections)<br>• Ask patient to share views (so far) of good things that occurred and experiences from last session<br>• Use positive reflections about patient's strengths, passion or emotions<br>• Ask patient to assess the previously selected short goals and accomplishments<br>• Use reflections to understand potential barriers prevent patient from achieving past goals<br>• Identify specific strategies that they may use to overcome the obstacles<br>• Explore what patient learned from past experience<br>• Ask and discuss with patient next short goals<br>• Share feedback on patient's progression<br>• Ask patient to connect current accomplishments to the general 3-months goals<br>• Review the general goals to see if patient want to revise them (to be more realistic and achievable)<br>• Affirm the patient's choices, strengths, and capability<br>• Use techniques such as reflective listening to address ambivalence and respond to the patient's resistance (motivational interviewing)	• Assessment of progression<br>• Review goal setting (behaviour)<br>• Review behaviour goals to examine a patient's performance progression toward the agreed goals<br>• Enable patient to develop problem-solving skills<br>• Enable patient to create action plan<br>• Prompt the participant to generate ideas and strategies to overcome barriers (problem-solving)<br>• Allow patients to monitor their behaviours (know the changes so far)<br>• Keep patient motivated (no matter the accomplishments)	• Persuasion<br>• Education<br>• Enablement<br>• Training<br>• Restriction<br>• Environmental restructuring

For peer review only

Table 2. *Cont.*

Phase #	Session Content	Session Goals	Intervention Function
3	• Middle phase (session # 3), the coach continues to observe the patient and give feedback to help them move forward in achieving their goals through bi-weekly SMART goal setting.<br>• Patient continues to identify strategies to address existing obstacles and enhance their self-ability<br>• If goals are not achieved, the barriers will be identified, action plan will be taken to address these obstacles and modified goals will be created<br>• The coach will assess the patient's self-efficacy by scoring goals to measure the participant's confidence in achieving their goals<br>• The coach continues using the skills needed to explore ambivalence and discrepancies between the participant's plans and their actual behaviour (Rollnick et al., 2005)<br>• Affirmations and appreciative inquiry will be used to appreciate progression and improve patients' self-confidence	• Assessment of current behavioural change<br>• Review goal setting (behaviour)<br>• Review behaviour goals to examine a patient's performance progression toward the agreed goals<br>• Review all previous goals and reassess goal progress<br>• Enable the participant to assess their progress<br>• Prompt the participant to analyse factors influencing their behaviour<br>• Participant's commitment to affirm to review and change behaviour	• Persuasion<br>• Education<br>• Enablement<br>• Training
4	• (session # 6)<br>• Conclude the coaching relationship<br>• Determine where the patient is in terms of their goals<br>• How the coach can best guide the client, and whether coaching is what will best benefit the client<br>• Participants' assessment of the intervention, general satisfaction of the participant with the process<br>• Appreciate the patient's engagement in the intervention<br>• Explore the patients' experience and how future coaching intervention would best support T2DM	• Allow patient to explore the difference at the endpoint<br>• Learn for participants' experience<br>• Findings from the feasibility study will be used to justify an expansion of the study (full-scale) or refined for better outcomes so that we can carry out a large RCT on the efficacy of this intervention	• Persuasion<br>• Education

For peer review only

In summary, a total of 29 BCTs will be used to carry out the content of health coaching, including the activities to achieve the desired outcomes. Out of 29 BCTs, 16 BCTs were identified from the intervention underpinning theories (IM and TTM) [40,41]. In addition, four BCTs were identified and showed effectiveness in reducing the levels of HbA1c [42]. Additional nine BCTs were included that match the provided skills of health coaching.

2.8.2. Intervention Procedure

Three trained and qualified health coaches will deliver the coaching sessions. They all will receive training on the intervention curriculum (see Table 2). Additionally, they will be monitored by the researchers throughout the period of delivery of the intervention. The intervention consists of 6 sessions that will be delivered biweekly via face-to-face meetings and telephone coaching over a 3-month period. Intervention group participants will be contacted fortnightly and coached using a combination of different methods; in person, by phone, and by smartphone (e.g., secure messaging such as WhatsApp) while the control group participants only receive the usual care for participants with T2DM with includes general lifestyle advice. Face-to-face coaching is only conducted at baseline and in the third month of the intervention for 30–45 min coaching sessions. In addition, 10–15 min telephone coaching will be delivered for the other four sessions (see Figure 1).

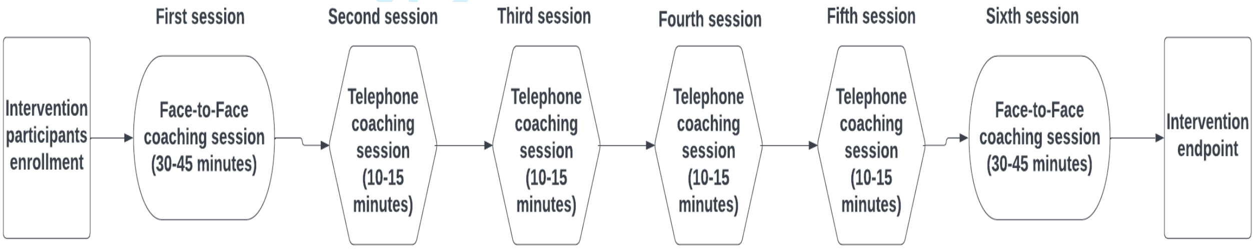

Figure 1. Health coaching intervention sessions biweekly.

The health coach works as an ally to enable participants to effectively engage in their self-care needs and address potential self-management barriers. They will be provided with different tools, including a patients' progress tracker, coach notes log and a coaching log to help them run each coaching session. Participants will be given the opportunity to learn and practice significant skills to help them reach target goals through action plans they set for themselves for better self-care [43]. Table 2 illustrates the operationalised protocol and phases of session delivery

2.8.3. Usual Care (Control Group)

Control group participants will receive only the usual care. Standard care includes providing written information on diabetes and brochures for raising awareness, including the benefits of modifying their health behaviours. Generally, T2DM patients have regularly scheduled visits to check diabetes management by endocrine specialists. The visit's main purpose is to check whether the patient's medications need to be replaced, increased, or continue with the same prescription.

2.9. Measures

Different tools and measures will be used to assess the primary and secondary study's outcomes as presented in below sections. To reduce the risk of bias, an independent interviewer will be trained and supervised by the researchers to undertake the role of collecting some data, including semi-structured interviews and providing questionnaires. The interview guide will be developed by the researchers and used by the interviewer to guide the interviews. The interviewer will remain blind to the intervention details, process, and activities. Control group participants will be requested to complete pre- and post-intervention measures and questionnaires.

For peer review only - http://bmjopen.bmj.com/site/about/guidelines.xhtml

2.9.1. Primary Outcome Measures

Feasibility

The primary outcome will assess the feasibility, including recruitment rates of the study and retention, and acceptability of the intervention by participants. During this step, the researchers seek to measure the possibility of recruiting sufficient participants within a specific period and the number of recruited participants who completed the study. Participants' responses and expressions of their engagement interests will be recorded, including reasons for interest, reasons for engagement, and reasons for not interest. The number and percentage of interested eligible, complete, and drop-out participants will be recorded to explain the recruitment rates and retention. In addition, the researchers will undertake a qualitative process evaluation to investigate participants' and their physicians' experiences and feedback on the intervention, including assessment of the most appropriate advertising method for the intervention. Different questionnaires will be used to provide further information. In addition, the health coach also will report on his experience with participants.

Acceptability

Qualitative and quantitative data will be used to assess to what extent the intervention and its implementation are acceptable to the patients. The purpose of assessing the implementation process is to identify any potential problem with the methodology used to deliver the intervention. The acceptability of the implementation process, including data collection procedures, will be assessed by conducting post-intervention semi-structured qualitative interviews, informal meetings and focus groups. Interview guidance will be used to direct the conversations and enable the researchers to explore more details about how the participants find and describe their intervention experience. A questionnaire will be used to quantify the participants' self-reported satisfaction (Likert-scale Satisfaction Questionnaire, 14-items). Healthcare providers and participants' opinions are important to help adapt the intervention in terms of ensuring that it corresponds with and is acceptable for the Saudi cultural context.

2.9.2. Secondary Outcome Measures

The secondary outcomes (preliminary efficacy) comprise anthropometric, clinical, and psychological variables selected as of the standard measures for monitoring and prevention of complications in diabetes [44]. These variables will be assessed twice at the intervention, at baseline and after 3 months for HbA1C, blood pressure, body mass index (BMI), weight and waist circumference. Change in outcomes post-intervention will help to determine if the intervention is feasible, acceptable, and effective. Table 3 provides the timeline for the assessment of the variables.

2.9.3. Demographic Information

Demographic information will be used to explain the nature of the recruited participants in the intervention. It will be collected at a single time during the intervention baseline.

2.9.4. Fidelity Assessment

Qualitative data, including interviews, focus groups, meetings, and coaching sessions will be audio recorded, transcribed, and translated into English. Thematic analysis will be used to analyse qualitative data. Multiple audio recorders will be used to ensure high reliability, and they will be transcript to text by two independent native Arabic speakers. Final coding will be used after reaching an agreement between the independent transcribers. Translation to English will be the next step completed by the researchers and an additional check of meaning by an independent native Saudi Arabian Arabic speaker.

For peer review only

Table 3. Timeline for the assessment of the intervention measurements.

Study Timeline				
**Pre-Study Allocation**			**Post-Study Allocation**	
**Activity**	**Enrolment**	**Allocation**	**Baseline**	**Endpoint**
Intervention advertising				
Screening eligibility				
Informed consent				
Baseline measures				
Randomisation				
Allocation				
Start of the Study:				
Intervention group				
Control group				
Assessments:				
Demographic				
BMI				
Weight				
Blood pressure				
Waist circumference				
HbA1c				
Feasibility Questionnaire				
Summary of Diabetes Self Care Activity (SDSCA), 12-items				
Self-efficacy Scale for Diabetes, 8-items				
Acceptibity Questionnaire				
Likert Scale Satisfaction Questionnaire, 14-items				

2.9.5. Predetermined Progression Criteria to Proceed to a Larger Trial

Before the feasibility of RCT started, the research team developed progression criteria. The progression criteria will help to decide on carrying out a full scale based on the findings generated from the feasibility study regarding recruitment, retention, adherence, and overall acceptability [45]. These criteria establish the benchmarks for assessing the trial's feasibility and determining whether undertake a further larger-scale study. The predetermined progression criteria are presented in Table 4.

For peer review only

Table 4. Health coaching predetermined criteria.

Criteria	Predetermined Cut-Offs
Screening prospective participants	• If 60–80% or more of those eligible to participate in the study consented, this supports conducting a large RCT scale trial, but if percentage is less than 50%, there is no significance to move forward.
Recruitment rate	• If the recruitment rate of people who were eligible and consented was ≥80% this would support conducting a large RCT trial.<br>• If the rate was between 70–65%, this needs further discussion to explore the reasons and whether, if they can be modified, then the trial may progress with cautions.<br>• If the rate was ≤65%, there is no significance to moving forward.
Retention rate at 3-months	• If the rate was ≥83%, this supports conducting a large RCT scale trial<br>• If the rate was less than 83%, there is no significance to moving forward.
Intervention adherence	• If the adherence rate was ≥84% of the intervention (≥5 out of the 6 coaching sessions)<br>• If the rate was between 84–67% (≥5.4 out of the 6 coaching sessions), this needs further discussion to explore the reasons and whether, if they can be modified, then the trial may progress with cautions.<br>• If the adherence rate was less than 67%, there is no significance to moving forward.

2.9.6. Data Management

Both textual and audio data will be generated and collected using different research methods. The textual data are (i) clinical observations and field notes; (ii) notes collected from formal and informal meetings with healthcare staff; and (iii) field notes from the health coaching sessions. The audio data will consist of (i) recordings of some formal and informal meetings with healthcare professionals; (ii) recordings from health coaching sessions; and (iii) recordings from focus groups. All recordings will be immediately downloaded on separate devices.

The majority of raw quantitative data will be stored as tabular data copies in Microsoft Excel format to allow for statistical analysis via (SPSS). Thematic analysis will be used to analyse qualitative data. Multiple audio recorders will be used to ensure high reliability, and they will be transcript to text by independent native Saudi speakers. Qualitative data will be stored temporarily on multiple hard drives, e.g., USB and Google Drive, and manual copies, e.g., handbooks and logbooks. Separate files will be used for each type of qualitative data for organising purposes. For ensuring the long-term usability of data, they will be stored in a secure place within (KFMC governmental institution) for confidentiality purposes and participants' privacy. Additional space storage will be requested from the University of Sheffield to ensure that enough multiple copies of data are there in case of any potential risk, for example, losing some data due to storage abilities or difficulty in accessing any other technical issues. I will continuously make backups of the files on external hard devices and in a safe and secure place through (KFMC and the University of Sheffield).

Qualitative data, including interviews, focus groups, meetings, and coaching sessions will be recorded on electronic recorders, transcribed, and translated. There will be interview guidance used to direct the conversations and enable the researchers to obtain the

For peer review only

intervention's information. All participants will be given a unique reference number used when referring directly to their responses. Quantitative data will be generated and collected by the researchers with support from the centre's health professionals, including HbA1c, BMI, blood pressure, weight and waist circumference. All data will be encrypted using a high-level encryption method to protect collected data (using FileVault on Mac) and the University Data Centre.

The researchers will be monitoring the quality of data from the beginning until the intervention endpoint. Data will be organised based on its type (e.g., interviews, coaching sessions, etc.), in separate files within one folder for easy access to each type at any time. Multiple copies of the folder will be backed up and stored throughout the period of data collection. Backups with up-to-date data will be completed continuously to keep data safe and updated. Qualitative data will be recorded to avoid accidentally missing information. This would allow for assessing the consistency and quality of the data collected. Vitro measures will be taken by a health practitioner (e.g., laboratory specialist) from KFMC in Riyadh. Multiple samples will be taken to ensure the quality of results by comparison.

3. Data Analysis

3.1. Quantitative Data

The feasibility and acceptability of the study's findings will be evaluated in relation to the quantitative data using descriptive analysis. The descriptive analysis covers different aspects as the following:

- Screening, recruitment process, retention and adherence (coaching sessions) rates will be calculated and presented as proportions
- Length of time to recruit the target sample
- Duration of time needed to complete the assessments
- Percentage of completed interventions sessions
- Average time needed to complete each session
- Description of participants interaction during coaching sessions (frequent BCTs used, interactions with coaches)
- Acceptability and suitability of intervention through participants' perceptions of, and experiences with, the health coaching intervention (Satisfaction Questionnaire)
- Additionally, other outcome measures such as diabetes self-management and patient self-efficacy will be evaluated at baseline and endpoint to investigate changes in participants' behaviours compared to another group
- Preliminary effects of the intervention
- Regarding the evaluation of the preliminary efficacy of the intervention, secondary variables will be described by means and S.Ds, investigated pre- and post-intervention to measure changes in the outcomes; glycosylated haemoglobin (HbA1C), blood pressure, body mass index (BMI), waist circumference, weight, patients' self-efficacy, and diabetes self-management
- Correlations will be performed between:
- The number of completed coaching sessions and A1c, BP, BMI, WC body weight at 3 months
- The number of completed coaching sessions and self-efficacy and diabetes self-management scales at 3 months

3.2. Qualitative Data

Qualitative data, including interviews, focus groups, meetings, and coaching sessions will be audio recorded then transcribed, and translated into English. Thematic analysis will be used to analyse qualitative data. Multiple audio recorders will be used to ensure high reliability, and they will be transcript to text by independent native Saudi Arabian speakers. The qualitative analysis includes the following:

All interviews and focus groups are recorded and transcribed

For peer review only

- A total of 10% of transcription will be translated from Arabic to English, back-translated for accuracy and validity purposes, and checked by a professional native speaker.

To prevent meaning from being lost in translation, to improve the validity of the data, which might be compromised if it is translated, and to expedite the process, the analysis will be carried out in the original language [46].

- The analysis will be conducted by using:
    - o A reflexive thematic analysis [47];
    - o From a Pragmatic philosophical standpoint [48];
    - o Thematic analysis will be manually conducted using NVivo software.

Next, all interactions with the health coaches through the coaching sessions will be mapped to the BCW and the BCTTv1 using NVivo for coding used BCTs. The final step is integrating QUALI and QUANT data and analysing them as they occurred concurrently simultaneously per the thesis process diagram (convergent design).

4. Discussion

Changing lifestyle is widely recognised as the key to supporting people with T2DM to adopt healthy behaviours to control their condition. An individualised, self-management approach for non-communicable diseases such as T2DM is now urged to a more prominent degree than at any other time [49,50]. Several studies have reported the positive effects of lifestyle intervention programmes among people at high-risk, with a 58% decrease in the incidence of T2DM [51,52]. Utilising a lifestyle intervention aims at reducing the probable risks of chronic diseases in addition to decreasing the occurrence of such conditions, if already existent, as part of a management plan. Adopting a healthy diet accompanied by physical activity is likely to decrease the risk of T2DM by 45% regardless of genetic risk [11]. Different studies have found that chronic diseases such as T2DM can be effectively addressed using health coaching programmes, which aim to promote healthy behaviours [53]. Moreover, studies have demonstrated the effectiveness of health coaching with a variety of chronic illnesses, including T2DM [21].

Conducting the feasibility study will help inform the research development process by identifying key uncertainties in the study before a future full-scale intervention is carried out. It is hoped that the findings of the present study will contribute to informing the research field of health coaching on T2DM and to ensure the intervention achieves a good fit with a new context; namely, Saudi patients with T2DM. Since health coaching as a new approach is now included in the Saudi Ministry of Health's (MOH) future plans, the study findings can directly inform future policy developments in SA. Additionally, it is hoped that the present study will make a significant contribution to the literature as it represents the first study that has been developed based on the use of specific "active ingredients" (BCTs), which directly match the intervention's underpinning theories. Consequently, health coaching intervention designers may benefit from the study's findings by allowing them to interpret and identify effective BCTs to facilitate future replication. Finally, the study's findings could lead to the increased uptake of other behaviour change interventions in the Saudi Arabian context.

Supplementary Materials: The following supporting information can be downloaded at: https://www.mdpi.com/article/10.3390/ijerph192215089/s1, File S1: CONSORT 2010 checklist of information to include when reporting a pilot or feasibility trial, File S2: BCTs matching the motivational interviewing, partly adopted from Hardcastle et al. (2017) [40], File S3: BCTs matching the stage of change model, partly adapted from Moore et al. (2015) [41].

Author Contributions: Conceptualization, A.N.A.; methodology, A.N.A.; software, A.N.A.; validation, A.N.A.; resources, A.N.A.; data curation, A.N.A.; writing—original draft preparation, A.N.A.; writing—review and editing, A.N.A., E.G. and S.J.C.; visualization, A.N.A.; supervision, E.G. and S.J.C.; funding acquisition, A.N.A. All authors have read and agreed to the published version of the manuscript.

Funding: This study received no external funding.

For peer review only - http://bmjopen.bmj.com/site/about/guidelines.xhtml

Institutional Review Board Statement: Ethical approval was obtained by the University of Sheffield and the Institutional Review Board (IRB) committee form ethical approval at (KFMC) (IRB Log Number: 21-062E).

Informed Consent Statement: Prior to data collection, written informed consent from participants will be obtained.

Data Availability Statement: All data generated or analysed during this study will be included and are available upon reasonable request from the corresponding author.

Acknowledgments: We would like to thank the University of Sheffield Institutional Open Access Fund for its support. This paper will be published under a Creative Commons Attribution (CC BY) licence.

Conflicts of Interest: The authors declare no conflict of interest.

References

1. *Global Report on Diabetes WHO Library Cataloguing-in-Publication Data Global Report on Diabetes*; WHO Press: Geneve, Switzerland, 2016.
2. Complications of Diabetes. Available online: https://www.diabetes.org.uk/guide-to-diabetes/complications (accessed on 4 April 2022).
3. IDF Diabetes Atlas Tenth Edition 2021. Available online: https://www.idf.org/aboutdiabetes/what-is-diabetes/facts-figures.html (accessed on 4 April 2022).
4. Diabetes. Available online: https://www.who.int/news-room/fact-sheets/detail/diabetes (accessed on 13 April 2022).
5. Al Slamah, T.; Nicholl, B.I.; Alslail, F.Y.; Melville, C.A. Self-management of type 2 diabetes in gulf cooperation council countries: A systematic review. *PLoS ONE* **2017**, *12*, e0189160. [CrossRef] [PubMed]
6. Alqurashi, K.A.; Aljabri, K.S.; Bokhari, S.A. Prevalence of diabetes mellitus in a Saudi community. *Ann. Saudi Med.* **2011**, *31*, 19–23. [CrossRef] [PubMed]
7. Liu, J.; Ren, Z.H.; Qiang, H.; Wu, J.; Shen, M.; Zhang, L.; Lyu, J. Trends in the incidence of diabetes mellitus: Results from the Global Burden of Disease Study 2017 and implications for diabetes mellitus prevention. *BMC Public Health* **2020**, *20*, 1415. [CrossRef]
8. Al-Rubeaan, K. National surveillance for type 1, type 2 diabetes and prediabetes among children and adolescents: A population-based study (SAUDI-DM). *J. Epidemiol. Community Health* **2015**, *69*, 1045–1051. [CrossRef] [PubMed]
9. Alanazi, F.K.; Alotaibi, J.S.; Paliadelis, P.; Alqarawi, N.; Alsharari, A.; Albagawi, B. Knowledge and awareness of diabetes mellitus and its risk factors in saudi arabia. *Saudi Med. J.* **2018**, *39*, 981–989. [CrossRef] [PubMed]
10. Azab, A.S. Glycemic control among diabetic patients. *Saudi Med. J.* **2001**, *22*, 407–409. [PubMed]
11. Midhet, F.M.; Al-Mohaimeed, A.A.; Sharaf, F.K. Lifestyle related risk factors of type 2 diabetes mellitus in Saudi Arabia. *Saudi Med. J.* **2010**, *31*, 768–774.
12. Gillies, C.L.; Abrams, K.R.; Lambert, P.C.; Cooper, N.J.; Sutton, A.J.; Hsu, R.T.; Khunti, K. Pharmacological and lifestyle interventions to prevent or delay type 2 diabetes in people with impaired glucose tolerance: Systematic review and meta-analysis. *BMJ* **2007**, *334*, 299. [CrossRef]
13. Wolever, R.Q.; Jordan, M.; Lawson, K.; Moore, M. Advancing a new evidence-based professional in health care: Job task analysis for health and wellness coaches. *BMC Health Serv. Res.* **2016**, *16*, 205. [CrossRef]
14. Wroth, S.W. Health Coaching Bridges Gaps in Patient Care. *Altern. Complement. Ther.* **2015**, *21*, 157–159. [CrossRef]
15. Newman, S.; Steed, L.; Mulligan, K. Self-management interventions for chronic illness. *Lancet* **2004**, *364*, 1523–1537. [CrossRef]
16. Aikaterini, T.; Papazafiropoulou, A.K.; Melidonis, A. Type 2 diabetes and quality of life. *World J. Diabetes* **2017**, *8*, 120–129.
17. Alabdulbaqi, D. A Cultural Adaptation of a Diabetes Self-Management Programme for Patients with Type 2 Diabetes Attending a Primary Health Care Centre in Saudi Arabia. Ph.D. Thesis, London School of Hygiene & Tropical Medicine, London, UK, April 2019.
18. Pearson, M.L.; Mattke, S.; Shaw, R.; Ridgely, M.M.S.; Wiseman, S.H. *Patient Self-Management Support Programs: An Evaluation*; Final Contract Report Agency for Healthcare Research and Quality: Rockville, MD, USA, 2007.
19. Wolever, R.Q.; Dreusicke, M.; Fikkan, J.; Hawkins, T.V.; Yeung, S.; Wakefield, J.; Duda, L.; Flowers, P.; Cook, C.; Skinner, E. Integrative Health Coaching for Patients With Type 2 Diabetes. *Diabetes Educ.* **2010**, *36*, 629–639. [CrossRef] [PubMed]
20. Wong-Rieger, D.; Rieger, F.P. Health coaching in diabetes: Empowering patients to self-manage. *Can. J. Diabetes* **2013**, *37*, 41–44. [CrossRef] [PubMed]
21. Pirbaglou, M.; Katz, J.; Motamed, M.; Pludwinski, S.; Walker, K.; Ritvo, P. Personal Health Coaching as a Type 2 Diabetes Mellitus Self-Management Strategy: A Systematic Review and Meta-Analysis of Randomized Controlled Trials. *Am. J. Health Promot.* **2018**, *32*, 1613–1626. [CrossRef]
22. Hill, B.; Richardson, B.; Skouteris, H. Do we know how to design effective health coaching interventions: A systematic review of the state of the literature. *Am. J. Health Promot.* **2015**, *29*, e158–e168. [CrossRef]
23. Michie, S.; van Stralen, M.M.; West, R. The behaviour change wheel: A new method for characterising and designing behaviour change interventions. *Implement. Sci.* **2011**, *6*, 42. [CrossRef]

For peer review only

24. Alamri, F.; Radwan, N.; Elolemy, A.; Alkhashan, H. Effectiveness of health coaching on diabetic patients: A Systematic Review and Meta-analysis. *Tradit. Med. Res.* **2019**, *4*, 314–325.
25. Davies, M.J.; Heller, S.; Skinner, T.C.; Campbell, M.J.; Carey, M.E.; Cradock, S.; Dallosso, H.M.; Daly, H.; Doherty, Y.; Eaton, S.; et al. Effectiveness of the diabetes education and self management for ongoing and newly diagnosed (DESMOND) programme for people with newly diagnosed type 2 diabetes: Cluster randomised controlled trial. *BMJ* **2008**, *336*, 491–495. [CrossRef]
26. van Bokhoven, M.A. Designing a quality improvement intervention: A systematic approach. *Qual. Saf. Health Care* **2003**, *12*, 215–220. [CrossRef]
27. Craig, P.; Dieppe, P.; Macintyre, S.; Michie, S.; Nazareth, I.; Petticrew, M. Developing and evaluating complex interventions. *BMJ* **2008**, *337*, a1655. [CrossRef]
28. Bowen, D.J.; Kreuter, M.; Spring, B.; Cofta-Woerpel, L.; Linnan, L.; Weiner, D.; Bakken, S.; Kaplan, C.P.; Squiers, L.; Fabrizio, C.; et al. How We Design Feasibility Studies. *Am. J. Prev. Med.* **2009**, *36*, 452–457. [CrossRef] [PubMed]
29. Michie, S.; Prestwich, A. Are interventions theory-based? Development of a theory coding scheme. *Health Psychol.* **2010**, *29*, 1–8. [PubMed]
30. Eldridge, S.M.; Chan, C.L.; Campbell, M.J.; Bond, C.M.; Hopewell, S.; Thabane, L.; Lancaster, G.A.; Altman, D.; Bretz, F.; Campbell, M.; et al. CONSORT 2010 statement: Extension to randomised pilot and feasibility trials. *BMJ* **2016**, *355*, i5239. [CrossRef]
31. Powell, H.; Mihalas, S.; Onwuegbuzie, A.J.; Suldo, S.; Daley, C.E. Mixed methods research in school psychology: A mixed methods investigation of trends in the literature. *Psychol. Sch.* **2008**, *45*, 291–309. [CrossRef]
32. Hariton, E.; Locascio, J.J. Randomised controlled trials—The gold standard for effectiveness research: Study design: Randomised controlled trials. *BJOG* **2018**, *125*, 1716. [CrossRef]
33. Schulz, K.F.; Altman, D.G.; Moher, D. CONSORT 2010 Statement: Updated guidelines for reporting parallel group randomised trials. *BMJ* **2010**, *340*, 698–702. [CrossRef]
34. Cocks, K.; Torgerson, D.J. Sample size calculations for pilot randomized trials: A confidence interval approach. *J. Clin. Epidemiol.* **2013**, *66*, 197–201. [CrossRef]
35. Julious, S.A. Sample size of 12 per group rule of thumb for a pilot study. *Pharm. Stat.* **2005**, *4*, 287–291. [CrossRef]
36. Atkins, L.; Francis, J.; Islam, R.; O'Connor, D.; Patey, A.; Ivers, N.; Foy, R.; Duncan, E.M.; Colquhoun, H.; Grimshaw, J.M.; et al. A guide to using the Theoretical Domains Framework of behaviour change to investigate implementation problems. *Implement. Sci.* **2017**, *12*, 77. [CrossRef]
37. Dyson, P.A.; Twenefour, D.; Breen, C.; Duncan, A.; Elvin, E.; Goff, L.; Hill, A.; Kalsi, P.; Marsland, N.; McArdle, P.; et al. Diabetes UK evidence-based nutrition guidelines for the prevention and management of diabetes. *Diabet. Med.* **2018**, *35*, 541–547. [CrossRef] [PubMed]
38. Michie, S.; Ashford, S.; Sniehotta, F.F.; Dombrowski, S.U.; Bishop, A.; French, D.P. A refined taxonomy of behaviour change techniques to help people change their physical activity and healthy eating behaviours: The CALO-RE taxonomy. *Psychol. Health* **2011**, *26*, 1479–1498. [CrossRef] [PubMed]
39. Van Vugt, M.; De Wit, M.; Cleijne, W.H.J.J.; Snoek, F.J. Use of behavioral change techniques in web-based self-management programs for type 2 diabetes patients: Systematic review. *J. Med. Internet Res.* **2013**, *15*, e279. [CrossRef] [PubMed]
40. Hardcastle, S.J.; Fortier, M.; Blake, N.; Hagger, M.S. Identifying content-based and relational techniques to change behaviour in motivational interviewing. *Health Psychol. Rev.* **2017**, *11*, 1–6. [CrossRef] [PubMed]
41. Moore, M. *Coaching Psychology Manual*, 2nd ed.; Lippincott Williams and Wilkins: St. Baltimore, MD, USA, 2015.
42. Cradock, K.A.; ÓLaighin, G.; Finucane, F.M.; Gainforth, H.L.; Quinlan, L.R.; Ginis, K.A.M. Behaviour change techniques targeting both diet and physical activity in type 2 diabetes: A systematic review and meta-analysis. *Int. J. Behav. Nutr. Phys. Act.* **2017**, *14*, 18. [CrossRef]
43. Schulman-Green, D.J.; Naik, A.D.; Bradley, E.H.; McCorkle, R.; Bogardus, S.T. Goal setting as a shared decision making strategy among clinicians and their older patients. *Patient Educ. Couns.* **2006**, *63*, 145–151. [CrossRef]
44. International Diabetes Federation: Daibetes—Facts & Figures. 2005. Available online: https://www.idf.org/aboutdiabetes/what-is-diabetes/facts-figures.html (accessed on 13 April 2022).
45. Avery, K.N.; Williamson, P.R.; Gamble, C.; Francischetto, E.O.C.; Metcalfe, C.; Davidson, P.; Williams, H.; Blazeby, J.M. Informing efficient randomised controlled trials: Exploration of challenges in developing progression criteria for internal pilot studies. *BMJ Open* **2017**, *7*, e013537. [CrossRef] [PubMed]
46. Smith, H.J.; Chen, J.; Liu, X. Language and rigour in qualitative research: Problems and principles in analyzing data collected in Mandarin. *BMC Med. Res. Methodol.* **2008**, *8*, 44. [CrossRef]
47. Braun, V.; Clarke, V. Using thematic analysis in psychology. *Qual. Res. Psychol.* **2006**, *3*, 77–101. [CrossRef]
48. Cherryholmes, C.H. Notes on pragmatism and scientific realism. *Educ. Res.* **1992**, *21*, 13–17. [CrossRef]
49. Lund, S.H.; Aspelund, T.; Kirby, P.; Russell, G.; Einarsson, S.; Palsson, O.; Stefánsson, E. Individualised risk assessment for diabetic retinopathy and optimisation of screening intervals: A scientific approach to reducing healthcare costs. *Br. J. Ophthalmol.* **2016**, *100*, 683–687. [CrossRef] [PubMed]
50. Powell, C.K.; Hill, E.G.; Clancy, D.E. The Relationship Between Health Literacy and Diabetes Knowledge and Readiness to Take Health Actions. *Diabetes Educ.* **2007**, *33*, 144–151. [CrossRef] [PubMed]

For peer review only

51. Knowler, W.C.; Barrett-Connor, E.; Fowler, S.E.; Hamman, R.F.; Lachin, J.M.; Walker, E.A.; Nathan, D.M.; Watson, P.; Mendoza, J.; Smith, K. Reduction in the incidence of type 2 diabetes with lifestyle intervention or metformin. *N. Engl. J. Med.* **2002**, *346*, 393–403. [PubMed]
52. Lindstrom, J. Sustained reduction in the incidence of type 2 diabetes by lifestyle intervention: Follow-up of the Finnish Diabetes Prevention Study. *Lancet* **2006**, *368*, 1673–1679. [CrossRef]
53. Gallé, F.; Di Onofrio, V.; Cirella, A.; Di Dio, M.; Miele, A.; Spinosa, T.; Liguori, G. Improving Self-Management of Type 2 Diabetes in Overweight and Inactive Patients Through an Educational and Motivational Intervention Addressing Diet and Physical Activity: A Prospective Study in Naples, South Italy. *Diabetes Ther.* **2017**, *8*, 875–886. [CrossRef] [PubMed]

For peer review only

Reporting checklist for quality improvement in health care.

Based on the SQUIRE guidelines.

Instructions to authors

Complete this checklist by entering the page numbers from your manuscript where readers will find each of the items listed below.

Your article may not currently address all the items on the checklist. Please modify your text to include the missing information. If you are certain that an item does not apply, please write "n/a" and provide a short explanation.

Upload your completed checklist as an extra file when you submit to a journal.

In your methods section, say that you used the SQUIREreporting guidelines, and cite them as:

Ogrinc G, Davies L, Goodman D, Batalden P, Davidoff F, Stevens D. SQUIRE 2.0 (Standards for QUality Improvement Reporting Excellence): revised publication guidelines from a detailed consensus process

		Reporting Item	Page Number
**Title**			
	#1	Indicate that the manuscript concerns an initiative to improve healthcare (broadly defined to include the quality, safety, effectiveness, patientcenteredness, timeliness, cost, efficiency, and equity of healthcare)	1
**Abstract**			
	#02a	Provide adequate information to aid in searching and indexing	1
	#02b	Summarize all key information from various sections of the text using the abstract format of the intended publication or a structured summary such as: background, local problem, methods, interventions, results, conclusions	1
**Introduction**			
Problem	#3	Nature and significance of the local problem	2

For peer review only

description			
Available knowledge	#4	Summary of what is currently known about the problem, including relevant previous studies	2
Rationale	#5	Informal or formal frameworks, models, concepts, and / or theories used to explain the problem, any reasons or assumptions that were used to develop the intervention(s), and reasons why the intervention(s) was expected to work	2
Specific aims	#6	Purpose of the project and of this report	2
**Methods**			
Context	#7	Contextual elements considered important at the outset of introducing the intervention(s)	3
Intervention(s)	#08a	Description of the intervention(s) in sufficient detail that others could reproduce it	3
Intervention(s)	#08b	Specifics of the team involved in the work	3
Study of the Intervention(s)	#09a	Approach chosen for assessing the impact of the intervention(s)	3
Study of the Intervention(s)	#09b	Approach used to establish whether the observed outcomes were due to the intervention(s)	n/a
Measures	#10a	Measures chosen for studying processes and outcomes of the intervention(s), including rationale for choosing them, their operational definitions, and their validity and reliability	5
Measures	#10b	Description of the approach to the ongoing assessment of contextual elements that contributed to the success, failure, efficiency, and cost	5
Measures	#10c	Methods employed for assessing completeness and accuracy of data	5
Analysis	#11a	Qualitative and quantitative methods used to draw inferences from the data	5
Analysis	#11b	Methods for understanding variation within the data, including the effects of time as a variable	n/a
Ethical considerations	#12	Ethical aspects of implementing and studying the intervention(s) and how they were addressed, including, but not limited to, formal ethics review and potential conflict(s) of interest	4

For peer review only

Results

	#13a	Initial steps of the intervention(s) and their evolution over time (e.g., time-line diagram, flow chart, or table), including modifications made to the intervention during the project	6
	#13b	Details of the process measures and outcome	9-26
	#13c	Contextual elements that interacted with the intervention(s)	n/a
	#13d	Observed associations between outcomes, interventions, and relevant contextual elements	27
	#13e	Unintended consequences such as unexpected benefits, problems, failures, or costs associated with the intervention(s).	n/a
	#13f	Details about missing data	n/a
**Discussion**			
Summary	#14a	Key findings, including relevance to the rationale and specific aims	27-28
Summary	#14b	Particular strengths of the project	29
Interpretation	#15a	Nature of the association between the intervention(s) and the outcomes	27-28
Interpretation	#15b	Comparison of results with findings from other publications	27-28
Interpretation	#15c	Impact of the project on people and systems	29
Interpretation	#15d	Reasons for any differences between observed and anticipated outcomes, including the influence of context	n/a
Interpretation	#15e	Costs and strategic trade-offs, including opportunity costs	n/a
Limitations	#16a	Limits to the generalizability of the work	29
Limitations	#16b	Factors that might have limited internal validity such as confounding, bias, or imprecision in the design, methods, measurement, or analysis	29
Limitations	#16c	Efforts made to minimize and adjust for limitations	n/a
Conclusion	#17a	Usefulness of the work	29
Conclusion	#17b	Sustainability	29
Conclusion	#17c	Potential for spread to other contexts	29

For peer review only

Conclusion	#17d	Implications for practice and for further study in the field	29
Conclusion	#17e	Suggested next steps	29
**Other information**			
Funding	#18	Sources of funding that supported this work. Role, if any, of the funding organization in the design, implementation, interpretation, and reporting	30

The SQUIRE 2.0 checklist is distributed under the terms of the Creative Commons Attribution License CC BY-NC 4.0. This checklist was completed on 06. August 2023 using https://www.goodreports.org/, a tool made by the EQUATOR Network in collaboration with Penelope.ai

For peer review only

Feasibility and Acceptability of Tailored Health Coaching Intervention to Improve Type 2 Diabetes Self-Management in Saudi Arabia: A Mixed-Methods Randomised Feasibility Trial

Journal:	*BMJ Open*
Manuscript ID	bmjopen-2023-078631.R1
Article Type:	Original research
Date Submitted by the Author:	03-Jan-2024
Complete List of Authors:	Almulhim, Abdullah; University of Sheffield, School of Medicine and Population Health; College of Health Sciences Saudi Electronic University<br>Alhowaish, Atheer; King Fahad Medical City<br>Madani, Alaa; King Fahad Medical City<br>AlQaddan, Munirah; King Fahad Medical City<br>Altuwalah, Abdulaziz S.; King Fahad Medical City<br>Goyder, Elizabeth; The University of Sheffield, School of Medicine and Population Health
**Primary Subject Heading**:	Public health
Secondary Subject Heading:	Public health
Keywords:	PUBLIC HEALTH, DIABETES & ENDOCRINOLOGY, Behavior

SCHOLARONE™ Manuscripts

BMJ

I, the Submitting Author has the right to grant and does grant on behalf of all authors of the Work (as defined in the below author licence), an exclusive licence and/or a non-exclusive licence for contributions from authors who are: i) UK Crown employees; ii) where BMJ has agreed a CC-BY licence shall apply, and/or iii) in accordance with the terms applicable for US Federal Government officers or employees acting as part of their official duties; on a worldwide, perpetual, irrevocable, royalty-free basis to BMJ Publishing Group Ltd ("BMJ") its licensees and where the relevant Journal is co-owned by BMJ to the co-owners of the Journal, to publish the Work in this journal and any other BMJ products and to exploit all rights, as set out in our licence.

The Submitting Author accepts and understands that any supply made under these terms is made by BMJ to the Submitting Author unless you are acting as an employee on behalf of your employer or a postgraduate student of an affiliated institution which is paying any applicable article publishing charge ("APC") for Open Access articles. Where the Submitting Author wishes to make the Work available on an Open Access basis (and intends to pay the relevant APC), the terms of reuse of such Open Access shall be governed by a Creative Commons licence – details of these licences and which Creative Commons licence will apply to this Work are set out in our licence referred to above.

Other than as permitted in any relevant BMJ Author's Self Archiving Policies, I confirm this Work has not been accepted for publication elsewhere, is not being considered for publication elsewhere and does not duplicate material already published. I confirm all authors consent to publication of this Work and authorise the granting of this licence.

For peer review only

Feasibility and Acceptability of Tailored Health Coaching Intervention to Improve Type 2 Diabetes Self-Management in Saudi Arabia: A Mixed-Methods Randomised Feasibility Trial

Abdullah N. Almulhim[1,2]*, Atheer Alhowaish[3], Alaa Madani[3], Munirah AlQaddan[3], Abdulaziz S. Altuwalah[3], Elizabeth Goyder[1]

1 School of Medicine and Population Health, University of Sheffield, Sheffield S1 4DA, UK

2 Public Health Department, College of Health Sciences, Saudi Electronic University, Riyadh 13316, Saudi Arabia

3 Health Education Administration, King Fahad Medical City, Riyadh 12231, Saudi Arabia

* Correspondence: aalmulhim@seu.edu.sa

Abstract

Background: Around a third of the population of Saudi Arabia have been diagnosed with Type 2 diabetes, a condition often requiring lifestyle changes. Personalised health coaching, a strategy developed to assist individuals in overcoming challenges to adopt healthy behaviours, has not yet been widely applied in the country.

Aims: We aim to explore the feasibility and acceptability of tailored health coaching in Saudi Arabia, in order to help those with Type 2 diabetes to more effectively manage their condition.

Methods: Using a mixed-methods approach, this research involved a randomised controlled trial with 30 Saudi adults who have Type 2 diabetes. They were randomly allocated into either the intervention or control arm for 12 weeks. The Capability, Opportunity, Motivation, and Behaviour framework was used to guide the intervention implementation along with the Behaviour Change Techniques Taxonomy version 1. The primary goal was to assess the suitability and duration of the intervention, recruitment, retention, and completion rates. The secondary outcome focused on the preliminary efficacy of the health coaching measured by the glycaemic index, blood pressure, body mass index, waist circumference, weight, patient self-efficacy, and diabetes self-management.

Results: The results showed high rates of eligibility, recruitment, and retention (a screening rate of 90%, a recruiting rate of 79%, and a retention rate of 97%). Notable improvements were observed in the health coaching group across five outcomes: HbA1c, BMI, waist circumference, patient self-efficacy, and diabetes self-care. The participants were satisfied with the intervention and requested its expansion.

Conclusion: The findings demonstrated positive outcomes, supporting the need for a larger randomised controlled trial to evaluate the efficacy of health coaching in improving diabetes self-management among individuals with Type 2 diabetes in Saudi Arabia.

For peer review only - http://bmjopen.bmj.com/site/about/guidelines.xhtml

Keywords: health behaviour change; health coaching; self-management; behaviour change techniques; type 2 diabetes

Summary Box:

Strengths and limitations of this study:

- First health coaching intervention in Saudi Arabia.
- First use of the COM-B model and BCTs taxonomy in a health coaching intervention
- Adherence to CONSORT 2010 progression criteria.
- Comprehensive impact assessment through mixed methods.
- Limited generalizability due to specific settings and demographics.

Introduction:

Type 2 Diabetes Mellitus (T2DM) is a chronic illness that is becoming more common globally and represents a significant public health concern. Lifestyle factors play a crucial role in the development of this disease, with obesity often manifesting as a symptom of other lifestyle factors such as physical inactivity (1). T2DM can lead to complications such as cardiovascular disease, kidney failure, nerve damage, and ocular and auditory problems, significantly increasing the risk of heart disease and stroke in people with diabetes (2).

Managing diabetes can be a challenging task that encompasses various aspects of life, including social, economic, and healthcare domains (3). To help T2DM patients effectively manage their condition, prioritising self-management is crucial (4). T2DM is a prevalent chronic disease in SA, with one of the highest rates in the Middle East and the seventh-highest globally (5). Unhealthy diets, inactive lifestyles, and rapid urbanisation are all contributing factors to this concerning trend (6).

Despite national efforts to control T2DM in the SA context, the disease continues to pose significant challenges (7). While awareness campaigns are being employed, there is a notable difficulty among most T2DM patients in adopting healthier diets and lifestyles (7). To address this issue, it is necessary to implement patient-centred approaches that prioritise the development of self-management skills rather than relying solely on education-based programs (8).

Health coaching, derived from various disciplines and behaviour change theories, has emerged as a client-centred approach gaining recognition in the field of health promotion. Although it has been widely explored in the literature and has shown promising results, this personalised one-on-one intervention has not yet been implemented within the healthcare systems of SA. Studies have demonstrated that health coaching, centred around patients' values, may offer additional benefits compared to traditional T2DM education programs (9). By focusing on enhancing patients' self-efficacy and essential skills such as goal-setting and problem-solving, health coaching can prove highly effective and financially viable (10). Its successful application in diverse contexts has shown it to be a valuable tool in improving diabetes self-management (11, 12).

However, the effectiveness of existing health coaching interventions exhibits inconsistency due to variations in methodology, duration, and intervention content (11). Recent systematic

For peer review only

For peer review only - http://bmjopen.bmj.com/site/about/guidelines.xhtml

reviews of randomised controlled trials employing health coaching have reported mixed results, with some studies suggesting its effectiveness while others claim it to be ineffective (11, 12). There is no agreed-upon health coaching intervention model, which creates uncertainty regarding the most effective strategies to adopt, particularly in the SA context. The inconsistent findings across studies may be due to the lack of agreement on the active ingredients and content of successful health coaching interventions (13). Additionally, cultural differences between SA and Western countries highlight the need for tailored interventions to ensure that proposed health coaching models are appropriate and relevant (14).

This feasibility study aims to assess the usability and acceptability of a health coaching intervention for T2DM self-management in SA, with the ultimate goal of reducing HbA1c levels and informing a future large-scale RCT.

Materials and Methods

This research was conducted in accordance with the Consolidated Standards of Reporting Trials (CONSORT) extension guidelines specific to pilot and feasibility randomised controlled trials (RCTs) (15). A comprehensive protocol for this study has already been published (16).

Design

This is a randomised two-arm feasibility trial evaluating a 3-month intervention. The study adopted a mixed-methods approach, enabling the collection of both quantitative and qualitative data. An RCT design to address both evaluate the feasibility of RCT methods for recruitment, deliver and data collection as well as a valid comparison of health coaching's effectiveness between the groups (17). Integral to our approach was the implementation of the Behaviour Change Wheel (BCW) framework, used in adapting the health coaching intervention for the SA context. The BCW informed our intervention design in several keyways: Firstly, by identifying suitable intervention functions from the Capability, Opportunity, Motivation-Behaviour (COM-B) model, we tailored the health coaching to the specific needs and barriers of our target population. Secondly, we utilized the Behaviour Change Technique Taxonomy version 1 (BCTTv1) to code the active ingredients of the intervention, mapping these to the BCW framework for alignment with established behaviour change principles (18). Furthermore, a behavioural analysis, guided by the Theoretical Domains Framework (TDF) functions and behavioural change techniques (BCTs), was conducted to address pre-identified barriers (see Supplemental Table 1). This analysis led to the selection of 36 relevant BCTs, which were mapped to directly target the behavioural barriers in adults with poorly managed T2DM, our intervention's target population (see Supplemental Table 2). By integrating the BCW, we were able to define the problem behaviours and target behaviours for intervention, ensuring a comprehensive and theory-driven approach as advised by BCW guidance (19). Further information on the intervention can be found in a prior publication (16).

Patient and public involvement

Patients and the public were not involved during the process of this research.

Intervention Development

For peer review only - http://bmjopen.bmj.com/site/about/guidelines.xhtml

There have been various identified target behaviours that would result in the adoption of a healthy lifestyle and diet as recommended by UK lifestyle guidelines.(20) As of now, there are no evidence-based guidelines in SA for T2DM to adopt a healthy lifestyle and diet. Therefore, we have referred to the UK's guidelines on lifestyle as they share similarities in their healthcare systems. (20, 21) These guidelines have been used to determine the most suitable behaviour targets for the Saudi context. We consulted healthcare professionals in the intervention setting, including a dietician, to establish these targets. Through collaborative discussions, we assessed and prioritised behaviours based on their potential impact, the feasibility of measurement, and achievability. As a result of this process, we have proposed four behaviour targets. (18)

The four general behaviours targets of the intervention were: (20)

- Decrease carbohydrate intake for each meal
- Use unsaturated fats as possible (avoid saturated fats)
- Do exercise for 30 min five days on a weekly basis
- Monitor waist circumference, and maintain it below (80 cm for women and 94 cm for men)

The aim was to gradually reduce carbohydrate intake while ensuring that participants met their nutritional requirements. The specific target amounts were communicated to participants during individualized dietary counselling sessions with health coaches, and participants were provided with guidance and meal plans to help them achieve and maintain the desired carbohydrate intake levels. The health coaching intervention used these targets as the benchmarks to track the progress and changes that the study population would make over three months.

We utilised diverse instruments, including questionnaires, interviews, focus groups, and clinical measures, to assess the intervention's feasibility, acceptability, and preliminary impact at two points: baseline and three months post-intervention. Ethical clearance for the study was granted by the University of Sheffield and the KFMC's Institutional Review Board (IRB Log Number: 21-062E).

Participant Recruitment

Inclusion criteria were: aged over 18, Haemoglobin A1c level of 7.0% or higher, able to read and understand Arabic, have access to a personal mobile phone, be willing to complete the intervention period, be willing to stay in Riyadh (the capital city of Saudi Arabia), and be able to read, understand, and sign the informed consent form.

The recruitment process involved advertising the intervention through multiple channels, including posters, brochures, social media, and healthcare provider referrals. Interested and eligible individuals met the research team for a brief study overview and questionnaire completion, providing demographic data and baseline laboratory reports.

For peer review only - http://bmjopen.bmj.com/site/about/guidelines.xhtml

Participants were randomly assigned to one of two groups in a 1:1 ratio by an independent individual using a computer-generated random numbers system (SPSS), ensuring equal allocation chances.

Intervention Delivery

The health coaching intervention was completed in 12 weeks. A detailed intervention curriculum was used in delivering the intervention activities toward achieving the four target behaviours (see Supplemental Table 3).

Three health coaches, AA, AM, and MA, who were qualified through the Saudi Ministry of Health, conducted the intervention sessions. They received training in biweekly face to face (F2F) meetings over six weeks on the intervention curriculum. A workbook was provided as a guide to help them deliver coaching sessions as planned. A detailed explanation has been published elsewhere (16). It is important to note that participants did not directly receive or review the intervention curriculum. Our approach involved ensuring uniformity in intervention procedures by providing health coaches with the curriculum. Each coach was asked to document the BCTs they use in each session. Different BCTs were reported in each session. Each participant was assigned to one of three coaches at the baseline. Each participant received six sessions in total, which were delivered biweekly via video meetings and telephone coaching over three months. Due to the COVID-19 pandemic, the first and last meetings were conducted via video call instead of face-to-face as planned, while the rest were via telephone. Participants were encouraged to contact their coaches via WhatsApp between sessions if they had any related concerns. Eighty-four sessions were undertaken for all participants in the intervention group.

Usual Care (Control Group)

The participants in the control group were provided with the standard care, which involved receiving written materials and brochures on diabetes to increase awareness and promote the advantages of making changes to their health behaviours. Typically, individuals with T2DM have regular appointments with endocrine specialists to monitor their diabetes management. During these visits, the primary objective is to assess if any adjustments are necessary regarding the patient's medication, such as replacement, dosage increase, or maintaining the current prescription.

Data Collection

Baseline data collection included: demographic information, HbA1c, blood pressure, BMI, weight, and waist circumference measurements. Subsequently, we asked participants to complete two electronic questionnaires: the Diabetes Self-Care Activities (SDSCA) scale and the Self-efficacy Scale for Diabetes. The post-intervention data collection replicated the initial session, with repeat measurements of HbA1c, blood pressure, Body mass index (BMI), weight, and waist circumference. Participants were also required to fill out the two initial questionnaires again, along with a Satisfaction Questionnaire designed specifically for the intervention group.

For peer review only - http://bmjopen.bmj.com/site/about/guidelines.xhtml

Reach and Retention

As per the study protocol (16), this study aimed to recruit a minimum of 9% (n = 30) of the required sample size for conducting a complete trial (22), Each group was expected to have a minimum of 12 participants to ensure meaningful data interpretation for informing a future definitive randomized controlled trial (23). A total of 24 participants in each group would be required to detect a meaningful clinical difference in HbA1c. The retention rate was evaluated based on the predefined progression criteria described elsewhere (16), with successful retention defined as achieving an 83% retention rate at the intervention endpoint.

Fidelity Assessment

All coaching sessions audio recorded and transcribed. An Arabic native speaker reviewed the entire transcription for accuracy. To ensure consistency and validity, a 10% sample of the transcription (approximately 50 minutes) was translated from Arabic to English, backtranslated, and then examined by a peer reviewer. The data, including coaching sessions, time spent in each session, semi-structured interviews, focus groups, and questionnaires, was meticulously stored and managed for quality control. Specifically, all data were entered and stored in Microsoft Excel twice, independently by two research team members. This double-entry process served as a precautionary measure to minimize data entry errors and enhance the accuracy and reliability of the data collected throughout the trial. Adherence to the intervention was defined by the number of participants who completed 5 out of six coaching sessions ≥ 84%.

Feasibility

Evaluation of feasibility included: participant eligibility and recruitment, retention, data collection adherence, and adherence to the intervention. We recorded participants' views about their engagement, capturing their reasons for interest, active participation, and lack of interest. We tracked and logged the number and percentage of those who were eligible and interested, those who completed the intervention, and those who withdrew to provide insight into the study's recruitment efficiency and participant retention

Acceptability

Acceptability of the intervention and its implementation was assessed using qualitative and quantitative data. The aim was to detect any potential challenges within the methodology used to deliver both the trial and the intervention. We conducted post-intervention semi-structured qualitative interviews and focus groups. The intervention's acceptability was primarily gauged through a Likert-scale Satisfaction Questionnaire (14 items) applied to the intervention group (n=14). This questionnaire, originally devised by the Dan Abrahams Healthy Living Centre and subsequently employed by DeJesus et al. (2018), incorporates a variety of question styles (open & closed). Using a Likert scale, it offered a response range from 0 (not at all) to 10 (very much) to capture participant feedback. Given that this scale had not been used in Arabic studies before, we translated it into Arabic and pretested it on a smaller subset before implementing it for the full study sample.

For peer review only

Data Analysis

We conducted both quantitative and qualitative analyses. For the quantitative part, descriptive analysis was undertaken including screening and recruitment rates, retention rates, adherence to coaching sessions, and recruitment duration. We evaluated the use of BCTs and interactions between participants and coaches during these sessions. A Satisfaction Questionnaire captured participants' experiences, while changes in diabetes self-management and patient self-efficacy were assessed at the beginning and end of the study. The data were entered into Excel and subsequently transferred to SPSS and STATA for advanced analysis, which included a linear regression to estimate the mean differences in outcomes.

For the qualitative aspect of our study, purposive sampling was employed to ensure data saturation in conducting interviews and focus groups. We conducted a total of 14 semi-structured interviews and two focus groups, engaging in detailed discussions with participants. The qualitative data were analysed using NVivo software through a reflexive thematic analysis process, adhering to six systematic phases: familiarising ourselves with the data, coding, generating themes, revising themes, defining, and finally reporting (24). During this process, consensus was reached through regular team meetings and discussions to ensure accuracy and comprehensiveness in the identification and categorization of themes.

In addition, we integrated the qualitative and quantitative data for comprehensive understanding (25), Using a joint display table (see Supplemental Table 4) (26), we compared and combined these data types. The display helped us identify areas of agreement (convergence), disagreement (divergence), and enhancement (expansion) between the two data types (27). In the integrated data, we denoted convergence with "=", complementarity with "+", and inconsistencies with "≠".

Although this feasibility study may not conclusively determine intervention effects, it provides valuable insights for estimating the sample size for a larger trial based on the mean difference and standard deviation of HbA1c.

Results:

Participant Characteristics

The control group (mean age=53.40, SD=8.47) was slightly older than the intervention group (mean age=52, SD=8.32). The majority were married, with monthly incomes ranging from less than 5000 SR (28.6%) to more than 15000 SR (7.1%); 50.0% chose not to declare. About a quarter had completed high school, and an equal proportion had primary education or were literate without formal education. Approximately 31% had lived with T2DM for over ten years, with 24.1% of the intervention group having T2DM for 1-3 years. The majority (89.7%) were on diabetes medications. Table 1 summarises the demographic characteristics of both groups.

Table 1: Summary of the participants' demographic characteristics

For peer review only - http://bmjopen.bmj.com/site/about/guidelines.xhtml

		Intervention group		Control group	
		Total N (%)	Median (IQR)	Total N (%)	Median (IQR)
Gender	Male	6 (42.9%)		7 (46.7%)	
	Female	8 (57.1%)		8 (53.3%)	
Age	Year	14	54.5 (44.5 to 59.25)	15	54 (50 to 60)
Marital status	Married	14 (100.0%)		14 (93.3%)	
	Single	0 (0.0%)		1 (6.7%)	
Monthly income	less than 5000 (SR)	4 (28.6%)		7 (46.7%)	
	5000-10000 (SR)	2 (14.3%)		0 (0.0%)	
	10000-15000 (SR)	0 (0.0%)		2 (13.3%)	
	more than 15000 (SR)	1 (7.1%)		1 (6.7%)	
	Prefer not to declare	7 (50.0%)		5 (33.3%)	
Education level	Illiterate	0 (0.0%)		0 (0.0%)	
	Primary school	3 (21.4%)		3 (20.0%)	
	Secondary school	2 (14.3%)		2 (13.3%)	
	High school	3 (21.4%)		4 (26.7%)	
	Bachelor's degree	2 (14.3%)		3 (20.0%)	
	Diploma	1 (7.1%)		0 (0.0%)	
	Can read and write	3 (21.4%)		3 (20.0%)	
Since when you were diagnosed with Type 2 diabetes	less than a year	2 (14.3%)		0 (0.0%)	
	1-3	6 (42.9%)		0 (0.0%)	
	3-5	1 (7.1%)		2 (13.3%)	
	5-7	0 (0.0%)		1 (6.7%)	
	7-10	1 (7.1%)		6 (40.0%)	
	more than ten years	3 (21.4%)		6 (40.0%)	
	do not know	1 (7.1%)		0 (0.0%)	
Do you use diabetes medications ?	Yes	12 (85.7%)		14 (93.3%)	
	No	2 (14.3%)		1 (6.7%)	

For peer review only

Feasibility of the Intervention

Eligibility and Recruitment

The recruitment process took about five weeks, starting on May 1st, 2021, to June 5th, 2021. Leaflets were used for advertising the intervention and were distributed in different places in the hospital, including in waiting areas and on hospital wall notice boards. In addition, we met physicians in-person to introduce the intervention and gave them leaflets with more details to encourage their patients to join in the study.

Of the 42 potential participants initially identified and assessed for eligibility, 38 met the study criteria, resulting in a screening rate of approximately 90%. These 42 individuals were referred by different sources. Twenty-two were referred by their doctors, while the others were recruited through various methods. Two were from leaflets, eight were from suggestions by friends or relatives, and ten were directly engaged at the diabetes clinic. Twelve were excluded, of which four were ineligible because they did not meet the intervention inclusion criteria for the following reasons: one had no access to a personal mobile phone/smartphone, one patient was diagnosed with T1DM, one had A1c below 7%, and one was unable to do pre-and post-assessments. Eight were excluded for other reasons; two did not respond, and six for different reasons, e.g., being too busy. Thirty eligible patients were recruited to take part in the intervention. Out of the 38 eligible patients, 30 were successfully recruited and consented to participate in the intervention, yielding a recruitment rate of approximately 79%. All of them completed baseline assessments and questionnaires, and then randomly, 15 patients were allocated to the intervention group, while the other 15 participants were assigned to the control group. One person who was part of the intervention group withdrew from the trial prior to the first session and was thus not included in the study (see Supplemental Figure 1 CONSORT diagram for more details).

Intervention Delivery

The intervention started with the first session on June 15th, 2021, and ended on September 23rd 2021. The average time spent per participant was between ~ 17.7 to 25.5 in each session. The total range time of all sessions per patient was 109-153 minutes, with an average of 120.8 (SD= 13.7). See Supplemental Table 5 for more details about the time duration of each session.

Retention of Participants

Thirty eligible participants consented to participate in the trial and completed the baseline assessment randomly allocated either to the coaching group (n=15) or the control group (n=15). Of those, all twenty-nine who started the intervention and took the first session remained till the endpoint and completed all the intervention activities. Only one participant allocated to the coaching group had withdrawn and discontinued the study before the first session (retention rate = 97%). All twenty-nine have completed the intervention endpoint assessments. The main progress criteria and feasibility measurements are summarised in Supplemental Table 6.

Adherence to the Coaching Sessions

Fifteen out of thirty participants were randomly allocated to the coaching group. Before the first session, one participant withdrew (P12) due to family issues. The rest of the fourteen have

For peer review only - http://bmjopen.bmj.com/site/about/guidelines.xhtml

completed all their coaching sessions (adherence rate 100%), which met the predetermined progression criteria of adhering to ≥ 84%. Half of the participants took their sessions at the planned time as previously scheduled. However, health coaches have rescheduled different sessions for seven participants for different reasons. The rescheduled sessions varied between 1 to 2 sessions for each one of them. Out of 84 sessions, only eleven sessions have been rescheduled (13%). The reasons for the rescheduling were home/work/appointment conflicts (n = 6 sessions), travel (n=1 session), illness (n=3 sessions), and being busy (n=1 session). All participants started their coaching sessions at the same time on June 15th, 2021, except four participants who started later on July 1st, 2021. A Fifteen-day delay because their coach could not begin at that time for family issues. In addition, the same four participants had another delay between the fourth and fifth sessions due to the coach's college exams. This led them to finish their last session on September 23rd, 2021.

Data Collection Adherence:

Participants recruited for the study were invited to Al-Zulfi General Hospital on June 10th, 2021. During this visit, they were given consent forms and information sheets about the intervention, providing them with an opportunity to ask questions and discuss any concerns related to their participation. Furthermore, baseline data, including measures of HbA1c, blood pressure, BMI, weight, and waist circumference, were collected during this visit. Due to the restrictions imposed by COVID-19, all required measurements and paperwork were completed in a single visit. Participants were then asked to complete two electronic questionnaires, the Diabetes Self-Care Activities (SDSCA) scale, and the Self-efficacy Scale for Diabetes, during the hospital visit on June 10th, 2021. The baseline data collection was successfully completed by all 30 participants, yielding a 100% completion rate. The time taken to complete the questionnaires ranged from 7 to 12 minutes, with no participants reporting difficulties or issues in the completion process.

At the end of the intervention, participants were once again invited to the hospital to gather post-intervention data. This second data collection session was much like the first, where we again measured HbA1c, blood pressure, BMI, weight, and waist circumference. Participants were also asked to complete the initial two questionnaires, with the addition of a Likert-scale Satisfaction Questionnaire for the intervention group only. This additional questionnaire resulted in a slightly longer completion time, ranging from 9 to 15 minutes, due to the inclusion of open-ended questions and certain items requiring justifications or explanations for selected responses. Overall, all participants effectively completed the data collection process, demonstrating successful adherence to our study's data collection procedures.

Progression Criteria

The feasibility measurements and predetermined progression criteria yielded positive results. The screening rate was 90%, exceeding the target of 80%, suggesting proceeding to the future definitive RCT. The recruitment rate reached 79%, demonstrating robust engagement with the target population. The retention rate at three months was 97%, surpassing the required 83% rate. Baseline data collection adherence and intervention adherence both achieved 100%. Endpoint data collection adherence was 97.6%, with only one participant withdrawing before

For peer review only - http://bmjopen.bmj.com/site/about/guidelines.xhtml

the first session. Overall, these results support the decision to proceed to the future definitive RCT, indicating the feasibility and acceptability of the health coaching approach for individuals with T2DM in SA.

Sample Size

The sample size for this feasibility study was determined in consultation with a statistician and was guided by practical considerations as recommended by the CONSORT guidelines for feasibility trials (28). Our aim was not to test the intervention for statistical significance but rather to assess the feasibility and variability of outcomes to inform the planning of a definitive RCT. The choice of sample size was also influenced by the need to manage resources efficiently and to ensure a broad enough representation of the target population to capture initial insights into the intervention's acceptability and implementation.

Furthermore, the statistician advised that for the upcoming main trial, a sample size calculation should be based on a clinically significant mean difference of 0.5% and a standard deviation of 1.4, given the substantial effect size of -0.93 observed in this feasibility study. With these parameters, using a power of 0.8 and an alpha level of 0.05, we estimated that 125 participants per group would be necessary. This sample size would not only allow us to detect a clinically significant difference but also accommodate subgroup analyses to explore variations in effectiveness across different settings or patient groups.

Acceptability of the Intervention

Acceptability and suitability of the intervention were assessed through a questionnaire (Likert-scale Satisfaction Questionnaire, 14 items) for the intervention group (n = 14). Using a Likert scale of 0 (not at all) to 10, most participants' responses show that the intervention affected their behaviour "quite a bit " (mean: 8.2 (SD= 2.3)). Participants replied, “very much” (mean 9.2 (SD= 1.4)) when asked how much participation in the intervention helped them establish a personal vision of wellness. When asked how the intervention boosted their confidence in taking actions toward improved well-being, the overall response was "very much" (mean 9.2 (SD= 1.4)). Their motives for making efforts toward enhanced well-being were "quite a bit" (mean 8.3 (SD= 1)). The average response to the usage of goal setting was "very much" (mean 9.2. (SD= 0.9)). Whereas their mean response to their use of problem-solving abilities was "quite a bit" (mean 8.6 (SD= 1.4)). Participants shared that the intervention greatly assisted them in overcoming obstacles and achieving increased levels of wellness, with a high mean score of 9 (SD= 1.1) reported. From the first through the eighth question, participants' responses are presented in Supplemental Table 7.

Participants were asked three "Yes" or "No" questions, and they all replied "Yes" when asked whether they expected to continue making improvements (n=14, 100%). The majority (n=13, 92.9%) responded with "Yes" the intervention met their expectations. All 14 participants who were asked whether they would recommend the intervention to others responded with a positive "yes".

For peer review only - http://bmjopen.bmj.com/site/about/guidelines.xhtml

Thematic Analysis Findings

We analysed 1691 minutes of coaching sessions to identify the BCTs used (see Supplemental Table 8) and transcribed 497 minutes of focus groups and interviews. A native Arabic speaker confirmed the accuracy of the entire transcription. To further ensure validity, about 10% of the transcription (50 minutes) underwent a back-translation process checked by a professional native speaker. Throughout the intervention, the first author (A.N.A), acting as the researcher, maintained field notes. Due to COVID-19 limitations, all interviews and focus groups were conducted online.

Evaluation of Participants and Health Coaches' Experiences

Various qualitative methods were used to assess participant experiences during the intervention, including interviews, focus groups, and field notes. After the intervention, two focus groups took place. Participants were given time to understand the questions before engaging in interviews and focus groups, fostering a deeper discussion. The researcher (A.N.A) conducted all focus groups and interviews, enabling active interaction and understanding of participant perspectives.

Between June 15th and September 23rd, 2021, 14 semi-structured interviews were conducted with seven participants (4 males, three females) from the intervention group, both pre-and post-study. Participants predominantly had a high school education or less, and the median age was 59. Three had long-standing diabetes of over ten years, three had a recent diagnosis of less than three years, and one was uncertain of the duration. Six of the seven were on diabetes medication.

Two post-study focus groups were conducted online. One focus group comprised six participants (three males and three females) from the intervention group with a median age of 52.5 years. The majority had a high school education or less, and most were on diabetes medication with a mix of recent and long-term diagnoses. The demographic characteristics of the participants can be seen in Table 2.

Three female health coaches from KFMC in Riyadh formed the second focus group to share their intervention delivery experiences. All had health coaching backgrounds, with two holding master's degrees in health education. The session lasted 265 minutes.

For peer review only

Table 2: Participants' Demographic characteristics

		**Qualitative interview participants (n=7)**		**Focus group participants (n=6)**	
Demographic characteristics		Number	%	Number	%
Gender	Male	4	57.1	3	50
	Female	3	42.9	3	50
Age	Years	43 to 62	Median 59	34 to 60	Median 52.5
Marital status	Married	7	100	6	100
Monthly	less than	1	14.3	2	33.3

For peer review only - http://bmjopen.bmj.com/site/about/guidelines.xhtml

income	5000				
	5000-10000	1	14.3	1	16.7
	10000-15000	1	14.3	0	
	Prefer not to declare	4	57.1	3	50
Education level	Primary school	2	28.6	1	16.7
	Secondary school	2	28.6	1	16.7
	High school	2	28.6	3	50
	Bachelor's degree	1	14.3	1	16.7
Since when you were diagnosed with Type 2 diabetes	less than a year	2	28.6	1	16.7
	1-3	1	14.3	3	50
	more than ten years	3	42.9	1	16.7
	do not know	1	14.3	1	16.7
Do you use diabetes medications?	Yes	6	85.7	5	83.3
	No	1	14.3	1	16.7

We used thematic analysis with inductive and deductive coding to evaluate the trial's acceptability and feasibility. Open coding initially identified emergent themes, with deductive coding following, grounded in qualitative research guidelines and the Medical Research Council (MRC) framework (29).

The analysis of translated transcripts produced four key themes, two of which were predetermined. The first theme pertained to trial design, conduct, and processes, divided into four sub-themes covering different aspects of intervention implementation. The second theme focused on intervention content and delivery, consisting of sub-themes on intervention components, perceived consequences, feasibility and acceptability in practice, and reach and dose. The third theme, intervention mechanism, encompassed sub-themes around COM-B model application, use of BCTs and intervention curriculum, and intervention impact. The fourth theme, future RCT, captured participant and coach suggestions. The thematic map used in the analysis is outlined in Supplemental Figure 2.

Theme 1: Intervention Design, Conduct, and Processes

This theme primarily focuses on the implementation and design of the intervention, along with the processes involved. Recruitment challenges during the pandemic prompted a shift to remote settings. This transition not only made recruitment a challenging task but also stretched the recruitment period beyond the planned timeframe.

For peer review only - http://bmjopen.bmj.com/site/about/guidelines.xhtml

For peer review only

Notably, a health coach stated that the recruitment phase was complicated as clinics moved online., *"...the recruitment phase, it was complicated only because all clinics become online and we could not see people with type 2 diabetes come to the hospital as usual, so the chance of meeting those people was very rare."* (Focus Group with Coaches, FGC01, Female).

Misunderstandings about health coaching in SA arose from a lack of understanding and familiarity. This highlighted the need for improved communication with recruiters, as one health coach observed, *"...from my communication with recruiters, some of them did not understand, so participants may get a wrong idea"* (Focus Group with Coaches, FGC01, Female).

Limited hospital access due to public health guidelines complicated recruitment further. However, the suggestion to recruit from a secondary hospital made the recruitment process manageable. Participants and health coaches recommended physicians as intermediaries for patient recruitment due to the trust and rapport they have established with patients; as one of them stated, *".....I think one of the best and most reliable ways to reach and recruit patients is to start from their direct physicians by suggesting the program for patients and encourage them to join"* (Focus Group with Coaches, FGC02, Female).

Proposing clear communication about program expectations aimed to reduce dropout rates, with one participant suggesting to *"ask participants to sign a contract to commit and if you are hesitating or you have the intention to withdraw to tell us [referred to program provider] at the beginning to give the opportunity to someone who needs it"* (Focus Group with Participants, P03, Female).

Health coaches and participants expressed general satisfaction with the intervention's design, and they believed that it effectively accommodated the local context of Saudi society. One participant expressed, *"I'm very very very satisfied and I think the program is easy to follow and very acceptable as it gradually improves different skills which last with a patient after the program…it is completely different from other programs.."* (Interview with Participants, P13, Male).

Additional workload challenged health coaches, mixing intervention tasks with regular duties. The COVID-19 pandemic further complicated matters by requiring greater flexibility in scheduling coaching sessions, particularly for participants with children engaged in online learning. To alleviate these issues, health coaches proposed securing formal workplace approval to dedicate full-time to the intervention. As one health coach noted, *"As you know and because of the workload, I was thinking of withdrawing from the intervention...... I think it is imperative to work a full-time job as a health coach in the intervention, not as a volunteer, and get formal permission from my work on this.."* (Focus Group with Coaches, FGC01, Female).

Theme 2: Intervention Content and Delivery

This theme encompassed multiple sub-themes reflecting on the intervention's implementation, including its acceptability and adaptability within the Saudi context, potential unintended consequences, the feasibility of the practical application, and intervention reach and dosage.

For peer review only - http://bmjopen.bmj.com/site/about/guidelines.xhtml

Health coaches suggested modifications for better acceptability and suitability of the intervention to the Saudi context. Emphasising the need for professionalism and structure, they proposed using formal platforms for communication, as one coach stated, *"...communication with the participant should be through a formal platform or application... to make the program more formal and organised..."* (Focus Group with Coaches, C02, Female). They also suggested the importance of physical meetings or video calls for a more serious engagement, as expressed by another coach, *"I prefer to have a physical meeting where I can sit with the patient face-to-face…"* (Focus Group with Coaches, FGC01, Female).

Coaches highlighted the necessity of prepared private spaces for coaching sessions. to give a professional image to the intervention. As one coach expressed, *"..As a coach, there should be a certain prepared private place at work to conduct all of my coaching sessions.."* (Focus Group with Coaches, C02, Female).

Participants preferred visual coaching sessions over phone calls for effective communication. Coaches felt that seeing the participant was vital for effective communication and coaching. A health coach expressed this, saying, *"...visual interaction with participants is very very important, you know the importance of reading and understanding the body language for me as a coach.."* (Focus Group with Coaches, C03, Female).

Participants praised the intervention for its straightforwardness and effectiveness in helping them make progress towards their goals. Some suggested a desire for additional sessions to further adopt and maintain new habits. One participant commented, *"I think three months was enough"* (Interview with Participants, P03, Female), *while another remarked, "…it enabled me to stay committed, continue, and achieve my goals"* (Interview with Participants, P09, Male).

Participants appreciated the use of health coaching techniques, such as action plans and SMART goals, which helped set achievable targets and keep them motivated. The flexibility and graduality of the program were also well-received, as one participant mentioned, *"...the most thing I liked about the program was the graduality to achieve goals .."* (Interview with Participants, P13, Male).

Coaches confirmed the intervention content's acceptability and observed its positive impacts. They also suggested the addition of monthly sessions or an extension of the intervention to six months for better patient progress monitoring and maintenance. A coach stated, *"... I suggest the intervention last longer… I think it would be good if the intervention lasts six months with a session each month …"* (Focus Group with Coaches, FGC01, Female).

Theme 3: Intervention Mechanism

This theme emphasises the practical application and effects of the COM-B health coaching model. Coaches received specialised training on the curriculum, and BCTs, utilising several tools for efficient delivery. Notably, coaches documented the BCTs employed in each session, enabling tailored strategies to target specific behaviours. Participants appreciated this approach, stating, *"The BCTs helped me during each session to address some behaviours…"* (Focus Group with Coaches, FGC01, Female).

For peer review only - http://bmjopen.bmj.com/site/about/guidelines.xhtml

The COM-B model played a crucial role in the intervention, targeting four behaviours. Participants noted marked improvements in their knowledge and motivation pre-and post-intervention. Testimonials showed that they were more knowledgeable about their condition, diet, and physical activity. One participant said, *"I became more knowledgeable...now I know diet types and what carbohydrates mean…"* (Focus Group with Participants, P05, Female).

The intervention also impacted participants' social and physical environment, leading to a supportive atmosphere for behavioural change. Participants indicated that the changing social environment in SA encouraged the adoption of a healthier lifestyle and influenced their social networks. As one participant put it, *"While I was changing some of my old habits, my social network adapted..."* (Interview with Participants, P03, Female).

Health coaching provided a more personalised approach than traditional care, fostering motivation and leading to a change in participants' views about their condition. This motivation was further fuelled by the noticeable changes in outcomes such as HbA1c levels. Participants recognised the power of lifestyle changes and felt empowered by their improved health results, stating, *"...after the intervention, I realised modifying the lifestyle is the actual treatment..."* (Interview with Participants, P13, Male). Consequently, there were requests to extend the intervention, given the observable impacts, including reductions in A1c levels and body weight.

Participants in SA observed notable differences between the novel concept of health coaching and traditional care. The former was seen as superior, with participants expressing dissatisfaction with traditional care's impersonal nature. Health coaching, in contrast, provided a more personalised approach, allowing patients to voice their concerns. As one participant expressed, *"I would choose this program without hesitation; at least I finish each session while I'm comfortable..."* (Interview with Participants, P03, Female).

Traditional care was perceived as a top-down process, with patients passively receiving pre-prepared information or plans. The health coaching intervention, however, positioned participants as active stakeholders in their own care by encouraging goal-setting and plan creation. A participant noted, *"Your program helped me use new skills such as setting plans and having short-term and long-term goals..."* (Interview with Participants, P08, Male).

Frustration and even anger marked participants' feelings about traditional care, citing time constraints, a lack of empathy, and the expense of private consultations. Health coaching was seen as a way to alleviate these issues, with participants suggesting the incorporation of health coaching clinics in hospitals to facilitate meaningful patient discussions and foster better disease self-management. A participant suggested, *"I think it is important to recruit a coach in the hospital... 90% of patients with chronic diseases need to sit with someone to have meaningful discussions..."* (Interview with Participants, P08, Male).

Theme 4: Improvement Opportunities in the Future Health Coaching RCT

As the inaugural health coaching intervention within the Saudi healthcare system, this initiative stands as a landmark reflecting the strategic direction of the Saudi Ministry of Health towards more proactive, patient-centred care. In anticipation of scaling up such interventions, participants and health coaches provided constructive feedback for refinement.

For peer review only - http://bmjopen.bmj.com/site/about/guidelines.xhtml

Participants expressed a preference for more frequent coaching interactions and the opportunity for extended face-to-face engagements, suggesting a deeper and more personal connection could enhance the intervention's impact. "*Having more time and direct sessions would greatly benefit the experience*" shared one participant (Interview with Participants, P01, Female).

Health coaches recommended the integration of multidisciplinary expertise, particularly dietitians and physicians, to provide comprehensive care and address the complex needs of diabetes management. "*Incorporating a nutritionist and a physician would offer a more rounded approach to patient care,"* proposed a coach (Focus Group with Coaches, C03, Female). This suggestion is indicative of a holistic strategy, recognizing the multifactorial nature of diabetes.

To bolster participant engagement, the provision of incentives was suggested, alongside enhanced access to healthcare services. Additionally, health coaches proposed the creation of educational content, such as a succinct video delineating the health coaching concept, to facilitate a clearer understanding for future participants. "*A visual introduction to health coaching might bridge the initial knowledge gap,*" a coach highlighted (Focus Group with Coaches, C02, Female).

Preliminary Effects of the Health Coaching Intervention

This study investigated the impact of a health coaching intervention on primary and secondary outcomes among individuals with T2DM. The preliminary intervention effects consisted of primary outcomes, including HbA1c, and secondary outcomes such as Mean Arterial Pressure (MAP), BMI, Waist Circumference, Weight, Patients' self-efficacy, and Diabetes self-management.

A linear regression model was used to evaluate the efficacy of a health coaching intervention between the two groups, adjusted for baseline levels. The primary outcome, HbA1c, revealed a substantial reduction in the intervention group compared to the control group, with a mean difference of -1.86 (95% CI: -2.71 to -1.01, P<0.001). Secondary outcomes, including MAP, exhibited notable group differences at the study's endpoint (mean difference: -0.68, 95% CI: -7.46 to 6.09, P=0.83). While both groups experienced a modest decline in weight, the change in the intervention group was not statistically significant (mean difference: -2.58, 95% CI: -5.25 to 0.082, P=0.057). Conversely, BMI demonstrated a significant post-intervention difference between the two groups (mean difference: -1.02, 95% CI: -2.01 to -0.041, P=0.042). Waist circumference also exhibited a statistically significant difference in the intervention group compared to the control group (mean difference: -6.89, 95% CI: -10.17 to -3.61, P<0.001, paired t-test). For more details, see Tables 3 and 4.

Table 3: Groups means and SDs at study baseline and endpoint.

	Group	N	Mean	Std. Deviation
A1c level at the study baseline	Intervention Group	14	9.12	2.50

For peer review only - http://bmjopen.bmj.com/site/about/guidelines.xhtml

	Control Group	15	8.73	1.69
A1c level at the study endpoint	Intervention Group	14	7.29	1.38
	Control Group	15	8.93	1.81
Mean Arterial Pressure at the study baseline	Intervention Group	14	91.7	13.56
	Control Group	15	86.73	11.66
Mean Arterial Pressure at the study endpoint	Intervention Group	14	92.57	9.11
	Control Group	15	90.33	12.91
Weight in Kg at the study baseline	Intervention Group	14	83.50	11.97
	Control Group	15	81.21	12.76
Weight in Kg at the study endpoint	Intervention Group	14	80.70	10.80
	Control Group	15	81.21	12.60
BMI at the study baseline	Intervention Group	14	30.39	4.57
	Control Group	15	30.87	5.43
BMI at the study endpoint	Intervention Group	14	29.39	4.33
	Control Group	15	30.86	5.318
Waist Circumference (cm) at the study baseline	Intervention Group	14	109.35	13.60
	Control Group	15	106.66	10.22
Waist Circumference (cm) at the study endpoint	Intervention Group	14	102.21	12.55
	Control Group	15	106.76	9.91

Table 4: Outcomes mean differences taking into account the baseline value regression

Endpoint HbA1c	Mean difference	t	p-value	*d*	[95% CI]	
Group*	-1.864463	-4.50	<0.001	-0.93	-2.716065	-1.012862
Baseline HbA1c	.566714		<0.001		.3601448	.7732833

For peer review only - http://bmjopen.bmj.com/site/about/guidelines.xhtml

Endpoint MAP	Mean difference	t	p-value	[95% CI]	
Group*	-.6833264	-0.21	0.837	-7.463123	6.09647
Baseline MAP	.5809645	4.38	<0.001	.3083653	.8535638
Endpoint Weight	Mean difference	t	p-value	[95% CI]	
Group	-2.584974	-1.99	0.057	-5.252461	.0825133
Baseline Weight T1	.9090889	16.84	<0.001	.7981218	1.020056
Endpoint BMI	Mean difference	t	p-value	[95% CI]	
Group	-1.024214	-2.14	0.042	-2.006754	-.0416741
Baseline BMI T1	.9322785	19.02	<0.001	.8315092	1.033048
Endpoint WC	Mean difference	t	p-value	[95% CI]	
Group	-6.895251	-4.32	<0.001	-10.17924	-3.611259
Baseline WC	.8732792	12.72	<0.001	.7322131	1.014345

Abbreviations: HbA1c: Haemoglobin A1c, MAP: Mean Arterial Pressure, BMI: Body Mass Index, WC: Waist Circumference, t: t-test, CI: Confidence Interval, d: Cohen's d (Effect size) *(Group = Intervention mean – Control mean)

Patients' Self-Efficacy Questionnaire

The self-efficacy of patients was assessed using an 8-item scale (30), where scores ranged from 1 (least confident) to 10 (most confident). Higher mean scores indicated greater self-efficacy. Both intervention and control groups displayed an increased average response from baseline to the study endpoint, with higher improvements observed in the intervention group (see Supplemental Table 9). The groups exhibited a statistically significant difference (P = .006, 59 % CI .713 to 3.839) in confidence related to controlling their condition and maintaining exercise. Similarly, a significant difference (P = .006, 59 % CI 0.82 to 4.35) was noted in confidence regarding appropriate food choices when hungry. This suggests the intervention group saw greater improvements in self-efficacy compared to the control group.

Diabetes Self-Care Activity

The Diabetes Self-Care Activity scale (31), scoring 0 (no adherence) to 7 (complete adherence), measured patients' adherence to specific activities over the past week. Average scores showed overall improvements in self-care for both the intervention and control groups across all

For peer review only - http://bmjopen.bmj.com/site/about/guidelines.xhtml

activities, with the intervention group often meeting or exceeding American Diabetes Association recommendations (see Supplemental Table 10) (32). For instance, adherence to a healthy eating plan in the intervention group increased from an average of 1.64 to 5.00 days per week. Physical exercise also increased in both groups, with a larger improvement in the intervention group (3.57 to 5.64 days). Similarly, there was improved management of carbohydrate intake in the intervention group, from 1.71 to 4.00 days per week.

Integration Results:

The key findings from both quantitative and qualitative data, aligned with research objectives, were assembled in a joint display table (see Supplemental Table 5). This table was structured to present the study's critical concepts and the corresponding progression criteria, which provide a comprehensive understanding of the mixed-methods findings. The phrase 'there is no significance in moving forward', as used within our progression criteria, draws from the principle of setting clear benchmarks for feasibility studies. These benchmarks are pivotal in assessing whether a study exhibits the potential for upscaling to a full-scale RCT. They embody a balance between the ambition to proceed and the pragmatism to address early issues, as recommended by best practices in trial management (33). In our context, this phrase indicates that if participant recruitment, retention, or adherence fall below the predetermined percentages, the study may not have the robustness required for expansion. Such a stance encourages rigorous scrutiny of recruitment rates and intervention adherence to ensure only well-substantiated research advances.

The integration of quantitative and qualitative data insights offered enhanced comprehension of findings collated and analysed separately. This approach bolstered the credibility and transparency of the overall results through data triangulation, allowing for comparison and validation of findings that a single method wouldn't permit. The integrated results substantiated the pertinence of mixed approaches, essential to the study's feasibility and acceptability exploration in the Saudi context for a large-scale intervention with T2DM patients.

The study reported high screening, recruitment, and retention rates. These results were corroborated by the integrated analysis, suggesting a promising potential for the study within the Saudi setting. This was further supported by participants' willingness to engage in the intervention, extend invitations to family members, and their adherence to complete the intervention, despite some rescheduled sessions.

Notably, improvements were observed across five out of seven preliminary outcomes, which were validated by qualitative data. Participants and coaches reported positive experiences with the intervention's usability, design, and content. Moreover, qualitative results echoed the high satisfaction levels among participants identified in the quantitative Likert-scale Satisfaction Questionnaire. Despite the challenge posed by session rescheduling flexibility for coaches, it facilitated participant adherence, as supported by quantitative data: all participants completed their sessions with only eleven sessions rescheduled.

Discussion:

For peer review only - http://bmjopen.bmj.com/site/about/guidelines.xhtml

The findings from this feasibility study endorse advancing towards a larger RCT for a health coaching intervention among patients with T2DM in SA. Notably, the high rates of eligibility, recruitment, and retention reflect the study's effectiveness in engaging participants, which is pivotal for ensuring the representativeness and reliability of the results.

Achieving a recruitment rate of 79% is particularly significant; it not only indicates the interest and willingness of the target population to participate but also reinforces the potential for this health coaching intervention to be scaled up and applied broadly within the Saudi healthcare setting. This recruitment performance, especially when contrasted with rates from prior studies, such as those by Basak C & Schou (2013) and Cho et al. (2011) (34-36), highlights the tailored approach's resonance with the participants even amidst the operational challenges presented by the COVID-19 pandemic.

Retention is another key indicator of a study's appeal and the practicality of its intervention. The retention rate in this study exceeded that of a similar study (83.6%) (34), as well as the rates reported by Basak C & Schou (2013) (87%) (36) and Cho et al. (2011) (90%).(35) Our retention rate, which remained high throughout the study period, suggests that the intervention was well-received and retained its relevance and value to the participants, motivating them to continue through the study's conclusion. The flexibility allowed in rescheduling coaching sessions contributed to this success, though it did increase the workload for the coaches, an aspect to consider in the planning of future RCTs.

As we reflect on the integrated quantitative and qualitative findings, we see confirmation of the feasibility of conducting a larger RCT, despite restrictions due to COVID-19. The comprehensive data collection, appropriate outcome assessments, and participant adherence collectively point to the potential for effective execution and data gathering in a subsequent, more extensive RCT. Enhancing reach and diversity through a variety of recruitment methods, and the use of a concise explanatory video to describe the intervention, are strategies that can further streamline the process and solidify understanding among future participants.

The feasibility study utilised the COM-B model and the BCTs taxonomy, which proved to be effective in guiding intervention content and addressing participant barriers. The most frequently used BCTs aligned with previous literature. The study adapted to telephone-based sessions due to COVID-19 restrictions, but a mixed approach with face-to-face interactions was preferred by both participants and coaches. A longer intervention duration of up to six months with more face-to-face sessions was recommended to ensure sustained behaviour changes.

The intervention's positive outcomes, particularly the reduction in HbA1c levels, align with evidence suggesting that interventions emphasizing HbA1c as the primary outcome are more effective. This underscores the importance of selecting outcomes aligned with the intervention's central goal. This study supports previous findings (37), indicating that using HbA1c as the primary intervention outcome can enhance effectiveness. Emphasizing glycaemic control as the primary outcome significantly impacted participants' diabetes self-management in this study. Participants actively developed and utilized key skills to manage their condition, indicating the efficacy of the coaching model. Their satisfaction was notably

For peer review only - http://bmjopen.bmj.com/site/about/guidelines.xhtml

linked to a meaningful reduction in HbA1c levels after the intervention, emphasizing the holistic impact of the health coaching model.

Although feasibility studies are not formally powered to detect conclusive effects on interventions (38), this study demonstrated the high acceptability and usability of the intervention in the Saudi context. However, concerns were raised by health coaches regarding their volunteer roles and the challenges of finding suitable locations for coaching sessions. These issues need to be addressed in planning for the larger RCT. The ongoing societal changes in SA, along with participants' satisfaction with the intervention, suggest potential acceptance for a large-scale RCT.

For the upcoming definitive RCT, it's crucial to integrate participant and coach feedback to refine the approach. Recommendations include lengthening the program to six months with increased in-person interactions, involving multidisciplinary experts, regular coach coordination, participant incentives, and a brief explanatory video to introduce health coaching.

This study introduces a patient-cantered health coaching approach to Saudi Arabia, showcasing its effectiveness in managing T2DM and its potential applicability to other chronic conditions. The use of a detailed curriculum and the COM-B model has proven beneficial, supporting the Saudi Ministry of Health's move towards behaviour-based strategies and paving the way for broader implementation.

The study's positive reception and outcomes reinforce the viability of health coaching in T2DM management, offering insights for future research and substantiating its growing acceptance and practicality.

Strengths and Limitations of this Study

This study applied the BCW framework, proving beneficial for a comprehensive understanding of the issue of T2DM in the context of SA through an in-depth analysis of prior literature. Accurate diagnosis of the identified problem, considering potential barriers and facilitators, plays a pivotal role in the systematic roadmap. This process initiates with recognizing the actual issue and concludes with specifying target behaviours necessary to achieve the intended goals. Employing the COM-B model, we conducted a narrative review to pinpoint facilitators and barriers in controlling T2DM. The unique approach involved employing the BCW, COM-B model, and BCTs taxonomy, enabling a meticulous analysis of each health coaching session and the coding of each applied BCT. This resulted in a clear and detailed description of the intervention.

The inclusion of progression criteria, as recommended by the CONSORT 2010 statement, aided in decision-making for the larger trial (39). The study findings, including the effect size and retention rate, will inform the estimation of the sample size for the future main trial. The study also utilised both the Summary of Diabetes Self-Care Activity questionnaire and the Self-efficacy Scale for Diabetes scale, providing a comprehensive assessment of health coaching's impact. A mixed methods approach was employed to gather diverse perspectives, enhance validity and reliability, and produce more comprehensive findings.

For peer review only - http://bmjopen.bmj.com/site/about/guidelines.xhtml

However, the study is not without limitations, particularly concerning generalizability due to the specific setting and population. The small sample size may have led to potential Type 2 errors, limiting the study's ability to detect certain effects. While the recruitment of the intervention sample from a public hospital may not be directly applicable to primary care settings, it could impact the broader generalizability of findings to other individuals.

It is essential to note that although statistically significant effects were detected, the study's acknowledgement of limitations in statistical power reflects an awareness of potential constraints in identifying smaller yet existing effects. These limitations should be considered when interpreting the study's outcomes.

Conclusion

This mixed methods feasibility RCT demonstrated the feasibility and acceptability of implementing a health coaching approach for individuals with T2DM in SA. The integration of qualitative and quantitative findings yielded positive outcomes, providing strong support for conducting a larger RCT to further investigate the effectiveness of health coaching in enhancing diabetes self-management among individuals with T2DM in SA. Importantly, this study offers valuable insights for future trials, particularly in terms of incorporating the COM-B model and BCTs taxonomy into the health coaching approach. The positive feedback received for implementing a patient-centred approach indicates that it could help manage other chronic diseases. This aligns with the Saudi Arabian Ministry of Health's behaviour-focused strategies, providing more evidence to support the expansion of health coaching. As a result, these insights have significant implications for the current management practices of T2DM and beyond. Future research should also explore the social and environmental impacts of Vision 2030, the national vision of SA, on diabetes self-management. By considering contextual factors that may influence the implementation and outcomes of health coaching interventions for diabetes management in SA, further investigations will deepen our understanding and inform strategies to improve diabetes care and other chronic diseases in the country.

For peer review only

Supplementary Figure:

Supplementary Figure 1: CONSORT Flow Diagram of Participant Progress through the Phases of the Randomised Trial. This diagram depicts the enrolment, allocation, follow-up, and analysis stages of participants. It includes the number of individuals assessed for eligibility, excluded, or randomised into the trial, as well as those who received the intervention, followed up, and were included in the analysis.

For peer review only - http://bmjopen.bmj.com/site/about/guidelines.xhtml

Supplementary Figure 2: Detailed Thematic Analysis of Intervention Components. This map illustrates the comprehensive thematic breakdown of the intervention, highlighting key areas such as feasibility, mechanisms, and potential improvements. It details the sub-themes of intervention design, content delivery, and participant engagement, as well as the impact on trial staff and researchers. Suggestions for the future RCT based on coaches' and participants' feedback are also included.

For peer review only

Author Contributions: A.N.A. was responsible for the study's conception and design, data acquisition, analysis, and interpretation, as well as the drafting the manuscript. E.G. contributed to the critical revision of the manuscript based on data analysis. AA, AM, MA, A.S.A were responsible for the implementation of the intervention. All authors have thoroughly reviewed and approved the final version of the manuscript for publication

Funding: This study received no external funding.

Competing interests: The authors declare no conflicts of interest.

Patient consent for publication: Not required.

Ethics approval: Ethical approval for this study was granted by both the University of Sheffield and the Institutional Review Board (IRB) committee at King Fahad Medical City (KFMC), with the assigned IRB Log Number: 21-062E.

Data Availability Statement: All data generated or analysed during this study are available upon reasonable request from the corresponding author.

Acknowledgement: We express our sincere gratitude to the study participants for their valuable time and willingness to participate. We are also grateful to KFMC and Al-Zulfi General Hospital for granting permission to conduct the intervention. Furthermore, we extend our thanks to the University of Sheffield Institutional Open Access Fund for their support towards this research. Finally, we would like to acknowledge that this paper will be published under a Creative Commons Attribution (CC BY) license.

For peer review only

References

1. Alqurashi KA, Aljabri KS, Bokhari SA. Prevalence of diabetes mellitus in a Saudi community. Annals of Saudi Medicine. 2011;31(1):19-23.

2. Association AD. Diagnosis and classification of diabetes mellitus. American Diabetes Association; 2010. p. S62-S.

For peer review only - http://bmjopen.bmj.com/site/about/guidelines.xhtml

3. Hex N, Bartlett C, Wright D, Taylor M, Varley D. Article: Health Economics Estimating the current and future costs of Type 1 and Type 2 diabetes in the UK, including direct health costs and indirect societal and productivity costs.
4. Alqahtani M, Almutairi FE, Albasseet AO, Almutairi KE. Knowledge, Attitude, and Practice of Diabetes Mellitus Among the Saudi Population in Riyadh, Saudi Arabia: A Quantitative Study. Cureus. 2020.
5. Organization WH. Noncommunicable diseases 2022 [Available from: https://www.who.int/news-room/fact-sheets/detail/noncommunicable-diseases.
6. Alneami YM, Coleman CL. Risk Factors for and Barriers to Control Type-2 Diabetes among Saudi Population. Global Journal of Health Science. 2016;8(9).
7. Alabdulbaqi D. A Cultural Adaptation of a Diabetes Self-Management Programme for Patients with Type 2 Diabetes Attending a Primary Health Care Centre in Saudi Arabia. 2019.
8. Al Slamah T, Nicholl BI, Alslail FY, Harris L, Melville CA, Kinnear D. Cultural adaptation of self-management of type 2 diabetes in Saudi Arabia (qualitative study). PLOS ONE. 2020;15(7):e0232904-e.
9. Wolever RQ, Dreusicke M, Fikkan J, Hawkins TV, Yeung S, Wakefield J, et al. Integrative Health Coaching for Patients With Type 2 Diabetes. The Diabetes Educator. 2010;36(4).
10. Wong-Rieger D, Rieger FP. Health coaching in diabetes: empowering patients to self-manage. Elsevier; 2013. p. 41-4.
11. Hill B, Richardson B, Skouteris H. Do we know how to design effective health coaching interventions: A systematic review of the state of the literature. American Journal of Health Promotion. 2015;29(5):e158-e68.
12. Pirbaglou M, Katz J, Motamed M, Pludwinski S, Walker K, Ritvo P. Personal Health Coaching as a Type 2 Diabetes Mellitus Self-Management Strategy: A Systematic Review and Meta-Analysis of Randomized Controlled Trials. SAGE Publications Inc.; 2018. p. 1613-26.
13. Hill B, Richardson B, Skouteris H. Do we know how to design effective health coaching interventions: a systematic review of the state of the literature. American journal of health promotion : AJHP. 2015;29(5):e158-68.
14. Alanazi FK, Alotaibi JS, Paliadelis P, Alqarawi N, Alsharari A, Albagawi B. Knowledge and awareness of diabetes mellitus and its risk factors in saudi arabia. Saudi Arabian Armed Forces Hospital; 2018. p. 981-9.
15. Eldridge SM, Chan CL, Campbell MJ, Bond CM, Hopewell S, Thabane L, et al. CONSORT 2010 statement: Extension to randomised pilot and feasibility trials. The BMJ. 2016;355.
16. Almulhim AN, Goyder E, Caton SJ. Assessing the Feasibility and Acceptability of Health Coaching as a New Diabetes Management Approach for the People with Type 2 Diabetes in Saudi Arabia: A Protocol for a Mixed Methods Feasibility Study. International Journal of Environmental Research and Public Health. 2022;19(22):15089.
17. Hariton E, Locascio JJ. Randomised controlled trials – the gold standard for effectiveness research: Study design: randomised controlled trials. Blackwell Publishing Ltd; 2018. p. 1716-.
18. Michie S, Ashford S, Sniehotta FF, Dombrowski SU, Bishop A, French DP. A refined taxonomy of behaviour change techniques to help people change their physical activity and healthy eating behaviours: The CALO-RE taxonomy. Psychology and Health. 2011;26(11):1479-98.
19. Michie S, van Stralen MM, West R. The behaviour change wheel: A new method for characterising and designing behaviour change interventions. Implementation Science. 2011;6(1):42-.
20. Dyson PA, Twenefour D, Breen C, Duncan A, Elvin E, Goff L, et al. Diabetes UK evidence-based nutrition guidelines for the prevention and management of diabetes. Diabetic Medicine. 2018;35(5):541-7.
21. Al Slamah T, Nicholl BI, Alslail FY, Melville CA. Self-management of type 2 diabetes in gulf cooperation council countries: A systematic review. PLOS ONE. 2017;12(12):e0189160-e.

For peer review only

22. Cocks K, Torgerson DJ. Sample size calculations for pilot randomized trials: A confidence interval approach. Journal of Clinical Epidemiology. 2013;66(2):197-201.
23. Julious SA. Sample size of 12 per group rule of thumb for a pilot study. Pharmaceutical Statistics. 2005;4(4):287-91.
24. Braun V, Clarke V. Using thematic analysis in psychology. Qualitative Research in Psychology. 2006;3(2):77-101.
25. McCrudden MT, Marchand G, Schutz PA. Joint displays for mixed methods research in psychology. Methods in Psychology. 2021;5:100067.
26. Morgan DL. Commentary—After triangulation, what next? : Sage Publications Sage CA: Los Angeles, CA; 2019. p. 6-11.
27. Creswell JW, Clark VLP. Designing and conducting mixed methods research: Sage publications; 2017.
28. Eldridge SM, Chan CL, Campbell MJ, Bond CM, Hopewell S, Thabane L, et al. CONSORT 2010 statement: extension to randomised pilot and feasibility trials. bmj. 2016;355.
29. Moore GF, Audrey S, Barker M, Bond L, Bonell C, Hardeman W, et al. Process evaluation of complex interventions: Medical Research Council guidance. BMJ (Online). 2015;350.
30. BOOK QC. Stanford Patient Education Research Center. American Journal of Public Health. 1990;80:446-52.
31. Toobert DJ, Hampson SE, Glasgow RE. The summary of diabetes self-care activities measure: Results from 7 studies and a revised scale. Diabetes Care. 2000;23(7):943-50.
32. Association AD. Understanding A1C Diagnosis: ADA; 2022 [Available from: https://diabetes.org/diabetes/a1c/diagnosis.
33. Avery KN, Williamson PR, Gamble C, Francischetto EOC, Metcalfe C, Davidson P, et al. Informing efficient randomised controlled trials: exploration of challenges in developing progression criteria for internal pilot studies. BMJ open. 2017;7(2):e013537.
34. Kim MT, Kim KB, Huh B, Nguyen T, Han H-R, Bone LR, et al. The Effect of a Community-Based Self-Help Intervention. American Journal of Preventive Medicine. 2015;49(5).
35. Cho J-H, Kwon H-S, Kim H-S, Oh J-A, Yoon K-H. Effects on diabetes management of a health-care provider mediated, remote coaching system via a PDA-type glucometer and the Internet. Journal of Telemedicine and Telecare. 2011;17(7).
36. Basak Cinar A, Schou L. Health promotion for patients with diabetes: health coaching or formal health education? International Dental Journal. 2014;64(1).
37. Almulhim AN, Hartley H, Norman P, Caton SJ, Doğru OC, Goyder E. Behavioural Change Techniques in Health Coaching-Based Interventions for Type 2 Diabetes: A Systematic Review and Meta-Analysis. BMC Public Health. 2023;23(1):1-21.
38. Lee EC, Whitehead AL, Jacques RM, Julious SA. The statistical interpretation of pilot trials: should significance thresholds be reconsidered? BMC medical research methodology. 2014;14(1):1-8.
39. Schulz KF, Altman DG, Moher D. CONSORT 2010 Statement: Updated guidelines for reporting parallel group randomised trials. BMJ (Online). 2010;340(7748):698-702.

For peer review only

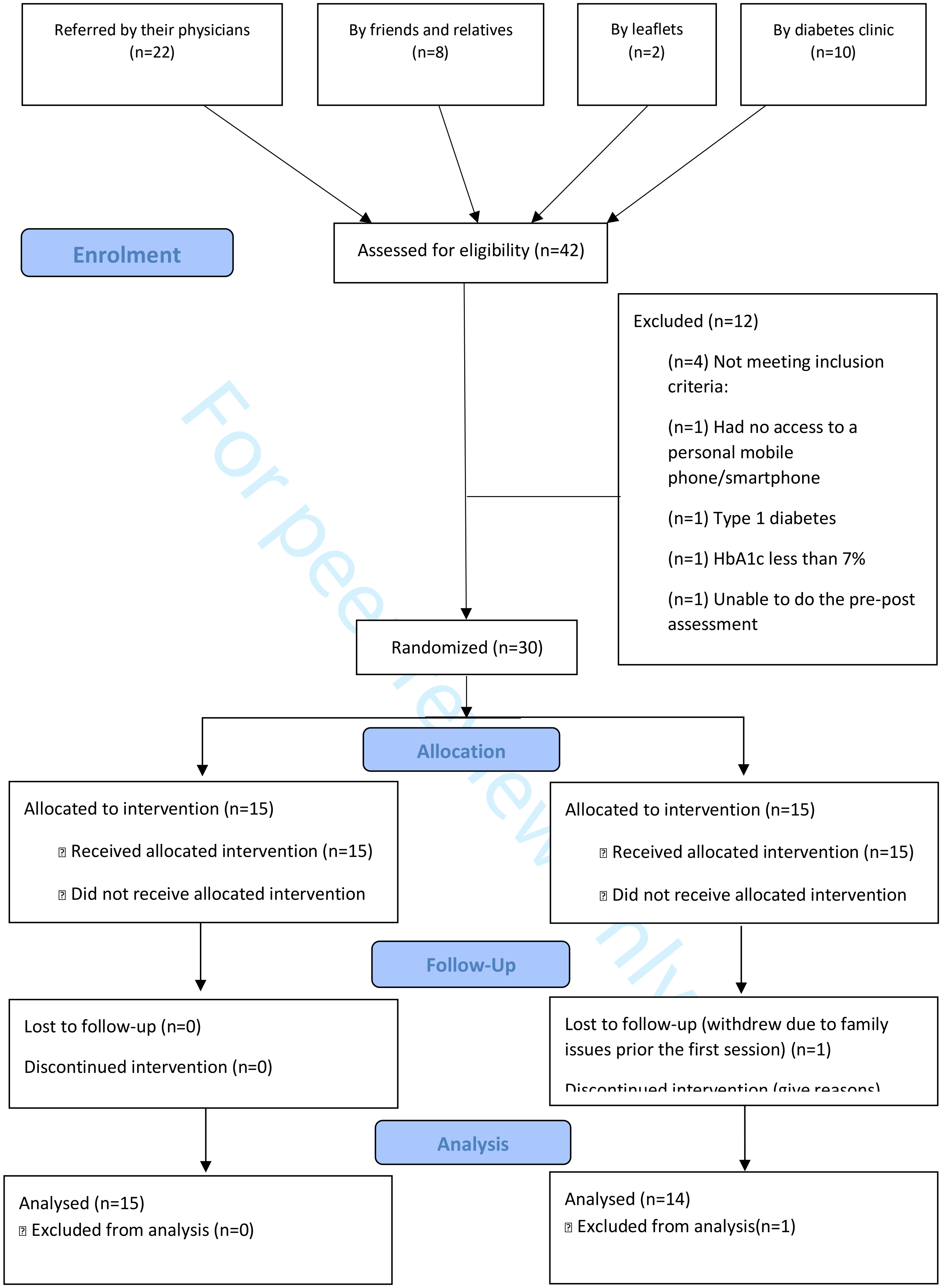

Figure 1: The study CONSORT flow chart

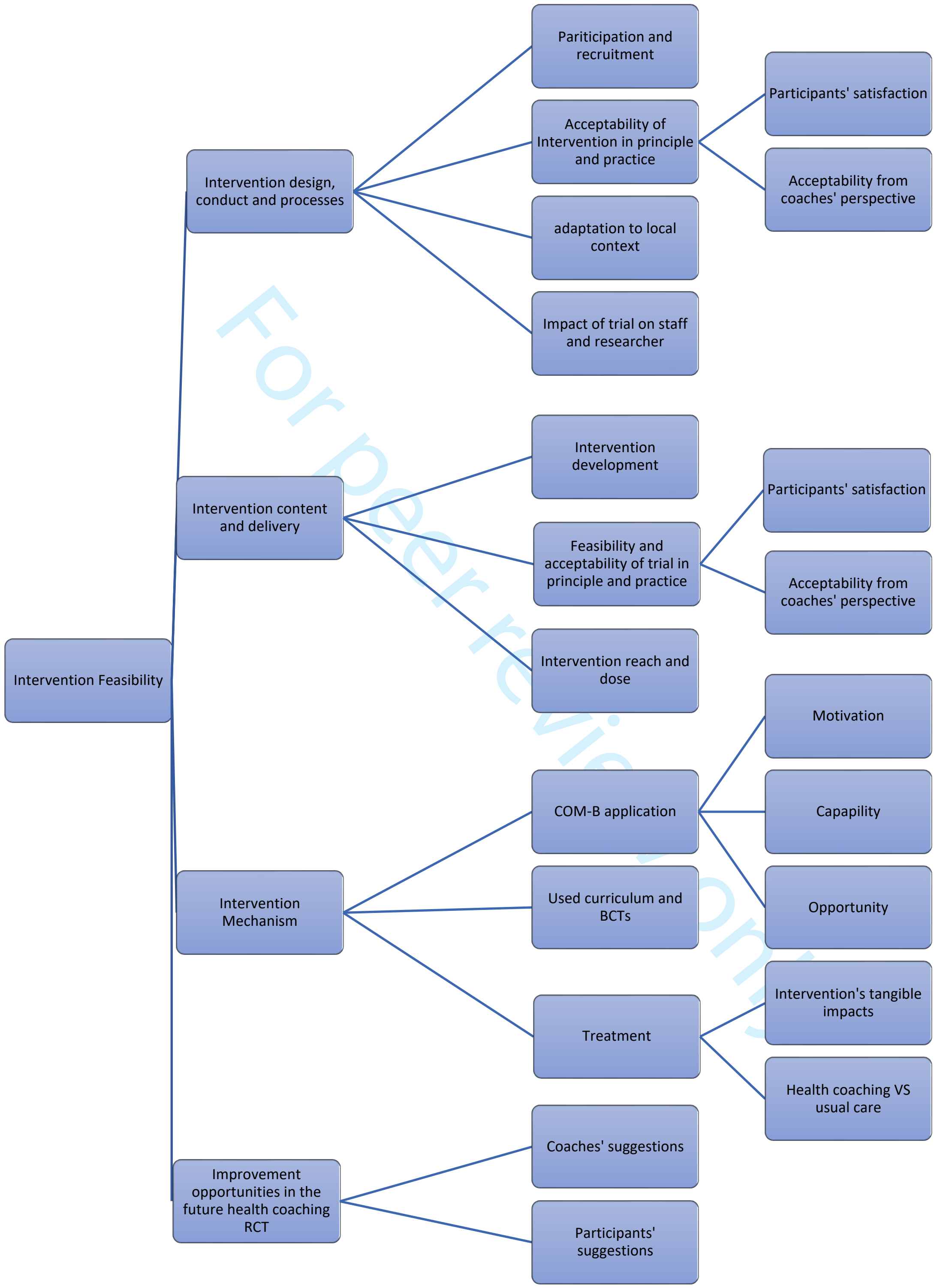

Figure 2: Thematic analysis map

For peer review only

CONSORT 2010 checklist of information to include when reporting a pilot or feasibility trial*

Section/Topic	Item No	Checklist item	Reported on page No
**Title and abstract**			
	1a	Identification as a pilot or feasibility randomised trial in the title	1
	1b	Structured summary of pilot trial design, methods, results, and conclusions (for specific guidance see CONSORT abstract extension for pilot trials)	1
**Introduction**			
Background and objectives	2a	Scientific background and explanation of rationale for future definitive trial, and reasons for randomised pilot trial	2
	2b	Specific objectives or research questions for pilot trial	3
**Methods**			
Trial design	3a	Description of pilot trial design (such as parallel, factorial) including allocation ratio	3
	3b	Important changes to methods after pilot trial commencement (such as eligibility criteria), with reasons	4
Participants	4a	Eligibility criteria for participants	4
	4b	Settings and locations where the data were collected	4
	4c	How participants were identified and consented	4
Interventions	5	The interventions for each group with sufficient details to allow replication, including how and when they were actually administered	4-5
Outcomes	6a	Completely defined prespecified assessments or measurements to address each pilot trial objective specified in 2b, including how and when they were assessed	5
	6b	Any changes to pilot trial assessments or measurements after the pilot trial commenced, with reasons	5
	6c	If applicable, prespecified criteria used to judge whether, or how, to proceed with future definitive trial	5
Sample size	7a	Rationale for numbers in the pilot trial	5
	7b	When applicable, explanation of any interim analyses and stopping guidelines	n/a
Randomisation:			
Sequence generation	8a	Method used to generate the random allocation sequence	4
	8b	Type of randomisation(s); details of any restriction (such as blocking and block size)	4
Allocation concealment mechanism	9	Mechanism used to implement the random allocation sequence (such as sequentially numbered containers), describing any steps taken to conceal the sequence until interventions were assigned	4

For peer review only - http://bmjopen.bmj.com/site/about/guidelines.xhtml

Implementation	10	Who generated the random allocation sequence, who enrolled participants, and who assigned participants to interventions	4
Blinding	11a	If done, who was blinded after assignment to interventions (for example, participants, care providers, those assessing outcomes) and how	n/a
	11b	If relevant, description of the similarity of interventions	n/a
Statistical methods	12	Methods used to address each pilot trial objective whether qualitative or quantitative	6
**Results**			
Participant flow (a diagram is strongly recommended)	13a	For each group, the numbers of participants who were approached and/or assessed for eligibility, randomly assigned, received intended treatment, and were assessed for each objective	9
	13b	For each group, losses and exclusions after randomisation, together with reasons	9-10
Recruitment	14a	Dates defining the periods of recruitment and follow-up	9
	14b	Why the pilot trial ended or was stopped	n/a
Baseline data	15	A table showing baseline demographic and clinical characteristics for each group	8
Numbers analysed	16	For each objective, number of participants (denominator) included in each analysis. If relevant, these numbers should be by randomised group	10
Outcomes and estimation	17	For each objective, results including expressions of uncertainty (such as 95% confidence interval) for any estimates. If relevant, these results should be by randomised group	19-20
Ancillary analyses	18	Results of any other analyses performed that could be used to inform the future definitive trial	13
Harms	19	All important harms or unintended effects in each group (for specific guidance see CONSORT for harms)	n/a
	19a	If relevant, other important unintended consequences	n/a
**Discussion**			
Limitations	20	Pilot trial limitations, addressing sources of potential bias and remaining uncertainty about feasibility	24
Generalisability	21	Generalisability (applicability) of pilot trial methods and findings to future definitive trial and other studies	24
Interpretation	22	Interpretation consistent with pilot trial objectives and findings, balancing potential benefits and harms, and considering other relevant evidence	24
	22a	Implications for progression from pilot to future definitive trial, including any proposed amendments	23-24
**Other information**			
Registration	23	Registration number for pilot trial and name of trial registry	n/a
Protocol	24	Where the pilot trial protocol can be accessed, if available	5
Funding	25	Sources of funding and other support (such as supply of drugs), role of funders	n/a
	26	Ethical approval or approval by research review committee, confirmed with reference number	26

For peer review only - http://bmjopen.bmj.com/site/about/guidelines.xhtml

Citation: Eldridge SM, Chan CL, Campbell MJ, Bond CM, Hopewell S, Thabane L, et al. CONSORT 2010 statement: extension to randomised pilot and feasibility trials. BMJ. 2016;355. This is an Open Access article distributed in accordance with the terms of the Creative Commons Attribution (CC BY 3.0) license (http://creativecommons.org/licenses/by/3.0/), which permits others to distribute, remix, adapt and build upon this work, for commercial use, provided the original work is properly cited.

*We strongly recommend reading this statement in conjunction with the CONSORT 2010, extension to randomised pilot and feasibility trials, Explanation and Elaboration for important clarifications on all the items. If relevant, we also recommend reading CONSORT extensions for cluster randomised trials, non-inferiority and equivalence trials, non-pharmacological treatments, herbal interventions, and pragmatic trials. Additional extensions are forthcoming: for those and for up-to-date references relevant to this checklist, see www.consort-statement.org.

For peer review only

For peer review only - http://bmjopen.bmj.com/site/about/guidelines.xhtml

BMJ Open

Feasibility and Acceptability of Tailored Health Coaching Intervention to Improve Type 2 Diabetes Self-Management in Saudi Arabia: A Mixed-Methods Randomised Feasibility Trial

Journal:	*BMJ Open*
Manuscript ID	bmjopen-2023-078631.R2
Article Type:	Original research
Date Submitted by the Author:	19-Mar-2024

Complete List of Authors:	Almulhim, Abdullah; The University of Sheffield, School of Medicine and Population Health; College of Health Sciences Saudi Electronic University<br>Alhowaish, Atheer; King Fahad Medical City<br>Madani, Alaa; King Fahad Medical City<br>AlQaddan, Munirah; King Fahad Medical City<br>Altuwalah, Abdulaziz S.; King Fahad Medical City<br>Goyder, Elizabeth; The University of Sheffield, School of Medicine and Population Health

**Primary Subject Heading**:	Public health
Secondary Subject Heading:	Public health
Keywords:	PUBLIC HEALTH, DIABETES & ENDOCRINOLOGY, Behavior

SCHOLARONE™ Manuscripts

I, the Submitting Author has the right to grant and does grant on behalf of all authors of the Work (as defined in the below author licence), an exclusive licence and/or a non-exclusive licence for contributions from authors who are: i) UK Crown employees; ii) where BMJ has agreed a CC-BY licence shall apply, and/or iii) in accordance with the terms applicable for US Federal Government officers or employees acting as part of their official duties; on a worldwide, perpetual, irrevocable, royalty-free basis to BMJ Publishing Group Ltd ("BMJ") its licensees and where the relevant Journal is co-owned by BMJ to the co-owners of the Journal, to publish the Work in this journal and any other BMJ products and to exploit all rights, as set out in our licence.

The Submitting Author accepts and understands that any supply made under these terms is made by BMJ to the Submitting Author unless you are acting as an employee on behalf of your employer or a postgraduate student of an affiliated institution which is paying any applicable article publishing charge ("APC") for Open Access articles. Where the Submitting Author wishes to make the Work available on an Open Access basis (and intends to pay the relevant APC), the terms of reuse of such Open Access shall be governed by a Creative Commons licence – details of these licences and which Creative Commons licence will apply to this Work are set out in our licence referred to above.

Other than as permitted in any relevant BMJ Author's Self Archiving Policies, I confirm this Work has not been accepted for publication elsewhere, is not being considered for publication elsewhere and does not duplicate material already published. I confirm all authors consent to publication of this Work and authorise the granting of this licence.

For peer review only

Feasibility and Acceptability of Tailored Health Coaching Intervention to Improve Type 2 Diabetes Self-Management in Saudi Arabia: A Mixed-Methods Randomised Feasibility Trial

Abdullah N. Almulhim[1,2]*, Atheer Alhowaish[3], Alaa Madani[3], Munirah AlQaddan[3], Abdulaziz S. Altuwalah[3], Elizabeth Goyder[1]

1 School of Medicine and Population Health, University of Sheffield, Sheffield S1 4DA, UK

2 Public Health Department, College of Health Sciences, Saudi Electronic University, Riyadh 13316, Saudi Arabia

3 Health Education Administration, King Fahad Medical City, Riyadh 12231, Saudi Arabia

* Correspondence: aalmulhim@seu.edu.sa

For peer review only

Abstract

Background: Around a third of the population of Saudi Arabia have been diagnosed with Type 2 diabetes, a condition often requiring lifestyle changes. Personalised health coaching, a strategy developed to assist individuals in overcoming challenges to adopt healthy behaviours, has not yet been widely applied in the country.

Aims: We aim to explore the feasibility and acceptability of tailored health coaching in Saudi Arabia, in order to help those with Type 2 diabetes to more effectively manage their condition.

Methods: Using a mixed-methods approach, this research involved a randomised controlled trial with 30 Saudi adults who have Type 2 diabetes. They were randomly allocated into either the intervention or control arm for 12 weeks. The Capability, Opportunity, Motivation, and Behaviour framework was used to guide the intervention implementation along with the Behaviour Change Techniques Taxonomy version 1. The primary goal was to assess the suitability and duration of the intervention, recruitment, retention, and completion rates. The secondary outcome focused on the preliminary efficacy of the health coaching measured by the glycaemic index, blood pressure, body mass index, waist circumference, weight, patient self-efficacy, and diabetes self-management.

Results: The results showed high rates of eligibility, recruitment, and retention (a screening rate of 90%, a recruiting rate of 79%, and a retention rate of 97%). Notable improvements were observed in the health coaching group across five outcomes: HbA1c, BMI, waist circumference, patient self-efficacy, and diabetes self-care. Qualitative findings highlighted the participants' perceived benefits from the intervention, including enhanced motivation, better understanding of diabetes management, and a supportive coaching relationship. Participants expressed high satisfaction with the intervention and advocated for its expansion.

Conclusion: The findings demonstrated positive outcomes, supporting the need for a larger randomised controlled trial to evaluate the efficacy of health coaching in improving diabetes self-management among individuals with Type 2 diabetes in Saudi Arabia.

Keywords: health behaviour change; health coaching; self-management; behaviour change techniques; type 2 diabetes

Summary Box:

Strengths and limitations of this study:

- First health coaching intervention in Saudi Arabia.
- First use of the COM-B model and BCTs taxonomy in a health coaching intervention
- Adherence to CONSORT 2010 progression criteria.
- Comprehensive impact assessment through mixed methods.
- Limited generalizability due to specific settings and demographics.

Introduction:

Type 2 Diabetes Mellitus (T2DM) is a chronic illness that is becoming more common globally and represents a significant public health concern. Lifestyle factors play a crucial role in the development of this disease, with obesity often manifesting as a symptom of other lifestyle factors such as physical inactivity (1). T2DM can lead to complications such as cardiovascular disease, kidney failure, nerve damage, and ocular and auditory problems, significantly increasing the risk of heart disease and stroke in people with diabetes (2).

Managing diabetes can be a challenging task that encompasses various aspects of life, including social, economic, and healthcare domains (3). To help T2DM patients effectively manage their condition, prioritising self-management is crucial (4). T2DM is a prevalent chronic disease in Saudi Arabia (SA), with one of the highest rates in the Middle East and the seventh-highest globally (5). Unhealthy diets, inactive lifestyles, and rapid urbanisation are all contributing factors to this concerning trend (6).

Despite national efforts to control T2DM in the SA context, the disease continues to pose significant challenges (7). While awareness campaigns are being employed, there is a notable difficulty among most T2DM patients in adopting healthier diets and lifestyles (7). To address this issue, it is necessary to implement patient-centred approaches that prioritise the development of self-management skills rather than relying solely on education-based programs (8).

Health coaching, derived from various disciplines and behaviour change theories, has emerged as a client-centred approach gaining recognition in the field of health promotion. Although it has been widely explored in the literature and has shown promising results, this personalised one-on-one intervention has not yet been implemented within the healthcare systems of SA. Studies have demonstrated that health coaching, centred around patients' values, may offer additional benefits compared to traditional T2DM education programs (9). By focusing on enhancing patients' self-efficacy and essential skills such as goal-setting and problem-solving, health coaching can prove highly effective and financially viable (10). Its successful application in diverse contexts has shown it to be a valuable tool in improving diabetes self-management (11, 12).

For peer review only

However, the effectiveness of existing health coaching interventions exhibits inconsistency due to variations in methodology, duration, and intervention content (11). Recent systematic reviews of randomised controlled trials employing health coaching have reported mixed results, with some studies suggesting its effectiveness while others claim it to be ineffective (11, 12). There is no agreed-upon health coaching intervention model, which creates uncertainty regarding the most effective strategies to adopt, particularly in the SA context. The inconsistent findings across studies may be due to the lack of agreement on the active ingredients and content of successful health coaching interventions (13). Additionally, cultural differences between SA and Western countries highlight the need for tailored interventions to ensure that proposed health coaching models are appropriate and relevant (14).

This feasibility study aims to assess the usability and acceptability of a health coaching intervention for T2DM self-management in SA, with the ultimate goal of reducing HbA1c levels and informing a future large-scale randomised controlled trial (RCT).

Materials and Methods

This research was conducted in accordance with the Consolidated Standards of Reporting Trials (CONSORT) extension guidelines specific to pilot and feasibility randomised controlled trials (RCTs) (15). A comprehensive protocol for this study has already been published (16).

Design

This is a randomised two-arm feasibility trial evaluating a 3-month intervention. The study adopted a mixed-methods approach, enabling the collection of both quantitative and qualitative data. An RCT design to address both evaluate the feasibility of RCT methods for recruitment, delivery, and data collection, as well as a valid comparison of health coaching's effectiveness between the groups (17). Integral to our approach was the implementation of the Behaviour Change Wheel (BCW) framework, used in adapting the health coaching intervention for the SA context. The BCW informed our intervention design in several key ways: Firstly, by identifying suitable intervention functions from the Capability, Opportunity, Motivation-Behaviour (COM-B) model, we tailored the health coaching to the specific needs and barriers of our target population. Secondly, we utilized the Behaviour Change Technique Taxonomy version 1 (BCTTv1) to code the active ingredients of the intervention, mapping these to the BCW framework for alignment with established behaviour change principles (18). Furthermore, a behavioural analysis, guided by the Theoretical Domains Framework (TDF) functions and behavioural change techniques (BCTs), was conducted to address pre-identified barriers (see Supplemental Table 1). This analysis led to the selection of 36 relevant BCTs, which were mapped to directly target the behavioural barriers in adults with poorly managed T2DM, our intervention's target population (see Supplemental Table 2). By integrating the BCW, we were able to define the problem behaviours and target behaviours for intervention, ensuring a comprehensive and theory-driven approach as advised by BCW guidance (19). Further information on the intervention can be found in a prior publication (16).

Patient and public involvement

Patients and the public were not involved during the process of this research.

For peer review only - http://bmjopen.bmj.com/site/about/guidelines.xhtml

Intervention Development

There have been various identified target behaviours that would result in the adoption of a healthy lifestyle and diet as recommended by UK lifestyle guidelines.(20) As of now, there are no evidence-based guidelines in SA for T2DM to adopt a healthy lifestyle and diet. Therefore, we have referred to the UK's guidelines on lifestyle as they share similarities in their healthcare systems. (20, 21) These guidelines have been used to determine the most suitable behaviour targets for the Saudi context. We consulted healthcare professionals in the intervention setting, including a dietician, to establish these targets. Through collaborative discussions, we assessed and prioritised behaviours based on their potential impact, the feasibility of measurement, and achievability. As a result of this process, we have proposed four behaviour targets. (18)

The four general behaviours targets of the intervention were: (20)

- Decrease carbohydrate intake for each meal
- Use unsaturated fats as possible (avoid saturated fats)
- Do exercise for 30 min five days on a weekly basis
- Monitor waist circumference, and maintain it below (80 cm for women and 94 cm for men)

The aim was to gradually reduce carbohydrate intake while ensuring that participants met their nutritional requirements. The specific target amounts were communicated to participants during individualized dietary counselling sessions with health coaches, and participants were provided with guidance and meal plans to help them achieve and maintain the desired carbohydrate intake levels. The health coaching intervention used these targets as the benchmarks to track the progress and changes that the study population would make over three months.

We utilised diverse instruments, including questionnaires, interviews, focus groups, and clinical measures, to assess the intervention's feasibility, acceptability, and preliminary impact at two points: baseline and three months post-intervention. To quantitatively evaluate the intervention's preliminary effects, we measured changes in clinical outcomes such as HbA1c, BMI, waist circumference, and weight. These changes were analysed using linear regression to determine the mean differences between the intervention and control groups, with adjustments for baseline levels. Ethical clearance for the study was granted by the University of Sheffield and the King Fahad Medical City's Institutional Review Board (IRB Log Number: 21-062E).

Participant Recruitment

Inclusion criteria were: aged over 18, Haemoglobin A1c level of 7.0% or higher, able to read and understand Arabic, have access to a personal mobile phone, be willing to complete the intervention period, be willing to stay in Riyadh (the capital city of Saudi Arabia), and be able to read, understand, and sign the informed consent form.

For peer review only - http://bmjopen.bmj.com/site/about/guidelines.xhtml

The recruitment process involved advertising the intervention through multiple channels, including posters, brochures, social media, and healthcare provider referrals. Interested and eligible individuals met the research team for a brief study overview and questionnaire completion, providing demographic data and baseline laboratory reports.

Participants were randomly assigned to one of two groups in a 1:1 ratio by an independent individual using a computer-generated random numbers system in Statistical Package for the Social Sciences (SPSS), ensuring equal allocation chances. The two groups consisted of the intervention group, which received the health coaching intervention, and the control group, which received standard care.

Intervention Delivery

The health coaching intervention was completed in 12 weeks. A detailed intervention curriculum was used in delivering the intervention activities toward achieving the four target behaviours (see Supplemental Table 3).

Three health coaches, AA, AM, and MA, who were qualified through the Saudi Ministry of Health, conducted the intervention sessions. They received training in biweekly face to face (F2F) meetings over six weeks on the intervention curriculum. A workbook was provided as a guide to help them deliver coaching sessions as planned. A detailed explanation has been published elsewhere (16). It is important to note that participants did not directly receive or review the intervention curriculum. Our approach involved ensuring uniformity in intervention procedures by providing health coaches with the curriculum. Each coach was asked to document the BCTs they use in each session. Different BCTs were reported in each session. Each participant was assigned to one of three coaches at the baseline. Each participant received six sessions in total, which were delivered biweekly via video meetings and telephone coaching over three months. Due to the COVID-19 pandemic, the first and last meetings were conducted via video call instead of face-to-face as planned, while the rest were via telephone. Participants were encouraged to contact their coaches via WhatsApp between sessions if they had any related concerns. Eighty-four sessions were undertaken for all participants in the intervention group.

Usual Care (Control Group)

The participants in the control group were provided with the standard care, which involved receiving written materials and brochures on diabetes to increase awareness and promote the advantages of making changes to their health behaviours. Typically, individuals with T2DM have regular appointments with endocrine specialists to monitor their diabetes management. During these visits, the primary objective is to assess if any adjustments are necessary regarding the patient's medication, such as replacement, dosage increase, or maintaining the current prescription.

Data Collection

Baseline data collection included: demographic information, HbA1c, blood pressure, BMI, weight, and waist circumference measurements. Subsequently, we asked participants to complete two electronic questionnaires: the Diabetes Self-Care Activities (SDSCA) scale and

For peer review only

the Self-efficacy Scale for Diabetes. The post-intervention data collection replicated the initial session, with repeat measurements of HbA1c, blood pressure, Body mass index (BMI), weight, and waist circumference. Participants were also required to fill out the two initial questionnaires again, along with a Satisfaction Questionnaire designed specifically for the intervention group.

Reach and Retention

As per the study protocol (16), this study aimed to recruit a minimum of 9% (n = 30) of the required sample size for conducting a complete trial (22), Each group was expected to have a minimum of 12 participants to ensure meaningful data interpretation for informing a future definitive randomized controlled trial (23). A total of 24 participants in each group would be required to detect a meaningful clinical difference in HbA1c. The retention rate was evaluated based on the predefined progression criteria described elsewhere (16), with successful retention defined as achieving an 83% retention rate at the intervention endpoint.

Fidelity Assessment

All coaching sessions audio recorded and transcribed. An Arabic native speaker reviewed the entire transcription for accuracy. To ensure consistency and validity, a 10% sample of the transcription (approximately 50 minutes) was translated from Arabic to English, backtranslated, and then examined by a peer reviewer. The data, including coaching sessions, time spent in each session, semi-structured interviews, focus groups, and questionnaires, was meticulously stored and managed for quality control. Specifically, all data were entered and stored in Microsoft Excel twice, independently by two research team members. This double-entry process served as a precautionary measure to minimize data entry errors and enhance the accuracy and reliability of the data collected throughout the trial. Adherence to the intervention was defined by the number of participants who completed 5 out of six coaching sessions ≥ 84%.

Feasibility

Evaluation of feasibility included: participant eligibility and recruitment, retention, data collection adherence, and adherence to the intervention. We recorded participants' views about their engagement, capturing their reasons for interest, active participation, and lack of interest. We tracked and logged the number and percentage of those who were eligible and interested, those who completed the intervention, and those who withdrew to provide insight into the study's recruitment efficiency and participant retention

Acceptability

Acceptability of the intervention and its implementation was assessed using qualitative and quantitative data. The aim was to detect any potential challenges within the methodology used to deliver both the trial and the intervention. We conducted post-intervention semi-structured qualitative interviews and focus groups. The intervention's acceptability was primarily gauged through a Likert-scale Satisfaction Questionnaire (14 items) applied to the intervention group (n=14). This questionnaire, originally devised by the Dan Abrahams Healthy Living Centre and subsequently employed by DeJesus et al. (2018), incorporates a variety of question styles

For peer review only - http://bmjopen.bmj.com/site/about/guidelines.xhtml

(open & closed). Using a Likert scale, it offered a response range from 0 (not at all) to 10 (very much) to capture participant feedback. Given that this scale had not been used in Arabic studies before, we translated it into Arabic and pretested it on a smaller subset before implementing it for the full study sample. In addition to quantitative measures, our mixed-methods approach involved semi-structured interviews and focus groups to qualitatively assess the acceptability of the health coaching intervention. Thematic analysis was used to identify and interpret patterns within the qualitative data, aiming to understand the perceived benefits, challenges, and overall acceptability of the intervention from the participants' perspective.

Data Analysis

We conducted both quantitative and qualitative analyses. For the quantitative part, descriptive analysis was undertaken including screening and recruitment rates, retention rates, adherence to coaching sessions, and recruitment duration. We evaluated the use of BCTs and interactions between participants and coaches during these sessions. A Satisfaction Questionnaire captured participants' experiences, while changes in diabetes self-management and patient self-efficacy were assessed at the beginning and end of the study. The data were entered into Excel and subsequently transferred to SPSS and Stata Statistical Software (STATA) for advanced analysis, which included a linear regression to estimate the mean differences in outcomes.

For the qualitative aspect of our study, purposive sampling was employed to ensure data saturation in conducting interviews and focus groups. We conducted a total of 14 semi-structured interviews and two focus groups, engaging in detailed discussions with participants. The qualitative data were analysed using NVivo software through a reflexive thematic analysis process, adhering to six systematic phases: familiarising ourselves with the data, coding, generating themes, revising themes, defining, and finally reporting (24). During this process, consensus was reached through regular team meetings and discussions to ensure accuracy and comprehensiveness in the identification and categorization of themes.

In addition, we integrated the qualitative and quantitative data for comprehensive understanding (25), Using a joint display table (see Supplemental Table 4) (26), we compared and combined these data types. The display helped us identify areas of agreement (convergence), disagreement (divergence), and enhancement (expansion) between the two data types (27). In the integrated data, we denoted convergence with "=", complementarity with "+", and inconsistencies with "≠".

Although this feasibility study may not conclusively determine intervention effects, it provides valuable insights for estimating the sample size for a larger trial based on the mean difference and standard deviation of HbA1c.

Results:

Participant Characteristics

The control group (mean age=53.40, SD=8.47) was slightly older than the intervention group (mean age=52, SD=8.32). The majority were married, with monthly incomes ranging from less than 5000 SR (28.6%) to more than 15000 SR (7.1%); 50.0% chose not to declare. About a quarter had completed high school, and an equal proportion had primary education or were

For peer review only - http://bmjopen.bmj.com/site/about/guidelines.xhtml

literate without formal education. Approximately 31% had lived with T2DM for over ten years, with 24.1% of the intervention group having T2DM for 1-3 years. The majority (89.7%) were on diabetes medications. Table 1 summarises the demographic characteristics of both groups.

Table 1: Summary of the participants' demographic characteristics

For peer review only

		Intervention group		Control group	
		Total N (%)	Median (IQR)	Total N (%)	Median (IQR)
Gender	Male	6 (42.9%)		7 (46.7%)	
	Female	8 (57.1%)		8 (53.3%)	
Age	Year	14	54.5 (44.5 to 59.25)	15	54 (50 to 60)
Marital status	Married	14 (100.0%)		14 (93.3%)	
	Single	0 (0.0%)		1 (6.7%)	
Monthly income	less than 5000 (SR)	4 (28.6%)		7 (46.7%)	
	5000-10000 (SR)	2 (14.3%)		0 (0.0%)	
	10000-15000 (SR)	0 (0.0%)		2 (13.3%)	
	more than 15000 (SR)	1 (7.1%)		1 (6.7%)	
	Prefer not to declare	7 (50.0%)		5 (33.3%)	
Education level	Illiterate	0 (0.0%)		0 (0.0%)	
	Primary school	3 (21.4%)		3 (20.0%)	
	Secondary school	2 (14.3%)		2 (13.3%)	
	High school	3 (21.4%)		4 (26.7%)	
	Bachelor's degree	2 (14.3%)		3 (20.0%)	
	Diploma	1 (7.1%)		0 (0.0%)	
	Can read and write	3 (21.4%)		3 (20.0%)	
Since when you were diagnosed with Type 2 diabetes	less than a year	2 (14.3%)		0 (0.0%)	
	1-3	6 (42.9%)		0 (0.0%)	
	3-5	1 (7.1%)		2 (13.3%)	
	5-7	0 (0.0%)		1 (6.7%)	
	7-10	1 (7.1%)		6 (40.0%)	
	more than ten years	3 (21.4%)		6 (40.0%)	
	do not know	1 (7.1%)		0 (0.0%)	
Do you use diabetes medications ?	Yes	12 (85.7%)		14 (93.3%)	
	No	2 (14.3%)		1 (6.7%)	

For peer review only

Feasibility of the Intervention

Eligibility and Recruitment

The recruitment process took about five weeks, starting on May 1st, 2021, to June 5th, 2021. Leaflets were used for advertising the intervention and were distributed in different places in the hospital, including in waiting areas and on hospital wall notice boards. In addition, we met physicians in-person to introduce the intervention and gave them leaflets with more details to encourage their patients to join in the study.

Of the 42 potential participants initially identified and assessed for eligibility, 38 met the study criteria, resulting in a screening rate of approximately 90%. These 42 individuals were referred by different sources. Twenty-two were referred by their doctors, while the others were recruited through various methods. Two were from leaflets, eight were from suggestions by friends or relatives, and ten were directly engaged at the diabetes clinic. Twelve were excluded, of which four were ineligible because they did not meet the intervention inclusion criteria for the following reasons: one had no access to a personal mobile phone/smartphone, one patient was diagnosed with T1DM, one had A1c below 7%, and one was unable to do pre-and post-assessments. Eight were excluded for other reasons; two did not respond, and six for different reasons, e.g., being too busy. Thirty eligible patients were recruited to take part in the intervention. Out of the 38 eligible patients, 30 were successfully recruited and consented to participate in the intervention, yielding a recruitment rate of approximately 79%. All of them completed baseline assessments and questionnaires, and then randomly, 15 patients were allocated to the intervention group, while the other 15 participants were assigned to the control group. One person who was part of the intervention group withdrew from the trial prior to the first session and was thus not included in the study (see Supplemental Figure 1 CONSORT diagram for more details).

Intervention Delivery

The intervention started with the first session on June 15th, 2021, and ended on September 23rd 2021. The average time spent per participant was between ~ 17.7 to 25.5 in each session. The total range time of all sessions per patient was 109-153 minutes, with an average of 120.8 (SD= 13.7). See Supplemental Table 5 for more details about the time duration of each session.

Retention of Participants

Thirty eligible participants consented to participate in the trial and completed the baseline assessment randomly allocated either to the coaching group (n=15) or the control group (n=15). Of those, all twenty-nine who started the intervention and took the first session remained till the endpoint and completed all the intervention activities. Only one participant allocated to the coaching group had withdrawn and discontinued the study before the first session (retention rate = 97%). All twenty-nine have completed the intervention endpoint assessments. The main progress criteria and feasibility measurements are summarised in Supplemental Table 6.

Adherence to the Coaching Sessions

Fifteen out of thirty participants were randomly allocated to the coaching group. Before the first session, one participant withdrew (P12) due to family issues. The rest of the fourteen have

For peer review only - http://bmjopen.bmj.com/site/about/guidelines.xhtml

completed all their coaching sessions (adherence rate 100%), which met the predetermined progression criteria of adhering to ≥ 84%. Half of the participants took their sessions at the planned time as previously scheduled. However, health coaches have rescheduled different sessions for seven participants for different reasons. The rescheduled sessions varied between 1 to 2 sessions for each one of them. Out of 84 sessions, only eleven sessions have been rescheduled (13%). The reasons for the rescheduling were home/work/appointment conflicts (n = 6 sessions), travel (n=1 session), illness (n=3 sessions), and being busy (n=1 session). All participants started their coaching sessions at the same time on June 15th, 2021, except four participants who started later on July 1st, 2021. A Fifteen-day delay because their coach could not begin at that time for family issues. In addition, the same four participants had another delay between the fourth and fifth sessions due to the coach's college exams. This led them to finish their last session on September 23rd, 2021.

Data Collection Adherence:

Participants recruited for the study were invited to Al-Zulfi General Hospital on June 10th, 2021. During this visit, they were given consent forms and information sheets about the intervention, providing them with an opportunity to ask questions and discuss any concerns related to their participation. Furthermore, baseline data, including measures of HbA1c, blood pressure, BMI, weight, and waist circumference, were collected during this visit. Due to the restrictions imposed by COVID-19, all required measurements and paperwork were completed in a single visit. Participants were then asked to complete two electronic questionnaires, the Diabetes Self-Care Activities (SDSCA) scale, and the Self-efficacy Scale for Diabetes, during the hospital visit on June 10th, 2021. The baseline data collection was successfully completed by all 30 participants, yielding a 100% completion rate. The time taken to complete the questionnaires ranged from 7 to 12 minutes, with no participants reporting difficulties or issues in the completion process.

At the end of the intervention, participants were once again invited to the hospital to gather post-intervention data. This second data collection session was much like the first, where we again measured HbA1c, blood pressure, BMI, weight, and waist circumference. Participants were also asked to complete the initial two questionnaires, with the addition of a Likert-scale Satisfaction Questionnaire for the intervention group only. This additional questionnaire resulted in a slightly longer completion time, ranging from 9 to 15 minutes, due to the inclusion of open-ended questions and certain items requiring justifications or explanations for selected responses. Overall, all participants effectively completed the data collection process, demonstrating successful adherence to our study's data collection procedures.

Progression Criteria

The feasibility measurements and predetermined progression criteria yielded positive results. The screening rate was 90%, exceeding the target of 80%, suggesting proceeding to the future definitive RCT. The recruitment rate reached 79%, demonstrating robust engagement with the target population. The retention rate at three months was 97%, surpassing the required 83% rate. Baseline data collection adherence and intervention adherence both achieved 100%. Endpoint data collection adherence was 97.6%, with only one participant withdrawing before

For peer review only - http://bmjopen.bmj.com/site/about/guidelines.xhtml

the first session. Overall, these results support the decision to proceed to the future definitive RCT, indicating the feasibility and acceptability of the health coaching approach for individuals with T2DM in SA.

Sample Size

The sample size for this feasibility study was determined in consultation with a statistician and was guided by practical considerations as recommended by the CONSORT guidelines for feasibility trials (28). Our aim was not to test the intervention for statistical significance but rather to assess the feasibility and variability of outcomes to inform the planning of a definitive RCT. The choice of sample size was also influenced by the need to manage resources efficiently and to ensure a broad enough representation of the target population to capture initial insights into the intervention's acceptability and implementation.

Furthermore, the statistician advised that for the upcoming main trial, a sample size calculation should be based on a clinically significant mean difference of 0.5% and a standard deviation of 1.4, given the substantial effect size of -0.93 observed in this feasibility study. With these parameters, using a power of 0.8 and an alpha level of 0.05, we estimated that 125 participants per group would be necessary. This sample size would not only allow us to detect a clinically significant difference but also accommodate subgroup analyses to explore variations in effectiveness across different settings or patient groups.

Acceptability of the Intervention

Acceptability and suitability of the intervention were assessed through a questionnaire (Likert-scale Satisfaction Questionnaire, 14 items) for the intervention group (n = 14). Using a Likert scale of 0 (not at all) to 10, most participants' responses show that the intervention affected their behaviour "quite a bit " (mean: 8.2 (SD= 2.3)). Participants replied, “very much” (mean 9.2 (SD= 1.4)) when asked how much participation in the intervention helped them establish a personal vision of wellness. When asked how the intervention boosted their confidence in taking actions toward improved well-being, the overall response was "very much" (mean 9.2 (SD= 1.4)). Their motives for making efforts toward enhanced well-being were "quite a bit" (mean 8.3 (SD= 1)). The average response to the usage of goal setting was "very much" (mean 9.2. (SD= 0.9)). Whereas their mean response to their use of problem-solving abilities was "quite a bit" (mean 8.6 (SD= 1.4)). Participants shared that the intervention greatly assisted them in overcoming obstacles and achieving increased levels of wellness, with a high mean score of 9 (SD= 1.1) reported. From the first through the eighth question, participants' responses are presented in Supplemental Table 7.

Participants were asked three "Yes" or "No" questions, and they all replied "Yes" when asked whether they expected to continue making improvements (n=14, 100%). The majority (n=13, 92.9%) responded with "Yes" the intervention met their expectations. All 14 participants who were asked whether they would recommend the intervention to others responded with a positive "yes".

For peer review only

Thematic Analysis Findings

The thematic analysis of the qualitative interviews and focus groups underscored the intervention's positive reception, with emergent themes revealing enhanced self-efficacy, increased motivation, and the supportive dynamics of the health coaching relationship, which participants found to be a significant aspect of the intervention's acceptability. We analysed 1691 minutes of coaching sessions to identify the BCTs used (see Supplemental Table 8) and transcribed 497 minutes of focus groups and interviews. A native Arabic speaker confirmed the accuracy of the entire transcription. To further ensure validity, about 10% of the transcription (50 minutes) underwent a back-translation process checked by a professional native speaker. Throughout the intervention, the first author (A.N.A), acting as the researcher, maintained field notes. Due to COVID-19 limitations, all interviews and focus groups were conducted online.

Evaluation of Participants and Health Coaches' Experiences

Various qualitative methods were used to assess participant experiences during the intervention, including interviews, focus groups, and field notes. After the intervention, two focus groups took place. Participants were given time to understand the questions before engaging in interviews and focus groups, fostering a deeper discussion. The researcher (A.N.A) conducted all focus groups and interviews, enabling active interaction and understanding of participant perspectives.

Between June 15th and September 23rd, 2021, 14 semi-structured interviews were conducted with seven participants (4 males, three females) from the intervention group, both pre-and post-study. Participants predominantly had a high school education or less, and the median age was 59. Three had long-standing diabetes of over ten years, three had a recent diagnosis of less than three years, and one was uncertain of the duration. Six of the seven were on diabetes medication.

Two post-study focus groups were conducted online. One focus group comprised six participants (three males and three females) from the intervention group with a median age of 52.5 years. The majority had a high school education or less, and most were on diabetes medication with a mix of recent and long-term diagnoses. The demographic characteristics of the participants can be seen in Table 2.

Three female health coaches from KFMC in Riyadh formed the second focus group to share their intervention delivery experiences. All had health coaching backgrounds, with two holding master's degrees in health education. The session lasted 265 minutes.

Table 2: Participants' Demographic characteristics

		Qualitative interview participants (n=7)		Focus group participants (n=6)	
Demographic characteristics		Number	%	Number	%
Gender	Male	4	57.1	3	50
	Female	3	42.9	3	50

For peer review only

Age	Years	43 to 62	Median 59	34 to 60	Median 52.5
Marital status	Married	7	100	6	100
Monthly income	less than 5000	1	14.3	2	33.3
	5000-10000	1	14.3	1	16.7
	10000-15000	1	14.3	0	
	Prefer not to declare	4	57.1	3	50
Education level	Primary school	2	28.6	1	16.7
	Secondary school	2	28.6	1	16.7
	High school	2	28.6	3	50
	Bachelor's degree	1	14.3	1	16.7
Since when you were diagnosed with Type 2 diabetes	less than a year	2	28.6	1	16.7
	1-3	1	14.3	3	50
	more than ten years	3	42.9	1	16.7
	do not know	1	14.3	1	16.7
Do you use diabetes medications?	Yes	6	85.7	5	83.3
	No	1	14.3	1	16.7

We used thematic analysis with inductive and deductive coding to evaluate the trial's acceptability and feasibility. Open coding initially identified emergent themes, with deductive coding following, grounded in qualitative research guidelines and the Medical Research Council (MRC) framework (29).

The analysis of translated transcripts produced four key themes, two of which were predetermined. The first theme pertained to trial design, conduct, and processes, divided into four sub-themes covering different aspects of intervention implementation. The second theme focused on intervention content and delivery, consisting of sub-themes on intervention components, perceived consequences, feasibility and acceptability in practice, and reach and dose. The third theme, intervention mechanism, encompassed sub-themes around COM-B model application, use of BCTs and intervention curriculum, and intervention impact. The fourth theme, future RCT, captured participant and coach suggestions. The thematic map used in the analysis is outlined in Supplemental Figure 2.

Theme 1: Intervention Design, Conduct, and Processes

For peer review only - http://bmjopen.bmj.com/site/about/guidelines.xhtml

For peer review only

This theme primarily focuses on the implementation and design of the intervention, along with the processes involved. Recruitment challenges during the pandemic prompted a shift to remote settings. This transition not only made recruitment a challenging task but also stretched the recruitment period beyond the planned timeframe.

Notably, a health coach stated that the recruitment phase was complicated as clinics moved online., *"...the recruitment phase, it was complicated only because all clinics become online and we could not see people with type 2 diabetes come to the hospital as usual, so the chance of meeting those people was very rare."* (Focus Group with Coaches, FGC01, Female).

Misunderstandings about health coaching in SA arose from a lack of understanding and familiarity. This highlighted the need for improved communication with recruiters, as one health coach observed, *"...from my communication with recruiters, some of them did not understand, so participants may get a wrong idea"* (Focus Group with Coaches, FGC01, Female).

Limited hospital access due to public health guidelines complicated recruitment further. However, the suggestion to recruit from a secondary hospital made the recruitment process manageable. Participants and health coaches recommended physicians as intermediaries for patient recruitment due to the trust and rapport they have established with patients; as one of them stated, *".....I think one of the best and most reliable ways to reach and recruit patients is to start from their direct physicians by suggesting the program for patients and encourage them to join"* (Focus Group with Coaches, FGC02, Female).

Proposing clear communication about program expectations aimed to reduce dropout rates, with one participant suggesting to *"ask participants to sign a contract to commit and if you are hesitating or you have the intention to withdraw to tell us [referred to program provider] at the beginning to give the opportunity to someone who needs it"* (Focus Group with Participants, P03, Female).

Health coaches and participants expressed general satisfaction with the intervention's design, and they believed that it effectively accommodated the local context of Saudi society. One participant expressed, *"I'm very very very satisfied and I think the program is easy to follow and very acceptable as it gradually improves different skills which last with a patient after the program…it is completely different from other programs.."* (Interview with Participants, P13, Male).

Additional workload challenged health coaches, mixing intervention tasks with regular duties. The COVID-19 pandemic further complicated matters by requiring greater flexibility in scheduling coaching sessions, particularly for participants with children engaged in online learning. To alleviate these issues, health coaches proposed securing formal workplace approval to dedicate full-time to the intervention. As one health coach noted, *"As you know and because of the workload, I was thinking of withdrawing from the intervention...... I think it is imperative to work a full-time job as a health coach in the intervention, not as a volunteer, and get formal permission from my work on this.."* (Focus Group with Coaches, FGC01, Female).

For peer review only - http://bmjopen.bmj.com/site/about/guidelines.xhtml

Theme 2: Intervention Content and Delivery

This theme encompassed multiple sub-themes reflecting on the intervention's implementation, including its acceptability and adaptability within the Saudi context, potential unintended consequences, the feasibility of the practical application, and intervention reach and dosage.

Health coaches suggested modifications for better acceptability and suitability of the intervention to the Saudi context. Emphasising the need for professionalism and structure, they proposed using formal platforms for communication, as one coach stated, *"...communication with the participant should be through a formal platform or application... to make the program more formal and organised..."* (Focus Group with Coaches, C02, Female). They also suggested the importance of physical meetings or video calls for a more serious engagement, as expressed by another coach, *"I prefer to have a physical meeting where I can sit with the patient face-to-face…"* (Focus Group with Coaches, FGC01, Female).

Coaches highlighted the necessity of prepared private spaces for coaching sessions. to give a professional image to the intervention. As one coach expressed, *"..As a coach, there should be a certain prepared private place at work to conduct all of my coaching sessions.."* (Focus Group with Coaches, C02, Female).

Participants preferred visual coaching sessions over phone calls for effective communication. Coaches felt that seeing the participant was vital for effective communication and coaching. A health coach expressed this, saying, *"...visual interaction with participants is very very important, you know the importance of reading and understanding the body language for me as a coach.."* (Focus Group with Coaches, C03, Female).

Participants praised the intervention for its straightforwardness and effectiveness in helping them make progress towards their goals. Some suggested a desire for additional sessions to further adopt and maintain new habits. One participant commented, *"I think three months was enough"* (Interview with Participants, P03, Female), *while another remarked, "…it enabled me to stay committed, continue, and achieve my goals"* (Interview with Participants, P09, Male).

Participants appreciated the use of health coaching techniques, such as action plans and SMART goals, which helped set achievable targets and keep them motivated. The flexibility and graduality of the program were also well-received, as one participant mentioned, *"...the most thing I liked about the program was the graduality to achieve goals .."* (Interview with Participants, P13, Male).

Coaches confirmed the intervention content's acceptability and observed its positive impacts. They also suggested the addition of monthly sessions or an extension of the intervention to six months for better patient progress monitoring and maintenance. A coach stated, *"... I suggest the intervention last longer… I think it would be good if the intervention lasts six months with a session each month …"* (Focus Group with Coaches, FGC01, Female).

Theme 3: Intervention Mechanism

This theme emphasises the practical application and effects of the COM-B health coaching model. Coaches received specialised training on the curriculum, and BCTs, utilising several

For peer review only - http://bmjopen.bmj.com/site/about/guidelines.xhtml

tools for efficient delivery. Notably, coaches documented the BCTs employed in each session, enabling tailored strategies to target specific behaviours. Participants appreciated this approach, stating, *"The BCTs helped me during each session to address some behaviours…"* (Focus Group with Coaches, FGC01, Female).

The COM-B model played a crucial role in the intervention, targeting four behaviours. Participants noted marked improvements in their knowledge and motivation pre-and post-intervention. Testimonials showed that they were more knowledgeable about their condition, diet, and physical activity. One participant said, *"I became more knowledgeable...now I know diet types and what carbohydrates mean…"* (Focus Group with Participants, P05, Female).

The intervention also impacted participants' social and physical environment, leading to a supportive atmosphere for behavioural change. Participants indicated that the changing social environment in SA encouraged the adoption of a healthier lifestyle and influenced their social networks. As one participant put it, *"While I was changing some of my old habits, my social network adapted..."* (Interview with Participants, P03, Female).

Health coaching provided a more personalised approach than traditional care, fostering motivation and leading to a change in participants' views about their condition. This motivation was further fuelled by the noticeable changes in outcomes such as HbA1c levels. Participants recognised the power of lifestyle changes and felt empowered by their improved health results, stating, *"...after the intervention, I realised modifying the lifestyle is the actual treatment..."* (Interview with Participants, P13, Male). Consequently, there were requests to extend the intervention, given the observable impacts, including reductions in A1c levels and body weight.

Participants in SA observed notable differences between the novel concept of health coaching and traditional care. The former was seen as superior, with participants expressing dissatisfaction with traditional care's impersonal nature. Health coaching, in contrast, provided a more personalised approach, allowing patients to voice their concerns. As one participant expressed, *"I would choose this program without hesitation; at least I finish each session while I'm comfortable..."* (Interview with Participants, P03, Female).

Traditional care was perceived as a top-down process, with patients passively receiving pre-prepared information or plans. The health coaching intervention, however, positioned participants as active stakeholders in their own care by encouraging goal-setting and plan creation. A participant noted, *"Your program helped me use new skills such as setting plans and having short-term and long-term goals..."* (Interview with Participants, P08, Male).

Frustration and even anger marked participants' feelings about traditional care, citing time constraints, a lack of empathy, and the expense of private consultations. Health coaching was seen as a way to alleviate these issues, with participants suggesting the incorporation of health coaching clinics in hospitals to facilitate meaningful patient discussions and foster better disease self-management. A participant suggested, *"I think it is important to recruit a coach in the hospital... 90% of patients with chronic diseases need to sit with someone to have meaningful discussions..."* (Interview with Participants, P08, Male).

Theme 4: Improvement Opportunities in the Future Health Coaching RCT

For peer review only - http://bmjopen.bmj.com/site/about/guidelines.xhtml

As the inaugural health coaching intervention within the Saudi healthcare system, this initiative stands as a landmark reflecting the strategic direction of the Saudi Ministry of Health towards more proactive, patient-centred care. In anticipation of scaling up such interventions, participants and health coaches provided constructive feedback for refinement. Participants expressed a preference for more frequent coaching interactions and the opportunity for extended face-to-face engagements, suggesting a deeper and more personal connection could enhance the intervention's impact. "*Having more time and direct sessions would greatly benefit the experience*" shared one participant (Interview with Participants, P01, Female).

Health coaches recommended the integration of multidisciplinary expertise, particularly dietitians and physicians, to provide comprehensive care and address the complex needs of diabetes management. "*Incorporating a nutritionist and a physician would offer a more rounded approach to patient care,*" proposed a coach (Focus Group with Coaches, C03, Female). This suggestion is indicative of a holistic strategy, recognizing the multifactorial nature of diabetes.

To bolster participant engagement, the provision of incentives was suggested, alongside enhanced access to healthcare services. Additionally, health coaches proposed the creation of educational content, such as a succinct video delineating the health coaching concept, to facilitate a clearer understanding for future participants. "*A visual introduction to health coaching might bridge the initial knowledge gap,*" a coach highlighted (Focus Group with Coaches, C02, Female).

Preliminary Effects of the Health Coaching Intervention

Our quantitative analysis, using a linear regression model adjusted for baseline levels, revealed significant health improvements from the health coaching intervention. Specifically, HbA1c levels in the intervention group decreased significantly, with a mean difference of -1.86 (95% CI: -2.71 to -1.01, P<0.001) compared to the control group. Secondary outcomes further underscored the intervention's efficacy; BMI decreased by a mean of -1.02 (95% CI: -2.01 to -0.041, P=0.042), and waist circumference reduced by -6.89 (95% CI: -10.17 to -3.61, P<0.001). While changes in Mean Arterial Pressure (MAP) and weight were observed, they did not reach statistical significance (MAP mean difference: -0.68, 95% CI: -7.46 to 6.09, P=0.83; weight mean difference: -2.58, 95% CI: -5.25 to 0.082, P=0.057). Detailed outcomes, including improvements in patients' self-efficacy and diabetes self-management, are delineated in Tables 3 and 4.

Table 3: Groups means and SDs at study baseline and endpoint.

	Group	N	Mean	Std. Deviation

For peer review only

A1c level at the study baseline	Intervention Group	14	9.12	2.50
	Control Group	15	8.73	1.69
A1c level at the study endpoint	Intervention Group	14	7.29	1.38
	Control Group	15	8.93	1.81
Mean Arterial Pressure at the study baseline	Intervention Group	14	91.7	13.56
	Control Group	15	86.73	11.66
Mean Arterial Pressure at the study endpoint	Intervention Group	14	92.57	9.11
	Control Group	15	90.33	12.91
Weight in Kg at the study baseline	Intervention Group	14	83.50	11.97
	Control Group	15	81.21	12.76
Weight in Kg at the study endpoint	Intervention Group	14	80.70	10.80
	Control Group	15	81.21	12.60
BMI at the study baseline	Intervention Group	14	30.39	4.57
	Control Group	15	30.87	5.43
BMI at the study endpoint	Intervention Group	14	29.39	4.33
	Control Group	15	30.86	5.318
Waist Circumference (cm) at the study baseline	Intervention Group	14	109.35	13.60
	Control Group	15	106.66	10.22
Waist Circumference (cm) at the study endpoint	Intervention Group	14	102.21	12.55
	Control Group	15	106.76	9.91

Table 4: Outcomes mean differences taking into account the baseline value regression.

For peer review only - http://bmjopen.bmj.com/site/about/guidelines.xhtml

Endpoint HbA1c	Mean difference	t	p-value	*d*	[95% CI]	
Group*	-1.864463	-4.50	<0.001	-0.93	-2.716065	-1.012862
Baseline HbA1c	.566714		<0.001		.3601448	.7732833
Endpoint MAP	Mean difference	t	p-value		[95% CI]	
Group*	-.6833264	-0.21	0.837		-7.463123	6.09647
Baseline MAP	.5809645	4.38	<0.001		.3083653	.8535638
Endpoint Weight	Mean difference	t	p-value		[95% CI]	
Group	-2.584974	-1.99	0.057		-5.252461	.0825133
Baseline Weight T1	.9090889	16.84	<0.001		.7981218	1.020056
Endpoint BMI	Mean difference	t	p-value		[95% CI]	
Group	-1.024214	-2.14	0.042		-2.006754	-.0416741
Baseline BMI T1	.9322785	19.02	<0.001		.8315092	1.033048
Endpoint WC	Mean difference	t	p-value		[95% CI]	
Group	-6.895251	-4.32	<0.001		-10.17924	-3.611259
Baseline WC	.8732792	12.72	<0.001		.7322131	1.014345

Abbreviations: HbA1c: Haemoglobin A1c, MAP: Mean Arterial Pressure, BMI: Body Mass Index, WC: Waist Circumference, t: t-test, CI: Confidence Interval, d: Cohen's d (Effect size) *(Group = Intervention mean – Control mean)

Patients' Self-Efficacy Questionnaire

The self-efficacy of patients was assessed using an 8-item scale (30), where scores ranged from 1 (least confident) to 10 (most confident). Higher mean scores indicated greater self-efficacy. Both intervention and control groups displayed an increased average response from baseline to the study endpoint, with higher improvements observed in the intervention group (see Supplemental Table 9). The groups exhibited a statistically significant difference (P = .006, 59 % CI .713 to 3.839) in confidence related to controlling their condition and maintaining exercise. Similarly, a significant difference (P = .006, 59 % CI 0.82 to 4.35) was noted in

For peer review only - http://bmjopen.bmj.com/site/about/guidelines.xhtml

confidence regarding appropriate food choices when hungry. This suggests the intervention group saw greater improvements in self-efficacy compared to the control group.

Diabetes Self-Care Activity

The Diabetes Self-Care Activity scale (31), scoring 0 (no adherence) to 7 (complete adherence), measured patients' adherence to specific activities over the past week. Average scores showed overall improvements in self-care for both the intervention and control groups across all activities, with the intervention group often meeting or exceeding American Diabetes Association recommendations (see Supplemental Table 10) (32). For instance, adherence to a healthy eating plan in the intervention group increased from an average of 1.64 to 5.00 days per week. Physical exercise also increased in both groups, with a larger improvement in the intervention group (3.57 to 5.64 days). Similarly, there was improved management of carbohydrate intake in the intervention group, from 1.71 to 4.00 days per week.

Integration Results:

The key findings from both quantitative and qualitative data, aligned with research objectives, were assembled in a joint display table (see Supplemental Table 4). This table was structured to present the study's critical concepts and the corresponding progression criteria, which provide a comprehensive understanding of the mixed-methods findings. The phrase 'there is no significance in moving forward', as used within our progression criteria, draws from the principle of setting clear benchmarks for feasibility studies. These benchmarks are pivotal in assessing whether a study exhibits the potential for upscaling to a full-scale RCT. They embody a balance between the ambition to proceed and the pragmatism to address early issues, as recommended by best practices in trial management (33). In our context, this phrase indicates that if participant recruitment, retention, or adherence fall below the predetermined percentages, the study may not have the robustness required for expansion. Such a stance encourages rigorous scrutiny of recruitment rates and intervention adherence to ensure only well-substantiated research advances.

The integration of quantitative and qualitative data insights offered enhanced comprehension of findings collated and analysed separately. This approach bolstered the credibility and transparency of the overall results through data triangulation, allowing for comparison and validation of findings that a single method wouldn't permit. The integrated results substantiated the pertinence of mixed approaches, essential to the study's feasibility and acceptability exploration in the Saudi context for a large-scale intervention with T2DM patients.

The study reported high screening, recruitment, and retention rates. These results were corroborated by the integrated analysis, suggesting a promising potential for the study within the Saudi setting. This was further supported by participants' willingness to engage in the intervention, extend invitations to family members, and their adherence to complete the intervention, despite some rescheduled sessions.

Notably, improvements were observed across five out of seven preliminary outcomes, which were validated by qualitative data. Participants and coaches reported positive experiences with the intervention's usability, design, and content. Moreover, qualitative results echoed the high

For peer review only

satisfaction levels among participants identified in the quantitative Likert-scale Satisfaction Questionnaire. Despite the challenge posed by session rescheduling flexibility for coaches, it facilitated participant adherence, as supported by quantitative data: all participants completed their sessions with only eleven sessions rescheduled.

Discussion:

The findings from this feasibility study endorse advancing towards a larger RCT for a health coaching intervention among patients with T2DM in SA. Notably, the high rates of eligibility, recruitment, and retention reflect the study's effectiveness in engaging participants, which is pivotal for ensuring the representativeness and reliability of the results.

Achieving a recruitment rate of 79% is particularly significant; it not only indicates the interest and willingness of the target population to participate but also reinforces the potential for this health coaching intervention to be scaled up and applied broadly within the Saudi healthcare setting. This recruitment performance, especially when contrasted with rates from prior studies, such as those by Basak C & Schou (2013) and Cho et al. (2011) (34-36), highlights the tailored approach's resonance with the participants even amidst the operational challenges presented by the COVID-19 pandemic.

Retention is another key indicator of a study's appeal and the practicality of its intervention. The retention rate in this study exceeded that of a similar study (83.6%) (34), as well as the rates reported by Basak C & Schou (2013) (87%) (36) and Cho et al. (2011) (90%).(35) Our retention rate, which remained high throughout the study period, suggests that the intervention was well-received and retained its relevance and value to the participants, motivating them to continue through the study's conclusion. The flexibility allowed in rescheduling coaching sessions contributed to this success, though it did increase the workload for the coaches, an aspect to consider in the planning of future RCTs.

As we reflect on the integrated quantitative and qualitative findings, we see confirmation of the feasibility of conducting a larger RCT, despite restrictions due to COVID-19. The comprehensive data collection, appropriate outcome assessments, and participant adherence collectively point to the potential for effective execution and data gathering in a subsequent, more extensive RCT. Enhancing reach and diversity through a variety of recruitment methods, and the use of a concise explanatory video to describe the intervention, are strategies that can further streamline the process and solidify understanding among future participants.

The feasibility study utilised the COM-B model and the BCTs taxonomy, which proved to be effective in guiding intervention content and addressing participant barriers. The most frequently used BCTs aligned with previous literature. The study adapted to telephone-based sessions due to COVID-19 restrictions, but a mixed approach with face-to-face interactions was preferred by both participants and coaches. A longer intervention duration of up to six months with more face-to-face sessions was recommended to ensure sustained behaviour changes.

The intervention's positive outcomes, particularly the reduction in HbA1c levels, align with evidence suggesting that interventions emphasizing HbA1c as the primary outcome are more

For peer review only

effective. This underscores the importance of selecting outcomes aligned with the intervention's central goal. This study supports previous findings (37), indicating that using HbA1c as the primary intervention outcome can enhance effectiveness. Emphasizing glycaemic control as the primary outcome significantly impacted participants' diabetes self-management in this study. Participants actively developed and utilized key skills to manage their condition, indicating the efficacy of the coaching model. Their satisfaction was notably linked to a meaningful reduction in HbA1c levels after the intervention, emphasizing the holistic impact of the health coaching model.

Although feasibility studies are not formally powered to detect conclusive effects on interventions (38), this study demonstrated the high acceptability and usability of the intervention in the Saudi context. However, concerns were raised by health coaches regarding their volunteer roles and the challenges of finding suitable locations for coaching sessions. These issues need to be addressed in planning for the larger RCT. The ongoing societal changes in SA, along with participants' satisfaction with the intervention, suggest potential acceptance for a large-scale RCT.

For the upcoming definitive RCT, it's crucial to integrate participant and coach feedback to refine the approach. Recommendations include lengthening the program to six months with increased in-person interactions, involving multidisciplinary experts, regular coach coordination, participant incentives, and a brief explanatory video to introduce health coaching.

This study introduces a patient-cantered health coaching approach to Saudi Arabia, showcasing its effectiveness in managing T2DM and its potential applicability to other chronic conditions. The use of a detailed curriculum and the COM-B model has proven beneficial, supporting the Saudi Ministry of Health's move towards behaviour-based strategies and paving the way for broader implementation.

The study's positive reception and outcomes reinforce the viability of health coaching in T2DM management, offering insights for future research and substantiating its growing acceptance and practicality.

Strengths and Limitations of this Study

This study applied the BCW framework, proving beneficial for a comprehensive understanding of the issue of T2DM in the context of SA through an in-depth analysis of prior literature. Accurate diagnosis of the identified problem, considering potential barriers and facilitators, plays a pivotal role in the systematic roadmap. This process initiates with recognizing the actual issue and concludes with specifying target behaviours necessary to achieve the intended goals. Employing the COM-B model, we conducted a narrative review to pinpoint facilitators and barriers in controlling T2DM. The unique approach involved employing the BCW, COM-B model, and BCTs taxonomy, enabling a meticulous analysis of each health coaching session and the coding of each applied BCT. This resulted in a clear and detailed description of the intervention.

The inclusion of progression criteria, as recommended by the CONSORT 2010 statement, aided in decision-making for the larger trial (39). The study findings, including the effect size

For peer review only - http://bmjopen.bmj.com/site/about/guidelines.xhtml

and retention rate, will inform the estimation of the sample size for the future main trial. The study also utilised both the Summary of Diabetes Self-Care Activity questionnaire and the Self-efficacy Scale for Diabetes scale, providing a comprehensive assessment of health coaching's impact. A mixed methods approach was employed to gather diverse perspectives, enhance validity and reliability, and produce more comprehensive findings.

However, the study is not without limitations, particularly concerning generalizability due to the specific setting and population. The small sample size may have led to potential Type 2 errors, limiting the study's ability to detect certain effects. While the recruitment of the intervention sample from a public hospital may not be directly applicable to primary care settings, it could impact the broader generalizability of findings to other individuals.

It is essential to note that although statistically significant effects were detected, the study's acknowledgement of limitations in statistical power reflects an awareness of potential constraints in identifying smaller yet existing effects. These limitations should be considered when interpreting the study's outcomes.

Conclusion

This mixed methods feasibility RCT demonstrated the feasibility and acceptability of implementing a health coaching approach for individuals with T2DM in SA. The integration of qualitative and quantitative findings yielded positive outcomes, providing strong support for conducting a larger RCT to further investigate the effectiveness of health coaching in enhancing diabetes self-management among individuals with T2DM in SA. Importantly, this study offers valuable insights for future trials, particularly in terms of incorporating the COM-B model and BCTs taxonomy into the health coaching approach. The positive feedback received for implementing a patient-centred approach indicates that it could help manage other chronic diseases. This aligns with the Saudi Arabian Ministry of Health's behaviour-focused strategies, providing more evidence to support the expansion of health coaching. As a result, these insights have significant implications for the current management practices of T2DM and beyond. Future research should also explore the social and environmental impacts of Vision 2030, the national vision of SA, on diabetes self-management. By considering contextual factors that may influence the implementation and outcomes of health coaching interventions for diabetes management in SA, further investigations will deepen our understanding and inform strategies to improve diabetes care and other chronic diseases in the country.

Supplementary Figure:

For peer review only - http://bmjopen.bmj.com/site/about/guidelines.xhtml

Supplementary Figure 1: CONSORT Flow Diagram of Participant Progress through the Phases of the Randomised Trial. This diagram depicts the enrolment, allocation, follow-up, and analysis stages of participants. It includes the number of individuals assessed for eligibility, excluded, or randomised into the trial, as well as those who received the intervention, followed up, and were included in the analysis.

Supplementary Figure 2: Detailed Thematic Analysis of Intervention Components. This map illustrates the comprehensive thematic breakdown of the intervention, highlighting key areas such as feasibility, mechanisms, and potential improvements. It details the sub-themes of intervention design, content delivery, and participant engagement, as well as the impact on trial staff and researchers. Suggestions for the future RCT based on coaches' and participants' feedback are also included.

For peer review only

Author Contributions: A.N.A. was responsible for the study's conception and design, data acquisition, analysis, and interpretation, as well as the drafting the manuscript. E.G.

contributed to the critical revision of the manuscript based on data analysis. AA, AM, MA, A.S.A were responsible for the implementation of the intervention. All authors have thoroughly reviewed and approved the final version of the manuscript for publication

Funding: This study received no external funding.

Competing interests: The authors declare no conflicts of interest.

Patient consent for publication: Not required.

Ethics approval: Ethical approval for this study was granted by both the University of Sheffield and the Institutional Review Board (IRB) committee at King Fahad Medical City (KFMC), with the assigned IRB Log Number: 21-062E.

Data Availability Statement: All data generated or analysed during this study are available upon reasonable request from the corresponding author.

Acknowledgement: We express our sincere gratitude to the study participants for their valuable time and willingness to participate. We are also grateful to KFMC and Al-Zulfi General Hospital for granting permission to conduct the intervention. Furthermore, we extend our thanks to the University of Sheffield Institutional Open Access Fund for their support towards this research. Finally, we would like to acknowledge that this paper will be published under a Creative Commons Attribution (CC BY) license.

References

For peer review only - http://bmjopen.bmj.com/site/about/guidelines.xhtml

1. Alqurashi KA, Aljabri KS, Bokhari SA. Prevalence of diabetes mellitus in a Saudi community. Annals of Saudi Medicine. 2011;31(1):19-23.
2. Association AD. Diagnosis and classification of diabetes mellitus. American Diabetes Association; 2010. p. S62-S.
3. Hex N, Bartlett C, Wright D, Taylor M, Varley D. Article: Health Economics Estimating the current and future costs of Type 1 and Type 2 diabetes in the UK, including direct health costs and indirect societal and productivity costs.
4. Alqahtani M, Almutairi FE, Albasseet AO, Almutairi KE. Knowledge, Attitude, and Practice of Diabetes Mellitus Among the Saudi Population in Riyadh, Saudi Arabia: A Quantitative Study. Cureus. 2020.
5. Organization WH. Noncommunicable diseases 2022 [Available from: https://www.who.int/news-room/fact-sheets/detail/noncommunicable-diseases.
6. Alneami YM, Coleman CL. Risk Factors for and Barriers to Control Type-2 Diabetes among Saudi Population. Global Journal of Health Science. 2016;8(9).
7. Alabdulbaqi D. A Cultural Adaptation of a Diabetes Self-Management Programme for Patients with Type 2 Diabetes Attending a Primary Health Care Centre in Saudi Arabia. 2019.
8. Al Slamah T, Nicholl BI, Alslail FY, Harris L, Melville CA, Kinnear D. Cultural adaptation of self-management of type 2 diabetes in Saudi Arabia (qualitative study). PLOS ONE. 2020;15(7):e0232904-e.
9. Wolever RQ, Dreusicke M, Fikkan J, Hawkins TV, Yeung S, Wakefield J, et al. Integrative Health Coaching for Patients With Type 2 Diabetes. The Diabetes Educator. 2010;36(4).
10. Wong-Rieger D, Rieger FP. Health coaching in diabetes: empowering patients to self-manage. Elsevier; 2013. p. 41-4.
11. Hill B, Richardson B, Skouteris H. Do we know how to design effective health coaching interventions: A systematic review of the state of the literature. American Journal of Health Promotion. 2015;29(5):e158-e68.
12. Pirbaglou M, Katz J, Motamed M, Pludwinski S, Walker K, Ritvo P. Personal Health Coaching as a Type 2 Diabetes Mellitus Self-Management Strategy: A Systematic Review and Meta-Analysis of Randomized Controlled Trials. SAGE Publications Inc.; 2018. p. 1613-26.
13. Hill B, Richardson B, Skouteris H. Do we know how to design effective health coaching interventions: a systematic review of the state of the literature. American journal of health promotion : AJHP. 2015;29(5):e158-68.
14. Alanazi FK, Alotaibi JS, Paliadelis P, Alqarawi N, Alsharari A, Albagawi B. Knowledge and awareness of diabetes mellitus and its risk factors in saudi arabia. Saudi Arabian Armed Forces Hospital; 2018. p. 981-9.
15. Eldridge SM, Chan CL, Campbell MJ, Bond CM, Hopewell S, Thabane L, et al. CONSORT 2010 statement: Extension to randomised pilot and feasibility trials. The BMJ. 2016;355.
16. Almulhim AN, Goyder E, Caton SJ. Assessing the Feasibility and Acceptability of Health Coaching as a New Diabetes Management Approach for the People with Type 2 Diabetes in Saudi Arabia: A Protocol for a Mixed Methods Feasibility Study. International Journal of Environmental Research and Public Health. 2022;19(22):15089.
17. Hariton E, Locascio JJ. Randomised controlled trials – the gold standard for effectiveness research: Study design: randomised controlled trials. Blackwell Publishing Ltd; 2018. p. 1716-.
18. Michie S, Ashford S, Sniehotta FF, Dombrowski SU, Bishop A, French DP. A refined taxonomy of behaviour change techniques to help people change their physical activity and healthy eating behaviours: The CALO-RE taxonomy. Psychology and Health. 2011;26(11):1479-98.
19. Michie S, van Stralen MM, West R. The behaviour change wheel: A new method for characterising and designing behaviour change interventions. Implementation Science. 2011;6(1):42-.

For peer review only - http://bmjopen.bmj.com/site/about/guidelines.xhtml

20. Dyson PA, Twenefour D, Breen C, Duncan A, Elvin E, Goff L, et al. Diabetes UK evidence-based nutrition guidelines for the prevention and management of diabetes. Diabetic Medicine. 2018;35(5):541-7.
21. Al Slamah T, Nicholl BI, Alslail FY, Melville CA. Self-management of type 2 diabetes in gulf cooperation council countries: A systematic review. PLOS ONE. 2017;12(12):e0189160-e.
22. Cocks K, Torgerson DJ. Sample size calculations for pilot randomized trials: A confidence interval approach. Journal of Clinical Epidemiology. 2013;66(2):197-201.
23. Julious SA. Sample size of 12 per group rule of thumb for a pilot study. Pharmaceutical Statistics. 2005;4(4):287-91.
24. Braun V, Clarke V. Using thematic analysis in psychology. Qualitative Research in Psychology. 2006;3(2):77-101.
25. McCrudden MT, Marchand G, Schutz PA. Joint displays for mixed methods research in psychology. Methods in Psychology. 2021;5:100067.
26. Morgan DL. Commentary—After triangulation, what next? : Sage Publications Sage CA: Los Angeles, CA; 2019. p. 6-11.
27. Creswell JW, Clark VLP. Designing and conducting mixed methods research: Sage publications; 2017.
28. Eldridge SM, Chan CL, Campbell MJ, Bond CM, Hopewell S, Thabane L, et al. CONSORT 2010 statement: extension to randomised pilot and feasibility trials. bmj. 2016;355.
29. Moore GF, Audrey S, Barker M, Bond L, Bonell C, Hardeman W, et al. Process evaluation of complex interventions: Medical Research Council guidance. BMJ (Online). 2015;350.
30. BOOK QC. Stanford Patient Education Research Center. American Journal of Public Health. 1990;80:446-52.
31. Toobert DJ, Hampson SE, Glasgow RE. The summary of diabetes self-care activities measure: Results from 7 studies and a revised scale. Diabetes Care. 2000;23(7):943-50.
32. Association AD. Understanding A1C Diagnosis: ADA; 2022 [Available from: https://diabetes.org/diabetes/a1c/diagnosis.
33. Avery KN, Williamson PR, Gamble C, Francischetto EOC, Metcalfe C, Davidson P, et al. Informing efficient randomised controlled trials: exploration of challenges in developing progression criteria for internal pilot studies. BMJ open. 2017;7(2):e013537.
34. Kim MT, Kim KB, Huh B, Nguyen T, Han H-R, Bone LR, et al. The Effect of a Community-Based Self-Help Intervention. American Journal of Preventive Medicine. 2015;49(5).
35. Cho J-H, Kwon H-S, Kim H-S, Oh J-A, Yoon K-H. Effects on diabetes management of a health-care provider mediated, remote coaching system via a PDA-type glucometer and the Internet. Journal of Telemedicine and Telecare. 2011;17(7).
36. Basak Cinar A, Schou L. Health promotion for patients with diabetes: health coaching or formal health education? International Dental Journal. 2014;64(1).
37. Almulhim AN, Hartley H, Norman P, Caton SJ, Doğru OC, Goyder E. Behavioural Change Techniques in Health Coaching-Based Interventions for Type 2 Diabetes: A Systematic Review and Meta-Analysis. BMC Public Health. 2023;23(1):1-21.
38. Lee EC, Whitehead AL, Jacques RM, Julious SA. The statistical interpretation of pilot trials: should significance thresholds be reconsidered? BMC medical research methodology. 2014;14(1):1-8.
39. Schulz KF, Altman DG, Moher D. CONSORT 2010 Statement: Updated guidelines for reporting parallel group randomised trials. BMJ (Online). 2010;340(7748):698-702.

For peer review only

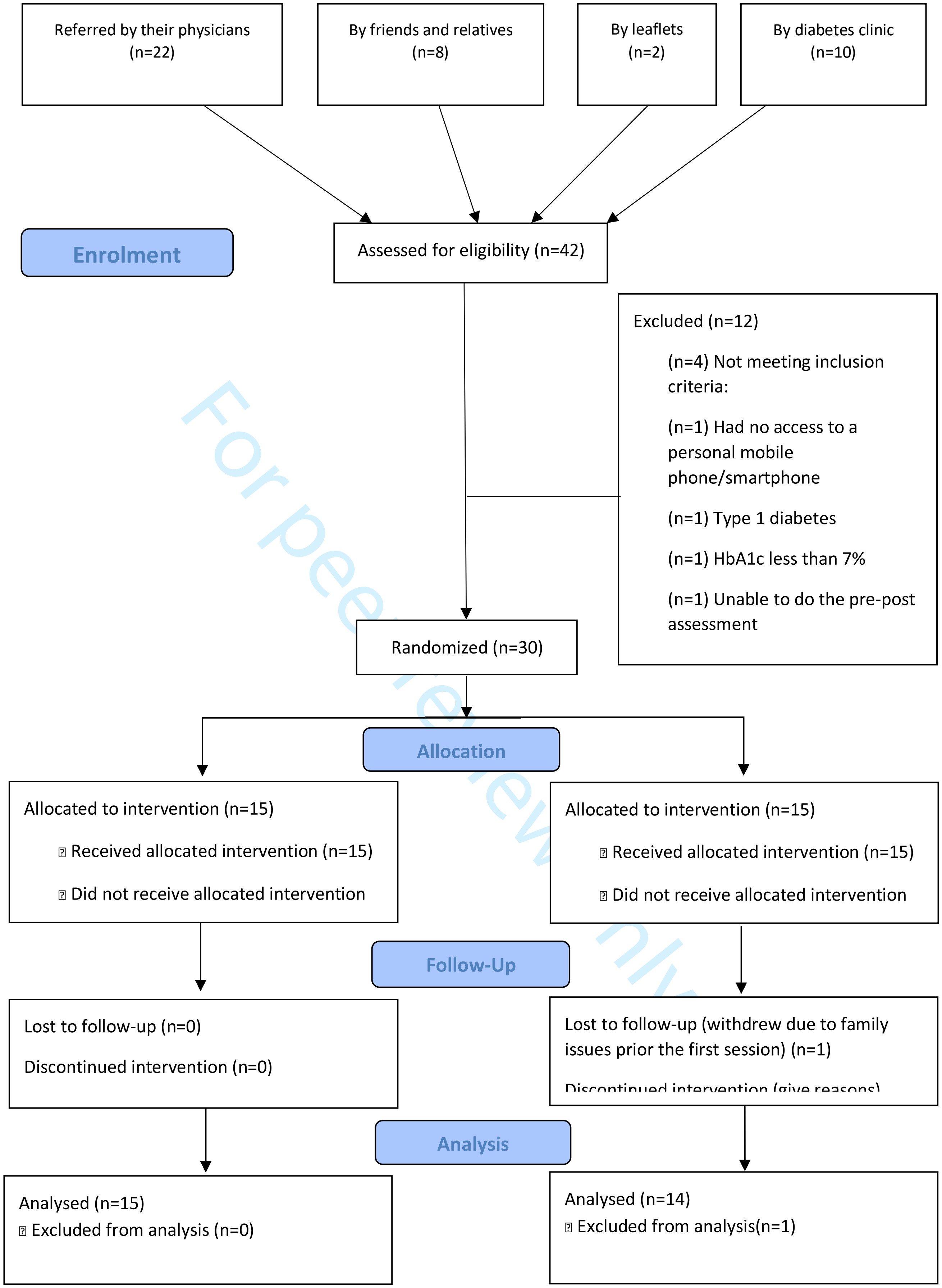

Figure 1: The study CONSORT flow chart

For peer review only

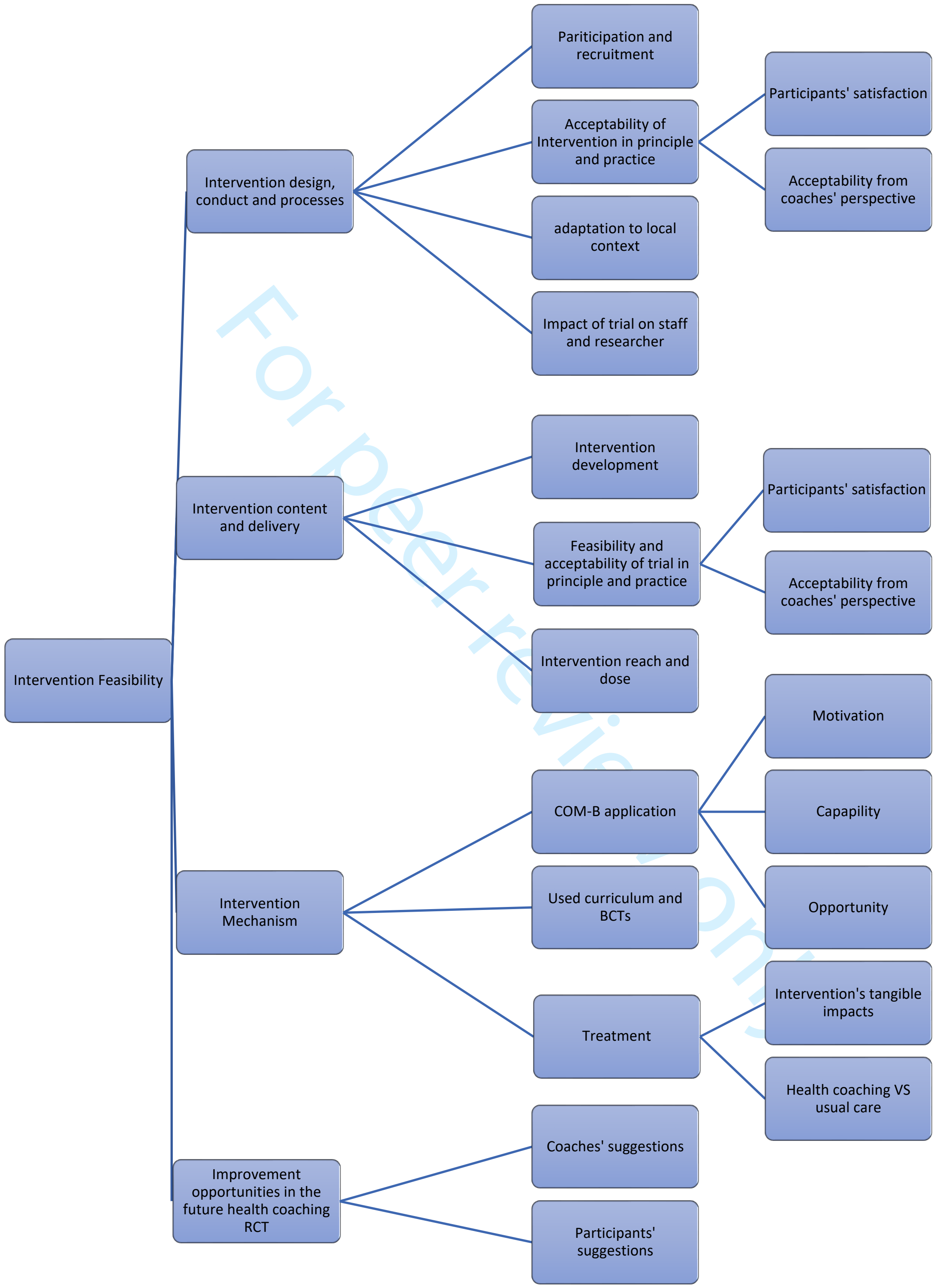

Figure 2: Thematic analysis map

For peer review only - http://bmjopen.bmj.com/site/about/guidelines.xhtml

CONSORT 2010 checklist of information to include when reporting a pilot or feasibility trial*

Section/Topic	Item No	Checklist item	Reported on page No
**Title and abstract**			
	1a	Identification as a pilot or feasibility randomised trial in the title	1
	1b	Structured summary of pilot trial design, methods, results, and conclusions (for specific guidance see CONSORT abstract extension for pilot trials)	1
**Introduction**			
Background and objectives	2a	Scientific background and explanation of rationale for future definitive trial, and reasons for randomised pilot trial	2
	2b	Specific objectives or research questions for pilot trial	3
**Methods**			
Trial design	3a	Description of pilot trial design (such as parallel, factorial) including allocation ratio	3
	3b	Important changes to methods after pilot trial commencement (such as eligibility criteria), with reasons	4
Participants	4a	Eligibility criteria for participants	4
	4b	Settings and locations where the data were collected	4
	4c	How participants were identified and consented	4
Interventions	5	The interventions for each group with sufficient details to allow replication, including how and when they were actually administered	4-5
Outcomes	6a	Completely defined prespecified assessments or measurements to address each pilot trial objective specified in 2b, including how and when they were assessed	5
	6b	Any changes to pilot trial assessments or measurements after the pilot trial commenced, with reasons	5
	6c	If applicable, prespecified criteria used to judge whether, or how, to proceed with future definitive trial	5
Sample size	7a	Rationale for numbers in the pilot trial	5
	7b	When applicable, explanation of any interim analyses and stopping guidelines	n/a
Randomisation:			
Sequence generation	8a	Method used to generate the random allocation sequence	4
	8b	Type of randomisation(s); details of any restriction (such as blocking and block size)	4
Allocation concealment mechanism	9	Mechanism used to implement the random allocation sequence (such as sequentially numbered containers), describing any steps taken to conceal the sequence until interventions were assigned	4

For peer review only

For peer review only - http://bmjopen.bmj.com/site/about/guidelines.xhtml

Implementation	10	Who generated the random allocation sequence, who enrolled participants, and who assigned participants to interventions	4
Blinding	11a	If done, who was blinded after assignment to interventions (for example, participants, care providers, those assessing outcomes) and how	n/a
	11b	If relevant, description of the similarity of interventions	n/a
Statistical methods	12	Methods used to address each pilot trial objective whether qualitative or quantitative	6
**Results**			
Participant flow (a diagram is strongly recommended)	13a	For each group, the numbers of participants who were approached and/or assessed for eligibility, randomly assigned, received intended treatment, and were assessed for each objective	9
	13b	For each group, losses and exclusions after randomisation, together with reasons	9-10
Recruitment	14a	Dates defining the periods of recruitment and follow-up	9
	14b	Why the pilot trial ended or was stopped	n/a
Baseline data	15	A table showing baseline demographic and clinical characteristics for each group	8
Numbers analysed	16	For each objective, number of participants (denominator) included in each analysis. If relevant, these numbers should be by randomised group	10
Outcomes and estimation	17	For each objective, results including expressions of uncertainty (such as 95% confidence interval) for any estimates. If relevant, these results should be by randomised group	19-20
Ancillary analyses	18	Results of any other analyses performed that could be used to inform the future definitive trial	13
Harms	19	All important harms or unintended effects in each group (for specific guidance see CONSORT for harms)	n/a
	19a	If relevant, other important unintended consequences	n/a
**Discussion**			
Limitations	20	Pilot trial limitations, addressing sources of potential bias and remaining uncertainty about feasibility	24
Generalisability	21	Generalisability (applicability) of pilot trial methods and findings to future definitive trial and other studies	24
Interpretation	22	Interpretation consistent with pilot trial objectives and findings, balancing potential benefits and harms, and considering other relevant evidence	24
	22a	Implications for progression from pilot to future definitive trial, including any proposed amendments	23-24
**Other information**			
Registration	23	Registration number for pilot trial and name of trial registry	n/a
Protocol	24	Where the pilot trial protocol can be accessed, if available	5
Funding	25	Sources of funding and other support (such as supply of drugs), role of funders	n/a
	26	Ethical approval or approval by research review committee, confirmed with reference number	26

For peer review only

For peer review only - http://bmjopen.bmj.com/site/about/guidelines.xhtml

Citation: Eldridge SM, Chan CL, Campbell MJ, Bond CM, Hopewell S, Thabane L, et al. CONSORT 2010 statement: extension to randomised pilot and feasibility trials. BMJ. 2016;355. This is an Open Access article distributed in accordance with the terms of the Creative Commons Attribution (CC BY 3.0) license (http://creativecommons.org/licenses/by/3.0/), which permits others to distribute, remix, adapt and build upon this work, for commercial use, provided the original work is properly cited.

*We strongly recommend reading this statement in conjunction with the CONSORT 2010, extension to randomised pilot and feasibility trials, Explanation and Elaboration for important clarifications on all the items. If relevant, we also recommend reading CONSORT extensions for cluster randomised trials, non-inferiority and equivalence trials, non-pharmacological treatments, herbal interventions, and pragmatic trials. Additional extensions are forthcoming: for those and for up-to-date references relevant to this checklist, see www.consort-statement.org.

For peer review only

For peer review only - http://bmjopen.bmj.com/site/about/guidelines.xhtml

BMJ Open

Feasibility and Acceptability of a Tailored Health Coaching Intervention to Improve Type 2 Diabetes Self-Management in Saudi Arabia: A Mixed-Methods Randomised Feasibility Trial

Journal:	*BMJ Open*
Manuscript ID	bmjopen-2023-078631.R3
Article Type:	Original research
Date Submitted by the Author:	24-Apr-2024

Complete List of Authors:	Almulhim, Abdullah; The University of Sheffield, School of Medicine and Population Health; College of Health Sciences Saudi Electronic University<br>Alhowaish, Atheer; King Fahad Medical City<br>Madani, Alaa; King Fahad Medical City<br>AlQaddan, Munirah; King Fahad Medical City<br>Altuwalah, Abdulaziz S.; King Fahad Medical City<br>Goyder, Elizabeth; The University of Sheffield, School of Medicine and Population Health

**Primary Subject Heading**:	Public health
Secondary Subject Heading:	Public health
Keywords:	PUBLIC HEALTH, DIABETES & ENDOCRINOLOGY, Behavior

SCHOLARONE™
Manuscripts

I, the Submitting Author has the right to grant and does grant on behalf of all authors of the Work (as defined in the below author licence), an exclusive licence and/or a non-exclusive licence for contributions from authors who are: i) UK Crown employees; ii) where BMJ has agreed a CC-BY licence shall apply, and/or iii) in accordance with the terms applicable for US Federal Government officers or employees acting as part of their official duties; on a worldwide, perpetual, irrevocable, royalty-free basis to BMJ Publishing Group Ltd ("BMJ") its licensees and where the relevant Journal is co-owned by BMJ to the co-owners of the Journal, to publish the Work in this journal and any other BMJ products and to exploit all rights, as set out in our licence.

The Submitting Author accepts and understands that any supply made under these terms is made by BMJ to the Submitting Author unless you are acting as an employee on behalf of your employer or a postgraduate student of an affiliated institution which is paying any applicable article publishing charge ("APC") for Open Access articles. Where the Submitting Author wishes to make the Work available on an Open Access basis (and intends to pay the relevant APC), the terms of reuse of such Open Access shall be governed by a Creative Commons licence – details of these licences and which Creative Commons licence will apply to this Work are set out in our licence referred to above.

Other than as permitted in any relevant BMJ Author's Self Archiving Policies, I confirm this Work has not been accepted for publication elsewhere, is not being considered for publication elsewhere and does not duplicate material already published. I confirm all authors consent to publication of this Work and authorise the granting of this licence.

For peer review only

Feasibility and Acceptability of a Tailored Health Coaching Intervention to Improve Type 2 Diabetes Self-Management in Saudi Arabia: A Mixed-Methods Randomised Feasibility Trial

Abdullah N. Almulhim[1,2]*, Atheer Alhowaish[3], Alaa Madani[3], Munirah AlQaddan[3], Abdulaziz S. Altuwalah[3], Elizabeth Goyder[1]

1 School of Medicine and Population Health, University of Sheffield, Sheffield S1 4DA, UK

2 Public Health Department, College of Health Sciences, Saudi Electronic University, Riyadh 13316, Saudi Arabia

3 Health Education Administration, King Fahad Medical City, Riyadh 12231, Saudi Arabia

* Correspondence: aalmulhim@seu.edu.sa

Abstract

Background: Around a third of the population of Saudi Arabia have been diagnosed with Type 2 diabetes, a condition often requiring lifestyle changes. Personalised health coaching, a strategy developed to assist individuals in overcoming challenges to adopt healthy behaviours, has not yet been widely applied in the country.

Aims: We aim to explore the feasibility and acceptability of tailored health coaching in Saudi Arabia, in order to help those with Type 2 diabetes to more effectively manage their condition.

Methods: Using a mixed-methods approach, this research involved a randomised controlled trial with 30 Saudi adults who have Type 2 diabetes. They were randomly allocated into either the intervention or control arm for 12 weeks. The Capability, Opportunity, Motivation, and Behaviour framework was used to guide the intervention implementation along with the Behaviour Change Techniques Taxonomy version 1. The primary goal was to assess the suitability and duration of the intervention, recruitment, retention, and completion rates. The secondary outcome focused on the preliminary efficacy of the health coaching measured by the glycaemic index, blood pressure, body mass index, waist circumference, weight, patient self-efficacy, and diabetes self-management.

Results: The results showed high rates of eligibility, recruitment, and retention (a screening rate of 90%, a recruiting rate of 79%, and a retention rate of 97%). Notable improvements were observed in the health coaching group across five outcomes: HbA1c, BMI, waist circumference, patient self-efficacy, and diabetes self-care. Qualitative findings highlighted the participants' perceived benefits from the intervention, including enhanced motivation, better understanding of diabetes management, and a supportive coaching relationship. Participants expressed high satisfaction with the intervention and advocated for its expansion.

Conclusion: The findings demonstrated positive outcomes, supporting the need for a larger randomised controlled trial to evaluate the efficacy of health coaching in improving diabetes self-management among individuals with Type 2 diabetes in Saudi Arabia.

For peer review only - http://bmjopen.bmj.com/site/about/guidelines.xhtml

Restricted - مقيد

For peer review only

Keywords: health behaviour change; health coaching; self-management; behaviour change techniques; type 2 diabetes

Summary Box:

Strengths and limitations of this study:

- First health coaching intervention in Saudi Arabia.
- First use of the COM-B model and BCTs taxonomy in a health coaching intervention
- Adherence to CONSORT 2010 progression criteria.
- Comprehensive impact assessment through mixed methods.
- Limited generalizability due to specific settings and demographics.

Introduction:

Type 2 Diabetes Mellitus (T2DM) is a chronic illness that is becoming more common globally and represents a significant public health concern. Lifestyle factors play a crucial role in the development of this disease, with obesity often manifesting as a symptom of other lifestyle factors such as physical inactivity (1). T2DM can lead to complications such as cardiovascular disease, kidney failure, nerve damage, and ocular and auditory problems, significantly increasing the risk of heart disease and stroke in people with diabetes (2).

Managing diabetes can be a challenging task that encompasses various aspects of life, including social, economic, and healthcare domains (3). To help T2DM patients effectively manage their condition, prioritising self-management is crucial (4). T2DM is a prevalent chronic disease in Saudi Arabia (SA), with one of the highest rates in the Middle East and the seventh-highest globally (5). Unhealthy diets, inactive lifestyles, and rapid urbanisation are all contributing factors to this concerning trend (6).

Despite national efforts to control T2DM in the SA context, the disease continues to pose significant challenges (7). While awareness campaigns are being employed, there is a notable difficulty among most T2DM patients in adopting healthier diets and lifestyles (7). To address this issue, it is necessary to implement patient-centred approaches that prioritise the development of self-management skills rather than relying solely on education-based programs (8).

Health coaching, derived from various disciplines and behaviour change theories, has emerged as a client-centred approach gaining recognition in the field of health promotion. Although it has been widely explored in the literature and has shown promising results, this personalised one-on-one intervention has not yet been implemented within the healthcare systems of SA. Studies have demonstrated that health coaching, centred around patients' values, may offer additional benefits compared to traditional T2DM education programs (9). By focusing on enhancing patients' self-efficacy and essential skills such as goal-setting and problem-solving, health coaching can prove highly effective and financially viable (10). Its successful application in diverse contexts has shown it to be a valuable tool in improving diabetes self-management (11, 12).

For peer review only - http://bmjopen.bmj.com/site/about/guidelines.xhtml

Restricted - مقيد

For peer review only

However, the effectiveness of existing health coaching interventions exhibits inconsistency due to variations in methodology, duration, and intervention content (11). Recent systematic reviews of randomised controlled trials employing health coaching have reported mixed results, with some studies suggesting its effectiveness while others claim it to be ineffective (11, 12). There is no agreed-upon health coaching intervention model, which creates uncertainty regarding the most effective strategies to adopt, particularly in the SA context. The inconsistent findings across studies may be due to the lack of agreement on the active ingredients and content of successful health coaching interventions (13). Additionally, cultural differences between SA and Western countries highlight the need for tailored interventions to ensure that proposed health coaching models are appropriate and relevant (14).

This feasibility study aims to assess the usability and acceptability of a health coaching intervention for T2DM self-management in SA, with the ultimate goal of reducing HbA1c levels and informing a future large-scale randomised controlled trial (RCT).

Materials and Methods

This research was reported in accordance with the Consolidated Standards of Reporting Trials (CONSORT) extension guidelines specific to pilot and feasibility randomised controlled trials (RCTs) (15). A comprehensive protocol for this study has already been published (16).

Design

This is a randomised two-arm feasibility trial evaluating a 3-month intervention. The study adopted a mixed-methods approach, enabling the collection of both quantitative and qualitative data. An RCT design to address both evaluate the feasibility of RCT methods for recruitment, delivery, and data collection, as well as a valid comparison of health coaching's effectiveness between the groups (17). Integral to our approach was the implementation of the Behaviour Change Wheel (BCW) framework, used in adapting the health coaching intervention for the SA context. The BCW informed our intervention design in several key ways: Firstly, by identifying suitable intervention functions from the Capability, Opportunity, Motivation-Behaviour (COM-B) model, we tailored the health coaching to the specific needs and barriers of our target population. Secondly, we utilized the Behaviour Change Technique Taxonomy version 1 (BCTTv1) to code the active ingredients of the intervention, mapping these to the BCW framework for alignment with established behaviour change principles (18). Furthermore, a behavioural analysis, guided by the Theoretical Domains Framework (TDF) functions and behavioural change techniques (BCTs), was conducted to address pre-identified barriers (see Supplemental Table 1). This analysis led to the selection of 36 relevant BCTs, which were mapped to directly target the behavioural barriers in adults with poorly managed T2DM, our intervention's target population (see Supplemental Table 2). By integrating the BCW, we were able to define the problem behaviours and target behaviours for intervention, ensuring a comprehensive and theory-driven approach as advised by BCW guidance (19). Further information on the intervention can be found in a prior publication (16).

Patient and public involvement

Patients and the public were not involved during the process of this research.

For peer review only - http://bmjopen.bmj.com/site/about/guidelines.xhtml

For peer review only

Restricted - مقيد

Intervention Development

There have been various identified target behaviours that would result in the adoption of a healthy lifestyle and diet as recommended by UK lifestyle guidelines.(20) As of now, there are no evidence-based guidelines in SA for T2DM to adopt a healthy lifestyle and diet. Therefore, we have referred to the UK's guidelines on lifestyle as they share similarities in their healthcare systems. (20, 21) These guidelines have been used to determine the most suitable behaviour targets for the Saudi context. We consulted healthcare professionals in the intervention setting, including a dietician, to establish these targets. Through collaborative discussions, we assessed and prioritised behaviours based on their potential impact, the feasibility of measurement, and achievability. As a result of this process, we have proposed four behaviour targets. (18)

The four general behaviours targets of the intervention were: (20, 22)

- Decrease carbohydrate intake for each meal
- Use unsaturated fats as possible (avoid saturated fats)
- Do exercise for 30 min five days on a weekly basis
- Monitor waist circumference, and maintain it below (80 cm for women and 94 cm for men)

The aim was to gradually reduce carbohydrate intake while ensuring that participants met their nutritional requirements. The specific target amounts were communicated to participants during individualized dietary counselling sessions with health coaches, and participants were provided with guidance and meal plans to help them achieve and maintain the desired carbohydrate intake levels. The health coaching intervention used these targets as the benchmarks to track the progress and changes that the study population would make over three months.

We utilised diverse instruments, including questionnaires, interviews, focus groups, and clinical measures, to assess the intervention's feasibility, acceptability, and preliminary impact at two points: baseline and three months post-intervention. In our mixed-methods study, purposive sampling was crucial to achieve data saturation and gather diverse perspectives. We conducted two distinct focus groups—one with six study participants and another with three health coaches—to assess the intervention's impact from multiple angles. Additionally, 14 semi-structured interviews were carried out with selected participants to delve deeper into individual experiences and perceptions. These qualitative methods were complemented by quantitative evaluations at baseline and three months post-intervention, using clinical measures such as HbA1c, BMI, waist circumference, and weight. The quantitative data were analysed using linear regression to determine the mean differences between the intervention and control groups, adjusted for baseline levels. Ethical clearance for the study was granted by the University of Sheffield and the King Fahad Medical City's Institutional Review Board (IRB Log Number: 21-062E).

For peer review only

Restricted - مقيد

Participant Recruitment

Inclusion criteria were: aged over 18, Haemoglobin A1c level of 7.0% or higher, able to read and understand Arabic, have access to a personal mobile phone, be willing to complete the intervention period, be willing to stay in Riyadh (the capital city of Saudi Arabia), and be able to read, understand, and sign the informed consent form.

The recruitment process involved advertising the intervention through multiple channels, including posters, brochures, social media, and healthcare provider referrals. Interested and eligible individuals met the research team for a brief study overview and questionnaire completion, providing demographic data and baseline laboratory reports.

Participants were randomly assigned to one of two groups in a 1:1 ratio by an independent individual using a computer-generated random numbers system in Statistical Package for the Social Sciences (SPSS), ensuring equal allocation chances. The two groups consisted of the intervention group, which received the health coaching intervention, and the control group, which received standard care.

Intervention Delivery

The health coaching intervention was completed in 12 weeks. A detailed intervention curriculum was used in delivering the intervention activities toward achieving the four target behaviours (see Supplemental Table 3).

Three health coaches, AA, AM, and MA, who were qualified through the Saudi Ministry of Health, conducted the intervention sessions. They received training in biweekly face to face (F2F) meetings over six weeks on the intervention curriculum. A workbook was provided as a guide to help them deliver coaching sessions as planned. A detailed explanation has been published elsewhere (16). It is important to note that participants did not directly receive or review the intervention curriculum. Our approach involved ensuring uniformity in intervention procedures by providing health coaches with the curriculum. Each coach was asked to document the BCTs they use in each session. Different BCTs were reported in each session. Each participant was assigned to one of three coaches at the baseline. Each participant received six sessions in total, which were delivered biweekly via video meetings and telephone coaching over three months. Due to the COVID-19 pandemic, the first and last meetings were conducted via video call instead of face-to-face as planned, while the rest were via telephone. Participants were encouraged to contact their coaches via WhatsApp between sessions if they had any related concerns. Eighty-four sessions were undertaken for all participants in the intervention group.

Usual Care (Control Group)

The participants in the control group were provided with the standard care, which involved receiving written materials and brochures on diabetes to increase awareness and promote the advantages of making changes to their health behaviours. Typically, individuals with T2DM have regular appointments with endocrine specialists to monitor their diabetes management. During these visits, the primary objective is to assess if any adjustments are necessary regarding

For peer review only - http://bmjopen.bmj.com/site/about/guidelines.xhtml

Restricted - مقيد

the patient's medication, such as replacement, dosage increase, or maintaining the current prescription.

Data Collection

Baseline data collection included: demographic information, HbA1c, blood pressure, BMI, weight, and waist circumference measurements. Subsequently, we asked participants to complete two electronic questionnaires: the Diabetes Self-Care Activities (SDSCA) scale and the Self-efficacy Scale for Diabetes. The post-intervention data collection replicated the initial session, with repeat measurements of HbA1c, blood pressure, Body mass index (BMI), weight, and waist circumference. Participants were also required to fill out the two initial questionnaires again, along with a Satisfaction Questionnaire designed specifically for the intervention group.

Reach and Retention

As per the study protocol (16), this study aimed to recruit a minimum of 9% (n = 30) of the required sample size for conducting a complete trial (23), Each group was expected to have a minimum of 12 participants to ensure meaningful data interpretation for informing a future definitive randomized controlled trial (24). A total of 24 participants in each group would be required to detect a meaningful clinical difference in HbA1c. The retention rate was evaluated based on the predefined progression criteria described elsewhere (16), with successful retention defined as achieving an 83% retention rate at the intervention endpoint.

Fidelity Assessment

All coaching sessions audio recorded and transcribed. An Arabic native speaker reviewed the entire transcription for accuracy. To ensure consistency and validity, a 10% sample of the transcription (approximately 50 minutes) was translated from Arabic to English, backtranslated, and then examined by a peer reviewer. The data, including coaching sessions, time spent in each session, semi-structured interviews, focus groups, and questionnaires, was meticulously stored and managed for quality control. Specifically, all data were entered and stored in Microsoft Excel twice, independently by two research team members. This double-entry process served as a precautionary measure to minimize data entry errors and enhance the accuracy and reliability of the data collected throughout the trial. Adherence to the intervention was defined by the number of participants who completed 5 out of six coaching sessions ≥ 84%.

Feasibility

Evaluation of feasibility included: participant eligibility and recruitment, retention, data collection adherence, and adherence to the intervention. We recorded participants' views about their engagement, capturing their reasons for interest, active participation, and lack of interest. We tracked and logged the number and percentage of those who were eligible and interested, those who completed the intervention, and those who withdrew to provide insight into the study's recruitment efficiency and participant retention

Acceptability

For peer review only - http://bmjopen.bmj.com/site/about/guidelines.xhtml

Restricted - مقيد

Acceptability of the intervention and its implementation was assessed using qualitative and quantitative data. The aim was to detect any potential challenges within the methodology used to deliver both the trial and the intervention. We conducted post-intervention semi-structured qualitative interviews and focus groups. The intervention's acceptability was primarily gauged through a Likert-scale Satisfaction Questionnaire (14 items) applied to the intervention group (n=14). This questionnaire, originally devised by the Dan Abrahams Healthy Living Centre and subsequently employed by DeJesus et al. (2018), incorporates a variety of question styles (open & closed). Using a Likert scale, it offered a response range from 0 (not at all) to 10 (very much) to capture participant feedback. Given that this scale had not been used in Arabic studies before, we translated it into Arabic and pretested it on a smaller subset before implementing it for the full study sample. In addition to quantitative measures, our mixed-methods approach involved semi-structured interviews and focus groups to qualitatively assess the acceptability of the health coaching intervention. Thematic analysis was used to identify and interpret patterns within the qualitative data, aiming to understand the perceived benefits, challenges, and overall acceptability of the intervention from the participants' perspective.

Data Analysis

We conducted both quantitative and qualitative analyses. For the quantitative part, descriptive analysis was undertaken including screening and recruitment rates, retention rates, adherence to coaching sessions, and recruitment duration. We evaluated the use of BCTs and interactions between participants and coaches during these sessions. A Satisfaction Questionnaire captured participants' experiences, while changes in diabetes self-management and patient self-efficacy were assessed at the beginning and end of the study. The data were entered into Excel and subsequently transferred to SPSS and Stata Statistical Software (STATA) for advanced analysis, which included a linear regression to estimate the mean differences in outcomes.

For the qualitative aspect of our study, purposive sampling was employed to ensure data saturation in conducting interviews and focus groups. We conducted a total of 14 semi-structured interviews and two focus groups, engaging in detailed discussions with participants. The qualitative data were analysed using NVivo software through a reflexive thematic analysis process, adhering to six systematic phases: familiarising ourselves with the data, coding, generating themes, revising themes, defining, and finally reporting (25). Initial coding was conducted by the researcher (A.N.A.), followed by a collaborative review with the research team. During this process, consensus on the emerging themes was reached through regular team meetings and discussions, ensuring accuracy and comprehensiveness in the identification and categorization of themes.

In addition, we integrated the qualitative and quantitative data for comprehensive understanding (26), Using a joint display table (see Supplemental Table 4) (27), we compared and combined these data types. The display helped us identify areas of agreement (convergence), disagreement (divergence), and enhancement (expansion) between the two data types (28). In the integrated data, we denoted convergence with "=", complementarity with "+", and inconsistencies with "≠".

For peer review only - http://bmjopen.bmj.com/site/about/guidelines.xhtml
Restricted - مقيد

Although this feasibility study may not conclusively determine intervention effects, it provides valuable insights for estimating the sample size for a larger trial based on the mean difference and standard deviation of HbA1c.

Results:

Participant Characteristics

The control group (mean age=53.40, SD=8.47) was slightly older than the intervention group (mean age=52, SD=8.32). The majority were married, with monthly incomes ranging from less than 5000 SR (28.6%) to more than 15000 SR (7.1%); 50.0% chose not to declare. About a quarter had completed high school, and an equal proportion had primary education or were literate without formal education. Approximately 31% had lived with T2DM for over ten years, with 24.1% of the intervention group having T2DM for 1-3 years. The majority (89.7%) were on diabetes medications. Table 1 summarises the demographic characteristics of both groups.

Table 1: Summary of the participants' demographic characteristics

For peer review only

For peer review only - http://bmjopen.bmj.com/site/about/guidelines.xhtml

Restricted - مقيد

		Intervention group		Control group	
		Total N (%)	Median (IQR)	Total N (%)	Median (IQR)
Gender	Male	6 (42.9%)		7 (46.7%)	
	Female	8 (57.1%)		8 (53.3%)	
Age	Year	14	54.5 (44.5 to 59.25)	15	54 (50 to 60)
Marital status	Married	14 (100.0%)		14 (93.3%)	
	Single	0 (0.0%)		1 (6.7%)	
Monthly income	less than 5000 (SR)	4 (28.6%)		7 (46.7%)	
	5000-10000 (SR)	2 (14.3%)		0 (0.0%)	
	10000-15000 (SR)	0 (0.0%)		2 (13.3%)	
	more than 15000 (SR)	1 (7.1%)		1 (6.7%)	
	Prefer not to declare	7 (50.0%)		5 (33.3%)	
Education level	Illiterate	0 (0.0%)		0 (0.0%)	
	Primary school	3 (21.4%)		3 (20.0%)	
	Secondary school	2 (14.3%)		2 (13.3%)	
	High school	3 (21.4%)		4 (26.7%)	
	Bachelor's degree	2 (14.3%)		3 (20.0%)	
	Diploma	1 (7.1%)		0 (0.0%)	
	Can read and write	3 (21.4%)		3 (20.0%)	
Since when you were diagnosed with Type 2 diabetes	less than a year	2 (14.3%)		0 (0.0%)	
	1-3	6 (42.9%)		0 (0.0%)	
	3-5	1 (7.1%)		2 (13.3%)	
	5-7	0 (0.0%)		1 (6.7%)	
	7-10	1 (7.1%)		6 (40.0%)	
	more than ten years	3 (21.4%)		6 (40.0%)	
	do not know	1 (7.1%)		0 (0.0%)	
Do you use diabetes medications ?	Yes	12 (85.7%)		14 (93.3%)	
	No	2 (14.3%)		1 (6.7%)	

For peer review only

Restricted - مقيد

Feasibility of the Intervention

Eligibility and Recruitment

The recruitment process took about five weeks, starting on May 1st, 2021, to June 5th, 2021. Leaflets were used for advertising the intervention and were distributed in different places in the hospital, including in waiting areas and on hospital wall notice boards. In addition, we met physicians in-person to introduce the intervention and gave them leaflets with more details to encourage their patients to join in the study.

Of the 42 potential participants initially identified and assessed for eligibility, 38 met the study criteria, resulting in a screening rate of approximately 90%. These 42 individuals were referred by different sources. Twenty-two were referred by their doctors, while the others were recruited through various methods. Two were from leaflets, eight were from suggestions by friends or relatives, and ten were directly engaged at the diabetes clinic. Twelve were excluded, of which four were ineligible because they did not meet the intervention inclusion criteria for the following reasons: one had no access to a personal mobile phone/smartphone, one patient was diagnosed with T1DM, one had A1c below 7%, and one was unable to do pre-and post-assessments. Eight were excluded for other reasons; two did not respond, and six for different reasons, e.g., being too busy. Thirty eligible patients were recruited to take part in the intervention. Out of the 38 eligible patients, 30 were successfully recruited and consented to participate in the intervention, yielding a recruitment rate of approximately 79%. All of them completed baseline assessments and questionnaires, and then randomly, 15 patients were allocated to the intervention group, while the other 15 participants were assigned to the control group. One person who was part of the intervention group withdrew from the trial prior to the first session and was thus not included in the study (see Supplemental Figure 1 CONSORT diagram for more details).

Intervention Delivery

The intervention started with the first session on June 15th, 2021, and ended on September 23rd 2021. The average time spent per participant was between ~ 17.7 to 25.5 in each session. The total range time of all sessions per patient was 109-153 minutes, with an average of 120.8 (SD= 13.7). See Supplemental Table 5 for more details about the time duration of each session.

Retention of Participants

Thirty eligible participants consented to participate in the trial and completed the baseline assessment randomly allocated either to the coaching group (n=15) or the control group (n=15). Of those, all twenty-nine who started the intervention and took the first session remained till the endpoint and completed all the intervention activities. Only one participant allocated to the coaching group had withdrawn and discontinued the study before the first session (retention rate = 97%). All twenty-nine have completed the intervention endpoint assessments. The main progress criteria and feasibility measurements are summarised in Supplemental Table 6.

Adherence to the Coaching Sessions

Fifteen out of thirty participants were randomly allocated to the coaching group. Before the first session, one participant withdrew (P12) due to family issues. The rest of the fourteen have

For peer review only - http://bmjopen.bmj.com/site/about/guidelines.xhtml

Restricted - مقيد

completed all their coaching sessions (adherence rate 100%), which met the predetermined progression criteria of adhering to ≥ 84%. Half of the participants took their sessions at the planned time as previously scheduled. However, health coaches have rescheduled different sessions for seven participants for different reasons. The rescheduled sessions varied between 1 to 2 sessions for each one of them. Out of 84 sessions, only eleven sessions have been rescheduled (13%). The reasons for the rescheduling were home/work/appointment conflicts (n = 6 sessions), travel (n=1 session), illness (n=3 sessions), and being busy (n=1 session). All participants started their coaching sessions at the same time on June 15th, 2021, except four participants who started later on July 1st, 2021. A Fifteen-day delay because their coach could not begin at that time for family issues. In addition, the same four participants had another delay between the fourth and fifth sessions due to the coach's college exams. This led them to finish their last session on September 23rd, 2021.

Data Collection Adherence:

Participants recruited for the study were invited to Al-Zulfi General Hospital on June 10th, 2021. During this visit, they were given consent forms and information sheets about the intervention, providing them with an opportunity to ask questions and discuss any concerns related to their participation. Furthermore, baseline data, including measures of HbA1c, blood pressure, BMI, weight, and waist circumference, were collected during this visit. Due to the restrictions imposed by COVID-19, all required measurements and paperwork were completed in a single visit. Participants were then asked to complete two electronic questionnaires, the Diabetes Self-Care Activities (SDSCA) scale, and the Self-efficacy Scale for Diabetes, during the hospital visit on June 10th, 2021. The baseline data collection was successfully completed by all 30 participants, yielding a 100% completion rate. The time taken to complete the questionnaires ranged from 7 to 12 minutes, with no participants reporting difficulties or issues in the completion process.

At the end of the intervention, participants were once again invited to the hospital to gather post-intervention data. This second data collection session was much like the first, where we again measured HbA1c, blood pressure, BMI, weight, and waist circumference. Participants were also asked to complete the initial two questionnaires, with the addition of a Likert-scale Satisfaction Questionnaire for the intervention group only. This additional questionnaire resulted in a slightly longer completion time, ranging from 9 to 15 minutes, due to the inclusion of open-ended questions and certain items requiring justifications or explanations for selected responses. Overall, all participants effectively completed the data collection process, demonstrating successful adherence to our study's data collection procedures.

Progression Criteria

The feasibility measurements and predetermined progression criteria yielded positive results. The screening rate was 90%, exceeding the target of 80%, suggesting proceeding to the future definitive RCT. The recruitment rate reached 79%, demonstrating robust engagement with the target population. The retention rate at three months was 97%, surpassing the required 83% rate. Baseline data collection adherence and intervention adherence both achieved 100%. Endpoint data collection adherence was 97.6%, with only one participant withdrawing before

For peer review only - http://bmjopen.bmj.com/site/about/guidelines.xhtml

Restricted - مقيد

the first session. Overall, these results support the decision to proceed to the future definitive RCT, indicating the feasibility and acceptability of the health coaching approach for individuals with T2DM in SA.

Sample Size

The sample size for this feasibility study was determined in consultation with a statistician and was guided by practical considerations as recommended by the CONSORT guidelines for feasibility trials (29). Our aim was not to test the intervention for statistical significance but rather to assess the feasibility and variability of outcomes to inform the planning of a definitive RCT. The choice of sample size was also influenced by the need to manage resources efficiently and to ensure a broad enough representation of the target population to capture initial insights into the intervention's acceptability and implementation.

Furthermore, the statistician advised that for the upcoming main trial, a sample size calculation should be based on a clinically significant mean difference of 0.5% and a standard deviation of 1.4, given the substantial effect size of -0.93 observed in this feasibility study. With these parameters, using a power of 0.8 and an alpha level of 0.05, we estimated that 125 participants per group would be necessary. This sample size would not only allow us to detect a clinically significant difference but also accommodate subgroup analyses to explore variations in effectiveness across different settings or patient groups.

Acceptability of the Intervention

Acceptability and suitability of the intervention were assessed through a questionnaire (Likert-scale Satisfaction Questionnaire, 14 items) for the intervention group (n = 14). Using a Likert scale of 0 (not at all) to 10, most participants' responses show that the intervention affected their behaviour "quite a bit " (mean: 8.2 (SD= 2.3)). Participants replied, “very much” (mean 9.2 (SD= 1.4)) when asked how much participation in the intervention helped them establish a personal vision of wellness. When asked how the intervention boosted their confidence in taking actions toward improved well-being, the overall response was "very much" (mean 9.2 (SD= 1.4)). Their motives for making efforts toward enhanced well-being were "quite a bit" (mean 8.3 (SD= 1)). The average response to the usage of goal setting was "very much" (mean 9.2. (SD= 0.9)). Whereas their mean response to their use of problem-solving abilities was "quite a bit" (mean 8.6 (SD= 1.4)). Participants shared that the intervention greatly assisted them in overcoming obstacles and achieving increased levels of wellness, with a high mean score of 9 (SD= 1.1) reported. From the first through the eighth question, participants' responses are presented in Supplemental Table 7.

Participants were asked three "Yes" or "No" questions, and they all replied "Yes" when asked whether they expected to continue making improvements (n=14, 100%). The majority (n=13, 92.9%) responded with "Yes" the intervention met their expectations. All 14 participants who were asked whether they would recommend the intervention to others responded with a positive "yes".

For peer review only - http://bmjopen.bmj.com/site/about/guidelines.xhtml

Restricted - مقيد

Thematic Analysis Findings

The thematic analysis of the qualitative interviews and focus groups underscored the intervention's positive reception, with emergent themes revealing enhanced self-efficacy, increased motivation, and the supportive dynamics of the health coaching relationship, which participants found to be a significant aspect of the intervention's acceptability. We analysed 1691 minutes of coaching sessions to identify the BCTs used (see Supplemental Table 8) and transcribed 497 minutes of focus groups and interviews. A native Arabic speaker confirmed the accuracy of the entire transcription. To further ensure validity, about 10% of the transcription (50 minutes) underwent a back-translation process checked by a professional native speaker. Throughout the intervention, the first author (A.N.A), acting as the researcher, maintained field notes. Due to COVID-19 limitations, all interviews and focus groups were conducted online.

Evaluation of Participants and Health Coaches' Experiences

Various qualitative methods were used to assess participant experiences during the intervention, including interviews, focus groups, and field notes. After the intervention, two focus groups took place. Participants were given time to understand the questions before engaging in interviews and focus groups, fostering a deeper discussion. The researcher (A.N.A) conducted all focus groups and interviews, enabling active interaction and understanding of participant perspectives.

Between June 15th and September 23rd, 2021, 14 semi-structured interviews were conducted with seven participants (4 males, three females) from the intervention group, both pre-and post-study. Participants predominantly had a high school education or less, and the median age was 59. Three had long-standing diabetes of over ten years, three had a recent diagnosis of less than three years, and one was uncertain of the duration. Six of the seven were on diabetes medication.

Two post-study focus groups were conducted online. One focus group comprised six participants (three males and three females) from the intervention group with a median age of 52.5 years. The majority had a high school education or less, and most were on diabetes medication with a mix of recent and long-term diagnoses. The demographic characteristics of the participants can be seen in Table 2.

Three female health coaches from KFMC in Riyadh formed the second focus group to share their intervention delivery experiences. All had health coaching backgrounds, with two holding master's degrees in health education. The session lasted 265 minutes.

Table 2: Participants' Demographic characteristics

		Qualitative interview participants (n=7)		Focus group participants (n=6)	
Demographic characteristics		Number	%	Number	%
Gender	Male	4	57.1	3	50
	Female	3	42.9	3	50

For peer review only - http://bmjopen.bmj.com/site/about/guidelines.xhtml
Restricted - مقيد

Age	Years	43 to 62	Median 59	34 to 60	Median 52.5
Marital status	Married	7	100	6	100
Monthly income	less than 5000	1	14.3	2	33.3
	5000-10000	1	14.3	1	16.7
	10000-15000	1	14.3	0	
	Prefer not to declare	4	57.1	3	50
Education level	Primary school	2	28.6	1	16.7
	Secondary school	2	28.6	1	16.7
	High school	2	28.6	3	50
	Bachelor's degree	1	14.3	1	16.7
Since when you were diagnosed with Type 2 diabetes	less than a year	2	28.6	1	16.7
	1-3	1	14.3	3	50
	more than ten years	3	42.9	1	16.7
	do not know	1	14.3	1	16.7
Do you use diabetes medications?	Yes	6	85.7	5	83.3
	No	1	14.3	1	16.7

For peer review only

We used thematic analysis with inductive and deductive coding to evaluate the trial's acceptability and feasibility. Open coding initially identified emergent themes, with deductive coding following, grounded in qualitative research guidelines and the Medical Research Council (MRC) framework (30).

The analysis of translated transcripts produced four key themes, two of which were predetermined. The first theme pertained to trial design, conduct, and processes, divided into four sub-themes covering different aspects of intervention implementation. The second theme focused on intervention content and delivery, consisting of sub-themes on intervention components, perceived consequences, feasibility and acceptability in practice, and reach and dose. The third theme, intervention mechanism, encompassed sub-themes around COM-B model application, use of BCTs and intervention curriculum, and intervention impact. The fourth theme, future RCT, captured participant and coach suggestions. The thematic map used in the analysis is outlined in Supplemental Figure 2.

Theme 1: Intervention Design, Conduct, and Processes

Restricted - مقيد

This theme primarily focuses on the implementation and design of the intervention, along with the processes involved. Recruitment challenges during the pandemic prompted a shift to remote settings. This transition not only made recruitment a challenging task but also stretched the recruitment period beyond the planned timeframe.

Notably, a health coach stated that the recruitment phase was complicated as clinics moved online., *"...the recruitment phase, it was complicated only because all clinics become online and we could not see people with type 2 diabetes come to the hospital as usual, so the chance of meeting those people was very rare."* (Focus Group with Coaches, FGC01, Female).

Misunderstandings about health coaching in SA arose from a lack of understanding and familiarity. This highlighted the need for improved communication with recruiters, as one health coach observed, *"...from my communication with recruiters, some of them did not understand, so participants may get a wrong idea"* (Focus Group with Coaches, FGC01, Female).

Limited hospital access due to public health guidelines complicated recruitment further. However, the suggestion to recruit from a secondary hospital made the recruitment process manageable. Participants and health coaches recommended physicians as intermediaries for patient recruitment due to the trust and rapport they have established with patients; as one of them stated, *".....I think one of the best and most reliable ways to reach and recruit patients is to start from their direct physicians by suggesting the program for patients and encourage them to join"* (Focus Group with Coaches, FGC02, Female).

Proposing clear communication about program expectations aimed to reduce dropout rates, with one participant suggesting to *"ask participants to sign a contract to commit and if you are hesitating or you have the intention to withdraw to tell us [referred to program provider] at the beginning to give the opportunity to someone who needs it"* (Focus Group with Participants, P03, Female).

Health coaches and participants expressed general satisfaction with the intervention's design, and they believed that it effectively accommodated the local context of Saudi society. One participant expressed, *"I'm very very very satisfied and I think the program is easy to follow and very acceptable as it gradually improves different skills which last with a patient after the program…it is completely different from other programs.."* (Interview with Participants, P13, Male).

Additional workload challenged health coaches, mixing intervention tasks with regular duties. The COVID-19 pandemic further complicated matters by requiring greater flexibility in scheduling coaching sessions, particularly for participants with children engaged in online learning. To alleviate these issues, health coaches proposed securing formal workplace approval to dedicate full-time to the intervention. As one health coach noted, *"As you know and because of the workload, I was thinking of withdrawing from the intervention...... I think it is imperative to work a full-time job as a health coach in the intervention, not as a volunteer, and get formal permission from my work on this.."* (Focus Group with Coaches, FGC01, Female).

Restricted - مقيد

For peer review only

Theme 2: Intervention Content and Delivery

This theme encompassed multiple sub-themes reflecting on the intervention's implementation, including its acceptability and adaptability within the Saudi context, potential unintended consequences, the feasibility of the practical application, and intervention reach and dosage.

Health coaches suggested modifications for better acceptability and suitability of the intervention to the Saudi context. Emphasising the need for professionalism and structure, they proposed using formal platforms for communication, as one coach stated, *"...communication with the participant should be through a formal platform or application... to make the program more formal and organised..."* (Focus Group with Coaches, C02, Female). They also suggested the importance of physical meetings or video calls for a more serious engagement, as expressed by another coach, *"I prefer to have a physical meeting where I can sit with the patient face-to-face…"* (Focus Group with Coaches, FGC01, Female).

Coaches highlighted the necessity of prepared private spaces for coaching sessions. to give a professional image to the intervention. As one coach expressed, *"..As a coach, there should be a certain prepared private place at work to conduct all of my coaching sessions.."* (Focus Group with Coaches, C02, Female).

Participants preferred visual coaching sessions over phone calls for effective communication. Coaches felt that seeing the participant was vital for effective communication and coaching. A health coach expressed this, saying, *"...visual interaction with participants is very very important, you know the importance of reading and understanding the body language for me as a coach.."* (Focus Group with Coaches, C03, Female).

Participants praised the intervention for its straightforwardness and effectiveness in helping them make progress towards their goals. Some suggested a desire for additional sessions to further adopt and maintain new habits. One participant commented, *"I think three months was enough"* (Interview with Participants, P03, Female), *while another remarked, "…it enabled me to stay committed, continue, and achieve my goals"* (Interview with Participants, P09, Male).

Participants appreciated the use of health coaching techniques, such as action plans and SMART goals, which helped set achievable targets and keep them motivated. The flexibility and graduality of the program were also well-received, as one participant mentioned, *"...the most thing I liked about the program was the graduality to achieve goals .."* (Interview with Participants, P13, Male).

Coaches confirmed the intervention content's acceptability and observed its positive impacts. They also suggested the addition of monthly sessions or an extension of the intervention to six months for better patient progress monitoring and maintenance. A coach stated, *"… I suggest the intervention last longer… I think it would be good if the intervention lasts six months with a session each month …"* (Focus Group with Coaches, FGC01, Female).

Theme 3: Intervention Mechanism

This theme emphasises the practical application and effects of the COM-B health coaching model. Coaches received specialised training on the curriculum, and BCTs, utilising several

For peer review only - http://bmjopen.bmj.com/site/about/guidelines.xhtml

Restricted - مقيد

tools for efficient delivery. Notably, coaches documented the BCTs employed in each session, enabling tailored strategies to target specific behaviours. Participants appreciated this approach, stating, *"The BCTs helped me during each session to address some behaviours…"* (Focus Group with Coaches, FGC01, Female).

The COM-B model played a crucial role in the intervention, targeting four behaviours. Participants noted marked improvements in their knowledge and motivation pre-and post-intervention. Testimonials showed that they were more knowledgeable about their condition, diet, and physical activity. One participant said, *"I became more knowledgeable...now I know diet types and what carbohydrates mean…"* (Focus Group with Participants, P05, Female).

The intervention also impacted participants' social and physical environment, leading to a supportive atmosphere for behavioural change. Participants indicated that the changing social environment in SA encouraged the adoption of a healthier lifestyle and influenced their social networks. As one participant put it, *"While I was changing some of my old habits, my social network adapted..."* (Interview with Participants, P03, Female).

Health coaching provided a more personalised approach than traditional care, fostering motivation and leading to a change in participants' views about their condition. This motivation was further fuelled by the noticeable changes in outcomes such as HbA1c levels. Participants recognised the power of lifestyle changes and felt empowered by their improved health results, stating, *"...after the intervention, I realised modifying the lifestyle is the actual treatment..."* (Interview with Participants, P13, Male). Consequently, there were requests to extend the intervention, given the observable impacts, including reductions in A1c levels and body weight.

Participants in SA observed notable differences between the novel concept of health coaching and traditional care. The former was seen as superior, with participants expressing dissatisfaction with traditional care's impersonal nature. Health coaching, in contrast, provided a more personalised approach, allowing patients to voice their concerns. As one participant expressed, *"I would choose this program without hesitation; at least I finish each session while I'm comfortable..."* (Interview with Participants, P03, Female).

Traditional care was perceived as a top-down process, with patients passively receiving pre-prepared information or plans. The health coaching intervention, however, positioned participants as active stakeholders in their own care by encouraging goal-setting and plan creation. A participant noted, *"Your program helped me use new skills such as setting plans and having short-term and long-term goals..."* (Interview with Participants, P08, Male).

Frustration and even anger marked participants' feelings about traditional care, citing time constraints, a lack of empathy, and the expense of private consultations. Health coaching was seen as a way to alleviate these issues, with participants suggesting the incorporation of health coaching clinics in hospitals to facilitate meaningful patient discussions and foster better disease self-management. A participant suggested, *"I think it is important to recruit a coach in the hospital... 90% of patients with chronic diseases need to sit with someone to have meaningful discussions..."* (Interview with Participants, P08, Male).

Theme 4: Improvement Opportunities in the Future Health Coaching RCT

For peer review only - http://bmjopen.bmj.com/site/about/guidelines.xhtml
Restricted - مقيد

As the inaugural health coaching intervention within the Saudi healthcare system, this initiative stands as a landmark reflecting the strategic direction of the Saudi Ministry of Health towards more proactive, patient-centred care. In anticipation of scaling up such interventions, participants and health coaches provided constructive feedback for refinement. Participants expressed a preference for more frequent coaching interactions and the opportunity for extended face-to-face engagements, suggesting a deeper and more personal connection could enhance the intervention's impact. "*Having more time and direct sessions would greatly benefit the experience*" shared one participant (Interview with Participants, P01, Female).

Health coaches recommended the integration of multidisciplinary expertise, particularly dietitians and physicians, to provide comprehensive care and address the complex needs of diabetes management. "*Incorporating a nutritionist and a physician would offer a more rounded approach to patient care,"* proposed a coach (Focus Group with Coaches, C03, Female). This suggestion is indicative of a holistic strategy, recognizing the multifactorial nature of diabetes.

To bolster participant engagement, the provision of incentives was suggested, alongside enhanced access to healthcare services. Additionally, health coaches proposed the creation of educational content, such as a succinct video delineating the health coaching concept, to facilitate a clearer understanding for future participants. "*A visual introduction to health coaching might bridge the initial knowledge gap,*" a coach highlighted (Focus Group with Coaches, C02, Female).

Preliminary Effects of the Health Coaching Intervention

Our quantitative analysis, using a linear regression model adjusted for baseline levels, revealed significant health improvements from the health coaching intervention. Specifically, HbA1c levels in the intervention group decreased significantly, with a mean difference of -1.86 (95% CI: -2.71 to -1.01, P<0.001) compared to the control group. Secondary outcomes further underscored the intervention's potential efficacy; BMI decreased by a mean of -1.02 (95% CI: -2.01 to -0.041, P=0.042), and waist circumference reduced by -6.89 (95% CI: -10.17 to -3.61, P<0.001). While changes in Mean Arterial Pressure (MAP) and weight were observed, they did not reach statistical significance (MAP mean difference: -0.68, 95% CI: -7.46 to 6.09, P=0.83; weight mean difference: -2.58, 95% CI: -5.25 to 0.082, P=0.057). Detailed outcomes, including improvements in patients' self-efficacy and diabetes self-management, are delineated in Tables 3 and 4.

Table 3: Groups means and SDs at study baseline and endpoint.

	Group	N	Mean	Std. Deviation

For peer review only - http://bmjopen.bmj.com/site/about/guidelines.xhtml

Restricted - مقيد

A1c level at the study baseline	Intervention Group	14	9.12	2.50
	Control Group	15	8.73	1.69
A1c level at the study endpoint	Intervention Group	14	7.29	1.38
	Control Group	15	8.93	1.81
Mean Arterial Pressure at the study baseline	Intervention Group	14	91.7	13.56
	Control Group	15	86.73	11.66
Mean Arterial Pressure at the study endpoint	Intervention Group	14	92.57	9.11
	Control Group	15	90.33	12.91
Weight in Kg at the study baseline	Intervention Group	14	83.50	11.97
	Control Group	15	81.21	12.76
Weight in Kg at the study endpoint	Intervention Group	14	80.70	10.80
	Control Group	15	81.21	12.60
BMI at the study baseline	Intervention Group	14	30.39	4.57
	Control Group	15	30.87	5.43
BMI at the study endpoint	Intervention Group	14	29.39	4.33
	Control Group	15	30.86	5.318
Waist Circumference (cm) at the study baseline	Intervention Group	14	109.35	13.60
	Control Group	15	106.66	10.22
Waist Circumference (cm) at the study endpoint	Intervention Group	14	102.21	12.55
	Control Group	15	106.76	9.91

only

Table 4: Outcomes mean differences taking into account the baseline value regression.

For peer review only - http://bmjopen.bmj.com/site/about/guidelines.xhtml

Restricted - مقيد

Endpoint HbA1c	Mean difference	t	p-value	*d*	[95% CI]	
Group*	-1.864463	-4.50	<0.001	-0.93	-2.716065	-1.012862
Baseline HbA1c	.566714		<0.001		.3601448	.7732833
Endpoint MAP	Mean difference	t	p-value		[95% CI]	
Group*	-.6833264	-0.21	0.837		-7.463123	6.09647
Baseline MAP	.5809645	4.38	<0.001		.3083653	.8535638
Endpoint Weight	Mean difference	t	p-value		[95% CI]	
Group	-2.584974	-1.99	0.057		-5.252461	.0825133
Baseline Weight T1	.9090889	16.84	<0.001		.7981218	1.020056
Endpoint BMI	Mean difference	t	p-value		[95% CI]	
Group	-1.024214	-2.14	0.042		-2.006754	-.0416741
Baseline BMI T1	.9322785	19.02	<0.001		.8315092	1.033048
Endpoint WC	Mean difference	t	p-value		[95% CI]	
Group	-6.895251	-4.32	<0.001		-10.17924	-3.611259
Baseline WC	.8732792	12.72	<0.001		.7322131	1.014345

Abbreviations: HbA1c: Haemoglobin A1c, MAP: Mean Arterial Pressure, BMI: Body Mass Index, WC: Waist Circumference, t: t-test, CI: Confidence Interval, d: Cohen's d (Effect size) *(Group = Intervention mean – Control mean)

Patients' Self-Efficacy Questionnaire

The self-efficacy of patients was assessed using an 8-item scale (31), where scores ranged from 1 (least confident) to 10 (most confident). Higher mean scores indicated greater self-efficacy. Both intervention and control groups displayed an increased average response from baseline to the study endpoint, with higher improvements observed in the intervention group (see Supplemental Table 9). The groups exhibited a statistically significant difference (P = .006, 59 % CI .713 to 3.839) in confidence related to controlling their condition and maintaining exercise. Similarly, a significant difference (P = .006, 59 % CI 0.82 to 4.35) was noted in

For peer review only - http://bmjopen.bmj.com/site/about/guidelines.xhtml

Restricted - مقيد

confidence regarding appropriate food choices when hungry. This suggests the intervention group saw greater improvements in self-efficacy compared to the control group.

Diabetes Self-Care Activity

The Diabetes Self-Care Activity scale (32), scoring 0 (no adherence) to 7 (complete adherence), measured patients' adherence to specific activities over the past week. Average scores showed overall improvements in self-care for both the intervention and control groups across all activities, with the intervention group often meeting or exceeding American Diabetes Association recommendations (see Supplemental Table 10) (33). For instance, adherence to a healthy eating plan in the intervention group increased from an average of 1.64 to 5.00 days per week. Physical exercise also increased in both groups, with a larger improvement in the intervention group (3.57 to 5.64 days). Similarly, there was improved management of carbohydrate intake in the intervention group, from 1.71 to 4.00 days per week.

Integration Results:

The key findings from both quantitative and qualitative data, aligned with research objectives, were assembled in a joint display table (see Supplemental Table 4). This table was structured to present the study's critical concepts and the corresponding progression criteria, which provide a comprehensive understanding of the mixed-methods findings. The phrase 'there is no significance in moving forward', as used within our progression criteria, draws from the principle of setting clear benchmarks for feasibility studies. These benchmarks are pivotal in assessing whether a study exhibits the potential for upscaling to a full-scale RCT. They embody a balance between the ambition to proceed and the pragmatism to address early issues, as recommended by best practices in trial management (34). In our context, this phrase indicates that if participant recruitment, retention, or adherence fall below the predetermined percentages, the study may not have the robustness required for expansion. Such a stance encourages rigorous scrutiny of recruitment rates and intervention adherence to ensure only well-substantiated research advances.

The integration of quantitative and qualitative data insights offered enhanced comprehension of findings collated and analysed separately. This approach bolstered the credibility and transparency of the overall results through data triangulation, allowing for comparison and validation of findings that a single method wouldn't permit. The integration was critical to comprehensively understand the impacts of our intervention. Qualitative insights not only complemented the quantitative data but also provided deeper explanations for the patterns observed in numerical outcomes. For instance, while quantitative data showed significant improvements in HbA1c levels and BMI, qualitative findings offered nuanced insights into how and why participants made specific behavioural changes. Participants frequently cited the personalised feedback and motivational support from health coaches as pivotal, which helped them to adhere more consistently to their management plans. The integrated results substantiated the pertinence of mixed approaches, essential to the study's feasibility and acceptability exploration in the Saudi context for a large-scale intervention with T2DM patients.

The study reported high screening, recruitment, and retention rates. These results were corroborated by the integrated analysis, suggesting a promising potential for the study within

For peer review only - http://bmjopen.bmj.com/site/about/guidelines.xhtml

Restricted - مقيد

For peer review only

the Saudi setting. This was further supported by participants' willingness to engage in the intervention, extend invitations to family members, and their adherence to complete the intervention, despite some rescheduled sessions.

Notably, improvements were observed across five out of seven preliminary outcomes, which were validated by qualitative data. Participants and coaches reported positive experiences with the intervention's usability, design, and content. Moreover, qualitative results echoed the high satisfaction levels among participants identified in the quantitative Likert-scale Satisfaction Questionnaire. Despite the challenge posed by session rescheduling flexibility for coaches, it facilitated participant adherence, as supported by quantitative data: all participants completed their sessions with only eleven sessions rescheduled.

Discussion:

The findings from this feasibility study endorse advancing towards a larger RCT for a health coaching intervention among patients with T2DM in SA. Notably, the high rates of eligibility, recruitment, and retention reflect the study's effectiveness in engaging participants, which is pivotal for ensuring the representativeness and reliability of the results.

Achieving a recruitment rate of 79% is particularly significant; it not only indicates the interest and willingness of the target population to participate but also reinforces the potential for this health coaching intervention to be scaled up and applied broadly within the Saudi healthcare setting. This recruitment performance, especially when contrasted with rates from prior studies, such as those by Basak C & Schou (2013) and Cho et al. (2011) (35-37), highlights the tailored approach's resonance with the participants even amidst the operational challenges presented by the COVID-19 pandemic.

Retention is another key indicator of a study's appeal and the practicality of its intervention. The retention rate in this study exceeded that of a similar study (83.6%) (35), as well as the rates reported by Basak C & Schou (2013) (87%) (37) and Cho et al. (2011) (90%).(36) Our retention rate, which remained high throughout the study period, suggests that the intervention was well-received and retained its relevance and value to the participants, motivating them to continue through the study's conclusion. The flexibility allowed in rescheduling coaching sessions contributed to this success, though it did increase the workload for the coaches, an aspect to consider in the planning of future RCTs.

As we reflect on the integrated quantitative and qualitative findings, we see confirmation of the feasibility of conducting a larger RCT, despite restrictions due to COVID-19. The comprehensive data collection, appropriate outcome assessments, and participant adherence collectively point to the potential for effective execution and data gathering in a subsequent, more extensive RCT. Enhancing reach and diversity through a variety of recruitment methods, and the use of a concise explanatory video to describe the intervention, are strategies that can further streamline the process and solidify understanding among future participants.

The feasibility study utilised the COM-B model and the BCTs taxonomy, which proved to be effective in guiding intervention content and addressing participant barriers. The most frequently used BCTs aligned with previous literature. The study adapted to telephone-based

For peer review only - http://bmjopen.bmj.com/site/about/guidelines.xhtml

Restricted - مقيد

sessions due to COVID-19 restrictions, but a mixed approach with face-to-face interactions was preferred by both participants and coaches. A longer intervention duration of up to six months with more face-to-face sessions was recommended to ensure sustained behaviour changes.

The intervention's positive outcomes, particularly the reduction in HbA1c levels, align with evidence suggesting that interventions emphasizing HbA1c as the primary outcome are more effective. This underscores the importance of selecting outcomes aligned with the intervention's central goal. This study supports previous findings (38), indicating that using HbA1c as the primary intervention outcome can enhance effectiveness. Emphasizing glycaemic control as the primary outcome significantly impacted participants' diabetes self-management in this study. Participants actively developed and utilized key skills to manage their condition, indicating the efficacy of the coaching model. Their satisfaction was notably linked to a meaningful reduction in HbA1c levels after the intervention, emphasizing the holistic impact of the health coaching model.

Although feasibility studies are not formally powered to detect conclusive effects on interventions (39), this study demonstrated the high acceptability and usability of the intervention in the Saudi context. However, concerns were raised by health coaches regarding their volunteer roles and the challenges of finding suitable locations for coaching sessions. These issues need to be addressed in planning for the larger RCT. The ongoing societal changes in SA, along with participants' satisfaction with the intervention, suggest potential acceptance for a large-scale RCT.

For the upcoming definitive RCT, it's crucial to integrate participant and coach feedback to refine the approach. Recommendations include lengthening the program to six months with increased in-person interactions, involving multidisciplinary experts, regular coach coordination, participant incentives, and a brief explanatory video to introduce health coaching.

This study introduces a patient-cantered health coaching approach to Saudi Arabia, showcasing its effectiveness in managing T2DM and its potential applicability to other chronic conditions. The use of a detailed curriculum and the COM-B model has proven beneficial, supporting the Saudi Ministry of Health's move towards behaviour-based strategies and paving the way for broader implementation.

The study's positive reception and outcomes reinforce the viability of health coaching in T2DM management, offering insights for future research and substantiating its growing acceptance and practicality.

Strengths and Limitations of this Study

This study applied the BCW framework, proving beneficial for a comprehensive understanding of the issue of T2DM in the context of SA through an in-depth analysis of prior literature. Accurate diagnosis of the identified problem, considering potential barriers and facilitators, plays a pivotal role in the systematic roadmap. This process initiates with recognizing the actual issue and concludes with specifying target behaviours necessary to achieve the intended goals. Employing the COM-B model, we conducted a narrative review to pinpoint facilitators and

For peer review only
Restricted - مقيد

barriers in controlling T2DM. The unique approach involved employing the BCW, COM-B model, and BCTs taxonomy, enabling a meticulous analysis of each health coaching session and the coding of each applied BCT. This resulted in a clear and detailed description of the intervention.

The inclusion of progression criteria, as recommended by the CONSORT 2010 statement, aided in decision-making for the larger trial (40). The study findings, including the effect size and retention rate, will inform the estimation of the sample size for the future main trial. The study also utilised both the Summary of Diabetes Self-Care Activity questionnaire and the Self-efficacy Scale for Diabetes scale, providing a comprehensive assessment of health coaching's impact. A mixed methods approach was employed to gather diverse perspectives, enhance validity and reliability, and produce more comprehensive findings.

However, the study is not without limitations, particularly concerning generalizability due to the specific setting and population. The small sample size may have led to potential Type 2 errors, limiting the study's ability to detect certain effects. While the recruitment of the intervention sample from a public hospital may not be directly applicable to primary care settings, it could impact the broader generalizability of findings to other individuals.

It is essential to note that although statistically significant effects were detected, the study's acknowledgement of limitations in statistical power reflects an awareness of potential constraints in identifying smaller yet existing effects. These limitations should be considered when interpreting the study's outcomes.

Conclusion

This mixed methods feasibility RCT demonstrated the feasibility and acceptability of implementing a health coaching approach for individuals with T2DM in SA. The integration of qualitative and quantitative findings yielded positive outcomes, providing strong support for conducting a larger RCT to further investigate the effectiveness of health coaching in enhancing diabetes self-management among individuals with T2DM in SA. Importantly, this study offers valuable insights for future trials, particularly in terms of incorporating the COM-B model and BCTs taxonomy into the health coaching approach. The positive feedback received for implementing a patient-centred approach indicates that it could help manage other chronic diseases. This aligns with the Saudi Arabian Ministry of Health's behaviour-focused strategies, providing more evidence to support the expansion of health coaching. As a result, these insights have significant implications for the current management practices of T2DM and beyond. Future research should also explore the social and environmental impacts of Vision 2030, the national vision of SA, on diabetes self-management. By considering contextual factors that may influence the implementation and outcomes of health coaching interventions for diabetes management in SA, further investigations will deepen our understanding and inform strategies to improve diabetes care and other chronic diseases in the country.

For peer review only - http://bmjopen.bmj.com/site/about/guidelines.xhtml

Restricted - مقيد

For peer review only

Supplementary Figure:

Supplementary Figure 1: CONSORT Flow Diagram of Participant Progress through the Phases of the Randomised Trial. This diagram depicts the enrolment, allocation, follow-up, and analysis stages of participants. It includes the number of individuals assessed for eligibility, excluded, or randomised into the trial, as well as those who received the intervention, followed up, and were included in the analysis.

Supplementary Figure 2: Detailed Thematic Analysis of Intervention Components. This map illustrates the comprehensive thematic breakdown of the intervention, highlighting key areas such as feasibility, mechanisms, and potential improvements. It details the sub-themes of intervention design, content delivery, and participant engagement, as well as the impact on trial staff and researchers. Suggestions for the future RCT based on coaches' and participants' feedback are also included.

For peer review only

Restricted - مقيد

Author Contributions: A.N.A. was responsible for the study's conception and design, data acquisition, analysis, and interpretation, as well as the drafting the manuscript. E.G. contributed to the critical revision of the manuscript based on data analysis. AA, AM, MA, A.S.A were responsible for the implementation of the intervention. All authors have thoroughly reviewed and approved the final version of the manuscript for publication

Funding: This study received no external funding.

Competing interests: The authors declare no conflicts of interest.

Patient consent for publication: Not required.

Ethics approval: Ethical approval for this study was granted by both the University of Sheffield and the Institutional Review Board (IRB) committee at King Fahad Medical City (KFMC), with the assigned IRB Log Number: 21-062E.

Data Availability Statement: All data generated or analysed during this study are available upon reasonable request from the corresponding author.

Acknowledgement: We express our sincere gratitude to the study participants for their valuable time and willingness to participate. We are also grateful to KFMC and Al-Zulfi General Hospital for granting permission to conduct the intervention. Furthermore, we extend our thanks to the University of Sheffield Institutional Open Access Fund for their support towards this research. Finally, we would like to acknowledge that this paper will be published under a Creative Commons Attribution (CC BY) license.

For peer review only

Restricted - مقيد

References

1. Alqurashi KA, Aljabri KS, Bokhari SA. Prevalence of diabetes mellitus in a Saudi community. Annals of Saudi Medicine. 2011;31(1):19-23.
2. Association AD. Diagnosis and classification of diabetes mellitus. American Diabetes Association; 2010. p. S62-S.
3. Hex N, Bartlett C, Wright D, Taylor M, Varley D. Article: Health Economics Estimating the current and future costs of Type 1 and Type 2 diabetes in the UK, including direct health costs and indirect societal and productivity costs.
4. Alqahtani M, Almutairi FE, Albasseet AO, Almutairi KE. Knowledge, Attitude, and Practice of Diabetes Mellitus Among the Saudi Population in Riyadh, Saudi Arabia: A Quantitative Study. Cureus. 2020.
5. Organization WH. Noncommunicable diseases 2022 [Available from: https://www.who.int/news-room/fact-sheets/detail/noncommunicable-diseases.
6. Alneami YM, Coleman CL. Risk Factors for and Barriers to Control Type-2 Diabetes among Saudi Population. Global Journal of Health Science. 2016;8(9).
7. Alabdulbaqi D. A Cultural Adaptation of a Diabetes Self-Management Programme for Patients with Type 2 Diabetes Attending a Primary Health Care Centre in Saudi Arabia. 2019.
8. Al Slamah T, Nicholl BI, Alslail FY, Harris L, Melville CA, Kinnear D. Cultural adaptation of self-management of type 2 diabetes in Saudi Arabia (qualitative study). PLOS ONE. 2020;15(7):e0232904-e.
9. Wolever RQ, Dreusicke M, Fikkan J, Hawkins TV, Yeung S, Wakefield J, et al. Integrative Health Coaching for Patients With Type 2 Diabetes. The Diabetes Educator. 2010;36(4).
10. Wong-Rieger D, Rieger FP. Health coaching in diabetes: empowering patients to self-manage. Elsevier; 2013. p. 41-4.
11. Hill B, Richardson B, Skouteris H. Do we know how to design effective health coaching interventions: A systematic review of the state of the literature. American Journal of Health Promotion. 2015;29(5):e158-e68.
12. Pirbaglou M, Katz J, Motamed M, Pludwinski S, Walker K, Ritvo P. Personal Health Coaching as a Type 2 Diabetes Mellitus Self-Management Strategy: A Systematic Review and Meta-Analysis of Randomized Controlled Trials. SAGE Publications Inc.; 2018. p. 1613-26.
13. Hill B, Richardson B, Skouteris H. Do we know how to design effective health coaching interventions: a systematic review of the state of the literature. American journal of health promotion : AJHP. 2015;29(5):e158-68.
14. Alanazi FK, Alotaibi JS, Paliadelis P, Alqarawi N, Alsharari A, Albagawi B. Knowledge and awareness of diabetes mellitus and its risk factors in saudi arabia. Saudi Arabian Armed Forces Hospital; 2018. p. 981-9.
15. Eldridge SM, Chan CL, Campbell MJ, Bond CM, Hopewell S, Thabane L, et al. CONSORT 2010 statement: Extension to randomised pilot and feasibility trials. The BMJ. 2016;355.
16. Almulhim AN, Goyder E, Caton SJ. Assessing the Feasibility and Acceptability of Health Coaching as a New Diabetes Management Approach for the People with Type 2 Diabetes in Saudi Arabia: A Protocol for a Mixed Methods Feasibility Study. International Journal of Environmental Research and Public Health. 2022;19(22):15089.
17. Hariton E, Locascio JJ. Randomised controlled trials – the gold standard for effectiveness research: Study design: randomised controlled trials. Blackwell Publishing Ltd; 2018. p. 1716-.

For peer review only - http://bmjopen.bmj.com/site/about/guidelines.xhtml

Restricted - مقيد

18. Michie S, Ashford S, Sniehotta FF, Dombrowski SU, Bishop A, French DP. A refined taxonomy of behaviour change techniques to help people change their physical activity and healthy eating behaviours: The CALO-RE taxonomy. Psychology and Health. 2011;26(11):1479-98.
19. Michie S, van Stralen MM, West R. The behaviour change wheel: A new method for characterising and designing behaviour change interventions. Implementation Science. 2011;6(1):42-.
20. Dyson PA, Twenefour D, Breen C, Duncan A, Elvin E, Goff L, et al. Diabetes UK evidence-based nutrition guidelines for the prevention and management of diabetes. Diabetic Medicine. 2018;35(5):541-7.
21. Al Slamah T, Nicholl BI, Alslail FY, Melville CA. Self-management of type 2 diabetes in gulf cooperation council countries: A systematic review. PLOS ONE. 2017;12(12):e0189160-e.
22. UK D. diabetes risk and waist measurement: Diabetes UK; 2022 [Available from: https://www.diabetes.org.uk/diabetes-the-basics/types-of-diabetes/type-2/waist-measurement.
23. Cocks K, Torgerson DJ. Sample size calculations for pilot randomized trials: A confidence interval approach. Journal of Clinical Epidemiology. 2013;66(2):197-201.
24. Julious SA. Sample size of 12 per group rule of thumb for a pilot study. Pharmaceutical Statistics. 2005;4(4):287-91.
25. Braun V, Clarke V. Using thematic analysis in psychology. Qualitative Research in Psychology. 2006;3(2):77-101.
26. McCrudden MT, Marchand G, Schutz PA. Joint displays for mixed methods research in psychology. Methods in Psychology. 2021;5:100067.
27. Morgan DL. Commentary—After triangulation, what next? : Sage Publications Sage CA: Los Angeles, CA; 2019. p. 6-11.
28. Creswell JW, Clark VLP. Designing and conducting mixed methods research: Sage publications; 2017.
29. Eldridge SM, Chan CL, Campbell MJ, Bond CM, Hopewell S, Thabane L, et al. CONSORT 2010 statement: extension to randomised pilot and feasibility trials. bmj. 2016;355.
30. Moore GF, Audrey S, Barker M, Bond L, Bonell C, Hardeman W, et al. Process evaluation of complex interventions: Medical Research Council guidance. BMJ (Online). 2015;350.
31. BOOK QC. Stanford Patient Education Research Center. American Journal of Public Health. 1990;80:446-52.
32. Toobert DJ, Hampson SE, Glasgow RE. The summary of diabetes self-care activities measure: Results from 7 studies and a revised scale. Diabetes Care. 2000;23(7):943-50.
33. Association AD. Understanding A1C Diagnosis: ADA; 2022 [Available from: https://diabetes.org/diabetes/a1c/diagnosis.
34. Avery KN, Williamson PR, Gamble C, Francischetto EOC, Metcalfe C, Davidson P, et al. Informing efficient randomised controlled trials: exploration of challenges in developing progression criteria for internal pilot studies. BMJ open. 2017;7(2):e013537.
35. Kim MT, Kim KB, Huh B, Nguyen T, Han H-R, Bone LR, et al. The Effect of a Community-Based Self-Help Intervention. American Journal of Preventive Medicine. 2015;49(5).
36. Cho J-H, Kwon H-S, Kim H-S, Oh J-A, Yoon K-H. Effects on diabetes management of a health-care provider mediated, remote coaching system via a PDA-type glucometer and the Internet. Journal of Telemedicine and Telecare. 2011;17(7).
37. Basak Cinar A, Schou L. Health promotion for patients with diabetes: health coaching or formal health education? International Dental Journal. 2014;64(1).
38. Almulhim AN, Hartley H, Norman P, Caton SJ, Doğru OC, Goyder E. Behavioural Change Techniques in Health Coaching-Based Interventions for Type 2 Diabetes: A Systematic Review and Meta-Analysis. BMC Public Health. 2023;23(1):1-21.
39. Lee EC, Whitehead AL, Jacques RM, Julious SA. The statistical interpretation of pilot trials: should significance thresholds be reconsidered? BMC medical research methodology. 2014;14(1):1-8.

For peer review only

For peer review only - http://bmjopen.bmj.com/site/about/guidelines.xhtml

Restricted - مقيد

40. Schulz KF, Altman DG, Moher D. CONSORT 2010 Statement: Updated guidelines for reporting parallel group randomised trials. BMJ (Online). 2010;340(7748):698-702.

For peer review only

Restricted - مقيد

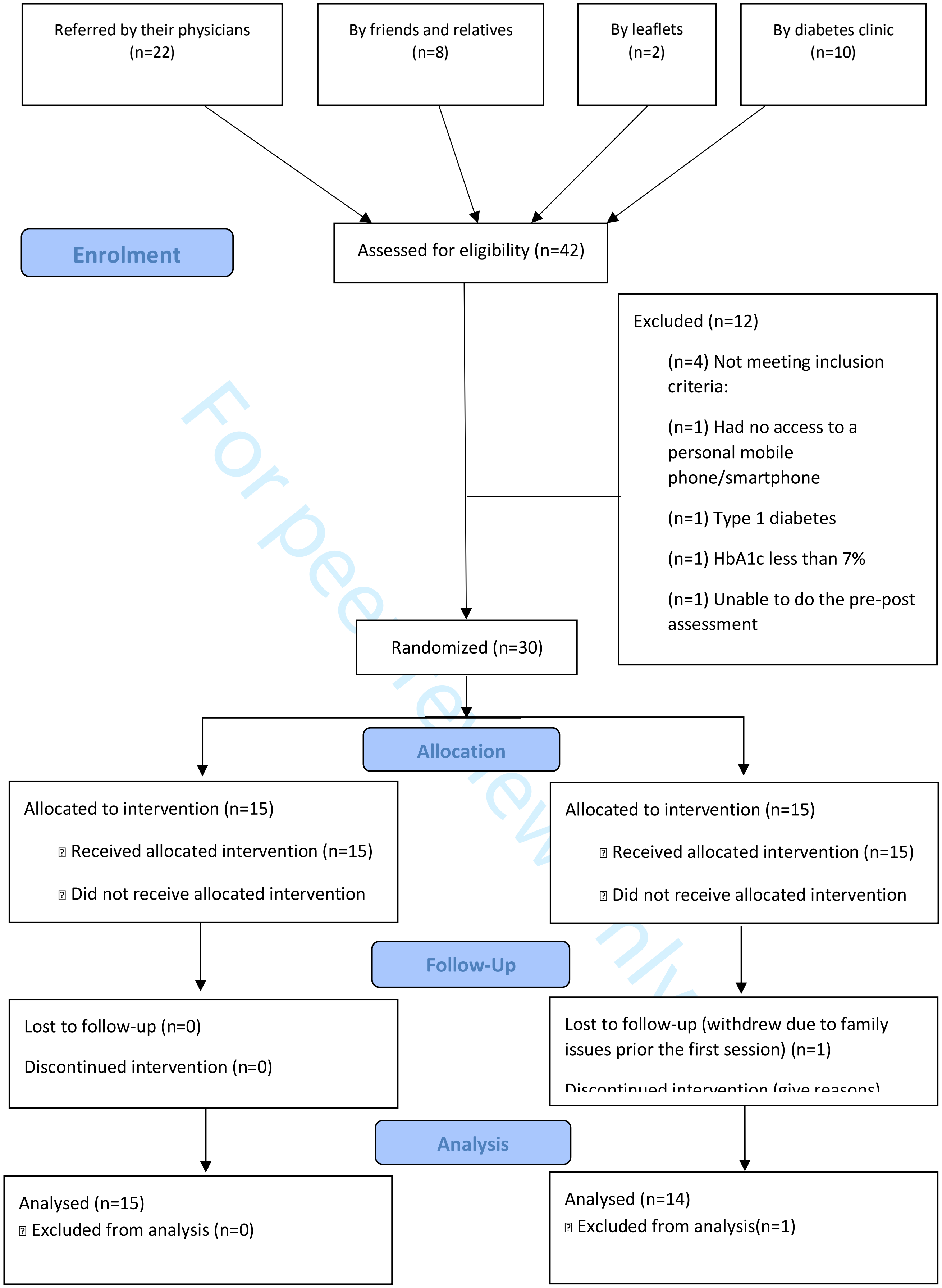

Figure 1: The study CONSORT flow chart

For peer review only

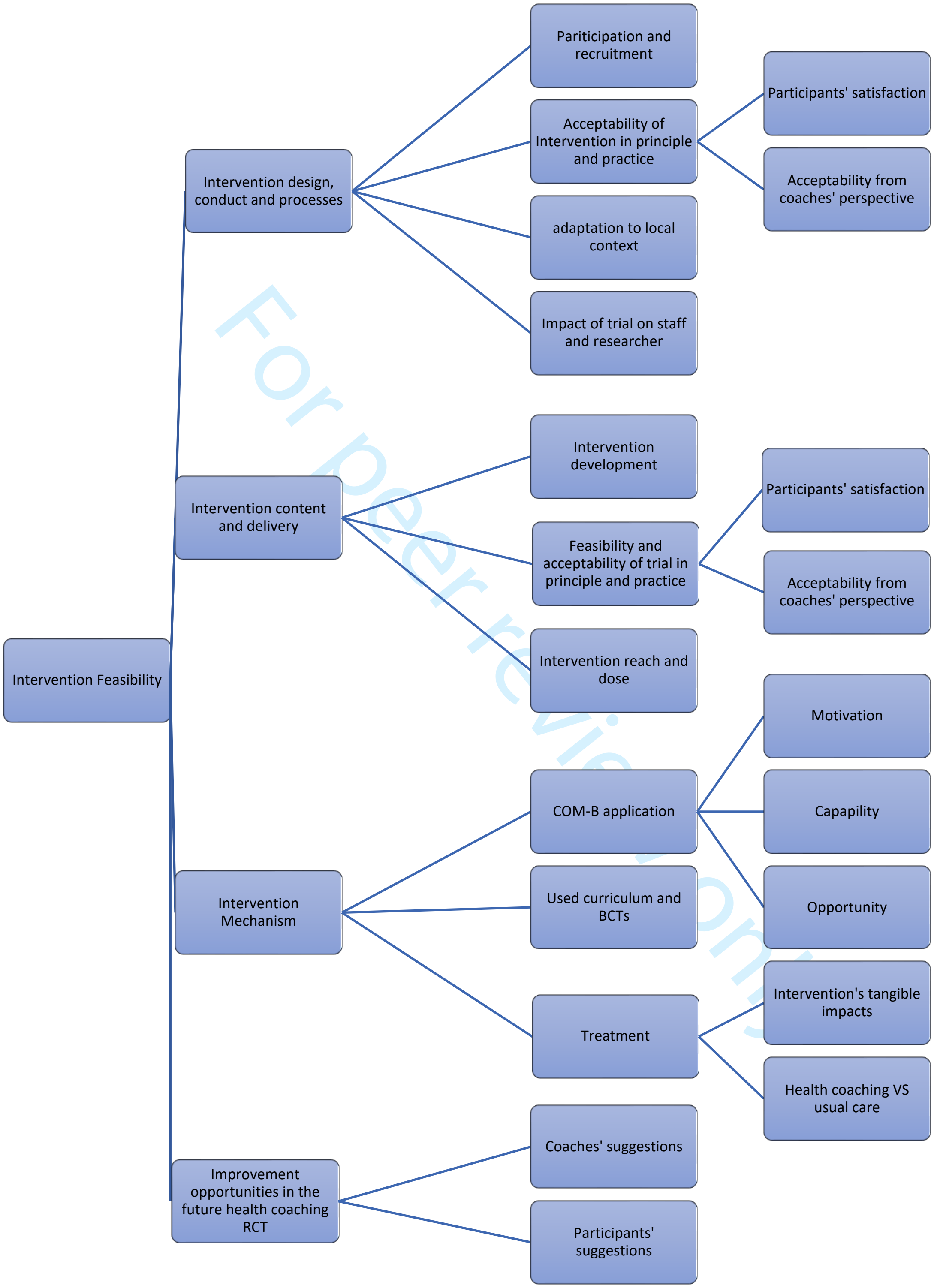

Figure 2: Thematic analysis map

Supplemental Table 1: link the pre-identified barriers to the Capability, Opportunity, Motivation-Behaviour (COM-B) model, the Theoretical Domains Framework (TDF), intervention functions and Behavioural Change Techniques (BCTs)

COM-B		TDF	Barrier	Intervention Function	BCTs
Capability	Psychological	Knowledge	Poor T2DM knowledge affect self-control<br>Poor nutrition knowledge (what the association between diabetes and diet, type of healthy food)<br>Poor knowledge about PA (how to engage in PA, how much time spend on PA, PA intensity, PA types, underestimate the role of PA	Education	5.1Information about health consequences
	Physical	Physical skills	Lack of the energy to do PA<br>Lack or limited skills of physical skills to do exercise	Enablement<br>Training	**Enablement:**<br>6.1Demonstration of the behaviour<br>1.5 Review behaviour goal (s)<br>1.7 Review outcome goal (s)<br>2.3 Self-monitoring of behaviour<br>12.1 Restructuring the physical environment<br>4.1 Instruction on how to perform a behaviour'<br>3.1 Social support (unspecified)<br>**Training:**<br>8.1 Behavioural practice/rehearsal<br>8.3 Habit Formation<br>8.4 Habit reversal<br>8.7 Graded tasks

Restricted - مقيد

COM-B		TDF	Barrier	Intervention Function	BCTs
					15.4 Self-talk<br>10.9 Self-reward<br>2.2 Feedback on behaviour<br>2.3 Self-monitoring of behaviour
Opportunity	Social	Social influences	Social norms & habits: overuse of high calories food intake; carbohydrates and fats, eating together (collectives), and social courtesy to eat unhealthy diet	Enablement	**Enablement**:<br>1.3 Goal setting (outcome)<br>1.1 Goal setting (Behaviour)<br>3.1 Social support (unspecified)<br>1.4 Action planning<br>1.2 Problem-solving<br>2.3 Self-monitoring of behaviour<br>8.7 Graded tasks<br>5.5 Anticipated regret<br>12.1 Restructuring the physical environment<br>12.2 Restructuring the social environment
	Physical	Environmental context and resources	Lack of time<br>Lack of resources (environmental, appropriate climate & financial ability)<br>Lack of access to do activity<br>Overuse of cars for transportations	Restriction<br>Environmental restructuring<br>Enablement<br>Modelling	**Enablement:**<br>1.3 Goal setting (outcome)<br>1.1 Goal setting (Behaviour)<br>3.1 Social support (unspecified)<br>1.4 Action planning<br>1.2 Problem-solving<br>2.3 Self-monitoring of behaviour

Restricted - مقيد

COM-B		TDF	Barrier	Intervention Function	BCTs
					9.2 Pros and cons<br>9.3 Comparative imagining of future outcomes<br>8.7 Graded tasks<br>1.9 Commitment<br>13.2 Framing/reframing<br>5.5 Anticipated regret<br>12.5 Adding objects to the environment<br>**Modelling:**<br>6.1 Demonstration of the behaviour<br>**Environmental restructuring:**<br>12.1 Restructuring the physical environment<br>7.1 Prompts/Cues<br>**Restriction:**<br>Use rules to reduce opportunity to engage in unwanted behaviour
Motivation	Reflective	Beliefs about own capability	Lack of willpower and self-confidence to do PA and maintain healthy diet	Persuasion<br>Education<br>Enablement	**Persuasion:**<br>15.1 Verbal persuasion about capability<br>15.2 Mental rehearsal of successful performance<br>9.1 Credible source<br>2.2 Feedback on behaviour<br>13.2 Framing/reframing<br>15.3 Focus on past success<br>**Education:**

Restricted - مقيد

COM-B		TDF	Barrier	Intervention Function	BCTs
					5.1 Information about health consequences<br>5.3 Information about social and environmental consequences<br>**Enablement:**<br>1.9 Commitment<br>5.5 Anticipated regret
		Beliefs consequences	Fear from consequences of PA (fear of injury and disease future complications)	Education	5.1 Information about health consequences
		Social role and identity	Struggle to change social identity associated with culture diet<br>Struggle to accept the fact of living with diabetes	Education<br>Persuasion	**Education**:<br>5.1 Information about health consequences<br>**Persuasion:**<br>13.5 Identity associated with changed behaviour

Restricted - مقيد

Supplemental Table 2: Summarise and map how the intervention links each behaviour target to specific intervention functions and Behavioural Change Techniques (BCTs)

COM-B		TDF coding included in this research	What needs to occur to bring about change?			
			Decrease carbohydrate intake in each meal	Use unsaturated fats as possible (avoid saturated fats)	Do exercise for 30 min, five days on a weekly basis	Monitor waist circumference
Capability	Psychological	Knowledge	Knowledge:<br>Understand food types (especially carbohydrate types, e.g., rice and dates)<br>Understand carbohydrate quantity targets<br>Understand alternative food (contain many fibres) to replace carbohydrate to avoid hunger	Knowledge:<br>Understand food types in terms of fats included<br>Understand unsaturated fats sources<br>Start learning how to cook to take control of meals' components	Knowledge:<br>Understand the role of PA and its impacts in simple language<br>Understand how to do exercise (indoor and outdoor)<br>Understand what the most suitable and enjoyable exercise	Knowledge:<br>Understand the associations between waist circumference and T2DM<br>Understand the recommended target for waist size<br>Understand how to maintain a good size
	Physical	Physical skills	How to measure carbohydrate quantity	NA	Have the energy and the required skills to do PA	Taking the measurement frequently for comparison
Opportunity	Social	Social influences	Having social support, e.g., family members or friends to stay motivated to change the behaviour<br>Preparing your own meal as possible when you outside the house<br>Explain and kindly refuse any meal would hurt your diet (many dates, rice, sweets…etc.)	Having social support, e.g., family members or friends, to stay motivated to change the behaviour<br>Preparing your own meal as possible when you outside the house<br>Explain and kindly refuse any meal that would hurt your diet e.g., unsaturated fats	Having social support, e.g., family members or friends, to stay motivated to change the behaviour<br>Meeting people at parks to walk together	Having social support, e.g., family members or friends, to stay motivated to change the behaviour
	Physical	Environmental context and resources	Avoid eating fast food as possible<br>Buy food that contains many fibres	Avoid eating fast food as possible<br>Buy food that contains unsaturated fats	Register in a fitness centre, if possible, to avoid any environmental restrictions<br>Buy essential PA equipment, if possible, to avoid any environmental restrictions<br>Have daily time for PA<br>Do the most suitable and enjoyable exercise	Have a tape to measure waist circumference

Restricted - مقيد

COM-B		TDF coding included in this research	What needs to occur to bring about change?			
			Decrease carbohydrate intake in each meal	Use unsaturated fats as possible (avoid saturated fats)	Do exercise for 30 min, five days on a weekly basis	Monitor waist circumference
Motivation	Reflective	Beliefs about own capability<br><br>Beliefs consequences<br><br>Social role and identity	Beliefs about own capability:<br><br>Have willpower and self-confidence in the ability to make the change<br><br>Have alternatives to cope with financial incapability<br><br>Beliefs about consequences:<br><br>Understand the negative impacts of much carbohydrate intake<br><br>Understand the risk factors for developing other diseases<br><br>Social Role and Identity:<br><br>Address the social barrier (carbohydrate portion size, like using a spoon)	Beliefs about own capability:<br><br>Have willpower and self-confidence in the ability to make the change<br><br>Have alternatives to cope with financial incapability<br><br>Beliefs about consequences: Understand the negative impacts of food containing saturated fats<br><br>Understand the risk factors of having saturated fats in developing other diseases<br><br>Social Role and Identity:<br><br>Address the social barrier (using unsaturated fats for cooking)	Beliefs about own capability:<br><br>Have willpower and self-confidence in the ability to do the exercise<br><br>Beliefs about consequences:<br><br>Overcome the fears of injury<br>Understand the risk factors of being physically inactive<br><br>Social Role and Identity:<br><br>Address the social barrier (overcome social barriers related to PA)	Change self-view of waist circumferences<br>Beliefs about consequences:<br>Understand the significance of accomplishing the waist circumference target

For peer review only

Restricted - مقيد

Supplemental Table 3: Intervention operation protocol

For peer review only

Restricted - مقيد

Phase #	*Session Content*	*Session Goals*	*Intervention function*
**1**	• Session #1, the patient's assessment form and consent supposed to be completed<br>• General introduction about the health coaching intervention<br>• Outline the intervention structure and content<br>• Discuss the coach's roles and the expectations from the participant (being completely clear with the client about the health coaching)<br>• Creating an alliance (Establish Trust)<br>• Learn from a patient (diseases history, obstacles, priorities, strengths, goals... etc)<br>• Help patient to create wellness vision<br>• Assess the readiness of patient's stage in relation to change health behaviour (the transtheoretical model)<br>• Introduce the importance of having a healthy diet<br>• Introduce the importance of increasing physical activity<br>• Increase awareness of adopting a healthy lifestyle in relation to controlling diabetes<br>• Identify 3-month general behavioural goals, and biweekly goals<br>• Explore resources needed to help achieve desirable behaviour<br>• Commitment	• Identifying the patient's current position in the overall health status (via the transtheoretical model)<br>• Build up a relationship (as an ally) between patient and coach<br>• Encourage open discussion<br>• Make sure the patient understands what health coaching is<br>• Allow the patient to develop a foundational conception of goal setting and action planning<br>• The patient creates (SMART) Specific, Measurable, Action-based, Realistic and Timely achievable goals<br>• Have better control of carbohydrate and fat intake<br>• Gradually increase physical activity<br>• Gradually increase achievable tasks until the intended behaviour is achieved	• Education<br>• Enablement<br>• Training<br>• Restriction<br>• Environmental restructuring
**2**	• Phase #2 (session # 2), (this phase will be used again in session # 4 & 5)<br>• Check ongoing progresses<br>• Understand patient's state (use reflections)<br>• Ask patient to share views (so far) good things occurred and experience from last session<br>• Use positive reflections about patient's strengths, passion or emotions<br>• Ask patient to assess the previous selected short-goals and accomplishments<br>• Use reflections to understand potential barriers prevent patient from achieving past goals<br>• Identify specific strategies that they may use to overcome the obstacles<br>• Explore what patient learned from past experience<br>• Ask and discuss with patient next short goals	• Assessment of progression<br>• Review goal setting (behaviour)<br>• Review behaviour goals to examine a patient's performance progression toward the agreed goals<br>• Enable patient to develop problem-solving skills<br>• Enable patient to create action plan<br>• Prompt the participant to generate ideas and strategies to overcome barriers (problem-solving)<br>• Allow patients to monitor their behaviours (know the changes so far)<br>• Keep patient motivated (no matter the accomplishments)	• Persuasion<br>• Education<br>• Enablement<br>• Training<br>• Restriction<br>• Environmental restructuring

review only

Restricted - مقيد

	• Share feedback on patient's progression<br>• Ask patient to connect current accomplishments to the general 3-montπhs goals<br>• Review the general goals to see if patient want to revise them (to be more realistic and achievable)<br>• Affirm the patient's choices, strengths, and capability<br>• Use techniques such as reflective listening to address ambivalence and respond to the patient's resistance (motivational interviewing)		
3	• Middle phase (session # 3), the coach continues to observe the patient and give feedback to help them move forward in achieving their goals through bi-weekly SMART goal setting.<br>• Patient continues to identify strategies to address existing obstacles and enhance their self-ability<br>• If goals are not achieved, the barriers will be identified, action plan will be taken to address these obstacles and modified goals will be cearted.<br>• The coach will assess the patient's self-efficacy by scoring goals to measure the participant's confidence in achieving their goals<br>• The coach continues using the skills needed to explore ambivalence and discrepancies between the participant's plans and their actual behaviour (Rollnick et al, 2005)<br>• Affirmations and appreciative inquiry will be used to appreciate progression and improve patients' self- confidence	• Assessment of current behavioural change<br>• Review goal setting (behaviour)<br>• Review behaviour goals to examine a patient's performance progression toward the agreed goals<br>• Review all previous goals and reassess goal progress<br>• Enable the participant to assess their progress<br>• Prompt the participant to analyse factors influencing their behaviour<br>• Participant's commitment to affirm to review and change behaviour	• Persuasion<br>• Education<br>• Enablement<br>• Training
4	• (session # 6)<br>• Conclude the coaching relationship<br>• Determine where the patient is in terms of their goals<br>• How the coach can best guide the client, and whether coaching is what will best benefit the client<br>• Participants' assessment of the intervention, general satisfaction of the participant with the process<br>• Appreciate the patient's engagement in the intervention<br>• Explore the patients' experience and how future coaching intervention would best support T2DM	• Allow patient to explore the difference at the endpoint<br>• Learn for participants' experience<br>• Findings from the pilot study will be used to justify an expansion of the study (full-scale) or refined for better outcomes so that we can carry out a large RCT on the efficacy of this intervention	• Persuasion<br>• Education

Restricted - مقيد

Supplemental Table 4: Joint display table

Key Concepts	Progression Criteria	Cut-off Results	Quantitative Results	Qualitative Results	Integration Result	Integration result (Morgan, 2013).	Meta-inference
**Screening prospective participants**	Consent rate ≥60-80% endorses RCT continuation; <50% disqualifies further action.	Achieved >80%	Screening rate: 90% (38/42 eligible)	-	Screening efficacy confirmed; criteria surpassed.	=	Convergent
**Recruitment rate**	Recruitment ≥80% validates RCT continuation; 65-70% prompts review and potential adjustments; ≤65% halts progress.	Achieved 70-80%	Recruitment rate: 79% (30/38 consented)	Recruitment feasible; interest in extending participation noted.	Recruitment nearly meets criteria; positive qualitative reinforcement.	=	Convergent
**Retention rate at 3-months**	Retention ≥83% supports RCT scale-up; <83% precludes further action.	Achieved >83%	Retention rate: 97% (29/30 completed)	One withdrawal due to family concern; commitment to participation noted.	High retention despite COVID-19 constraints.	=	Convergent
**Intervention adherence**	Adherence ≥84% signals RCT progression; 67-84% warrants discussion; <67% stops trial.	Achieved >84%	100% adherence	High satisfaction; suggestions for more/flexible sessions.	Adherence exceeds criteria; qualitative data affirms.	=	Convergent
**Secondary measurements acceptability**	-	-	Baseline: 100%; Endpoint: 96.6%	Assessments deemed easy, straightforward.	High adherence to data collection; assessments suitable.	=	Convergent

For peer review only

Restricted - مقيد

**Intervention preliminary efficacy and tangible effects**	-	-	Improvements in HbA1C, weight, BMI, waist circumference.	Positive participant feedback on tangible health benefits.	Qualitative feedback aligns with quantitative improvements.	=	Convergent
**Intervention experience**	-	-	Session length and BCT usage detailed.	Intervention fit for context; skills acquisition noted.	Intervention delivery and curriculum effective.	=	Convergent
**Satisfaction with the intervention**	-	-	High satisfaction rates (8.14-9.21/10).	Strong participant endorsements; suggestions for expansion.	Participant satisfaction confirmed; qualitative concurrence.	=	Convergent
**Flexibility in scheduling coaching sessions**	-	-	13% of sessions rescheduled.	Flexibility aided adherence but increased coach burden.	Flexibility supported participants; coach challenges noted.	=	Convergent

For peer review only

Restricted - مقيد

Supplemental Table 5: Time spent in each coaching session.

Participant #	Gender	Age	Session#						TOTAL (minutes)	Mean (minutes)
			1	2	3	4	5	6		
**P01**	Female	50-59	35	22	12	15	12	37	133	22.2
**P06**	Female	50-59	27	18	17	14	17	25	118	19.7
**P09**	Male	60-69	29	17	10	14	12	24	106	17.7
**P10**	Male	50-59	30	12	14	13	12	30	111	18.5
**P03**	Female	40-49	32	19	19	12	11	33	126	21
**P04**	Female	50-59	27	15	11	15	10	35	113	18.8
**P07**	Male	60-69	33	11	10	14	11	30	109	18.2
**P02**	Female	50-59	35	12	10	11	10	33	111	18.5
**P05**	Female	40-49	31	14	11	12	10	30	108	18
**P11**	Male	30-39	32	18	16	12	15	40	133	22.2
**P08**	Male	50-59	35	13	15	10	12	31	116	19.3
**P14**	Female	50-59	40	13	11	12	10	31	117	19.5

For peer review only

Restricted - مقيد

**P15**	Female	40-49	44	22	10	15	12	34	137	22.8
**P13**	Male	40-49	43	15	13	17	15	50	153	25.5
**TOTAL (minutes)**			473	221	179	186	169	463	1691	
**Mean**			33.8	15.8	12.8	13.3	12.1	33.1		
**Median**			32.5	15	11.5	13.5	12	32		
**Range**			17 (27-44)	11 (12-22)	9 (10-19)	7 (10-17)	7 (10-17)	26 (24-50)		

For peer review only

Restricted - مقيد

Supplemental Table 6: Feasibility Measurements and Predetermined progression criteria

Measure	Definition	Result	Predetermined progression criteria	Notes
**Screening rate**	Number of screened participants that met the inclusion criteria	38 out of 42 (91%)	> 80% screening rate (this suggests proceeding to the future definitive RCT)	The target approached participants was adjusted due to the COVID-19 pandemic
**Recruitment rate**	Number of recruited participants out of the total participants who were eligible during recruited phase	30 out of 30 (100%)	> 80% screening rate (this suggests proceeding to the future definitive RCT)	Despite the COVID-19 epidemic, the recruitment rate standard was reached
**Retention rate at 3-months**	Number of participants who completed the trial out of the total sample	29 out of 30 (96.6%)	> 83 % retention rate at Three months (this suggests proceeding to the future definitive RCT)	The retention rate at 3-months was high (all participants who took the first session have completed all six sessions)
**Baseline data collection adherence**	Number of participants who completed the assessments at the intervention baseline out of the total study sample	30 out of 30 (100%)		All participants have completed study assessments at the baseline
**Intervention adherence (coaching group only)**	Number of participants who completed 5 out of six coaching sessions ≥ 84%	6 out of 6 sessions per participant (100%)	>84% of the intervention (≥ 5 out of the six coaching sessions) (this suggests proceeding to the future definitive RCT)	
**Endpoint data collection adherence**	Number of participants who completed the assessments at the intervention endpoint out of the total study sample	29 out of 30 participants (96.6%)		Only one participant has withdrawn before the first session

For peer review only - http://bmjopen.bmj.com/site/about/guidelines.xhtml

Restricted - مقيد

Supplemental Table 7: Participant ratings of health coaching on a 10-Point Likert Scale (intervention group only)

Question	N	Mean [a]	Std. Deviation	Range
To what extent did participation in health coaching:				
1. Changed you/your behaviour	14	8.14	2.381	3-10
2. Helped create a personal vision of wellness	14	9.21	1.369	6-10
3. Increase your confidence to take steps to greater wellness	14	9.21	1.369	6-10
4. Increase your motivation to take steps to greater wellness	14	9.14	1.351	6-10
5. Increase your use of goal-setting skills	14	9.21	.893	7-10
6. Increase your use of problem-solving skills	14	8.57	1.399	5-10
7. Help get you back on track after running into barriers	14	9.07	1.072	7-10
8. Help you achieve greater wellness	14	9.14	1.099	7-10

[a] Scale of 0 to 10, with 0 (not at all) to 10 (very much)

For peer review only

Restricted - مقيد

Supplemental Table 8: Frequency of used Behavioural Change Techniques (BCTs) to each behaviour target

	BCT	Behaviour target (s)	Frequency Used
1.	*1.1 Goal setting (Behaviour)*	• Decrease carbohydrate intake for each meal<br>• Use unsaturated fats as possible (avoid saturated fats)<br>• Do exercise for 30 min, five days on a weekly basis<br>• Monitor waist circumference	43
2.	*1.2 Problem-solving*	• Decrease carbohydrate intake for each meal<br>• Use unsaturated fats as possible (avoid saturated fats)<br>• Do exercise for 30 min, five days on a weekly basis	28
3.	*1.3 Goal setting (outcome)*	• Decrease carbohydrate intake for each meal<br>• Do exercise for 30 min, five days on a weekly basis<br>• Monitor waist circumference	34
4.	*1.4 Action planning*	• Decrease carbohydrate intake for each meal<br>• Do exercise for 30 min, five days on a weekly basis	27

For peer review only

Restricted - مقيد

	BCT	Behaviour target (s)	Frequency Used
5.	*1.5 Review behaviour goal (s)*	• Decrease carbohydrate intake for each meal<br>• Do exercise for 30 min, five days on a weekly basis	34
6.	*1.7 Review outcome goal (s)*	• Decrease carbohydrate intake for each meal<br>• Do exercise for 30 min, five days on a weekly basis	39
7.	*1.9 Commitment*	• Decrease carbohydrate intake for each meal<br>• Use unsaturated fats as possible (avoid saturated fats)<br>• Do exercise for 30 min, five days on a weekly basis	18
8.	*2.2 Feedback on behaviour*	• Decrease carbohydrate intake for each meal	17
9.	*2.3 Self-monitoring of behaviour*	• Decrease carbohydrate intake for each meal<br>• Do exercise for 30 min, five days on a weekly basis	15
10.	*2.7. Feedback on outcome(s) of behaviour*	• Decrease carbohydrate intake for each meal<br>• Use unsaturated fats as possible (avoid saturated fats)<br>• Do exercise for 30 min, five days on a weekly basis<br>• Monitor waist circumference	22

For peer review only

Restricted - مقيد

	BCT	Behaviour target (s)	Frequency Used
11.	*3.1 Social support (unspecified)*	• Decrease carbohydrate intake for each meal<br>• Use unsaturated fats as possible (avoid saturated fats)<br>• Do exercise for 30 min, five days on a weekly basis	29
12.	*4.1 Instruction on how to perform a behaviour*	• Decrease carbohydrate intake for each meal<br>• Do exercise for 30 min, five days on a weekly basis<br>• Monitor waist circumference	14
13.	*5.1Information about health consequences*	• Decrease carbohydrate intake for each meal<br>• Use unsaturated fats as possible (avoid saturated fats)<br>• Do exercise for 30 min, five days on a weekly basis<br>• Monitor waist circumference	27
14.	*5.3 Information about social and environmental consequences*	• Decrease carbohydrate intake for each meal<br>• Use unsaturated fats as possible (avoid saturated fats)<br>• Do exercise for 30 min, five days on a weekly basis	19
15.	*5.5 Anticipated regret*	• Decrease carbohydrate intake for each meal<br>• Do exercise for 30 min, five days on a weekly basis	15

For peer review only - http://bmjopen.bmj.com/site/about/guidelines.xhtml
Restricted - مقيد

	BCT	Behaviour target (s)	Frequency Used
16.	*5.6 Information about emotional consequences*	• Decrease carbohydrate intake for each meal<br>• Do exercise for 30 min, five days on a weekly basis	11
17.	*6.1 Demonstration of the behaviour*	• Decrease carbohydrate intake for each meal<br>• Use unsaturated fats as possible (avoid saturated fats)<br>• Do exercise for 30 min, five days on a weekly basis	14
18.	*6.3 Information about others' approval*	• Decrease carbohydrate intake for each meal<br>• Use unsaturated fats as possible (avoid saturated fats)<br>• Do exercise for 30 min, five days on a weekly basis	14
19.	*8.1 Behavioural practice/rehearsal*	• Decrease carbohydrate intake for each meal	22
20.	*8.3 Habit Formation*	• Decrease carbohydrate intake for each meal<br>• Use unsaturated fats as possible (avoid saturated fats)<br>• Do exercise for 30 min, five days on a weekly basis	28
21.	*8.7 Graded tasks*	• Decrease carbohydrate intake for each meal<br>• Use unsaturated fats as possible (avoid saturated fats)<br>• Do exercise for 30 min, five days on a weekly basis	31

For peer review only

Restricted - مقيد

	BCT	Behaviour target (s)	Frequency Used
22.	*9.1Credible source*	• Decrease carbohydrate intake for each meal<br>• Do exercise for 30 min, five days on a weekly basis	17
23.	*9.2 Pros and cons*	• Decrease carbohydrate intake for each meal<br>• Use unsaturated fats as possible (avoid saturated fats)<br>• Do exercise for 30 min, five days on a weekly basis	15
24.	*7.1 Prompts/Cues*	• Decrease carbohydrate intake for each meal<br>• Do exercise for 30 min, five days on a weekly basis	9
25.	*9.3 Comparative imagining of future outcomes*	• Decrease carbohydrate intake for each meal<br>• Use unsaturated fats as possible (avoid saturated fats)<br>• Do exercise for 30 min, five days on a weekly basis<br>• Monitor waist circumference	27
26.	*10.9 Self-reward*	• Decrease carbohydrate intake for each meal<br>• Do exercise for 30 min, five days on a weekly basis	8
27.	*12.1 Restructuring the physical environment*	• Decrease carbohydrate intake for each meal<br>• Use unsaturated fats as possible (avoid saturated fats)<br>• Do exercise for 30 min, five days on a weekly basis	26

For peer review only

Restricted - مقيد

	BCT	Behaviour target (s)	Frequency Used
28.	*12.2 Restructuring the social environment*	• Decrease carbohydrate intake for each meal<br>• Use unsaturated fats as possible (avoid saturated fats)<br>• Do exercise for 30 min, five days on a weekly basis	28
29.	*12.3Avoidance/reducing exposure to cues for the behaviour*	• Decrease carbohydrate intake for each meal<br>• Use unsaturated fats as possible (avoid saturated fats)	18
30.	*12.5 Adding objects to the environment*	• Decrease carbohydrate intake for each meal<br>• Use unsaturated fats as possible (avoid saturated fats)<br>• Do exercise for 30 min, five days on a weekly basis	16
31.	*13.1 Identification of self as role model*	• Decrease carbohydrate intake for each meal<br>• Use unsaturated fats as possible (avoid saturated fats)<br>• Do exercise for 30 min, five days on a weekly basis	14
32.	*13.2 Framing/reframing*	• Decrease carbohydrate intake for each meal<br>• Do exercise for 30 min, five days on a weekly basis	16

For peer review only

Restricted - مقيد

	BCT	Behaviour target (s)	Frequency Used
33.	*15.1 Verbal persuasion about capability*	• Decrease carbohydrate intake for each meal<br>• Use unsaturated fats as possible (avoid saturated fats)<br>• Do exercise for 30 min, five days on a weekly basis<br>• Monitor waist circumference	30
34.	*15.2 Mental rehearsal of successful performance*	• Decrease carbohydrate intake for each meal<br>• Do exercise for 30 min, five days on a weekly basis	17
35.	*15.3 Focus on past success*	• Decrease carbohydrate intake for each meal<br>• Use unsaturated fats as possible (avoid saturated fats)<br>• Do exercise for 30 min, five days on a weekly basis	24
36.	*15.4 Self-talk*	• Decrease carbohydrate intake for each meal	7

For peer review only

Restricted - مقيد

Supplemental Table 9: Participants' mean differences in each group at baseline and endpoint for patients' self-efficacy questionnaire

	Group	N	Mean	Std. Deviation	An independent two-sample t-test: Sig. (2-tailed)	Mean Difference	95% Confidence Interval of the Difference	
How confident do you feel that you can eat your meals every 4 to 5 hours every day, including breakfast every day? (At the baseline)	Intervention	14	5.36	3.296	.445	.757	-1.249	2.763
	Control	15	4.60	1.805				
How confident do you feel that you can eat your meals every 4 to 5 hours every day, including breakfast every day? (At endpoint)	Intervention	14	7.21	2.082	.505	.481	-.981	1.943
	Control	15	6.73	1.751				
How confident do you feel that you can follow your diet when you have to prepare or share food with other people who do not have diabetes? (At the baseline)	Intervention	14	5.29	3.688	.891	.152	-2.114	2.419
	Control	15	5.13	2.100				
How confident do you feel that you can follow your diet when you have to prepare or share food with other people who do not have diabetes? (At endpoint)	Intervention	14	8.07	1.979	.274	.871	-.732	2.474
	Control	15	7.20	2.210				
How confident do you feel that you can choose the appropriate foods to eat when you are hungry (for example, snacks)? (At the baseline)	Intervention	14	6.07	3.174	.569	.538	-1.379	2.455
	Control	15	5.53	1.685				

Restricted - مقيد

How confident do you feel that you can choose the appropriate foods to eat when you are hungry (for example, snacks)? (At endpoint)	Intervention	14	8.21	2.082	.144	1.014	-.368	2.397
	Control	15	7.20	1.521				
How confident do you feel that you can exercise 15 to 30 minutes, 4 to 5 times a week? (At the baseline)	Intervention	14	6.50	3.632	.217	1.500	-.936	3.936
	Control	15	5.00	2.726				
How confident do you feel that you can exercise 15 to 30 minutes, 4 to 5 times a week? (At endpoint)	Intervention	14	9.14	1.994	.006	2.276	.713	3.839
	Control	15	6.87	2.100				
How confident do you feel that you can do something to prevent your blood sugar level from dropping when you exercise? (At the baseline)	Intervention	14	6.71	3.148	.072	1.848	-.178	3.873
	Control	15	4.87	2.100				
How confident do you feel that you can do something to prevent your blood sugar level from dropping when you exercise? (At endpoint)	Intervention	14	8.71	2.054	.000	3.048	1.493	4.602
	Control	15	5.67	2.024				
How confident do you feel that you know what to do when your blood sugar level goes higher or lower than it should be? (At the baseline)	Intervention	14	5.71	2.867	.616	.448	-1.360	2.256
	Control	15	5.27	1.792				
How confident do you feel that you know what to do when your blood sugar level goes higher or lower than it should be? (At endpoint)	Intervention	14	8.36	2.023	.006	2.157	.678	3.636
	Control	15	6.20	1.859				
	Intervention	14	6.93	2.786	.284	.995	-.872	2.862

Restricted - مقيد

How confident do you feel that you can judge when the changes in your illness mean you should visit the doctor? (At the baseline)	Control	15	5.93	2.086				
How confident do you feel that you can judge when the changes in your illness mean you should visit the doctor? (At endpoint)	Intervention	14	9.43	1.016	.000	3.295	2.075	4.516
	Control	15	6.13	1.995				
How confident do you feel that you can control your diabetes so that it does not interfere with the things you want to do? (At the baseline)	Intervention	14	5.93	3.125	.660	.462	-1.668	2.592
	Control	15	5.47	2.446				
How confident do you feel that you can control your diabetes so that it does not interfere with the things you want to do? (At endpoint)	Intervention	14	8.86	2.033	.006	2.590	.826	4.355
	Control	15	6.27	2.549				

view only

Restricted - مقيد

Supplemental Table 10: Participants' mean differences in each group at baseline and endpoint for diabetes self-care activity questionnaire

					An independent two-sample t-test			
	Group	N	Mean	Std. Deviation	Sig. (2-tailed)	Mean Difference	95% Confidence Interval of the Difference	
On average, over the past month, how many days per week have you followed your eating plan? (At the baseline)	Intervention	14	2.36	2.098	.461	-.510	-1.909	.890
	Control	15	2.87	1.552				
On average, over the past month, how many days per week have you followed your eating plan? (At endpoint)	Intervention	14	4.86	1.610	.160	.790	-.333	1.914
	Control	15	4.07	1.335				
On how many of the last seven days did you eat five or more servings of fruits and vegetables? (At the baseline)	Intervention	14	2.29	1.590	.063	-1.181	-2.431	.069
	Control	15	3.47	1.685				
On how many of the last seven days did you eat five or more servings of fruits and vegetables? (At endpoint)	Intervention	14	3.93	1.385	.100	-.871	-1.923	.180
	Control	15	4.80	1.373				
On how many of the last seven days did you eat high fat foods such as red meat or full-fat dairy products? (At the baseline)	Intervention	14	2.50	1.912	.046	-1.167	-2.311	-.022
	Control	15	3.67	.976				
On how many of the last seven days did you eat high fat foods such as red meat or full-fat dairy products? (At endpoint)	Intervention	14	3.50	1.829	.323	-.633	-1.923	.656
	Control	15	4.13	1.552				
On how many of the last seven days did you space carbohydrates evenly through the day? (At the baseline)	Intervention	14	1.71	1.729	.236	-.686	-1.845	.474
	Control	15	2.40	1.298				
On how many of the last seven days did you space carbohydrates evenly through the day? (At endpoint)	Intervention	14	4.00	1.240	.258	-.533	-1.481	.415

Restricted - مقيد

	Control	15	4.53	1.246				
On how many of the last seven days have you followed a healthful eating plan? (At the baseline)	Intervention	14	1.64	1.447	.011	-1.490	-2.617	-.364
	Control	15	3.13	1.506				
On how many of the last seven days have you followed a healthful eating plan? (At endpoint)	Intervention	14	5.00	1.468	.452	.400	-.674	1.474
	Control	15	4.60	1.352				
On how many of the last seven days did you participate in at least 30 minutes of physical activity? (At the baseline)	Intervention	14	3.57	2.243	.674	.305	-1.165	1.775
	Control	15	3.27	1.580				
On how many of the last seven days did you participate in at least 30 minutes of physical activity? (At endpoint)	Intervention	14	5.64	1.692	.162	.776	-.331	1.884
	Control	15	4.87	1.187				
On how many of the last seven days did you participate in a specific exercise session (such as such swimming, walking, biking) other than what you do around the house or as part of your work? (At the baseline)	Intervention	14	2.29	2.016	.775	-.181	-1.465	1.103
	Control	15	2.47	1.302				
On how many of the last seven days did you participate in a specific exercise session (such as such swimming, walking, biking) other than what you do around the house or as part of your work? (At endpoint)	Intervention	14	4.93	2.018	.145	.929	-.342	2.199
	Control	15	4.00	1.254				

Restricted - مقيد

CONSORT 2010 checklist of information to include when reporting a pilot or feasibility trial*

Section/Topic	Item No	Checklist item	Reported on page No
**Title and abstract**			
	1a	Identification as a pilot or feasibility randomised trial in the title	1
	1b	Structured summary of pilot trial design, methods, results, and conclusions (for specific guidance see CONSORT abstract extension for pilot trials)	1
**Introduction**			
Background and objectives	2a	Scientific background and explanation of rationale for future definitive trial, and reasons for randomised pilot trial	2
	2b	Specific objectives or research questions for pilot trial	3
**Methods**			
Trial design	3a	Description of pilot trial design (such as parallel, factorial) including allocation ratio	3
	3b	Important changes to methods after pilot trial commencement (such as eligibility criteria), with reasons	4
Participants	4a	Eligibility criteria for participants	4
	4b	Settings and locations where the data were collected	4
	4c	How participants were identified and consented	4
Interventions	5	The interventions for each group with sufficient details to allow replication, including how and when they were actually administered	4-5
Outcomes	6a	Completely defined prespecified assessments or measurements to address each pilot trial objective specified in 2b, including how and when they were assessed	5
	6b	Any changes to pilot trial assessments or measurements after the pilot trial commenced, with reasons	5
	6c	If applicable, prespecified criteria used to judge whether, or how, to proceed with future definitive trial	5
Sample size	7a	Rationale for numbers in the pilot trial	5
	7b	When applicable, explanation of any interim analyses and stopping guidelines	n/a
Randomisation:			
Sequence generation	8a	Method used to generate the random allocation sequence	4
	8b	Type of randomisation(s); details of any restriction (such as blocking and block size)	4
Allocation concealment mechanism	9	Mechanism used to implement the random allocation sequence (such as sequentially numbered containers), describing any steps taken to conceal the sequence until interventions were assigned	4

For peer review only - http://bmjopen.bmj.com/site/about/guidelines.xhtml

Implementation	10	Who generated the random allocation sequence, who enrolled participants, and who assigned participants to interventions	4
Blinding	11a	If done, who was blinded after assignment to interventions (for example, participants, care providers, those assessing outcomes) and how	n/a
	11b	If relevant, description of the similarity of interventions	n/a
Statistical methods	12	Methods used to address each pilot trial objective whether qualitative or quantitative	6
**Results**			
Participant flow (a diagram is strongly recommended)	13a	For each group, the numbers of participants who were approached and/or assessed for eligibility, randomly assigned, received intended treatment, and were assessed for each objective	9
	13b	For each group, losses and exclusions after randomisation, together with reasons	9-10
Recruitment	14a	Dates defining the periods of recruitment and follow-up	9
	14b	Why the pilot trial ended or was stopped	n/a
Baseline data	15	A table showing baseline demographic and clinical characteristics for each group	8
Numbers analysed	16	For each objective, number of participants (denominator) included in each analysis. If relevant, these numbers should be by randomised group	10
Outcomes and estimation	17	For each objective, results including expressions of uncertainty (such as 95% confidence interval) for any estimates. If relevant, these results should be by randomised group	19-20
Ancillary analyses	18	Results of any other analyses performed that could be used to inform the future definitive trial	13
Harms	19	All important harms or unintended effects in each group (for specific guidance see CONSORT for harms)	n/a
	19a	If relevant, other important unintended consequences	n/a
**Discussion**			
Limitations	20	Pilot trial limitations, addressing sources of potential bias and remaining uncertainty about feasibility	24
Generalisability	21	Generalisability (applicability) of pilot trial methods and findings to future definitive trial and other studies	24
Interpretation	22	Interpretation consistent with pilot trial objectives and findings, balancing potential benefits and harms, and considering other relevant evidence	24
	22a	Implications for progression from pilot to future definitive trial, including any proposed amendments	23-24
**Other information**			
Registration	23	Registration number for pilot trial and name of trial registry	n/a
Protocol	24	Where the pilot trial protocol can be accessed, if available	5
Funding	25	Sources of funding and other support (such as supply of drugs), role of funders	n/a
	26	Ethical approval or approval by research review committee, confirmed with reference number	26

For peer review only - http://bmjopen.bmj.com/site/about/guidelines.xhtml

Citation: Eldridge SM, Chan CL, Campbell MJ, Bond CM, Hopewell S, Thabane L, et al. CONSORT 2010 statement: extension to randomised pilot and feasibility trials. BMJ. 2016;355. This is an Open Access article distributed in accordance with the terms of the Creative Commons Attribution (CC BY 3.0) license (http://creativecommons.org/licenses/by/3.0/), which permits others to distribute, remix, adapt and build upon this work, for commercial use, provided the original work is properly cited.

*We strongly recommend reading this statement in conjunction with the CONSORT 2010, extension to randomised pilot and feasibility trials, Explanation and Elaboration for important clarifications on all the items. If relevant, we also recommend reading CONSORT extensions for cluster randomised trials, non-inferiority and equivalence trials, non-pharmacological treatments, herbal interventions, and pragmatic trials. Additional extensions are forthcoming: for those and for up-to-date references relevant to this checklist, see www.consort-statement.org.

For peer review only

For peer review only - http://bmjopen.bmj.com/site/about/guidelines.xhtml
